# Time series smoother for effect detection

**DOI:** 10.1371/journal.pone.0195360

**Published:** 2018-04-23

**Authors:** Cheng You, Dennis K. J. Lin, S. Stanley Young

**Affiliations:** 1 Department of Statistics, Pennsylvania State University, State College, PA, United States of America; 2 CGStat LLC, Raleigh, NC, United States of America; Cleveland Clinic Lerner Research Institute, UNITED STATES

## Abstract

In environmental epidemiology, it is often encountered that multiple time series data with a long-term trend, including seasonality, cannot be fully adjusted by the observed covariates. The long-term trend is difficult to separate from abnormal short-term signals of interest. This paper addresses how to estimate the long-term trend in order to recover short-term signals. Our case study demonstrates that the current spline smoothing methods can result in significant positive and negative cross-correlations from the same dataset, depending on how the smoothing parameters are chosen. To circumvent this dilemma, three classes of time series smoothers are proposed to detrend time series data. These smoothers do not require fine tuning of parameters and can be applied to recover short-term signals. The properties of these smoothers are shown with both a case study using a factorial design and a simulation study using datasets generated from the original dataset. General guidelines are provided on how to discover short-term signals from time series with a long-term trend. The benefit of this research is that a problem is identified and characteristics of possible solutions are determined.

## Introduction

In environmental epidemiology, how the variations in the environment affect human health is of great interest. In particular, do daily fluctuations in air quality induce fluctuations in human mortality? The United States Environmental Protection Agency claims that small particulate matter, PM_2.5_, can cause acute death and would enforce cleaner energy production by new regulations. Although their claim should be evaluated with some caution, the air quality problem not only affects long-term human health but also poses a major economic challenge to the United States and many other countries.

While the air quality study is a broad question, whether a higher amount of ozone in the ambient air would associate with higher mortality is an important concern. The mortality data available are typically aggregated counts at different time scales. These count data display certain long-term trend including seasonality over time at any spatial location, see [1]. The long-term trend cannot be completely adjusted by the observed weather covariates. Besides, there are other issues discussed in [2], such as lag effects, i.e. what happens in today’s air quality can have an impact on the mortality tomorrow or the day after tomorrow.

To estimate the long-term trend with seasonality, smoothing methods are proposed, which are very often embedded in a semi-parametric time series Poisson regression model. In [3], the LOESS smooth functions of covariates including time was suggested in generalized additive Poisson models of air quality variables and human mortality. The smooth function of time is used to remove any long-term trend including seasonality in both the air quality and mortality time series. Thereafter, several alternative smoothing methods were proposed, such as parametric natural splines and penalized splines in [4], [5], [6], [7], [8], [9] and [10]. The conclusions are drawn based on these embedded time series Poisson regression models and a significantly positive association between the air quality variable of interest and human mortality is often found. However, this result is subject to the degree of detrending in multiple time series data. Therefore, we intend to conduct a comprehensive investigation of the detrending process in order to understand and suggest how multiple time series with a long-term trend, including seasonality, should be detrended.

Human mortality, excluding accidental deaths, can be attributed to various reasons, from either latent variables or air quality fluctuations. It is crucial to separate the short-term signals from the long-term trend to study any short-term effect. However, the long-term trend including seasonality in either human mortality or an air quality variable is not precisely known. Overly rough functions of time can be too harsh on removing trends, even local fluctuations, and thus leave very little short-term signals in the deviations for the detection of short-term effect; overly smooth functions of time can be too lenient on removing trends and thus leave much long-term trend in the deviations, which overwhelms any short-term effect of human mortality and air quality. We have found that including smoothing functions of time can possibly introduce spurious correlation. The current spline smoothing without examining the variability of smooth functions can be problematic in detrending multiple time series. It will be shown that by varying the smoothing parameters, any significant result, from negative to positive, can be obtained. This excessive modeling flexibility could undermine the plausibility of any air quality study.

In [11], natural spline smoothing and penalized spline smoothing were studied and model-based simulations showed that under moderate concurvity, an analog of multicollinearity, smoother spline of air quality variable can lead to less confounding bias. In [12], an overview of generalized additive models based on the penalized likelihood approach with regression splines was provided. In [13], efficient smoothing parameter estimation was claimed to be performed and reduced rank spline smoothing methods were demonstrated for large datasets. In [14], Bayesian model averaging with generalized additive mixed models was proposed to address the modeling uncertainty. Nonetheless, different model selection criteria can lead to different smoothness; the smoothness of a certain spline is usually vaguely described and hardly defined.

To circumvent this problem, we propose robust and stable nonparametric smoothing methods that can be specified before analysis without undue experimentation and separate the long-term trend and the local short-term signals. After smoothing, the deviations, which are the raw time series subtracting the estimated long-term trend, are examined. These deviations contain the local information useful for investigating the short-term association between human mortality and air quality fluctuations, namely the acute effect.

For our case study, the illustrative data are obtained from [15]. This dataset contains the time series of daily human mortality, air quality and weather covariates in Los Angeles California, from 2000 to 2012. The mortality data were obtained from California Department of Public Health. The air quality data PM_2.5_ and ozone were downloaded from California Environmental Protection Agency. The temperature data were downloaded from Carbon Dioxide Information Analysis Center of United States Historical Climatology Network and the relative humidity data were from the United States Environmental Protection Agency. For our simulation study, the synthetic datasets were generated from first decomposing the original time series into the nominal trend and detrended time series and then recomposing with our pre-specified correlation structure on the detrended time series, which makes that the synthetic data are almost indistinguishable from the real data.

There are three major contributions of our research study. First, it is found that the current spline smoothing methods can obtain different, both positive and negative, effects depending on how the smoothing parameter is varied. Second, the proposed time series smoothers are shown to be robust and stable by the tight confidence intervals of correlation as well as partial correlation, and by the insensitivity of different factors via a factorial design of scenarios. Third, the characteristics of different solutions of time series detrending and association detection are determined by the sensitivity analysis in both our case and simulation studies.

The rest of the paper is organized as follows. In Section Problem Formulation, the formulation of our problem is described in detail. In Section Proposed Methodology, three classes of time series smoothers are proposed with a discussion on their properties. In Section Case Study, the air quality and mortality data in Los Angeles are analyzed by the proposed smoothers with sensitivity analysis, compared to cubic spline smoothing as a representative of spline smoothing methods. In Section Simulation Study, synthetic datasets are generated and the proposed smoothers show their capability of detecting even small acute effects, compared to cubic spline smoothing. In Section Conclusions, the overall conclusion and general recommendations are made.

## Problem Formulation

In air quality studies, mortality count data over time are very often encountered. The common model formulation is a generalized linear model with log link or Poisson regression, as suggested in [16].

$$\begin{array}{c} \begin{array}{cc} y_{t} & ∼Poisson(\mu_{t})\\ log(\mu_{t}) & =\alpha +\beta s_{1}\left(x_{t}\right)+\eta^{\prime }s_{2}\left(z_{t}\right) \end{array} \end{array}$$ (1)

In Eq (1), *y*_*t*_ is the mortality count variable of certain aggregation level at Time *t*. *x*_*t*_ is an air quality variable of interest at Time *t*. **z**_**t**_ is a vector of measured covariates at Time *t*. *s*_1_(⋅) and **s**_**2**_(**⋅**) are a spline smoothing estimator and a vector of such estimators, respectively.

Regarding the above formulation, there are two major concerns. First of all, the long-term trend including seasonality is dominating the overall pattern of the original time series data. Due to the common long-term trend of human mortality and air quality, no positive association between human mortality and air quality variable of interest can be found, even if the observed weather covariates are included for adjustment. In fact, both Type I and Type II errors raise the concerns: claimed effects that are not real but artifacts of the smoothing method, and real effects that fail to be detected because of inappropriate smoothing methods. Second, Poisson regression model assumes that the observations are independent; however, in time series data, the observations close in time are more alike than those distant in time and therefore the autocorrelated structure violates the independence assumption.

To address both concerns, several spline smoothing methods were proposed for time series Poisson regression modeling. In [11], the refined model formulation becomes the following.

$$\begin{array}{c} \begin{array}{cc} y_{t} & ∼Poisson(\mu_{t})\\ log(\mu_{t}) & =\alpha +s_{1}\left(t,\lambda_{1}\right)+\beta x_{t}+\eta^{\prime }s\left(z_{t}\right)\\ x_{t} & =s_{2}\left(t,\lambda_{2}\right)+\xi_{t} \end{array} \end{array}$$ (2)

In Eq (2), *ξ*_*t*_ is the detrended air quality variable of interest at Time *t*. *s*_1_(⋅), *s*_2_(⋅) and **s**(**⋅**) are embedded spline smoothing estimators over time. λ_1_ and λ_2_ are the parameters that control the smoothness of each corresponding spline estimator during the model fitting process. The choice of λ_1_ and λ_2_ can be selected via experimentation and result in rougher or smoother splines *s*_1_(⋅) and *s*_2_(⋅). This arbitrariness makes the resulting model ambiguous for further evaluation.

Hence, how to detrend the long-term trend including seasonality and extract these short-term signals becomes an important issue. We intend to propose a new collection of estimators with robustness and stability. Our model formulation is stated below.

$$\begin{array}{c} \begin{array}{cc} D_{t}^{y} & =y_{t}-m_{1}\left(y_{t}^{*}\right)\\ D_{t}^{x} & =x_{t}-m_{2}\left(x_{t}^{*}\right) \end{array} \end{array}$$ (3)

where $y_{t}^{*}$ and $x_{t}^{*}$ are sets of time series points before and after Time *t* excluding the points in the neighborhood of Time *t*, which are used for the estimation of long-term central tendency at Time *t*; *m*_1_(⋅) and *m*_2_(⋅) are robust and stable estimators of the long-term trend to be defined. Thereafter, certain dependence measures or models are built on the detrended time series. For instance,

$$\begin{array}{c} E\left(D_{t}^{y}|x_{t},z_{t}\right)=\alpha +\beta D_{t}^{x}+\eta^{\prime }z_{t} \end{array}$$ (4)

This can be viewed as a two-stage model so that a researcher can first detrend multiple time series and then analyze the deviations. Both *y*_*t*_ and *x*_*t*_ are detrended respectively so the dominating long-term trend including seasonality is removed without any tuning in the model fitting. Besides, the concurvity between *x*_*t*_ and **z**_**t**_, an analog of multicollinearity, are also eliminated. In a two-stage model, the detrending should happen before Eq (4) is fitted so that there is no iteration back and forth between detrending and fitting.

More precisely, our problem is formulated to find robust and stable time series smoothers such that the long-term trend can be estimated without manipulation of tuning parameters and other observed covariates. The mathematical statement is the first stage of our model formulation. Note that in Eq (3), different *m*_1_(⋅) and *m*_2_(⋅) can result in different $D_{t}^{y}$ and $D_{t}^{x}$. Robustness and stability mean that no matter how the pre-selected parameters or centrality measures change, the cross-association between human mortality and air quality under certain dependence measure is approximately the same.

$$\begin{array}{c} |max\left(\mu \left(D_{t}^{y},D_{t}^{x}\right)\right)-min\left(\mu \left(D_{t}^{y},D_{t}^{x}\right)\right)|<c \end{array}$$ (5)

where *μ*(⋅) is certain dependence measure and *c* is a small constant.

The remaining deviations should not display any long-term pattern except heterogeneity. In other words, when the observations are in the non-volatile period, both $Var(D_{t}^{y})$ and $Var(D_{t}^{x})$ are small constants; when they are in the volatile period, $Var(D_{t}^{y})$ and $Var(D_{t}^{x})$ differ largely. The main purpose of detrending time series is that the deviations contain little information about the long-term trend but all the information of the shock events with a low level of noise. After detrending, the deviations are still non-stationary, but only due to the shock of short-term events, such as forest fires or human interventions, that cause the human mortality or air quality to change drastically. Based on these mild assumptions, the time series decomposition can be written below.

$$\begin{array}{c} x_{t}=l_{t}+\xi_{t}+\epsilon_{t} \end{array}$$ (6)

where *l*_*t*_ is the long-term trend with seasonality, *ξ*_*t*_ is the abnormal signal driven by a short-term event and *ϵ*_*t*_ is the independently and normally distributed random error with mean 0 and variance *σ*^2^, where *σ* is assumed to be much smaller than the abnormal signals. During a non-volatile period, *ξ*_*t*_ is close to 0; during a volatile period, *ξ*_*t*_ displays a certain short-term signal. The ultimate research aim is to discover *ξ*_*t*_ by estimating and eliminating *l*_*t*_.

## Proposed Methodology

To solve the formulated problem in Eq (3), three classes of methods utilizing a centered moving window with a centered gap of varying width are hereby proposed.

The moving window includes the most useful information before and after the time point of interest. The gap at the center can be helpful in retaining any local abnormality in time series data. The width of the window and gap will be discussed later. If there is a strong signal at the time point of interest, the gap reduces the local signal in the smoothed estimate and thus leaves it in the deviation; if not, this window with a centered gap is approximately the same as the ordinary moving window. The points in the gap are excluded to ensure that the short-term signals do not contaminate the trend estimate. The equal number of points inside the center-gapped window before and after the time of interest are used to estimate the long-term trend; this ensures that equal amount of pre and post information is taken. This type of window includes the points on both sides of the time of interest to estimate the central tendency at the time point of interest and the resulting estimate resembles an experimental control. After time series smoothing, the detrended observations can behave as if independently with much less serial correlation and little long-term trend including seasonality left.

To elaborate further, the formulation in Eqs (3) and (6) is utilized for justification. For simplicity, the average with certain window and gap size is selected as the smoothing instrument.

$$\begin{array}{c} \begin{array}{cc} D_{t}^{x} & =x_{t}-\bar{x_{t}^{*}}\\ & =l_{t}+\xi_{t}+\epsilon_{t}-\left(\bar{l_{t}^{*}+\xi_{t}^{*}+\epsilon_{t}^{*}}\right)\\ & =\left(l_{t}-\bar{l_{t}^{*}}\right)+\left(\xi_{t}-\bar{\xi_{t}^{*}}\right)+\left(\epsilon_{t}-\bar{\epsilon_{t}^{*}}\right) \end{array} \end{array}$$ (7)

Here, $x_{t}^{*}$ is the set of time series points outside the neighborhood of Time *t*, excluding Time *t* and the closest points to Time *t*, to estimate the long-term central tendency at Time *t*.

For the first term in the last sum, $\bar{l_{t}^{*}}$ is the interpolation of *l*_*t*_, instead of extrapolation. Compared to the extrapolation, the interpolation should be a good estimate for the long-term trend. Thus, the first term is close enough to 0.

For the second term, there are three cases discussed as follows. If *t* is the volatile time, *ξ*_*t*_ should display a sharp spike while $\bar{\xi_{t}^{*}}$ is near 0 and therefore the second term exposes the shock signal. If *t* is the non-volatile time and the gapped window does not cover the volatile time, *ξ*_*t*_ and $\bar{\xi_{t}^{*}}$ are both near 0 and therefore the second term is close to 0. If *t* is the non-volatile time and the gapped window covers the volatile time, *ξ*_*t*_ is near 0; $\bar{\xi_{t}^{*}}$ slightly deviates from 0 but much smaller than the spike in absolute value. Therefore, the second term slightly deviates from 0; there is a small pattern in this case that reveals a local abnormality, if moving average is used. However, under robust central tendency measures, for instance moving median, this small pattern will become close to 0.

For the third term, since *ϵ*_*t*_ and $\left\{\epsilon_{t}^{*}\right\}$ are independent and identically normal distributed with mean 0 and relatively small variance *σ*^2^, $E(\epsilon_{t}-\bar{\epsilon_{t}^{*}})=0$ and $Var\left(\epsilon_{t}-\bar{\epsilon_{t}^{*}}\right)=\sigma^{2}+\frac{1}{\text{Window}\;\text{Size}-\text{Gap}\;\text{Size}}\sigma^{2}$. $\epsilon_{t}-\bar{\epsilon_{t}^{*}}$ should be close to 0 in most cases. Hence, the remaining term is mainly the shock signal.

In general, $x_{t}^{*}$ is a set of points in the neighborhood of Time *t* with a gap centered at Time *t*.

$$\begin{array}{c} \begin{array}{cc} D_{t}^{x} & =x_{t}-m\left(x_{t}^{*}\right)\\ & \approx l_{t}+\xi_{t}+\epsilon_{t}-m\left(l_{t}^{*}\right)-m\left(\xi_{t}^{*}\right)-m\left(\epsilon_{t}^{*}\right)\\ & =\left(l_{t}-m\left(l_{t}^{*}\right)\right)+\left(\xi_{t}-m\left(\xi_{t}^{*}\right)\right)+\left(\epsilon_{t}-m\left(\epsilon_{t}^{*}\right)\right)\\ & \approx \xi_{t} \end{array} \end{array}$$ (8)

since $l_{t}-m\left(l_{t}^{*}\right)\approx 0$, $\xi_{t}-m\left(\xi_{t}^{*}\right)\approx \xi_{t}$ and $\epsilon_{t}-m\left(\epsilon_{t}^{*}\right)\approx 0$. $D_{t}^{x}$ is a robust and stable estimator to *ξ*_*t*_.

In summary, under the definition of time series decomposition,

$$\begin{array}{c} x_{t}=l_{t}+\xi_{t}+\epsilon_{t} \end{array}$$ (6)

where *x*_*t*_ is the original time series, *l*_*t*_ is the long-term trend with seasonality, *ξ*_*t*_ is the abnormal signal driven by an event and *ϵ*_*t*_ is the independently and normally distributed random error with mean 0 and small variance *σ*^2^. If $m\left(x_{t}^{*}\right)\approx l_{t}$(under certain distribution assumption on $x_{t}^{*}$, *m*(⋅) is the optimal estimator for *l*_*t*_),

$$\begin{array}{c} D_{t}^{x}=x_{t}-m\left(x_{t}^{*}\right)\approx \xi_{t} \end{array}$$ (9)

So far, the idea of defining the time series smoothers has been elaborated. These smoothers can estimate and remove the long-term trend of multiple time series efficiently, regardless of the window or gap size. In the following, three classes of smoothers are proposed and each has its own merits. The first class is moving trimmed mean, where the trimmed mean is used within each window. It can change its local robustness, controlled by the trimming percentage, up to a researcher’s subject knowledge or preference. The second class is moving weighted mean, where the weighting scheme can also be determined by a researcher’s subject knowledge or preference. The third class is moving recursive weighted mean. It can re-weight the points within each moving window recursively in order to greedily detect abnormal signals. The re-weighting function is non-increasing and specified by a researcher, based on one’s subject knowledge or preference.

### Moving trimmed mean

A trimmed mean is a statistical measure of central tendency which equals to the mean after discarding given parts of a sample at the upper and lower tail of the observed distribution, and typically discarding an equal amount of both. A trimmed mean with nonzero trimming percentage is less sensitive to outliers and can give a reasonable estimate of central tendency. In this regard, it is referred to as a robust estimator.

The trimming percentage, ranging from 0% to 50%, allows us to obtain a collection of estimators with different robustness. When the trimming percentage is 0%, no points are trimmed and the estimator is the arithmetic mean; when the trimming percentage is 50%, 50% of high and low points are trimmed and this results in the median.

Moving average is defined as the average of several days’ observations with a centered gap. Here, we assume that the window size is 2*k* + 1 and the gap size is 2*l* + 1.

$$\begin{array}{c} MA\left(x_{t}^{*}\right)=\frac{1}{2(k-l)}\left(x_{t-k}+x_{t-k+1}+\cdots +x_{t-l-1}+x_{t+l+1}+\cdots +x_{t+k-1}+x_{t+k}\right) \end{array}$$ (10)

Moving median is defined as the median of several days’ observations with a centered gap.

$$\begin{array}{c} MM\left(x_{t}^{*}\right)=\frac{1}{2}\left(x_{\left(k-l\right)}+x_{\left(k-l+1\right)}\right) \end{array}$$ (11)

where *x*_(*k*−*l*)_ and *x*_(*k*−*l*+1)_ are the (*k* − *l*)th and (*k* − *l* + 1)th ordered statistics of the set {*x*_*t*−*k*_, *x*_*t*−*k*+1_, ⋯, *x*_*t*−*l*−1_, *x*_*t*+*l*+1_, ⋯, *x*_*t*+*k*−1_, *x*_*t*+*k*_}; *t* is the time of our estimation; *k* is the number of observations we use before and after the estimation time point (*k* ≥ 1) hence the total number of observations is 2*k* + 1 before gapping; *l* is the number of observations we take away from the center before and after the estimation time point (*l* ≥ 0) excluding *x*_*t*_. Thus, the total number of observations taken away is 2*l* + 1 and the total number of observations left is 2(*k* − *l*).

### Moving weighted mean

The weighted mean assigns different weights to the data points within each window in order to form an informed estimator. In general, it is up to a researcher to determine how to design the weighting scheme. Two typical types of moving weighted mean are presented here, center-weighted mean and edge-weighted mean. Center-weighted mean puts more weight on the center outside the gap than on the edges symmetrically. It weighs more on the local fluctuation near the time point of interest. Edge-weighted mean puts more weight on the edges than on the center symmetrically. It weighs less on the local fluctuation but more on the long-term trend. Moving weighted mean is defined as below.

$$\begin{array}{c} \begin{array}{cc} WM(x_{t}^{*})= & w_{t-k}x_{t-k}+w_{t-k+1}x_{t-k+1}+\cdots +w_{t-l-1}x_{t-l-1}+w_{t+l+1}x_{t+l+1}\\ & +\cdots +w_{t+k-1}x_{t+k-1}+w_{t+k}x_{t+k} \end{array} \end{array}$$ (12)

The sum of the normalized weight vector (*w*_*t*−*k*_, *w*_*t*−*k*+1_, ⋯, *w*_*t*−*l*−1_, *w*_*t*−*l*+1_, ⋯, *w*_*t*+*k*−1_, *w*_*t*+*k*_) is equal to 1. Dividing each element of the weight vector by its sum is called normalizing the weights.

Center-weighted moving average is defined as the center-weighted average of several days’ observations with a centered gap. The weight vector contains ∧-shaped linear weights and is normalized to 1, with more weight at the center and less weight at the edges symmetric to Time *t*.

Edge-weighted moving average is defined as the edge-weighted average of several days’ observations with a centered gap. The weight vector contains ∨-shaped linear weights and is normalized to 1, with less weight at the center and more weight at the edges symmetric to Time *t*.

### Moving recursive weighted mean

Recursiveness means that after the first set of deviations are obtained, more weight is assigned on small deviations and less weight is assigned on large deviations to form a new weighted mean and then we perform this procedure repeatedly until convergence. The idea is that those abnormal signals would stand out while those around the central tendency are almost zero. The detailed algorithm can be described as follows.

Step 1 Apply moving average, meaning equal weights, in the center-gapped window to obtain the estimated trend of time series.
Step 2 Calculate deviations from the estimated trend and standardize all the deviations.
Step 3 Apply a user-defined weight function on standardized deviations, for example, $e^{-x^{2}}$, normalize them and obtain moving weighted mean.
Step 4 Repeat Step 2 and 3 until the estimated trend converges with the difference of the estimated long-term trends ‖ ⋅ ‖_∞_ < 10^−6^.

The convergence of trend estimates is very fast. Typically, it takes less than 10 iterations for each window. The R code is available upon request.

There are infinitely many choices of the recursive weight function. In the following section, two recursive weight functions $e^{-{\left|x\right|}^{1/2}}$ and $e^{-x^{2}}$ are used for demonstration, where $e^{-{\left|x\right|}^{1/2}}$ has less penalized weighting on deviations while $e^{-x^{2}}$ has more penalized weighting on deviations.

### General properties

In general, choosing the width of window and gap should incorporate two basic considerations. First, if the period of seasonality is roughly known, then the window size should be a fraction of the period. The literature in the case crossover design, see [17], of air quality study typically use a window size from a few weeks up to a month. If certain shock events are roughly known, then the gap size should be approximately the shock width. Shocks are usually certain extreme phenomena that immediately raise the level of ozone, for example, wildfires.

For the three classes of time series smoothers, there are both shared and individual properties. The shared properties come from the window and gap. When the window size increases, the trend estimated is smoother and the deviation will contain more long-term signal; when the gap size increases, the trend is also smoother and the deviation will contain more long-term signal. The individual properties come from the different measures of central tendency. Moving trimmed mean has widespread use. When the trimming percentage increases, the estimated trend becomes more robust. Moving weighted mean is more flexible, depending on the weighting scheme. It can accommodate a researcher’s subject knowledge and preference. Moving recursive weighted mean estimates a relatively robust central tendency and makes the abnormal deviations more prominent and the ordinary deviations near zero. In this sense, it should be more helpful in recovering abnormal signals.

More specifically, the exemplified smoothers also have their own properties. Moving average is less robust than moving median but more efficient in the trend removal. Within the moving window, if the fluctuations around the trend are symmetric, moving average can easily recover the underlying trend of the time series. If the fluctuations around the trend are normally distributed, then moving average is statistically optimal. Moving median is the most robust moving measure that captures the overall trend of the time series. Within the moving window, no matter whether the fluctuations around the trend are symmetric or not, the median can estimate the central tendency with little influence by outliers. If the fluctuations are Laplace distributed, then moving median is statistically optimal.

Center-weighted moving average can be used when a researcher wants more weight on the observations near the time point of interest to obtain a better local or short-term fluctuation estimate. It weighs heavily on the local observations in forming a long-term estimate thus is more aggressive on the short-term signal removal. Edge-weighted moving average is used when a researcher wants more weight on the observations further away from the time point of interest to obtain a longer-term estimate. It weighs lightly on the local observations in forming a long-term estimate thus less aggressive on the short-term signal removal.

Moving recursive weighted mean with $e^{-{\left|x\right|}^{1/2}}$ places more weight on the ordinary observations and less weight on the abnormal observations. This weight function penalizes less on large deviations and the resulting smoothed estimate follows the fluctuated curve closer. Hence, the deviations would moderately stand out. Moving recursive weighted mean with $e^{-{\left|x\right|}^{2}}$ has a weight function with much heavier weight on the ordinary observations and much lighter weight on the abnormal observations. This weight function penalizes more on large deviations and the resulting smoothed estimate follows the long-term trend closer. Hence, the deviations would be more prominent.

## Case study

### Data and description

For decades, the air quality problem in California has been discussed, due to potential impacts on the large population. In Los Angeles California, there is much sunshine and low rainfall; these weather characteristics contribute to high levels of ozone, fine particles, and dust. Moreover, there is often low wind speed accompanied by high air pressure. In [15], a large dataset on human morality and air quality of Los Angeles was collected and investigated. This dataset contains mortality counts, ozone levels, PM_2.5_ levels, minimum temperature, maximum temperature and maximum relative humidity, daily from Year 2000 to 2012.

**Fig 1:**
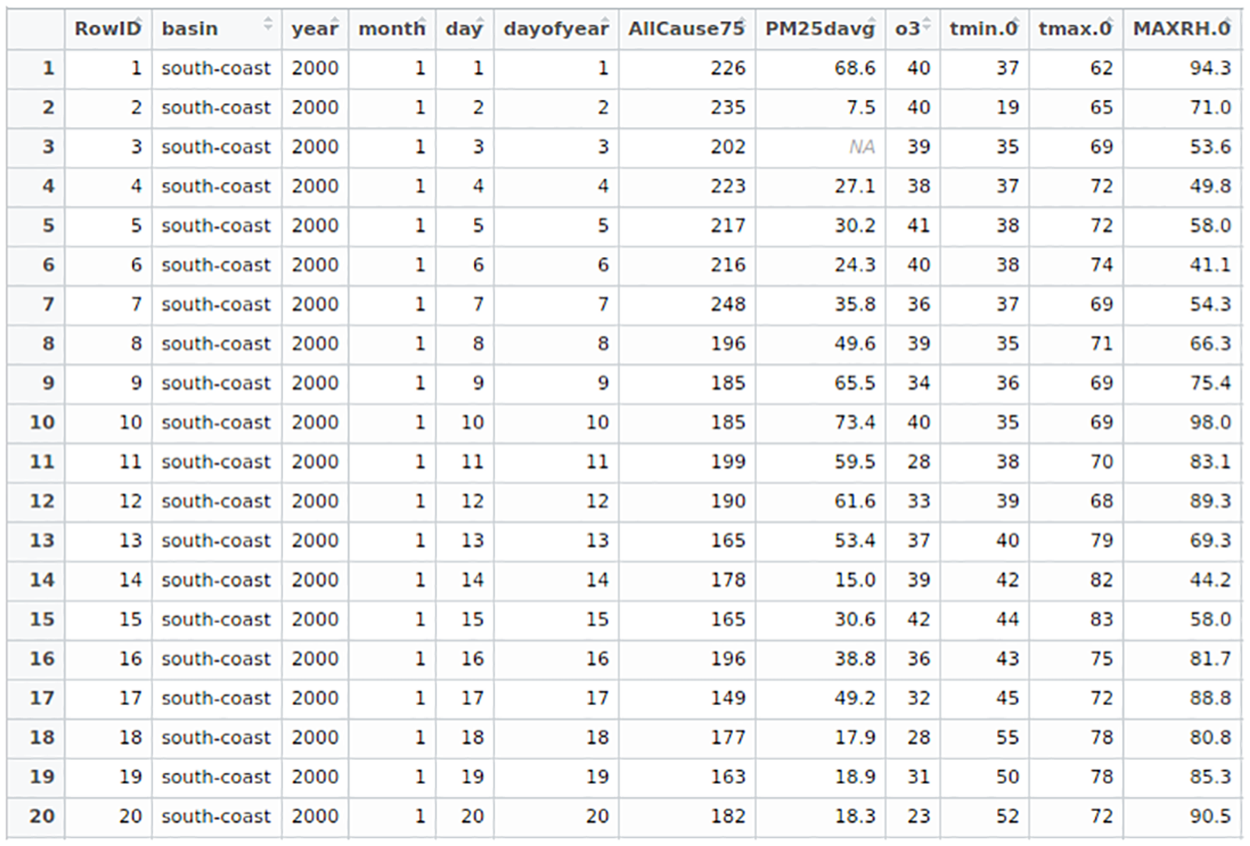
A screenshot of twenty data entries of Los Angeles. RowID: the number of data entry counting from Jan 1 2000 till Dec 31 2012. basin: the air basin south coast in Los Angeles California. year: the year of the data entry. month: the month of the data entry. day: the day of the data entry. dayofyear: the day of the year of the data entry. AllCause75: the daily number of mortality at Age 75 and above, excluding accidental deaths. PM25davg: the daily average level of PM_2.5_ in microgram per cubic meter (*μgm*^−3^). o3: the daily average level of ozone in parts per billion (*ppb*). tmin.0: the daily minimum temperatures are recorded in Fahrenheit (*°F*). tmax.0: the daily maximum temperatures are recorded in Fahrenheit (*°F*). MAXRH.0: the daily maximum relative humidity level in percentages of the air-water mixture.

Fig 1 is a snapshot of the research dataset, downloadable from S1 Dataset. *AllCause*75 is the daily mortality at Age 75 and above, excluding accidental deaths. *o*3 is the daily average level of ozone. *tmin*.0 is the daily minimum temperature. *tmax*.0 is the daily maximum temperature. *MAXRH*.0 is the daily maximum relative humidity level.

In this case study, the cross-association between daily ozone level and mortality is our main interest. Due to a large number of analysis choices, a factorial design of different scenarios is configured. By the factorial design and its corresponding sensitivity analysis, characteristics of the proposed smoothers are shown, along with the subject question about the acute effect.

For the dependence measure, the correlation and partial correlation, as well as their p-values, are adopted. Partial correlation adjusts for the observed weather covariates in this dataset: minimum temperature, maximum temperature, and maximum relative humidity level.

### Current smoothing methods

The current smoothing methods, such as spline smoothing, require the smoothing parameter tuning. Depending on how to choose the smoothing parameters, significant correlations can be induced, from negative to positive. For demonstration, cubic smoothing splines are utilized. Cubic smoothing splines perform a regularized regression over the natural spline basis, placing knots at all the points. They circumvent the problem of knot selection and simultaneously control for over-fitting by shrinking the coefficients of the estimated smooth function. More details can be seen in [18]. It can be shown that the spline smoothing methods can be too smooth to be relied on.

**Fig 2:**
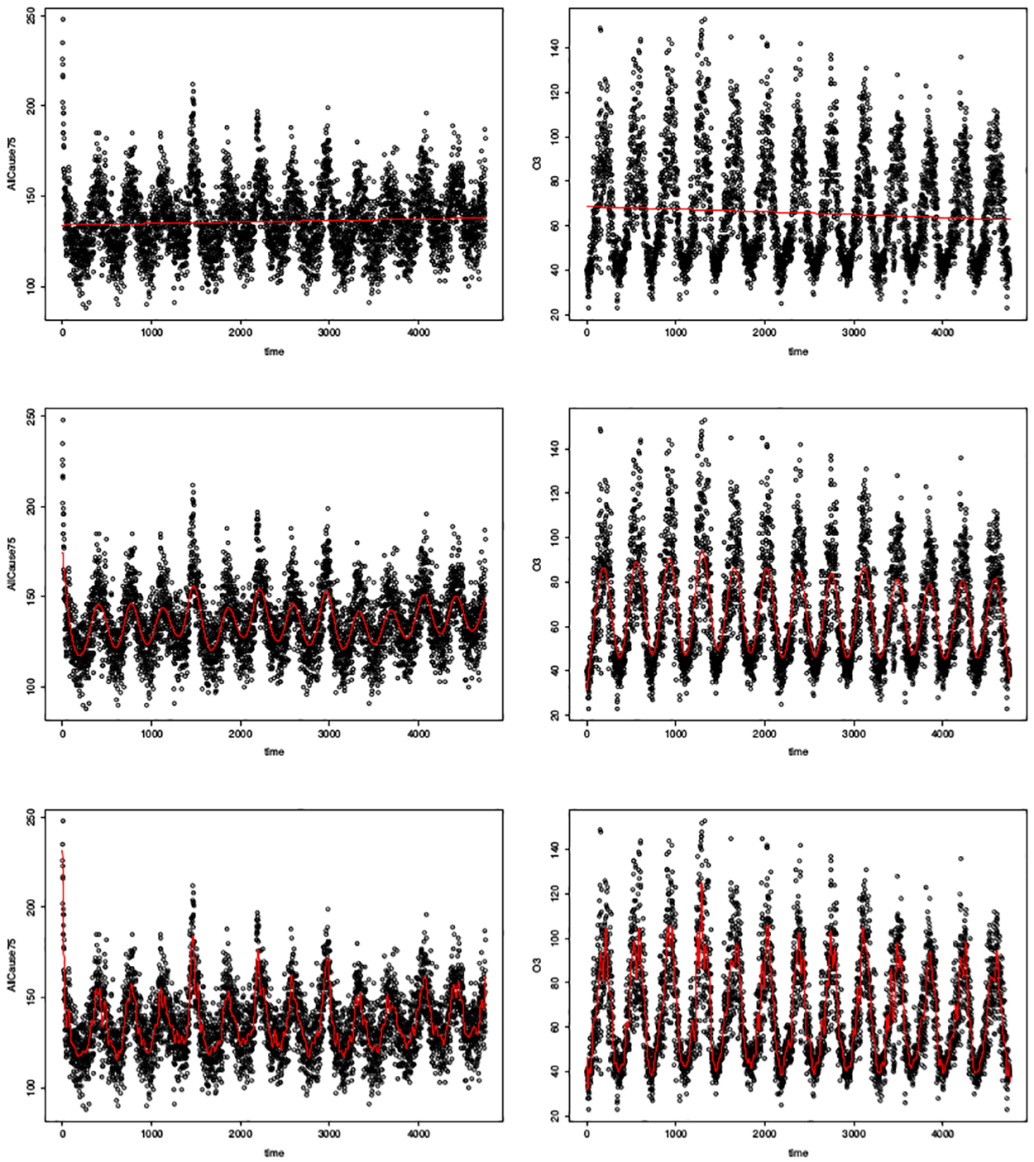
Raw time series and smoothing splines of mortality and ozone. The left three figures are the time series of mortality and the right three figures are the time series of ozone. The black dots represent the observed values and the red curves represent the smoothing splines. The smoothing parameters from top to bottom are set to be 1.5, 0.6, -1.5, representing highly smooth, moderately smooth and highly rough splines.

In Fig 2, the cubic smoothing spline changes from a straight line to the roughest curve by varying the smoothing parameter. The smoothing parameter is controlled by the option *spar* of *smooth*.*spline*() in R. The upper bound *spar* = 1.50 represents the smoothest while the lower bound *spar* = −1.50 represents the roughest. For simplicity, the smoothing parameters are set to be equal for both *AllCause*75 and *O*3.

In Fig 2, the red line in each plot is a cubic smoothing spline with a certain *spar*; *spar* changes from the highly smooth *spar* = 1.50, the moderately smooth *spar* = 0.60 to the highly rough *spar* = −1.50, from top to bottom. The deviations are obtained by using the observed values subtracting the spline values at each time.

In Table 1, Spar is the smoothing parameter *spar* in *smooth*.*spline*() and its values are chosen based on the sign changes of the resulting correlations. Correlation means Pearson correlation between deviations of mortality and deviations of ozone, while partial correlation means the partial correlation between deviations of mortality and deviations of ozone given the weather covariates temperature and relative humidity. As the long-term trend including seasonality gets eliminated more and more, both the correlation and partial correlations between mortality and ozone changes from significantly negative, insignificant and then to significantly positive. Hence, researchers have to find a proper way to justify their detrending method or find a method with better accuracy and precision before making a claim on any possible association between air quality and acute mortality.

**Table 1:** Correlations and partial correlations with p-values between mortality and ozone.

Spar	Correlation	P-value	Partial Correlation	P-value
1.50	-0.4313	≈ 0	-0.0835	≈ 0
0.71	-0.2665	≈ 0	-0.0010	0.9427
0.70	-0.2429	≈ 0	0.0081	0.5750
0.61	-0.0052	0.7203	0.0698	≈ 0
0.60	0.0140	0.3367	0.0717	≈ 0
-1.50	0.0725	≈ 0	0.0463	≈ 0

### Proposed smoothing methods

To study the proposed smoothers, a factorial design of different scenarios is utilized. By experimenting with all representative combinations of analysis choices, the factorial design can give us a thorough sensitivity analysis. Table 2 gives a summary of the factorial design of scenarios; detailed explanations follow.

**Table 2:** Factors and levels of factorial design of scenarios.

Factor	Level
Window	7, 15, 21, 29, 35, 43, 49 or 57
Gap	0, 1, 3, 5, 7, 9, 11 or 13
Measure	e.g. Trimming Percentage = 0% or 50%
Type	DD, DR, RD, RR

For the moving window size, the factor Window is defined as 7, 15, 21, 29, 35, 43, 49 or 57 days. The actual meanings are half a week, one week and up to one month before and after the time of interest. For the gap size, the factor Gap is defined as 0, 1, 3, 5, 7, 9, 11 or 13 days. The actual meanings are no removal, one-day point removal and up to one-week points removal before and after the time point of interest. Note that when the gap size is 0, the ordinary moving measures are simply used. The gap is always smaller than the window. For the measure, the factor Measure uses two distinctive levels on central tendency to study the effect of the pre-selected parameter or weighting scheme. For moving trimmed mean, the trimming percentages are selected to be 0% the average or 50% the median. There are infinite choices of the parameters or weighting schemes within the window. This can be decided by a researcher’s subject knowledge or preference.

The factor Type is defined, where D and R stands for deviations and raw data, respectively, to investigate whether there is any effect between deviations of mortality and deviations of ozone by the abbreviation DD, between deviations of mortality and raw time series of ozone by the abbreviation DR, between raw time series of mortality and deviations of ozone by the abbreviation RD and between raw time series of mortality on raw time series of ozone by the abbreviation RR. Since no smoother is applied on RR, it is a special case in this design.

All scenarios combined resembles a full factorial design of experiments for us to examine all possible effects. This factorial design is of the size 8 × 8 × 2 × 4 − # missing = 325.

For the factorial analysis, a linear regression model is utilized and all main effects and two-way interactions are chosen. Volcano plots are utilized to summarize the effect size and significance.

For illustration, the results of moving trimmed mean are demonstrated. Moving weighted mean and moving recursive weighted mean can be analyzed in a similar fashion. For the factor Measure, moving average with trimming percentage = 0% and moving median with trimming percentage = 50% are selected.

**Fig 3:**
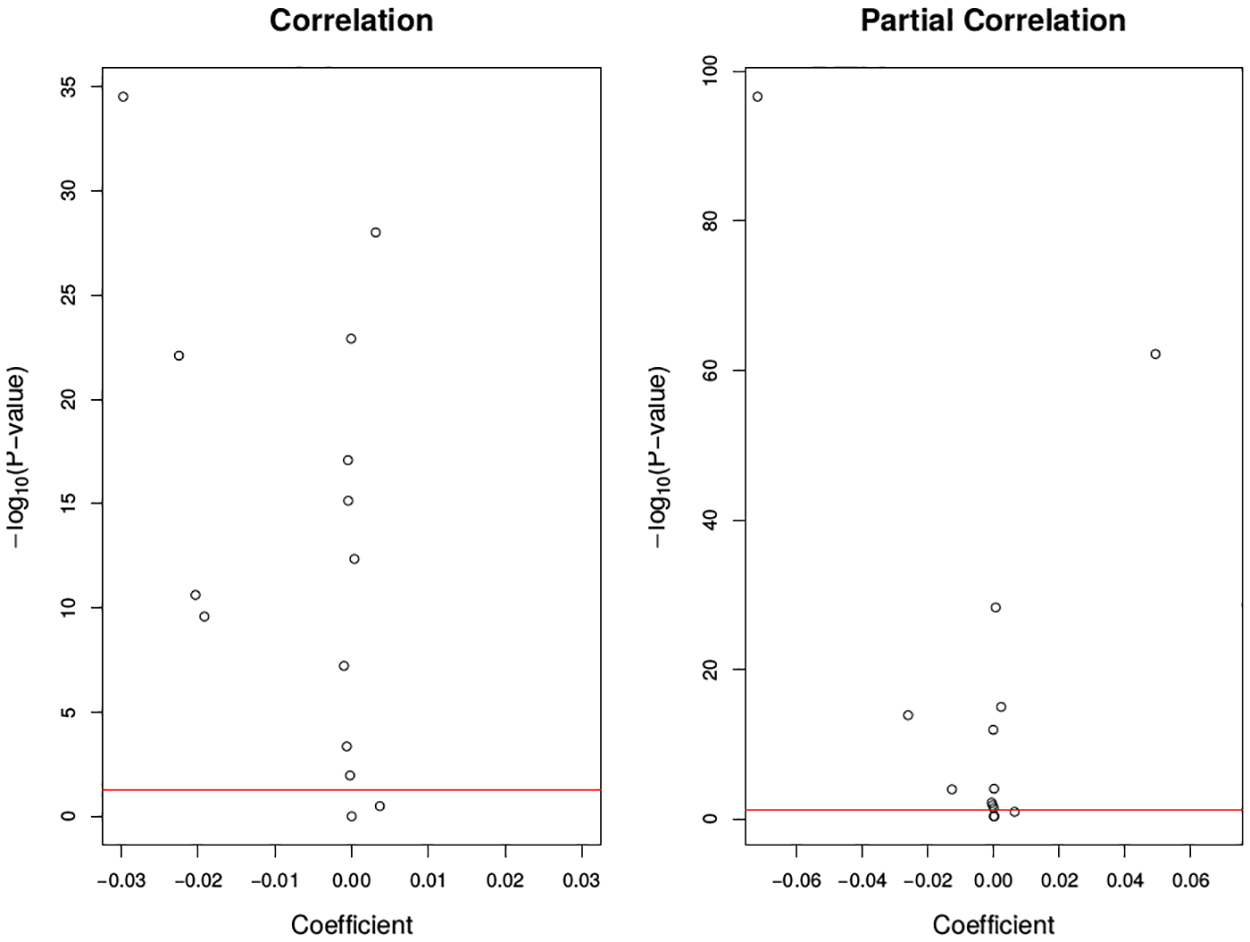
Volcano plot of regression coefficient and p-values by moving trimmed mean. The x-axis is the effect size measured by regression coefficient. The y-axis is the transformed p-value by -log_10_(); the larger transformed value, the more significance. The black dots represent each coefficient and the red lines are *y* = −log_10_0.05: any black dot above the red line means that the coefficient is nominally significant.

In Fig 3 and S1 Appendix, it is shown that only the effects with Type have both large size and significance; all other effects have small size although they are significant. For correlation, all main effects and two-way interaction effects are significant except the main effect Lambda and the interaction effect Gap×Lambda. However, the increments of Window or Gap are so small when Type is fixed that they would not alter the sign of the correlation. On the other hand, Type can make a significant difference on the cross-association between mortality and ozone. The trimming percentage Lambda is insignificant. For the response measure partial correlation, we can obtain similar results. The table of detailed coefficient estimates and significance are in S1 Appendix. Thus, moving trimmed mean is robust and stable with respect to different Window, Gap and Lambda for a fixed Type.

**Fig 4:**
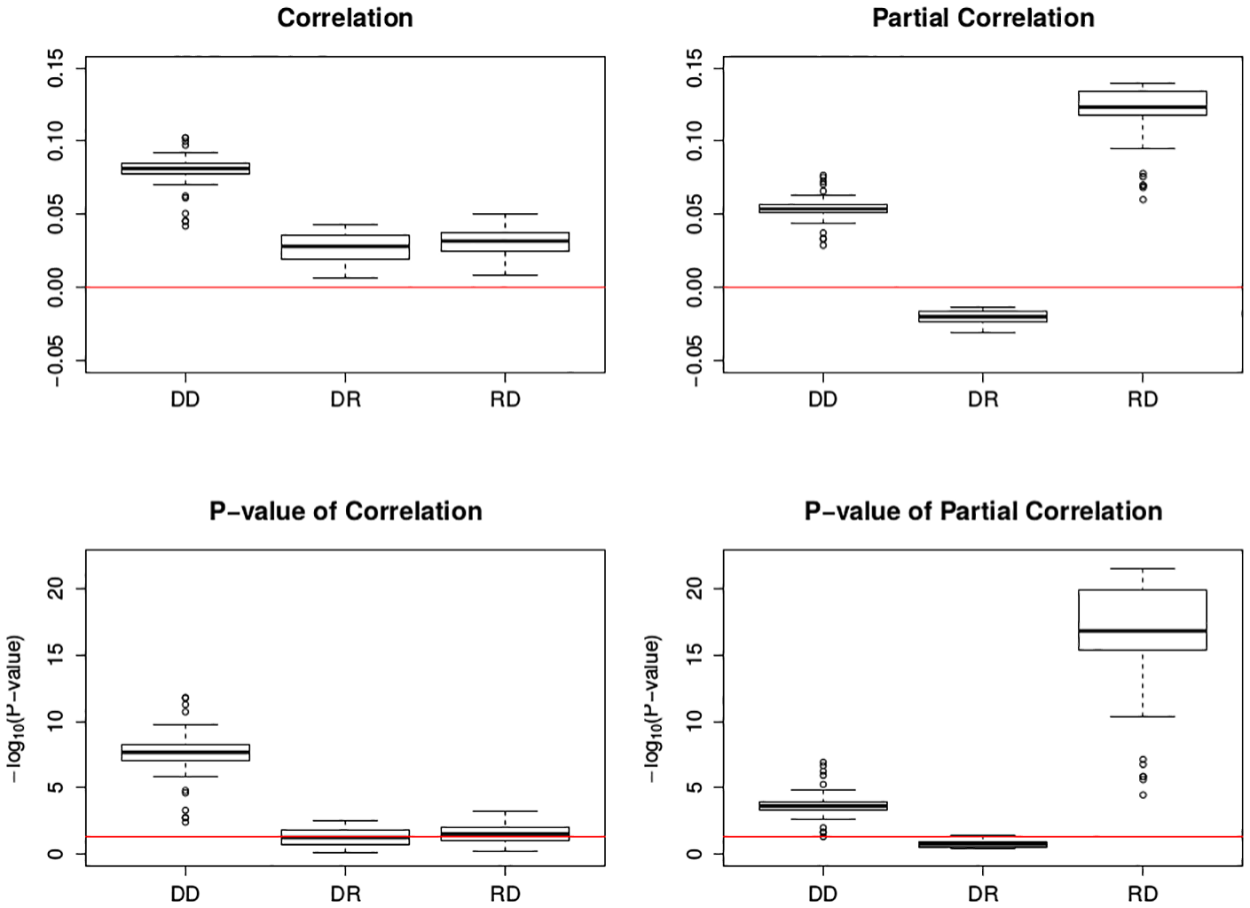
Boxplots of association between mortality and ozone by moving trimmed mean. The top two figures are the correlation and partial correlation between mortality and ozone; the bottom two figures are their corresponding transformed p-value plots by -log_10_(): the larger transformed value, the more significance. The x-axis has the three levels of the factor Type. The top two red lines are *y* = 0; the bottom two red lines are *y* = −log_10_0.05. Each box plot is the graphical summary under different Window, Gap and Lambda.

In Fig 4, correlation and partial correlation across different types are summarized in boxplots. Table 3 shows the correlations and partial correlations with their p-values for each type when Window = 21, Gap = 5 and Trimming Percentage = 50%.

**Table 3:** Correlations and partial correlations with p-values by type for moving trimmed mean: Window = 21, Gap = 5, Trimming Percentage = 50%.

Type	Correlation	P-value	Partial Correlation	P-value
DD	0.0869	≈ 0	0.0568	≈ 0
DR	0.0331	0.0227	-0.0283	0.0515
RD	0.0336	0.0207	0.1140	≈ 0
RR	-0.4338	≈ 0	-0.0956	≈ 0

Based on Fig 4 and Table 3, it can be seen that the correlations and partial correlations when Type = DD are positively significant. This means that the acute mortality has a nominally significant positive linear association with the sudden change in ozone, with or without meteorological covariates. When Type = DR, half of the correlations are insignificant while all the partial correlations are insignificant. This means that without meteorological covariates, the linear relationship between the acute mortality and ozone level is unclear; with meteorological covariates, there is no significant linear relationship between the acute mortality and ozone level. When Type = RD, half of the correlations are significant while all the partial correlations are positively significant. This means that without meteorological covariates, the linear relationship between the mortality level and ozone change is unclear; with meteorological covariates, there is no significant linear relationship between the mortality level and ozone change. Detrending on both time series appears to be a greedy search on positive significance. In addition, it is a researcher’s decision to examine the raw data or the deviations. Is it the jump in the air quality variable that has an acute effect on mortality or is it the absolute level? Depending on the researcher’s decision on Type, either positive significant effect or null effect can be deduced.

From the sensitivity analysis, Window and Gap can adjust the cross-association slightly; however, the sign of cross-association will not flip when Type is fixed. For moving trimmed mean, the small effect of Window and Gap implies the robustness to different window and gap sizes, and the null effect of Lambda implies the stability.

### Discussion & recommendations

In this case illustration, it is discovered that the current spline smoothing methods can induce significant correlations, both negative and positive, depending on how the smoothing parameters are varied, see Table 1. On the other hand, robust and stable smoothers are proposed to avoid the fine tuning of smoothing parameters; the sensitivity of the proposed smoothers is also evaluated on the sample dataset.

The framework of the proposed methods is to utilize a moving window with a centered gap. Within each window, different central tendency measures can be selected, based on a researcher’s preference. As the window size increases, the estimated curve is smoother and the detrending is less severe; a stronger association will be found between deviations. As the gap size increases, more local signals are removed from the window, the estimated curve is also smoother and the detrending is less severe; stronger association will be found between deviations. However, no matter how the window or gap size changes, within a wide range of days used by subject experts, the sign or significance of the cross-association for each type remains the same. This is the first important property, robustness to different window and gap sizes.

Within each moving window with a centered gap, three classes of time series smoothers are proposed. For each moving measure, varying the parameter or weight can adjust the correlation or partial correlation. However, the sign or significance does not change. This is the second important property, stability to different central tendency measures.

For moving trimmed mean, a collection of trimmed means is utilized, changing from mean to median. Within each moving window, different trimmed means can reach statistical optimality by certain goodness of fit criterion, depending on the underlying distributions. If a researcher does not have any subject knowledge or preference on the abnormal signals of time series data, moving average or median should work well.

For moving weighted mean, weighted arithmetic mean is utilized. Within each moving window, a researcher can decide how to assign different weights to the points based on one’s subject knowledge or preference on the abnormal signals of multiple time series data. If a researcher has insight on the nature of the data, moving weighted mean can be quite useful.

For moving recursive weighted mean, a certain re-weighting scheme is utilized in order to retain central tendency conservatively and enhance abnormal short-term signals. It is more greedy in searching for an anomaly. If a researcher emphasizes on conserving the long-term central tendency and revealing more anomalies, moving recursive weighted mean can be a good choice.

## Simulation study

### Simulation setting

For our setup, the target cross-correlations between deviations of mortality and deviations of ozone are selected to be 0, 0.01, 0.02, 0.05 and 0.10, because it is generally thought that the acute effect between air quality and mortality is relatively small. In Section Case Study, the detected correlation is between 0.05 and 0.10 and therefore it is reasonable to set the correlation to be under 0.10. Including 0.01 and 0.02 can demonstrate how sensitive the proposed smoothers are in discovering abnormal short-term effect. Including 0 can demonstrate whether the proposed smoothers cause large spurious correlation. When the cross-correlation between deviations of mortality and ozone is pre-specified, leaving the other covariate structures unchanged, the partial correlation will also be pre-specified and can be obtained from the whole correlation matrix. Using the R package *corpcor*, the corresponding partial correlations are computed as -0.0290, -0.0182, -0.0074, 0.0250 and 0.0791.

Since the proposed smoothers give consistent results, the choice of Window and Gap size can be flexible. In our case, Window = 21, meaning the extracted information are within 10 days before and after the time point of interest, and Gap = 5, meaning the removal of the local signal are within 2 days before and after, are chosen, based on the literature of air quality studies. The number of simulations for each scenario is 100 and the total number of simulations is 1000.

In this simulation study, the idea is to generate synthetic datasets very close to the original dataset, namely indistinguishable. In our case, indistinguishable means that the measures correlation and partial correlation are identical on both the original and synthetic datasets. To maintain the original correlation structure, each time series is decomposed by LOESS into the linear, seasonal and remainder terms. LOESS is locally weighted regression developed by [19] and the seasonal-trend decomposition is further developed in [20]. Only the deviations are simulated by multivariate normal distribution with the actual means and variances of the decomposed deviations, and pre-defined correlations thus covariances. The detailed algorithm is as follows.

Step 1 Decompose each time series into linear, seasonal and deviation components.
Step 2 Retain the actual means and variances of each time series of deviations. Set the correlation between deviations of mortality and ozone to be the pre-specified value above and obtain the new covariance matrix of all the variables.
Step 3 Simulate the deviations by multivariate normal distribution with the actual means and new covariance matrix.
Step 4 Add the simulated deviations back to the decomposed linear and seasonal components so that the simulated data are generated.

In the following, the boxplots of the differences between the found correlation (or partial correlation) and the specified correlation (or partial correlation) across different pre-specified values of correlation (or partial correlation) are displayed and interpreted. These boxplots are expected to be narrowly centered at 0 so that the estimation is accurate and precise. Additionally, the boxplots of correlation (or partial correlation) and −*log*_10_(p-value) across different pre-specified values of correlation (or partial correlation) are displayed and interpreted. The correlations (or partial correlations) are expected to be consistently larger or smaller than 0 to show stability and the −*log*_10_(p-value) plot should be above the threshold −*log*(0.05) to show significance.

### Simulation comparison

#### Current smoothing methods

Given the cubic spline smoothing, generalized cross-validation can be adopted to select the smoothing parameter.

**Fig 5:**
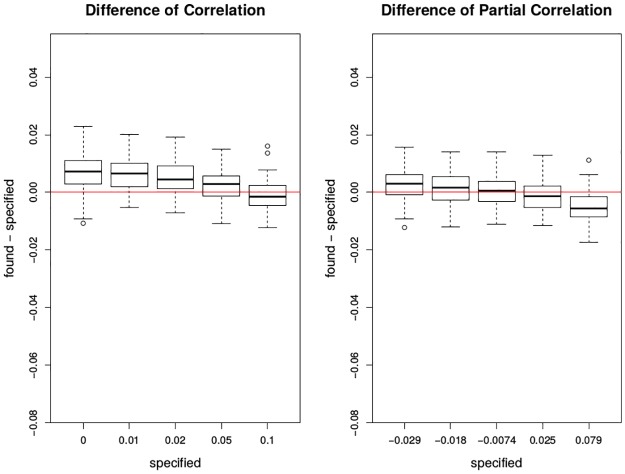
Plots of difference between found and specified in correlation and partial correlation by cubic spline smoothing under GCV. The x-axis has the five levels of specified correlations or partial correlations between the deviations of mortality and the deviations of ozone. The y-axis is the found—specified correlation or partial correlation after applying cubic spline smoothing under GCV. The red lines are *y* = 0. Each box plot is the graphical summary under simulations.

In Fig 5, it is seen that these boxplots are mostly around 0 and becomes closer to 0 when the pre-specified correlations value gets larger. Under multivariate normality, the current smoothing with generalized cross-validation (GCV) can identify the correct correlation and partial correlation with small errors. Generalized cross-validation is a common criterion for model selection, in this case, spline tuning parameter selection, which is a computationally efficient alternative to cross-validation, see [18].

**Fig 6:**
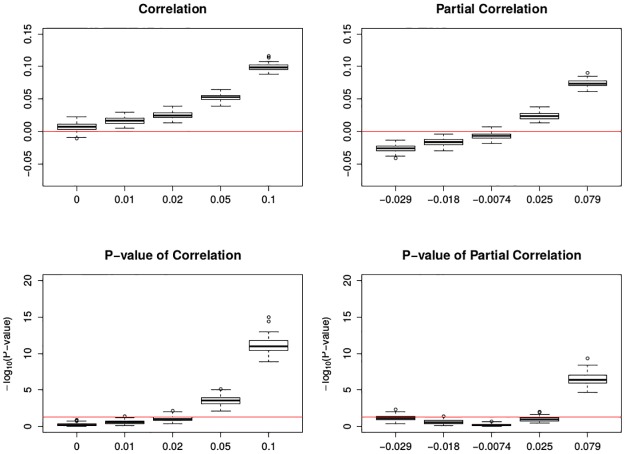
Boxplots of association between mortality deviations and ozone deviations by cubic spline smoothing under GCV. The top two figures are the correlation and partial correlation between mortality deviations and ozone deviations; the bottom two figures are their corresponding transformed p-value plots by -log_10_(): the larger transformed value, the more significance. The x-axis has the five levels of specified correlations and partial correlations. The top two red lines are *y* = 0; the bottom two red lines are *y* = −log_10_0.05. Each box plot is the graphical summary under simulations.

In Fig 6, both pre-specified correlation and partial correlation can be found consistently on the same side of 0; the significance can be detected when the pre-specified correlation is higher than 0.05.

Without the loss of generality, the highly rough *S*_*p*_ = −1.5 and the moderately smooth *S*_*p*_ = 0.60 are selected as the other smoothing scenarios.

**Fig 7:**
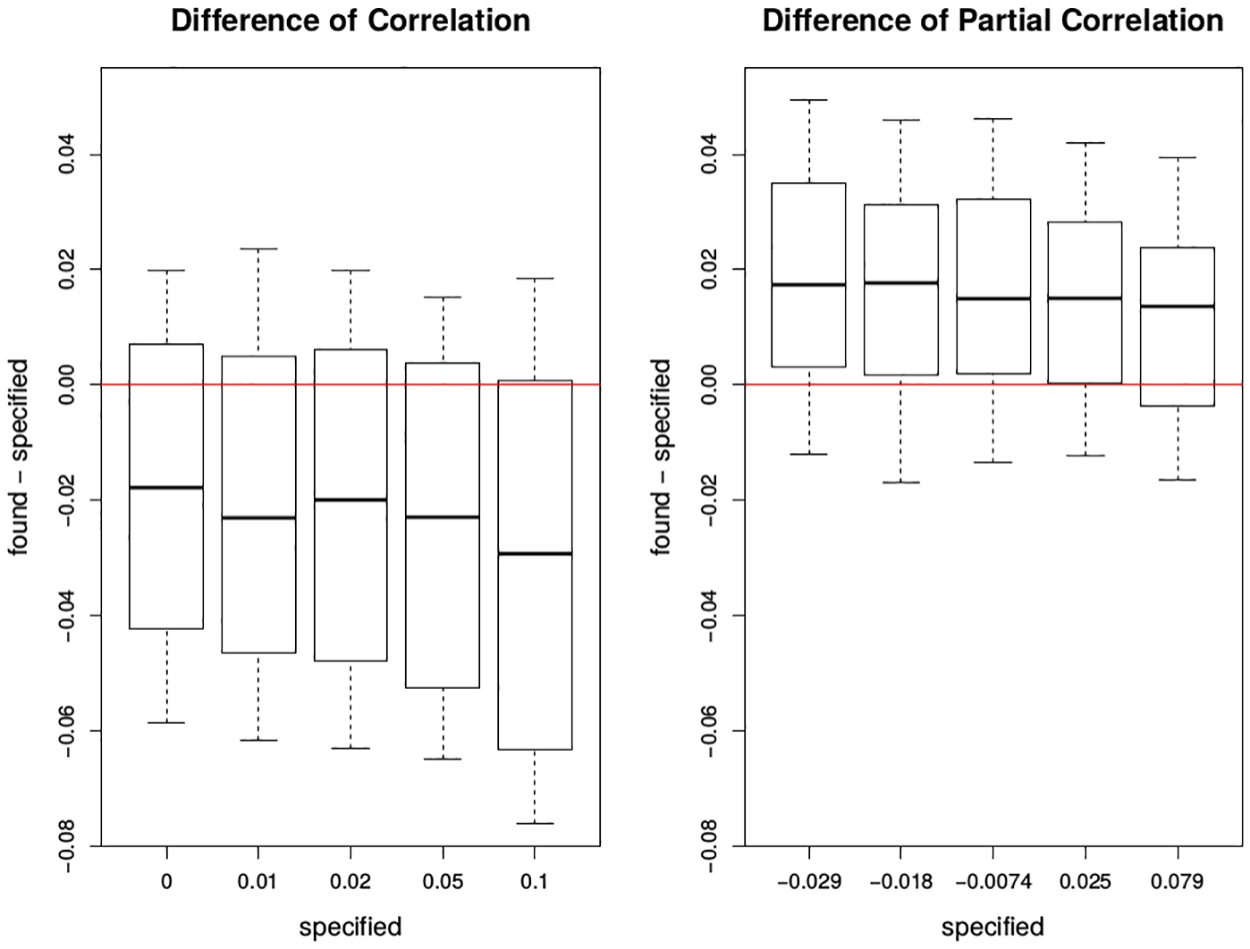
Plots of difference between found and specified in correlation and partial correlation by cubic spline smoothing. The x-axis has the five levels of specified correlations or partial correlations between the deviations of mortality and the deviations of ozone. The y-axis is the found—specified correlation or partial correlation after applying cubic spline smoothing under the two smoothing scenarios. The red lines are *y* = 0. Each box plot is the graphical summary under simulations.

**Fig 8:**
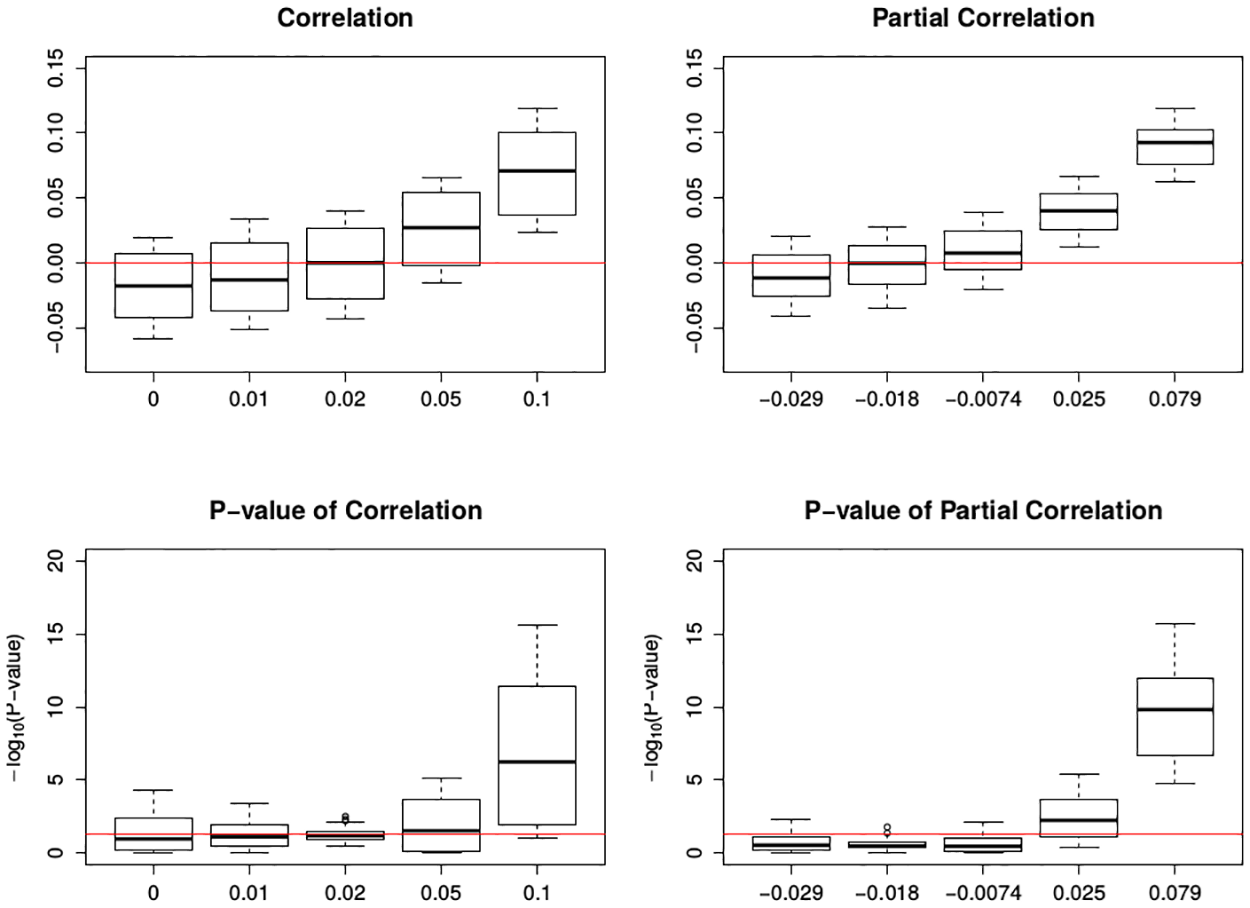
Boxplots of association between mortality deviations and ozone deviations by cubic spline smoothing. The top two figures are the correlation and partial correlation between mortality deviations and ozone deviations; the bottom two figures are their corresponding transformed p-value plots by -log_10_(): the larger transformed value, the more significance. The x-axis has the five levels of specified correlations and partial correlations. The top two red lines are *y* = 0; the bottom two red lines are *y* = −log_10_0.05. Each box plot is the graphical summary under simulations.

In Fig 7, it can be seen that the estimated correlations or partial correlations display some systematic bias, i.e., the estimated correlations are uniformly smaller while the estimated partial correlations are uniformly larger than the pre-defined values. Also, the estimation variation is large. In Fig 8, both correlation and partial correlation switch sides, from negative to positive. Statistical significance generally cannot be obtained except when the pre-defined correlation is 0.10, for either correlation or partial correlation.

#### Proposed smoothing methods

Following the above convention, moving trimmed mean is demonstrated. Moving average with trimming percentage = 0% and moving median with trimming percentage = 50% are used for simulation illustration.

**Fig 9:**
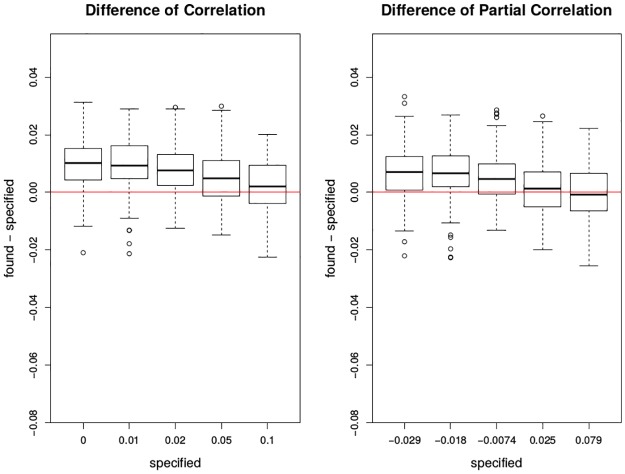
Plots of difference between found and specified in correlation and partial correlation by moving trimmed mean. The x-axis has the five levels of specified correlations or partial correlations between the deviations of mortality and the deviations of ozone. The y-axis is the found—specified correlation or partial correlation under the two trimming percentages. The red lines are *y* = 0. Each box plot is the graphical summary under simulations.

**Fig 10:**
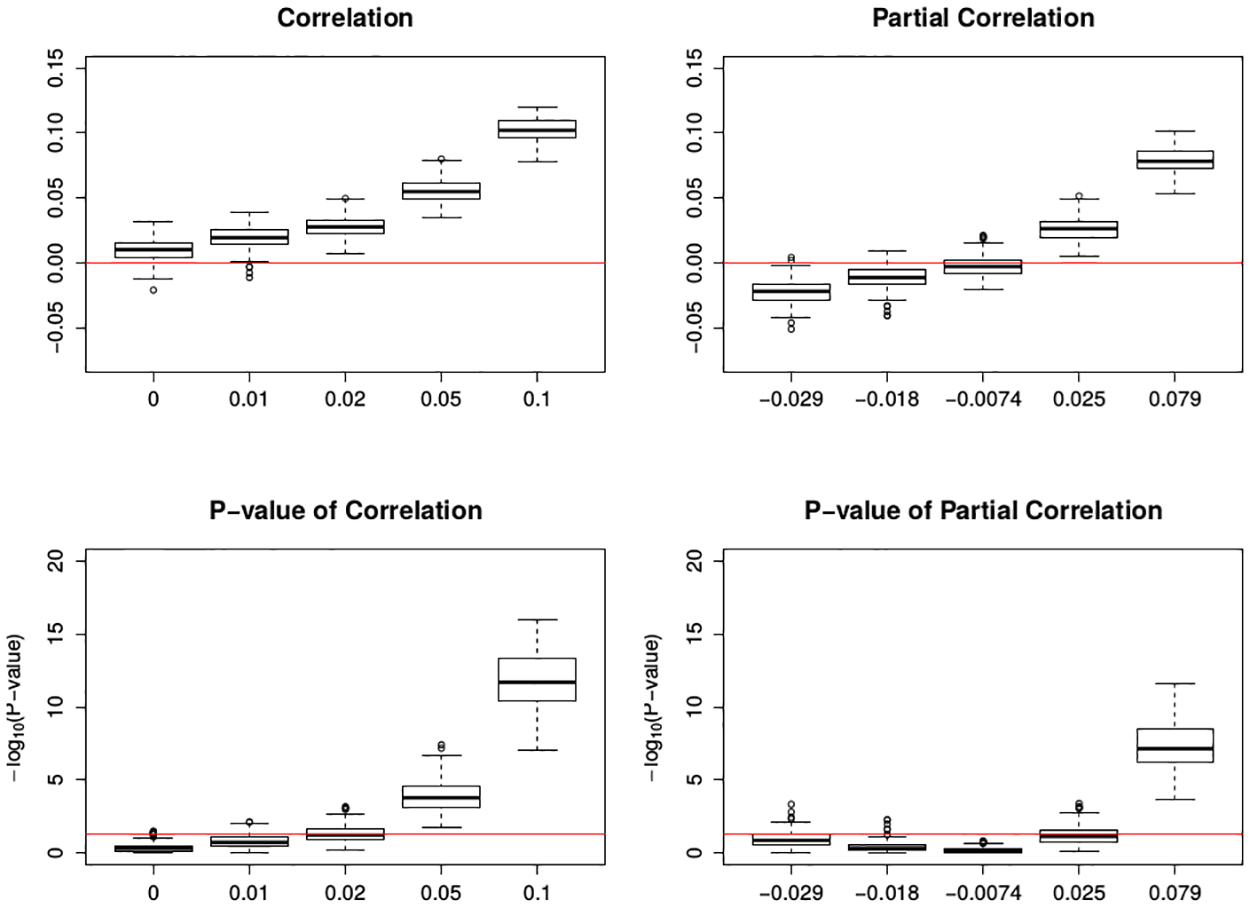
Boxplots of association between mortality deviations and ozone deviations by moving trimmed mean. The top two figures are the correlation and partial correlation between mortality deviations and ozone deviations; the bottom two figures are their corresponding transformed p-value plots by -log_10_(): the larger transformed value, the more significance. The x-axis has the five levels of specified correlations and partial correlations. The top two red lines are *y* = 0; the bottom two red lines are *y* = −log_10_0.05. Each box plot is the graphical summary under simulations.

In Fig 9, it can be seen that when the pre-specified correlation or partial correlation increases, the estimated correlation or partial correlation will be less biased. When the pre-specified correlation or partial correlation is relatively small, the found correlation or partial correlation tends to be slightly larger than the pre-specified ones. In Fig 10, when the pre-specified correlation is 0, no statistical significance can be found in either correlation or partial correlation. When the pre-specified correlation is small, such as 0.01 or 0.02, even if the correlations can be correctly found, we did not obtain statistical significance. When the pre-specified correlation gets larger than 0.05, our estimate becomes more accurate. Likewise, the partial correlation behaves similarly except that less significance can be found when the pre-specified cross-correlation is set small.

### Discussion & recommendations

Without a proper tuning parameter, the conventional approach of spline smoothing can change the association or even bias the estimation; it may also introduce large variation in estimation. Three proposed classes of time series smoothers can correctly detect small correlations and partial correlations, typically over 0.05, and the accuracy and precision of detection are improved when the actual association increases, given that the sample size is fixed.

If the long-term trend can be estimated more accurately, abnormal short-term signals are better recovered as well. One possible improvement is to adjust the window and gap size adaptively. For instance, we can enlarge the window and gap size during a non-volatile period and shrink their sizes during a volatile period. Another possible improvement is ideally to choose the most appropriate measure within each moving window with a centered gap. Assuming that the detrended time series are weakly stationary, there is a certain measure of central tendency that can achieve the optimality within the moving window. For instance, when the fluctuations around the long-term trend are normally distributed, moving average is statistically optimal for recovering the abnormal signals; when the fluctuations around the long-term trend are Laplace distributed, which places a higher probability on rare events than does the normal, moving median is statistically optimal. In more complicated distributions, different weighting schemes in moving weighted mean can empirically separate the long-term trend and abnormal signals well. Different recursive weighting schemes can also be useful in greedily searching for abnormal short-term signals. In our simulation setting, multivariate normal datasets are generated and all the measures behave very well under the normal assumption and thus the simulation results are quite similar.

The general recommendations can be stated as follows. First of all, the window and gap size should be chosen based on the scientific background or the literature. Ideally, they should be adaptive to capture the long-term trend. If no other information is available, a researcher can simply choose any pair of window and gap that fits the subject knowledge. Due to the robustness of the proposed smoothers, different window and gap sizes should always detect the cross-association reasonably well. Secondly, if a researcher has no subject knowledge or preference on the moving measure, moving trimmed mean should be used; the trimming percentage can be decided by the researcher, depending on how one wants to truncate for the central tendency within each window. If a researcher has some subject knowledge or preference on the moving measure, moving weighted mean should be used; the weighting scheme is up to the researcher, depending on the relevance of each data point to the time point of interest. If a researcher wants to focus on finding abnormal short-term signals, moving recursive weighted mean can be used; the re-weighting scheme is also up to the researcher’s choice, depending on how flexible a researcher wants for the long-term central tendency.

## Conclusions

First of all, the major formulations of smoothing in air quality studies are reviewed. A potential pitfall has been found: the prevailing spline smoothing methods can obtain any significant result, from negative to positive, by varying the smoothing parameters. This can undermine the validity of the discovered relationship between air quality and acute death. In general, the de-trending method is devised to estimate the deviation of the daily value from the trend by refitting the long-term trend value. As it turns out, the researchers in air quality studies have been quite versatile in how they fit the long-term trend, with little explanation or justification. That flexibility can lead to different answers, which is one of the novel results of our research.

Second, robust and stable time series smoothers are proposed. Each smoothed estimate can serve as an experimental control at the time point of interest. There are three classes of robust time series smoothers: moving trimmed mean, moving weighted mean and moving recursive mean. The strengths of the proposed methods are the robustness to different window/gap sizes and the stability to different tuning of the central tendency measure. One limitation is the inherent difficulty of knowing the correct separation between long-term trend and short-term fluctuation. Spline smoothing can obtain a wider range of smoothed curves, at the expense of moving the fluctuations into the trend or vice versa, while the proposed method results in less variation by different window and gap sizes. Another limitation is that the proposed method could be computationally heavy. The moving window with a centered gap needs to slide point by point to calculate the long-term trend estimate; it is time consuming to go through all the points from the beginning to the end. It would be more computationally expensive if we attempt to optimize the window and gap sizes. All of these moving window methods can generate robust and stable detrended time series.

Third, the characteristics and sensitivities of time series smoothers are examined using a factorial design of scenarios in our case illustration and indistinguishable synthetic datasets in our simulation. Choice of data type, raw data or deviations, has a dramatic effect on results. Within each data type, the proposed smoothers have the capacity of obtaining the correct cross-association with little bias and small variation.

Last but not least, although the robustness and stability of our proposed smoothers can assure the result’s consistency, it is still recommended to choose the window and gap size using domain knowledge, rather than experimenting with the dataset at issue. The window size should be a fraction of the period of the long-term trend with seasonality and the gap size should be the average length of the shock period. Within each moving window, a researcher can tailor-make the time series smoother, according to the data characteristics. A researcher should use moving trimmed mean if no extra information is available about the time series distribution. Otherwise, one should consider using moving weighted mean with an empirical weighting scheme that addresses the specific distribution. If one wants to estimate the central tendency conservatively and retain more short-term abnormalities, moving recursive weighted mean can be adopted. In a nutshell, the proposed time series smoothers provide us a reliable and efficient collection of statistical tools that do not require fine tuning of smoothing parameters and produce reliable results with little bias and small variation.

## Supporting information

S1 Dataset The whole dataset. This dataset contains the relevant air quality variables of Los Angeles California from Year 2000 to Year 2012.
(PDF)

S1 Appendix The linear model appendix. This appendix contains the summaries of the coefficient estimates, standard errors, t statistics and 2-sided p-values of the linear model with main effects and interactions for correlation and partial correlation.
(ZIP)
