# Supplementary material for: Time series smoother for effect detection
Source: PLoS One. 2018 Apr 23;13(4):e0195360. doi: 10.1371/journal.pone.0195360 (PMC5912770; doi:10.1371/journal.pone.0195360)
Supplement: S1 Dataset — This dataset contains the relevant air quality variables of Los Angeles California from Year 2000 to Year 2012. (PDF) [file pone.0195360.s001.pdf]

Dataset

RowID: the number of data entry counting from Jan 1 2000 till Dec 31 2012.
basin: the air basin south coast in Los Angeles California.
year: the year of the data entry.
month: the month of the data entry.
day: the day of the data entry.
dayofyear: the day of the year of the data entry.
AllCause75: the daily number of mortality at Age 75 and above, excluding accidental deaths.
PM25davg: the daily average level of PM2.5 in microgram per cubic meter.
o3: the daily average level of ozone in parts per billion.
tmin.0: the daily minimum temperatures are recorded in Fahrenheit.
tmax.0: the daily maximum temperatures are recorded in Fahrenheit.
MAXRH.0: the daily maximum relative humidity level in percentages of the air-water mixture.

RowID	basin	year	month	day	dayofyear	AllCause75	PM25davg	o3	tmin 0	tmax 0	MAXRH 0
1	south-coast	2000	1	1	1	226	68.6	40	37	62	94.3
2	south-coast	2000	1	2	2	235	7.5	40	19	65	71
3	south-coast	2000	1	3	3	202		39	35	69	53.6
4	south-coast	2000	1	4	4	223	27.1	38	37	72	49.8
5	south-coast	2000	1	5	5	217	30.2	41	38	72	58
6	south-coast	2000	1	6	6	216	24.3	40	38	74	41.1
7	south-coast	2000	1	7	7	248	35.8	36	37	69	54.3
8	south-coast	2000	1	8	8	196	49.6	39	35	71	66.3
9	south-coast	2000	1	9	9	185	65.5	34	36	69	75.4
10	south-coast	2000	1	10	10	185	73.4	40	35	69	98
11	south-coast	2000	1	11	11	199	59.5	28	38	70	83.1
12	south-coast	2000	1	12	12	190	61.6	33	39	68	89.3
13	south-coast	2000	1	13	13	165	53.4	37	40	79	69.3
14	south-coast	2000	1	14	14	178	15	39	42	82	44.2
15	south-coast	2000	1	15	15	165	30.6	42	44	83	58
16	south-coast	2000	1	16	16	196	38.8	36	43	75	81.7
17	south-coast	2000	1	17	17	149	49.2	32	45	72	88.8
18	south-coast	2000	1	18	18	177	17.9	28	55	78	80.8
19	south-coast	2000	1	19	19	163	18.9	31	50	78	85.3
20	south-coast	2000	1	20	20	182	18.3	23	52	72	90.5
21	south-coast	2000	1	21	21	160	20.3	31	52	65	90.1
22	south-coast	2000	1	22	22	154	36.2	34	46	65	86.5
23	south-coast	2000	1	23	23	149	57.9	33	43	68	89.2
24	south-coast	2000	1	24	24	163	40.3	30	44	76	82.3
25	south-coast	2000	1	25	25	154	14.7	28	49	71	97.3
26	south-coast	2000	1	26	26	161	10.3	39	47	69	82.2
27	south-coast	2000	1	27	27	132	26.1	41	39	69	80.6
28	south-coast	2000	1	28	28	148	46.9	42	39	71	75.4
29	south-coast	2000	1	29	29	150	46.2	45	39	71	81.8
30	south-coast	2000	1	30	30	150	40.9	43	47	64	91.5
31	south-coast	2000	1	31	31	137	16.5	48	49	65	93.2
32	south-coast	2000	2	1	32	126	24.9	41	43	78	63.6
33	south-coast	2000	2	2	33	127	12.9	44	44	82	61
34	south-coast	2000	2	3	34	152	19.5	51	42	80	41.3
35	south-coast	2000	2	4	35	155	17.9	44	45	75	74.9

36	south-coast	2000	2	5	36	140	29.1	55	45	70	84.9
37	south-coast	2000	2	6	37	124	46.8	51	43	79	74.1
38	south-coast	2000	2	7	38	153	40	55	44	85	71.5
39	south-coast	2000	2	8	39	138	34.1	36	50	80	84.8
40	south-coast	2000	2	9	40	147	39.3	37	46	75	85.1
41	south-coast	2000	2	10	41	123	17.5	39	47	67	98.7
42	south-coast	2000	2	11	42	117	9.8	40	41	65	89.7
43	south-coast	2000	2	12	43	133	7.1	43	49	64	90.9
44	south-coast	2000	2	13	44	120	11.5	40	42	64	100
45	south-coast	2000	2	14	45	135	12.7	35	42	68	99.5
46	south-coast	2000	2	15	46	150	27.2	43	48	68	88.6
47	south-coast	2000	2	16	47	118	19.1	42	49	64	99.5
48	south-coast	2000	2	17	48	119	9	42	43	63	88.8
49	south-coast	2000	2	18	49	140	31.6	45	36	72	76.6
50	south-coast	2000	2	19	50	132	6.7	48	42	81	61
51	south-coast	2000	2	20	51	158	4.5	45	43	75	87.3
52	south-coast	2000	2	21	52	132	19.5	44	43	61	98.8
53	south-coast	2000	2	22	53	145	15	42	42	66	89.6
54	south-coast	2000	2	23	54	135	9	41	41	66	99.9
55	south-coast	2000	2	24	55	136	14.6	40	37	61	80.1
56	south-coast	2000	2	25	56	139	14.6	39	42	60	88
57	south-coast	2000	2	26	57	134	36.4	49	40	71	85.9
58	south-coast	2000	2	27	58	148	42.3	46	41	69	98.2
59	south-coast	2000	2	28	59	136	11.4	44	40	63	85.7
60	south-coast	2000	2	29	60	140	22.3	42	44	65	80
61	south-coast	2000	3	1	61	129	24.8	40	42	67	84.1
62	south-coast	2000	3	2	62	143	31.1	57	40	72	73.2
63	south-coast	2000	3	3	63	145	16.8	54	47	68	89.8
64	south-coast	2000	3	4	64	151	14.7	50	45	59	97
65	south-coast	2000	3	5	65	126	6.6	52	37	57	96.8
66	south-coast	2000	3	6	66	147	8.9	54	36	61	84.9
67	south-coast	2000	3	7	67	127	17.2	51	40	61	89.6
68	south-coast	2000	3	8	68	158	7.1	51	40	60	96.2
69	south-coast	2000	3	9	69	141	11.3	50	34	65	83
70	south-coast	2000	3	10	70	138	25.7	54	40	75	66.1
71	south-coast	2000	3	11	71	166	18.4	56	43	84	60
72	south-coast	2000	3	12	72	132	30.4	67	45	81	76.3
73	south-coast	2000	3	13	73	164	44.6	64	44	78	82.9
74	south-coast	2000	3	14	74	140	53.4	86	46	83	74.1
75	south-coast	2000	3	15	75	134		84	50	81	82
76	south-coast	2000	3	16	76	160	70.3	80	53	78	84.8
77	south-coast	2000	3	17	77	144	45.6	59	51	83	75.7
78	south-coast	2000	3	18	78	127	36.2	66	48	87	70.1
79	south-coast	2000	3	19	79	132	39.2	91	46	85	78.3
80	south-coast	2000	3	20	80	144	11.5	62	47	72	45.1
81	south-coast	2000	3	21	81	124	10.3	60	49	75	28.5
82	south-coast	2000	3	22	82	129	29.3	57	42	79	65.4
83	south-coast	2000	3	23	83	157	16	63	42	74	73.1
84	south-coast	2000	3	24	84	126	21.7	67	42	75	81.2

85	south-coast	2000	3	25	85	106	39.2	63	44	73	81.5
86	south-coast	2000	3	26	86	131	18.3	77	44	80	75.6
87	south-coast	2000	3	27	87	149	35.1	66	44	70	85.2
88	south-coast	2000	3	28	88	138	18.6	49	49	66	91.3
89	south-coast	2000	3	29	89	117	20.1	52	48	69	89.3
90	south-coast	2000	3	30	90	140	42.1	55	48	72	81.4
91	south-coast	2000	3	31	91	139	16.7	62	45	82	39
92	south-coast	2000	4	1	92	148	12.1	72	48	87	55
93	south-coast	2000	4	2	93	123	16.9	73	46	88	67.6
94	south-coast	2000	4	3	94	127	30.3	77	47	83	73.8
95	south-coast	2000	4	4	95	137	40	68	50	87	72.8
96	south-coast	2000	4	5	96	131	34.4	66	48	82	82
97	south-coast	2000	4	6	97	124	49.5	59	49	83	90.2
98	south-coast	2000	4	7	98	121	89.2	74	51	78	84.1
99	south-coast	2000	4	8	99	114	59.6	78	50	87	68.9
100	south-coast	2000	4	9	100	120	21.2	66	46	85	77.4
101	south-coast	2000	4	10	101	125	27.4	55	46	81	77.9
102	south-coast	2000	4	11	102	121	64.4	61	48	89	74.8
103	south-coast	2000	4	12	103	118	36.9	75	50	90	67.7
104	south-coast	2000	4	13	104	142	36.9	62	51	85	78.6
105	south-coast	2000	4	14	105	125	8.8	42	51	78	87.4
106	south-coast	2000	4	15	106	125	13.5	46	49	72	80.9
107	south-coast	2000	4	16	107	118	11.6	53	47	73	81
108	south-coast	2000	4	17	108	129	6.7	45	48	68	90.6
109	south-coast	2000	4	18	109	114	6.6	57	46	65	97.4
110	south-coast	2000	4	19	110	131	11.8	55	45	73	79.8
111	south-coast	2000	4	20	111	137	31.4	68	46	84	67.9
112	south-coast	2000	4	21	112	146	31.5	56	50	78	89.5
113	south-coast	2000	4	22	113	129	16.6	58	41	69	94.8
114	south-coast	2000	4	23	114	128	29.1	69	51	72	91
115	south-coast	2000	4	24	115	107	47.6	69	49	88	76.2
116	south-coast	2000	4	25	116	115	21.9	88	51	88	64.8
117	south-coast	2000	4	26	117	124	30.9	100	51	95	69.5
118	south-coast	2000	4	27	118	128	61.2	90	51	93	85.1
119	south-coast	2000	4	28	119	114	38.6	81	56	78	89.6
120	south-coast	2000	4	29	120	115	19.3	67	49	83	61.2
121	south-coast	2000	4	30	121	126	18.9	82	49	91	66.5
122	south-coast	2000	5	1	122	143	28.9	85	39	91	64.8
123	south-coast	2000	5	2	123	119	32.3	97	52	88	70.1
124	south-coast	2000	5	3	124	131	38.8	97	55	88	81.6
125	south-coast	2000	5	4	125	125	57.2	106	55	85	86
126	south-coast	2000	5	5	126	118	67.4	86	58	78	87.4
127	south-coast	2000	5	6	127	109	43	90	57	80	80
128	south-coast	2000	5	7	128	128	29.1	83	55	80	81.3
129	south-coast	2000	5	8	129	113	22.2	41	54	77	89
130	south-coast	2000	5	9	130	104	48.2	84	56	84	80.9
131	south-coast	2000	5	10	131	118	26.9	63	56	77	86.2
132	south-coast	2000	5	11	132	124	12.8	62	49	78	64.5
133	south-coast	2000	5	12	133	132	15.8	61	45	86	44.7

134	south-coast	2000	5	13	134	123	14	86	46	87	63.9
135	south-coast	2000	5	14	135	131	23	84	46	89	71.4
136	south-coast	2000	5	15	136	122	12.4	70	45	79	78.2
137	south-coast	2000	5	16	137	117	6	54	50	78	76.5
138	south-coast	2000	5	17	138	126	18.1	59	47	77	75
139	south-coast	2000	5	18	139	130	21.9	69	49	92	72.4
140	south-coast	2000	5	19	140	145	33.2	78	52	94	69.8
141	south-coast	2000	5	20	141	131	36.6	110	50	98	71.7
142	south-coast	2000	5	21	142	121	40.1	117	51	103	78.3
143	south-coast	2000	5	22	143	130	51.3	88	53	95	92.1
144	south-coast	2000	5	23	144	136	45.2	91	55	83	92.5
145	south-coast	2000	5	24	145	106	26.8	73	55	73	96.7
146	south-coast	2000	5	25	146	139	22.4	45	54	66	100
147	south-coast	2000	5	26	147	104	31	69	56	83	99.2
148	south-coast	2000	5	27	148	116	42.8	124	56	102	72.9
149	south-coast	2000	5	28	149	112	28.9	149	55	100	70.9
150	south-coast	2000	5	29	150	117	37.3	111	56	93	86
151	south-coast	2000	5	30	151	103	48.3	95	57	91	84.5
152	south-coast	2000	5	31	152	120	36.8	89	59	86	81.3
153	south-coast	2000	6	1	153	142	24.3	93	54	92	81.9
154	south-coast	2000	6	2	154	114	35.6	90	55	91	78.4
155	south-coast	2000	6	3	155	118	38.4	106	53	93	72.4
156	south-coast	2000	6	4	156	101	30	148	52	93	69.2
157	south-coast	2000	6	5	157	120	20.6	56	53	93	73.8
158	south-coast	2000	6	6	158	116	18.5	71	54	93	73.7
159	south-coast	2000	6	7	159	109	22.3	73	54	88	71.5
160	south-coast	2000	6	8	160	105	9.2	39	54	82	79.1
161	south-coast	2000	6	9	161	130	14.2	58	51	80	73.7
162	south-coast	2000	6	10	162	130	20.3	91	52	86	76.9
163	south-coast	2000	6	11	163	117	29	101	53	89	79.4
164	south-coast	2000	6	12	164	116	20.5	104	54	93	71.1
165	south-coast	2000	6	13	165	116	29.6	112	56	97	81
166	south-coast	2000	6	14	166	129	24.3	72	58	104	86.4
167	south-coast	2000	6	15	167	132	20.9	122	56	96	80.9
168	south-coast	2000	6	16	168	116	30.4	90	52	87	82.4
169	south-coast	2000	6	17	169	127	32.1	103	61	89	86.4
170	south-coast	2000	6	18	170	105	31.2	89	58	87	87.7
171	south-coast	2000	6	19	171	112	28.6	79	60	88	78.2
172	south-coast	2000	6	20	172	116	30.3	82	57	93	81.3
173	south-coast	2000	6	21	173	101	37.8	104	59	93	83.4
174	south-coast	2000	6	22	174	118	29.6	103	61	92	84.4
175	south-coast	2000	6	23	175	112	22.1	81	61	88	84.9
176	south-coast	2000	6	24	176	116	20.4	100	61	93	79.3
177	south-coast	2000	6	25	177	128	16.1	78	59	96	71.5
178	south-coast	2000	6	26	178	133	17.8	87	60	96	76.1
179	south-coast	2000	6	27	179	123	20.4	85	62	98	77.4
180	south-coast	2000	6	28	180	124	23.4	89	62	97	75.7
181	south-coast	2000	6	29	181	134	25.7	90	63	96	82.1
182	south-coast	2000	6	30	182	118	37.7	84	62	92	83.2

183	south-coast	2000	7	1	183	127	30.4	82	60	93	82.5
184	south-coast	2000	7	2	184	120	29.2	78	57	91	82.8
185	south-coast	2000	7	3	185	114	31	75	60	91	79.7
186	south-coast	2000	7	4	186	120	39.3	75	55	85	79.1
187	south-coast	2000	7	5	187	125	55.7	75	56	87	77.9
188	south-coast	2000	7	6	188	119	19.7	89	56	89	79.8
189	south-coast	2000	7	7	189	127	19.3	69	56	87	80.5
190	south-coast	2000	7	8	190	98	24	78	56	87	79
191	south-coast	2000	7	9	191	115	27.7	85	56	86	81.5
192	south-coast	2000	7	10	192	101	16	78	57	85	77
193	south-coast	2000	7	11	193	115	40.7	87	57	86	80.2
194	south-coast	2000	7	12	194	105	34.3	97	56	89	80.9
195	south-coast	2000	7	13	195	110	25.8	93	55	93	74.7
196	south-coast	2000	7	14	196	95	17.2	88	58	94	79.5
197	south-coast	2000	7	15	197	111	15.9	77	57	92	81.8
198	south-coast	2000	7	16	198	104	22.4	84	59	90	82.4
199	south-coast	2000	7	17	199	133	23.5	85	56	95	74.8
200	south-coast	2000	7	18	200	128	21.3	88	58	102	71.6
201	south-coast	2000	7	19	201	126	21	87	59	106	61.6
202	south-coast	2000	7	20	202	133	24.4	107	59	104	84.9
203	south-coast	2000	7	21	203	123	24.4	106	61	97	87.1
204	south-coast	2000	7	22	204	121	28.8	126	61	97	77.9
205	south-coast	2000	7	23	205	119	22.3	98	63	99	70.3
206	south-coast	2000	7	24	206	134	24.2	93	60	98	76.9
207	south-coast	2000	7	25	207	106	27.1	103	63	100	76.8
208	south-coast	2000	7	26	208	120	27.2	101	62	100	80.1
209	south-coast	2000	7	27	209	113	38.3	109	61	100	78
210	south-coast	2000	7	28	210	135	37	104	60	101	77.8
211	south-coast	2000	7	29	211	111	34	97	61	98	79.5
212	south-coast	2000	7	30	212	117	36.3	125	62	101	80.2
213	south-coast	2000	7	31	213	101	19.5	87	63	101	78.3
214	south-coast	2000	8	1	214	124	23	97	66	100	77.5
215	south-coast	2000	8	2	215	123	20.4	97	65	98	71.5
216	south-coast	2000	8	3	216	124	21.8	95	64	98	77.6
217	south-coast	2000	8	4	217	126	19.7	97	63	99	77.6
218	south-coast	2000	8	5	218	123	21.7	110	60	102	77.6
219	south-coast	2000	8	6	219	113	32.7	115	59	99	84.1
220	south-coast	2000	8	7	220	107	28.8	82	63	93	87.5
221	south-coast	2000	8	8	221	112	27.7	80	60	96	81.5
222	south-coast	2000	8	9	222	124	33.2	99	61	98	68.3
223	south-coast	2000	8	10	223	117	18	84	59	102	64.4
224	south-coast	2000	8	11	224	96	21.4	91	61	106	62.5
225	south-coast	2000	8	12	225	120	20.3	114	62	106	68.2
226	south-coast	2000	8	13	226	134	27.6	121	63	105	70.6
227	south-coast	2000	8	14	227	116	24.4	102	60	101	77.3
228	south-coast	2000	8	15	228	135	33.9	92	65	101	73.1
229	south-coast	2000	8	16	229	131	24.7	100	66	102	66.8
230	south-coast	2000	8	17	230	139	22.7	82	66	97	78.9
231	south-coast	2000	8	18	231	119	30.1	70	64	98	72.8

232	south-coast	2000	8	19	232	90	19.4	123	60	100	72.8
233	south-coast	2000	8	20	233	116	17.3	100	56	95	83.3
234	south-coast	2000	8	21	234	122	24	94	58	92	81.7
235	south-coast	2000	8	22	235	115	34	113	60	92	83
236	south-coast	2000	8	23	236	135	35.8	85	61	98	82.2
237	south-coast	2000	8	24	237	136	40.1	90	63	96	80.9
238	south-coast	2000	8	25	238	132	29.7	89	66	91	86
239	south-coast	2000	8	26	239	114	27.9	98	63	96	78.6
240	south-coast	2000	8	27	240	111	27.2	90	60	96	83.8
241	south-coast	2000	8	28	241	121	27.1	56	62	84	86.7
242	south-coast	2000	8	29	242	120	28.2	60	62	83	97.9
243	south-coast	2000	8	30	243	112	22.1	53	61	80	92.8
244	south-coast	2000	8	31	244	124	15.7	54	61	80	81.6
245	south-coast	2000	9	1	245	108	8.9	41	59	78	79
246	south-coast	2000	9	2	246	115	7.4	58	55	85	75.1
247	south-coast	2000	9	3	247	110	14.6	68	53	88	71.3
248	south-coast	2000	9	4	248	133	14.8	65	54	93	74
249	south-coast	2000	9	5	249	110	23.1	72	54	90	70.2
250	south-coast	2000	9	6	250	120	21.9	74	56	98	69.7
251	south-coast	2000	9	7	251	123	41.1	67	56	94	72.1
252	south-coast	2000	9	8	252	107	33.6	75	57	95	73
253	south-coast	2000	9	9	253	127	33.6	83	52	95	72
254	south-coast	2000	9	10	254	114	34.8	92	54	97	70.5
255	south-coast	2000	9	11	255	120	40.7	67	54	102	55
256	south-coast	2000	9	12	256	140	33.7	86	61	106	51.9
257	south-coast	2000	9	13	257	125	35.7	87	61	107	57
258	south-coast	2000	9	14	258	119	17.4	73	63	98	82.4
259	south-coast	2000	9	15	259	125	22.5	85	62	100	76.5
260	south-coast	2000	9	16	260	116	23	102	65	109	66.1
261	south-coast	2000	9	17	261	130	23.2	112	65	107	72.4
262	south-coast	2000	9	18	262	114	37.4	86	62	100	77
263	south-coast	2000	9	19	263	105	53.7	105	63	95	79.4
264	south-coast	2000	9	20	264	116	50.8	89	62	87	87.5
265	south-coast	2000	9	21	265	88	34.5	53	62	82	98
266	south-coast	2000	9	22	266	125	6.8	46	61	72	94.9
267	south-coast	2000	9	23	267	127	9.9	45	58	75	85.5
268	south-coast	2000	9	24	268	133	17.7	63	52	94	77
269	south-coast	2000	9	25	269	121	16.6	77	52	94	68.1
270	south-coast	2000	9	26	270	118	28.5	75	56	90	82.7
271	south-coast	2000	9	27	271	126	59.4	65	59	84	92.3
272	south-coast	2000	9	28	272	131	60.2	66	62	80	89.3
273	south-coast	2000	9	29	273	117	61.1	80	60	81	84.7
274	south-coast	2000	9	30	274	146	60.8	83	57	87	87.7
275	south-coast	2000	10	1	275	117	69.8	111	63	88	86.8
276	south-coast	2000	10	2	276	146	58.3	94	56	88	83.9
277	south-coast	2000	10	3	277	108	50.1	70	52	83	89.2
278	south-coast	2000	10	4	278	110	25.7	61	59	76	96.5
279	south-coast	2000	10	5	279	115	18.8	61	60	81	89
280	south-coast	2000	10	6	280	118	63.7	61	61	77	96.9

281	south-coast	2000	10	7	281	112	13.5	59	61	74	92.6
282	south-coast	2000	10	8	282	137	61.3	83	54	80	88.2
283	south-coast	2000	10	9	283	129	89.8	74	54	76	90.4
284	south-coast	2000	10	10	284	123	9.1	56	56	73	83.7
285	south-coast	2000	10	11	285	106	6.5	48	51	69	89.3
286	south-coast	2000	10	12	286	124	14.3	46	49	70	81.2
287	south-coast	2000	10	13	287	129	15.5	59	45	79	70.3
288	south-coast	2000	10	14	288	139	20.9	75	47	87	75.9
289	south-coast	2000	10	15	289	113	32.7	76	48	85	79.8
290	south-coast	2000	10	16	290	135	38	69	49	90	71.1
291	south-coast	2000	10	17	291	143	48.6	72	51	91	72.5
292	south-coast	2000	10	18	292	118	84.4	53	55	87	92.9
293	south-coast	2000	10	19	293	112	100.2	64	53	81	87.6
294	south-coast	2000	10	20	294	114	119.6	69	53	86	92.5
295	south-coast	2000	10	21	295	118	52	65	55	77	92.4
296	south-coast	2000	10	22	296	105	15.8	46	51	81	68.2
297	south-coast	2000	10	23	297	126	15.1	40	53	76	85
298	south-coast	2000	10	24	298	140	19.2	51	54	76	78.8
299	south-coast	2000	10	25	299	91	19.1	56	51	77	77.9
300	south-coast	2000	10	26	300	116	7.9	38	49	74	92.6
301	south-coast	2000	10	27	301	123	16.9	39	52	66	90
302	south-coast	2000	10	28	302	142	12.3	45	52	71	91.2
303	south-coast	2000	10	29	303	132	11.3	46	53	73	94.8
304	south-coast	2000	10	30	304	144	12.5	39	46	68	87.1
305	south-coast	2000	10	31	305	119	19.3	42	43	69	80.6
306	south-coast	2000	11	1	306	150	32.9	45	42	73	71.4
307	south-coast	2000	11	2	307	128	36.5	47	46	76	89.1
308	south-coast	2000	11	3	308	146	15.3	48	44	77	42
309	south-coast	2000	11	4	309	130	15.4	45	45	81	45
310	south-coast	2000	11	5	310	124	29	53	43	75	76.8
311	south-coast	2000	11	6	311	123	29.2	47	48	67	94.5
312	south-coast	2000	11	7	312	138	17.7	44	44	72	54.1
313	south-coast	2000	11	8	313	131	19.2	45	39	74	62
314	south-coast	2000	11	9	314	135	18.7	48	42	66	87.6
315	south-coast	2000	11	10	315	140	8.4	44	44	62	84.3
316	south-coast	2000	11	11	316	140	16.1	53	40	65	83.2
317	south-coast	2000	11	12	317	116	17.8	52	35	68	59.1
318	south-coast	2000	11	13	318	145	22.7	41	33	67	55.2
319	south-coast	2000	11	14	319	126	35.1	42	31	62	72.5
320	south-coast	2000	11	15	320	144	32.3	42	35	64	70.7
321	south-coast	2000	11	16	321	133	31.4	43	34	64	76.8
322	south-coast	2000	11	17	322	133	23.2	46	34	70	51
323	south-coast	2000	11	18	323	138	22	44	36	73	42.4
324	south-coast	2000	11	19	324	142	18.1	42	34	82	40.3
325	south-coast	2000	11	20	325	146	29.2	37	38	78	51
326	south-coast	2000	11	21	326	154	26.2	40	48	82	65.8
327	south-coast	2000	11	22	327	115	41.6	38	42	76	84.3
328	south-coast	2000	11	23	328	125	48.8	48	39	70	83.5
329	south-coast	2000	11	24	329	151	64.3	49	39	72	78

330	south-coast	2000	11	25	330	149	87.5	40	43	80	70.5
331	south-coast	2000	11	26	331	138	56.2	47	44	79	65.9
332	south-coast	2000	11	27	332	117	74.8	44	41	77	76
333	south-coast	2000	11	28	333	134	67	53	42	82	69.4
334	south-coast	2000	11	29	334	123	70.8	55	44	77	85.8
335	south-coast	2000	11	30	335	132	75.5	41	40	74	83.9
336	south-coast	2000	12	1	336	119	87.8	42	43	71	85.3
337	south-coast	2000	12	2	337	161	113.9	47	40	76	85.9
338	south-coast	2000	12	3	338	152	99	50	42	82	72.3
339	south-coast	2000	12	4	339	140	56	47	45	79	61.9
340	south-coast	2000	12	5	340	147	41.9	42	47	84	56
341	south-coast	2000	12	6	341	128	49.8	26	48	80	57.7
342	south-coast	2000	12	7	342	151	32.8	23	52	78	76.5
343	south-coast	2000	12	8	343	140	25.9	27	49	73	99.2
344	south-coast	2000	12	9	344	146	26.4	29	50	68	91.9
345	south-coast	2000	12	10	345	125	29.6	35	51	66	92.5
346	south-coast	2000	12	11	346	118	42.7	41	42	64	85.3
347	south-coast	2000	12	12	347	123	30.2	41	43	64	91.5
348	south-coast	2000	12	13	348	108	19.8	40	37	64	87.1
349	south-coast	2000	12	14	349	119	34.7	33	41	60	90.5
350	south-coast	2000	12	15	350	155	68.4	43	42	62	84.7
351	south-coast	2000	12	16	351	138	26.7	42	42	78	53.1
352	south-coast	2000	12	17	352	126	21.7	44	41	83	56
353	south-coast	2000	12	18	353	151	15.7	43	39	78	21.9
354	south-coast	2000	12	19	354	138	12.9	39	38	79	23
355	south-coast	2000	12	20	355	155	40.4	41	40	76	58
356	south-coast	2000	12	21	356	139	44.4	35	39	77	76
357	south-coast	2000	12	22	357	132		41	38	72	83
358	south-coast	2000	12	23	358	142	83	54	39	72	78.6
359	south-coast	2000	12	24	359	149	84.5	54	40	70	87.1
360	south-coast	2000	12	25	360	131	57.5	54	42	74	45.7
361	south-coast	2000	12	26	361	152	56	54	41	76	58
362	south-coast	2000	12	27	362	152	19.9	39	36	76	35.3
363	south-coast	2000	12	28	363	146	40.1	35	39	79	45.9
364	south-coast	2000	12	29	364	170	54.2	34	39	82	62.1
365	south-coast	2000	12	30	365	145	55.7	36	39	81	63.8
366	south-coast	2000	12	31	366	141	69.3	41	40	82	58.3
367	south-coast	2001	1	1	1	140	77.3	43	40	78	99
368	south-coast	2001	1	2	2	152	27.7	48	30	80	36.4
369	south-coast	2001	1	3	3	140	18.4	47	40	83	25.3
370	south-coast	2001	1	4	4	172	19.8	45	42	82	62
371	south-coast	2001	1	5	5	163	33.8	35	44	75	42.3
372	south-coast	2001	1	6	6	155	37.7	39	46	74	64.8
373	south-coast	2001	1	7	7	155	65.8	41	42	74	70.1
374	south-coast	2001	1	8	8	162	44.4	46	39	67	96.7
375	south-coast	2001	1	9	9	135	33.8	37	43	64	91.9
376	south-coast	2001	1	10	10	155	30.5	44	42	59	94.6
377	south-coast	2001	1	11	11	132	8	46	35	57	98.9
378	south-coast	2001	1	12	12	153	19.1	44	35	56	94.9

379	south-coast	2001	1	13	13	164	36.5	37	35	63	86.7
380	south-coast	2001	1	14	14	135	48.3	41	35	63	82
381	south-coast	2001	1	15	15	185	36.4	38	38	59	89.1
382	south-coast	2001	1	16	16	155	33.8	44	33	60	74.8
383	south-coast	2001	1	17	17	133	22.4	45	32	64	50.2
384	south-coast	2001	1	18	18	158	40.5	43	33	64	63.5
385	south-coast	2001	1	19	19	170	52.2	33	37	65	69
386	south-coast	2001	1	20	20	141	55	39	38	74	60.9
387	south-coast	2001	1	21	21	179	44.8	40	39	74	61
388	south-coast	2001	1	22	22	156	45.6	39	40	67	76.8
389	south-coast	2001	1	23	23	133	48.6	45	37	64	85.3
390	south-coast	2001	1	24	24	144	29.5	42	38	59	93.5
391	south-coast	2001	1	25	25	148	30.5	38	38	59	85.3
392	south-coast	2001	1	26	26	157	24.2	40	37	56	98.4
393	south-coast	2001	1	27	27	129	32.3	38	36	61	86.6
394	south-coast	2001	1	28	28	137	32.9	44	38	62	82.8
395	south-coast	2001	1	29	29	149	22.3	44	38	58	94.3
396	south-coast	2001	1	30	30	159	31	47	35	66	68
397	south-coast	2001	1	31	31	145	30.5	46	37	66	62
398	south-coast	2001	2	1	32	136	22.7	43	38	72	42.2
399	south-coast	2001	2	2	33	169	24.7	40	38	78	41.3
400	south-coast	2001	2	3	34	161	31.1	39	43	84	62
401	south-coast	2001	2	4	35	161	8	42	45	90	40.6
402	south-coast	2001	2	5	36	157	16	42	50	84	55
403	south-coast	2001	2	6	37	157	31.7	52	43	78	87.9
404	south-coast	2001	2	7	38	135	7.8	43	42	59	91.3
405	south-coast	2001	2	8	39	124	16.9	44	33	60	57.3
406	south-coast	2001	2	9	40	152	21.9	48	35	61	77.3
407	south-coast	2001	2	10	41	163	17.4	45	39	58	92.3
408	south-coast	2001	2	11	42	176	11.3	46	35	60	87
409	south-coast	2001	2	12	43	166	22.5	43	39	55	98.6
410	south-coast	2001	2	13	44	185	4.9	47	35	55	96.7
411	south-coast	2001	2	14	45	155	9.7	45	37	58	85.3
412	south-coast	2001	2	15	46	158	32.2	44	34	62	82.3
413	south-coast	2001	2	16	47	147	43.3	53	37	68	72.5
414	south-coast	2001	2	17	48	144	30.8	51	41	68	61.5
415	south-coast	2001	2	18	49	156	33.3	48	45	67	82.9
416	south-coast	2001	2	19	50	170	17.4	45	42	63	91.3
417	south-coast	2001	2	20	51	162	19.5	52	42	64	87.3
418	south-coast	2001	2	21	52	172	26	43	40	69	80.5
419	south-coast	2001	2	22	53	154	20.2	44	46	65	86
420	south-coast	2001	2	23	54	121	12.4	41	41	58	95.5
421	south-coast	2001	2	24	55	151	17.9	45	38	55	96.6
422	south-coast	2001	2	25	56	153	8.5	44	44	58	99.9
423	south-coast	2001	2	26	57	152	8.3	39	45	62	96.5
424	south-coast	2001	2	27	58	128	24.8	47	46	57	97
425	south-coast	2001	2	28	59	158	11.6	44	42	53	99.2
426	south-coast	2001	3	1	60	147	19.1	60	37	66	81.2
427	south-coast	2001	3	2	61	144	29.9	51	38	65	88.3

428	south-coast	2001	3	3	62	164	22.3	58	44	65	85.1
429	south-coast	2001	3	4	63	133	17.2	54	47	61	83.6
430	south-coast	2001	3	5	64	152	10.7	49	46	70	89.4
431	south-coast	2001	3	6	65	144	7.9	45	48	67	98.2
432	south-coast	2001	3	7	66	151	16.2	45	48	68	100
433	south-coast	2001	3	8	67	151	37.5	49	48	69	94.4
434	south-coast	2001	3	9	68	133	25.2	53	45	66	95.7
435	south-coast	2001	3	10	69	143	13.2	50	40	63	90.2
436	south-coast	2001	3	11	70	128	13.7	49	45	68	94.9
437	south-coast	2001	3	12	71	143	18.8	53	42	72	68.5
438	south-coast	2001	3	13	72	145	25.1	50	41	75	71.6
439	south-coast	2001	3	14	73	129	32.5	58	42	71	83.9
440	south-coast	2001	3	15	74	149	54.9	55	46	71	87.3
441	south-coast	2001	3	16	75	132	61	57	49	69	93.2
442	south-coast	2001	3	17	76	159	57.2	67	45	78	76.7
443	south-coast	2001	3	18	77	106	54	79	46	87	68
444	south-coast	2001	3	19	78	137	33.8	58	47	89	60.7
445	south-coast	2001	3	20	79	162	56.4	65	51	88	74.2
446	south-coast	2001	3	21	80	143	92.3	68	40	85	87.7
447	south-coast	2001	3	22	81	129	56.1	61	53	69	96.3
448	south-coast	2001	3	23	82	112	57.3	76	41	70	90.9
449	south-coast	2001	3	24	83	144	61.1	68	49	74	88.9
450	south-coast	2001	3	25	84	115	57	57	50	74	92.3
451	south-coast	2001	3	26	85	147	57	51	54	74	94.3
452	south-coast	2001	3	27	86	146	48.5	52	46	79	79.5
453	south-coast	2001	3	28	87	153	50.9	73	49	75	88.4
454	south-coast	2001	3	29	88	130	54.2	58	42	75	96
455	south-coast	2001	3	30	89	155	71.5	65	48	85	81.7
456	south-coast	2001	3	31	90	147	85	82	44	78	90
457	south-coast	2001	4	1	91	112	68.4	72	48	75	96.6
458	south-coast	2001	4	2	92	129	13.8	69	48	68	91.5
459	south-coast	2001	4	3	93	128	9.7	69	45	66	86.7
460	south-coast	2001	4	4	94	133	10.4	55	47	64	85.8
461	south-coast	2001	4	5	95	150	11.3	57	44	65	81.5
462	south-coast	2001	4	6	96	132	14.1	56	43	64	82.5
463	south-coast	2001	4	7	97	132	7.6	52	44	61	96.4
464	south-coast	2001	4	8	98	132	9.7	55	37	65	76.3
465	south-coast	2001	4	9	99	154	10.4	53	37	62	89
466	south-coast	2001	4	10	100	148	18.6	52	38	66	89.7
467	south-coast	2001	4	11	101	143	16	51	42	66	97.5
468	south-coast	2001	4	12	102	122	12	50	46	66	86
469	south-coast	2001	4	13	103	156	25.4	66	42	76	71
470	south-coast	2001	4	14	104	152	23.9	65	43	76	77.3
471	south-coast	2001	4	15	105	143	19.6	90	43	77	69.5
472	south-coast	2001	4	16	106	126	37.3	86	43	80	69.5
473	south-coast	2001	4	17	107	148	34.7	66	42	85	65.1
474	south-coast	2001	4	18	108	174	40.6	67	48	80	84.8
475	south-coast	2001	4	19	109	156	25.2	52	46	70	82.5
476	south-coast	2001	4	20	110	139	8.7	50	45	68	83.3

477	south-coast	2001	4	21	111	161	5.6	52	43	69	88.1
478	south-coast	2001	4	22	112	153	16.6	61	39	76	74.3
479	south-coast	2001	4	23	113	167	15.3	67	43	86	60
480	south-coast	2001	4	24	114	152	13.1	65	46	93	42.5
481	south-coast	2001	4	25	115	170	27.4	71	53	92	68.7
482	south-coast	2001	4	26	116	123	49.2	83	53	88	84.8
483	south-coast	2001	4	27	117	167	68.9	80	53	79	88.5
484	south-coast	2001	4	28	118	152	78.5	75	53	74	91.3
485	south-coast	2001	4	29	119	115	50.4	74	52	79	80.8
486	south-coast	2001	4	30	120	142	82.7	87	49	86	78.2
487	south-coast	2001	5	1	121	134	69.4	97	53	85	86.4
488	south-coast	2001	5	2	122	125	42.3	70	52	75	92
489	south-coast	2001	5	3	123	145	10.6	61	44	83	46.8
490	south-coast	2001	5	4	124	146	17.2	64	47	83	67.7
491	south-coast	2001	5	5	125	158	17.7	99	48	87	64.6
492	south-coast	2001	5	6	126	133	26.5	109	49	90	69.4
493	south-coast	2001	5	7	127	141	34.8	96	50	94	69.8
494	south-coast	2001	5	8	128	182	30.6	97	53	99	69.2
495	south-coast	2001	5	9	129	129	36.8	103	54	95	77.1
496	south-coast	2001	5	10	130	139	38.4	97	56	92	86.2
497	south-coast	2001	5	11	131	133	54.9	88	54	91	85.9
498	south-coast	2001	5	12	132	135	61.3	62	53	83	91.6
499	south-coast	2001	5	13	133	140	39.1	80	56	78	87.8
500	south-coast	2001	5	14	134	125	43.7	72	50	81	83.6
501	south-coast	2001	5	15	135	141	70	89	52	83	88.7
502	south-coast	2001	5	16	136	134	74.9	106	54	85	87.2
503	south-coast	2001	5	17	137	146	56.9	105	55	86	82.7
504	south-coast	2001	5	18	138	124	44.2	78	57	80	87.2
505	south-coast	2001	5	19	139	128	43.1	82	58	75	86.9
506	south-coast	2001	5	20	140	138	57.6	103	52	82	89.4
507	south-coast	2001	5	21	141	119	63	87	50	86	89.5
508	south-coast	2001	5	22	142	129	87.9	104	58	97	83.6
509	south-coast	2001	5	23	143	140	63	112	59	100	72.8
510	south-coast	2001	5	24	144	126	29	90	59	93	89.6
511	south-coast	2001	5	25	145	143	54.9	94	59	87	86.1
512	south-coast	2001	5	26	146	136	57.9	100	53	82	91.9
513	south-coast	2001	5	27	147	114	21.7	59	50	78	97.3
514	south-coast	2001	5	28	148	123	16.5	60	50	70	92.1
515	south-coast	2001	5	29	149	127	13.1	82	53	89	76.4
516	south-coast	2001	5	30	150	130	55.2	100	54	94	77.2
517	south-coast	2001	5	31	151	120	62.4	122	59	98	82.5
518	south-coast	2001	6	1	152	145	56.5	112	56	87	94.3
519	south-coast	2001	6	2	153	127	32.2	80	57	83	98.9
520	south-coast	2001	6	3	154	103	17.1	65	57	77	88.2
521	south-coast	2001	6	4	155	124	18.6	63	53	78	79.3
522	south-coast	2001	6	5	156	140	39.8	98	53	82	82
523	south-coast	2001	6	6	157	105	50.7	113	57	89	81.9
524	south-coast	2001	6	7	158	127	53.1	129	56	92	83.1
525	south-coast	2001	6	8	159	125	63.4	135	60	93	81.1

526	south-coast	2001	6	9	160	127	64.2	135	60	94	78.7
527	south-coast	2001	6	10	161	130	40.5	109	57	90	75.7
528	south-coast	2001	6	11	162	116	18.8	97	56	90	77.3
529	south-coast	2001	6	12	163	127	29.2	58	56	90	87.3
530	south-coast	2001	6	13	164	122	24.8	70	58	81	82.2
531	south-coast	2001	6	14	165	120	27.6	76	53	94	57.3
532	south-coast	2001	6	15	166	116	46.7	107	59	98	62.8
533	south-coast	2001	6	16	167	118	23.8	113	55	98	66.5
534	south-coast	2001	6	17	168	114	44.5	117	56	98	68.2
535	south-coast	2001	6	18	169	139	43.2	110	53	98	64.8
536	south-coast	2001	6	19	170	134	41.7	94	56	99	62.7
537	south-coast	2001	6	20	171	124	40.1	93	55	96	66.8
538	south-coast	2001	6	21	172	126	36.4	85	55	100	62.5
539	south-coast	2001	6	22	173	123	31.8	94	59	97	51.6
540	south-coast	2001	6	23	174	108	27.5	87	55	96	61
541	south-coast	2001	6	24	175	131	27.6	76	54	98	64.5
542	south-coast	2001	6	25	176	121	29.8	68	56	89	65.9
543	south-coast	2001	6	26	177	123	15.7	72	55	88	63.2
544	south-coast	2001	6	27	178	128	14	71	54	90	61.3
545	south-coast	2001	6	28	179	136	18.3	89	54	93	59.3
546	south-coast	2001	6	29	180	139	22	104	56	96	66.1
547	south-coast	2001	6	30	181	124	30.3	105	57	98	68.5
548	south-coast	2001	7	1	182	152	30.8	132	56	99	70.3
549	south-coast	2001	7	2	183	151	23.3	133	62	103	70.5
550	south-coast	2001	7	3	184	120	26	117	63	98	67.4
551	south-coast	2001	7	4	185	125	61.8	104	64	101	65.1
552	south-coast	2001	7	5	186	123	43	77	64	88	78.6
553	south-coast	2001	7	6	187	132	53.5	75	64	88	82.4
554	south-coast	2001	7	7	188	110	24.2	94	64	95	74.6
555	south-coast	2001	7	8	189	136	16	88	62	93	73.9
556	south-coast	2001	7	9	190	135	21	64	59	87	78.3
557	south-coast	2001	7	10	191	111	17.6	72	57	83	82.2
558	south-coast	2001	7	11	192	114	18.1	79	55	88	95.1
559	south-coast	2001	7	12	193	131	26.4	74	53	95	64.3
560	south-coast	2001	7	13	194	111	26.5	89	58	97	70.9
561	south-coast	2001	7	14	195	116	38.8	100	58	95	78.2
562	south-coast	2001	7	15	196	108	45.3	82	59	90	76.9
563	south-coast	2001	7	16	197	122	20.4	61	59	85	70.1
564	south-coast	2001	7	17	198	106	17.8	64	55	87	70.2
565	south-coast	2001	7	18	199	131	39.8	71	56	87	71.2
566	south-coast	2001	7	19	200	123	26.5	73	49	89	72.7
567	south-coast	2001	7	20	201	160	19.9	79	56	88	72.4
568	south-coast	2001	7	21	202	123	25.5	83	57	91	72.1
569	south-coast	2001	7	22	203	109	16.1	96	56	93	72.6
570	south-coast	2001	7	23	204	112	19.9	74	56	88	74.4
571	south-coast	2001	7	24	205	115	28.8	79	58	87	77.6
572	south-coast	2001	7	25	206	129	34.9	89	59	89	79.3
573	south-coast	2001	7	26	207	125	41.7	108	58	95	77.7
574	south-coast	2001	7	27	208	130	46.7	114	59	96	79.4

575	south-coast	2001	7	28	209	130	38.3	124	58	99	78.5
576	south-coast	2001	7	29	210	104	32.8	120	59	94	82
577	south-coast	2001	7	30	211	106	33.7	79	60	87	82.7
578	south-coast	2001	7	31	212	124	22.3	77	59	88	78.8
579	south-coast	2001	8	1	213	118	27.3	77	59	89	82.7
580	south-coast	2001	8	2	214	131	41.3	90	59	93	84.5
581	south-coast	2001	8	3	215	121	35.8	91	57	93	82.7
582	south-coast	2001	8	4	216	117	44.2	139	54	95	79.8
583	south-coast	2001	8	5	217	121	33.1	131	58	101	74.9
584	south-coast	2001	8	6	218	130	16.7	96	64	100	77
585	south-coast	2001	8	7	219	135	18.3	95	63	102	78
586	south-coast	2001	8	8	220	132	29.3	86	64	96	83
587	south-coast	2001	8	9	221	114	34	88	59	95	82.5
588	south-coast	2001	8	10	222	123	31.6	92	60	95	77.9
589	south-coast	2001	8	11	223	128	31.6	127	60	96	74.8
590	south-coast	2001	8	12	224	111	33.5	138	61	96	75.3
591	south-coast	2001	8	13	225	132	29.5	101	60	98	70.6
592	south-coast	2001	8	14	226	126	20	93	62	103	71.8
593	south-coast	2001	8	15	227	104	21.3	96	62	101	78.8
594	south-coast	2001	8	16	228	120	19.6	100	63	101	78.4
595	south-coast	2001	8	17	229	120	31.9	102	61	102	76.3
596	south-coast	2001	8	18	230	121	25	115	62	103	76.3
597	south-coast	2001	8	19	231	110	18.7	84	61	102	77.4
598	south-coast	2001	8	20	232	122	20.6	73	62	96	79
599	south-coast	2001	8	21	233	113	30.8	68	59	87	79.8
600	south-coast	2001	8	22	234	101	26.9	79	59	84	83.1
601	south-coast	2001	8	23	235	131	43.7	94	56	87	80.2
602	south-coast	2001	8	24	236	105	41.1	130	58	91	77.2
603	south-coast	2001	8	25	237	129	44.8	143	59	95	81.6
604	south-coast	2001	8	26	238	112	43	144	62	100	82.8
605	south-coast	2001	8	27	239	116	41.1	123	62	100	79.7
606	south-coast	2001	8	28	240	93	28	124	61	96	81.6
607	south-coast	2001	8	29	241	123	30.8	94	58	88	92.7
608	south-coast	2001	8	30	242	118	23.1	72	60	88	76.2
609	south-coast	2001	8	31	243	123	21.4	69	58	92	80.3
610	south-coast	2001	9	1	244	129	24.5	73	59	95	81.2
611	south-coast	2001	9	2	245	111	27	68	63	97	81.3
612	south-coast	2001	9	3	246	114	22.8	81	61	89	88.1
613	south-coast	2001	9	4	247	128	32.8	74	63	94	83.3
614	south-coast	2001	9	5	248	123	44.4	84	63	93	83.8
615	south-coast	2001	9	6	249	120	40.2	87	58	87	86.8
616	south-coast	2001	9	7	250	110	39.4	71	61	84	85.7
617	south-coast	2001	9	8	251	121	30.9	85	60	83	81.5
618	south-coast	2001	9	9	252	126	45.2	96	58	85	86.9
619	south-coast	2001	9	10	253	116	45.1	78	56	86	86.7
620	south-coast	2001	9	11	254	130	35.5	77	58	91	82.8
621	south-coast	2001	9	12	255	120	22.2	83	58	88	76.5
622	south-coast	2001	9	13	256	99	27.1	73	54	95	77.9
623	south-coast	2001	9	14	257	117	29.3	65	57	100	87.6

624	south-coast	2001	9	15	258	104	35.6	83	57	97	81.7
625	south-coast	2001	9	16	259	90	30.4	75	56	92	78.6
626	south-coast	2001	9	17	260	108	34.6	76	53	88	83.8
627	south-coast	2001	9	18	261	116	58.1	104	54	89	91.4
628	south-coast	2001	9	19	262	124	58.4	97	56	89	86.9
629	south-coast	2001	9	20	263	107	59.4	102	55	90	82.6
630	south-coast	2001	9	21	264	120	53	68	56	95	82.4
631	south-coast	2001	9	22	265	109	51.4	95	57	96	82.6
632	south-coast	2001	9	23	266	137	56.5	109	57	96	86.1
633	south-coast	2001	9	24	267	121	29.8	81	56	104	75.3
634	south-coast	2001	9	25	268	129	24.3	61	60	102	68.5
635	south-coast	2001	9	26	269	120	31.6	52	59	101	74.8
636	south-coast	2001	9	27	270	109	41.3	70	59	99	82
637	south-coast	2001	9	28	271	143	43	76	58	97	78.8
638	south-coast	2001	9	29	272	130	43.3	90	57	103	79.7
639	south-coast	2001	9	30	273	122	31	97	60	106	73.5
640	south-coast	2001	10	1	274	131	26.7	65	63	104	74.5
641	south-coast	2001	10	2	275	132	36.8	66	60	97	84.5
642	south-coast	2001	10	3	276	127	42.5	73	60	89	84.1
643	south-coast	2001	10	4	277	124	45.4	68	58	88	84.8
644	south-coast	2001	10	5	278	120	23.1	51	56	81	84.6
645	south-coast	2001	10	6	279	121	18.6	59	57	78	81
646	south-coast	2001	10	7	280	111	29.7	63	51	80	77.8
647	south-coast	2001	10	8	281	115	38.8	71	53	83	85.5
648	south-coast	2001	10	9	282	122	32.5	64	46	81	87
649	south-coast	2001	10	10	283	117	45.7	61	50	91	83.3
650	south-coast	2001	10	11	284	112	65.9	75	52	88	84.5
651	south-coast	2001	10	12	285	122	48	60	52	93	75.8
652	south-coast	2001	10	13	286	118	49.3	78	54	96	83.9
653	south-coast	2001	10	14	287	97	62.7	93	54	99	83.3
654	south-coast	2001	10	15	288	123	76.4	71	53	94	84.4
655	south-coast	2001	10	16	289	138	73.4	76	56	94	83.6
656	south-coast	2001	10	17	290	105	47.3	68	53	87	83.5
657	south-coast	2001	10	18	291	124	39	57	48	90	80.8
658	south-coast	2001	10	19	292	122	51.7	59	50	88	84
659	south-coast	2001	10	20	293	116	63.5	84	52	85	89.3
660	south-coast	2001	10	21	294	117	63.8	76	51	83	92.4
661	south-coast	2001	10	22	295	102	72.2	65	55	85	88.5
662	south-coast	2001	10	23	296	107	99.3	70	50	84	90.3
663	south-coast	2001	10	24	297	126	98.6	56	49	86	88
664	south-coast	2001	10	25	298	131	101.3	67	51	89	83.8
665	south-coast	2001	10	26	299	128	74.3	64	52	89	87
666	south-coast	2001	10	27	300	116	61.2	72	50	87	86.2
667	south-coast	2001	10	28	301	135	43	48	51	78	97
668	south-coast	2001	10	29	302	105	39	47	47	84	78.6
669	south-coast	2001	10	30	303	144	31.4	45	52	82	92.2
670	south-coast	2001	10	31	304	119	25.5	38	53	78	92.5
671	south-coast	2001	11	1	305	121	41.7	49	50	80	85.9
672	south-coast	2001	11	2	306	128	63.9	51	48	78	89.8

673 south-coast	2001	11	3	307	147	82.4	57	48	85	85.5
674 south-coast	2001	11	4	308	131	98	42	50	79	94.8
675 south-coast	2001	11	5	309	138	94.6	43	50	85	89.9
676 south-coast	2001	11	6	310	134	104.1	61	52	80	93.1
677 south-coast	2001	11	7	311	120	94.8	51	51	82	84.4
678 south-coast	2001	11	8	312	132	59.6	48	52	90	82.1
679 south-coast	2001	11	9	313	125	73.4	55	51	85	86.9
680 south-coast	2001	11	10	314	156	50.8	56	53	80	86.9
681 south-coast	2001	11	11	315	124	34.9	48	52	84	96.4
682 south-coast	2001	11	12	316	113	25.9	44	53	74	94
683 south-coast	2001	11	13	317	125	16.3	40	45	66	90.1
684 south-coast	2001	11	14	318	114	31.2	45	44	79	78
685 south-coast	2001	11	15	319	123	51.9	45	46	80	71.2
686 south-coast	2001	11	16	320	132	49.7	46	47	79	80.8
687 south-coast	2001	11	17	321	148	68.8	55	48	81	88.3
688 south-coast	2001	11	18	322	121	60.2	50	45	81	86.6
689 south-coast	2001	11	19	323	135	53.4	55	46	82	73.9
690 south-coast	2001	11	20	324	131	46.3	50	49	81	71.8
691 south-coast	2001	11	21	325	100	52.4	39	48	72	79
692 south-coast	2001	11	22	326	147	37.6	38	46	68	91.3
693 south-coast	2001	11	23	327	127	28.6	46	47	69	76
694 south-coast	2001	11	24	328	151	20.3	40	44	67	92.5
695 south-coast	2001	11	25	329	122	13.3	40	43	64	84
696 south-coast	2001	11	26	330	117	23.8	40	38	68	80.4
697 south-coast	2001	11	27	331	143	23.9	43	38	66	56
698 south-coast	2001	11	28	332	137	21.6	35	33	66	41.7
699 south-coast	2001	11	29	333	154	20.6	40	43	60	77.5
700 south-coast	2001	11	30	334	150	32.3	35	42	61	87.7
701 south-coast	2001	12	1	335	138	40.8	48	35	68	81.8
702 south-coast	2001	12	2	336	142	52.4	50	41	67	86.2
703 south-coast	2001	12	3	337	144	19.5	39	43	61	91.6
704 south-coast	2001	12	4	338	153	19.3	39	40	62	80.3
705 south-coast	2001	12	5	339	130	31.9	29	35	62	77.5
706 south-coast	2001	12	6	340	148	34.8	39	39	73	61.3
707 south-coast	2001	12	7	341	140	23.2	47	41	82	38.1
708 south-coast	2001	12	8	342	148	18	43	40	82	32.3
709 south-coast	2001	12	9	343	159	17.3	48	38	80	59
710 south-coast	2001	12	10	344	141	13.1	40	37	60	88
711 south-coast	2001	12	11	345	132	17.7	42	36	61	70
712 south-coast	2001	12	12	346	145	38.4	40	39	62	59
713 south-coast	2001	12	13	347	127	32.6	32	35	61	73.4
714 south-coast	2001	12	14	348	141	30.1	36	38	60	93.6
715 south-coast	2001	12	15	349	174	32	45	34	63	65.7
716 south-coast	2001	12	16	350	136	25.9	41	33	65	54
717 south-coast	2001	12	17	351	123	31.3	45	36	65	67.4
718 south-coast	2001	12	18	352	134	44.4	32	36	73	74.3
719 south-coast	2001	12	19	353	140	56.8	50	38	77	81.9
720 south-coast	2001	12	20	354	147	33.3	38	41	75	85.9
721 south-coast	2001	12	21	355	168	15.9	39	41	61	99.3

722	south-coast	2001	12	22	356	131	38.9	35	39	63	89.7
723	south-coast	2001	12	23	357	156	45.5	43	37	59	88.5
724	south-coast	2001	12	24	358	148	49.7	45	38	65	61
725	south-coast	2001	12	25	359	152	29.2	39	38	69	52.7
726	south-coast	2001	12	26	360	134	32.4	32	37	74	63
727	south-coast	2001	12	27	361	139	49.9	36	40	70	79.1
728	south-coast	2001	12	28	362	143	67.1	36	41	71	85.2
729	south-coast	2001	12	29	363	165	58.2	38	49	69	90.2
730	south-coast	2001	12	30	364	152	24.2	28	44	65	95.7
731	south-coast	2001	12	31	365	153	42	36	46	65	99.5
732	south-coast	2002	1	1	1	162	66.3	37	43	64	89.6
733	south-coast	2002	1	2	2	144	74.4	42	42	65	93.1
734	south-coast	2002	1	3	3	147	51.4	44	49	61	93.8
735	south-coast	2002	1	4	4	144	57.3	43	41	72	87.5
736	south-coast	2002	1	5	5	132	43.6	34	40	73	69
737	south-coast	2002	1	6	6	149	35	30	43	76	65.9
738	south-coast	2002	1	7	7	167	38.2	27	46	80	72.3
739	south-coast	2002	1	8	8	161	37.7	32	44	82	74.3
740	south-coast	2002	1	9	9	144	41.5	41	48	72	84.5
741	south-coast	2002	1	10	10	156	42.1	49	43	80	80.3
742	south-coast	2002	1	11	11	145	28.2	43	42	82	63
743	south-coast	2002	1	12	12	133	31.4	38	40	82	52.4
744	south-coast	2002	1	13	13	151	54.9	44	41	80	81.1
745	south-coast	2002	1	14	14	163	54.9	49	38	76	86.8
746	south-coast	2002	1	15	15	142	28.3	42	38	62	79.6
747	south-coast	2002	1	16	16	147	21.5	45	45	61	80
748	south-coast	2002	1	17	17	144	31.8	42	35	62	72.9
749	south-coast	2002	1	18	18	137	42.9	44	34	66	64.8
750	south-coast	2002	1	19	19	156	27	44	35	69	48.8
751	south-coast	2002	1	20	20	172	36.5	45	35	68	63
752	south-coast	2002	1	21	21	157	39.5	42	36	70	58.5
753	south-coast	2002	1	22	22	145	27.3	43	37	65	61.1
754	south-coast	2002	1	23	23	163	17.7	46	37	63	63
755	south-coast	2002	1	24	24	148	27	45	38	71	41.6
756	south-coast	2002	1	25	25	145	24.6	39	38	75	47.2
757	south-coast	2002	1	26	26	124	37.4	48	40	78	78.3
758	south-coast	2002	1	27	27	142	25.5	48	37	64	92.5
759	south-coast	2002	1	28	28	152	11.3	44	38	58	80.3
760	south-coast	2002	1	29	29	151	18.3	41	34	56	85.7
761	south-coast	2002	1	30	30	147	14	45	32	59	50.3
762	south-coast	2002	1	31	31	158	25.1	41	29	61	44.3
763	south-coast	2002	2	1	32	173	32.1	39	32	63	63
764	south-coast	2002	2	2	33	176	33.8	42	33	69	58.2
765	south-coast	2002	2	3	34	140	39.8	47	35	75	59.2
766	south-coast	2002	2	4	35	154	25	48	35	76	63
767	south-coast	2002	2	5	36	140	49.1	43	37	77	61.8
768	south-coast	2002	2	6	37	156	51.8	45	36	74	72.9
769	south-coast	2002	2	7	38	150	60.9	47	38	73	82.3
770	south-coast	2002	2	8	39	161	65	47	38	67	84.3

771	south-coast	2002	2	9	40	136	30.5	52	41	80	49.3
772	south-coast	2002	2	10	41	158	17.6	52	36	81	63
773	south-coast	2002	2	11	42	161	11.4	50	38	79	39.1
774	south-coast	2002	2	12	43	166	39.5	46	39	86	46.5
775	south-coast	2002	2	13	44	149	48.4	48	40	80	76.5
776	south-coast	2002	2	14	45	163	32.5	49	42	71	82.6
777	south-coast	2002	2	15	46	145	51.1	53	45	78	84.1
778	south-coast	2002	2	16	47	175	58.9	56	40	75	86.1
779	south-coast	2002	2	17	48	136	12.8	48	43	69	99.2
780	south-coast	2002	2	18	49	159	18.5	50	40	62	87.3
781	south-coast	2002	2	19	50	154	28.2	47	39	64	81
782	south-coast	2002	2	20	51	173	54.4	49	43	73	83.6
783	south-coast	2002	2	21	52	176	29.8	50	44	87	54.1
784	south-coast	2002	2	22	53	185	14.7	52	47	89	63
785	south-coast	2002	2	23	54	166	23.5	64	45	91	73.5
786	south-coast	2002	2	24	55	143	28.8	55	42	83	78.6
787	south-coast	2002	2	25	56	158	55.3	52	44	88	83.8
788	south-coast	2002	2	26	57	151	32	50	45	87	60
789	south-coast	2002	2	27	58	154	37.6	52	50	86	71.4
790	south-coast	2002	2	28	59	138	48	62	44	82	84.8
791	south-coast	2002	3	1	60	175	30.6	52	44	71	67.9
792	south-coast	2002	3	2	61	155	21.3	51	38	77	62.1
793	south-coast	2002	3	3	62	139	29.2	54	35	77	64
794	south-coast	2002	3	4	63	154	13.5	53	36	82	35.3
795	south-coast	2002	3	5	64	144	33.5	61	43	77	72.5
796	south-coast	2002	3	6	65	139	36.3	54	42	70	82.2
797	south-coast	2002	3	7	66	140	13	43	48	67	99
798	south-coast	2002	3	8	67	147	15.3	54	46	65	73.3
799	south-coast	2002	3	9	68	152	26	57	41	78	64
800	south-coast	2002	3	10	69	150	17.6	60	43	79	72.1
801	south-coast	2002	3	11	70	134	27	55	43	84	77.5
802	south-coast	2002	3	12	71	150	45.3	68	48	80	82.3
803	south-coast	2002	3	13	72	163	19.3	60	49	73	71.2
804	south-coast	2002	3	14	73	139	9.5	52	37	66	46.9
805	south-coast	2002	3	15	74	145	15.3	59	35	65	64
806	south-coast	2002	3	16	75	152	11.2	57	38	59	75.2
807	south-coast	2002	3	17	76	129	13.7	56	35	65	83.5
808	south-coast	2002	3	18	77	155	10	56	38	69	67.4
809	south-coast	2002	3	19	78	144	21.6	52	37	77	50.6
810	south-coast	2002	3	20	79	177	28.3	53	40	88	54.5
811	south-coast	2002	3	21	80	163	32.6	65	45	86	66.5
812	south-coast	2002	3	22	81	138	28.3	65	43	79	76
813	south-coast	2002	3	23	82	148	10.9	50	42	69	86.5
814	south-coast	2002	3	24	83	164	10.6	53	41	72	87.1
815	south-coast	2002	3	25	84	144	15.7	54	41	71	72.5
816	south-coast	2002	3	26	85	164	26.6	61	43	80	73.7
817	south-coast	2002	3	27	86	142	26.6	57	44	75	79.7
818	south-coast	2002	3	28	87	140	19	48	51	64	95
819	south-coast	2002	3	29	88	151	34.1	52	45	67	87.6

820	south-coast	2002	3	30	89	127	69.6	64	44	76	85.4
821	south-coast	2002	3	31	90	152	67.6	92	44	83	87.2
822	south-coast	2002	4	1	91	153	80.2	82	45	83	87.3
823	south-coast	2002	4	2	92	149	78.1	74	48	75	86.9
824	south-coast	2002	4	3	93	159	73.2	70	50	72	87.2
825	south-coast	2002	4	4	94	117	61.6	69	50	75	75.8
826	south-coast	2002	4	5	95	160	26.7	51	45	67	89.3
827	south-coast	2002	4	6	96	133	17.6	49	42	61	99.6
828	south-coast	2002	4	7	97	133	18.4	56	43	69	87.1
829	south-coast	2002	4	8	98	148	31.8	55	49	70	83.3
830	south-coast	2002	4	9	99	151	32.3	72	46	78	84.8
831	south-coast	2002	4	10	100	141	41.4	80	48	82	80
832	south-coast	2002	4	11	101	150	63.2	92	50	85	83.2
833	south-coast	2002	4	12	102	121	67	80	53	80	87.3
834	south-coast	2002	4	13	103	141	75.8	81	51	99	82
835	south-coast	2002	4	14	104	146	72	101	47	90	88.4
836	south-coast	2002	4	15	105	129	14.8	64	54	80	92
837	south-coast	2002	4	16	106	133	17.1	60	47	68	83
838	south-coast	2002	4	17	107	140	16.7	60	45	67	80.7
839	south-coast	2002	4	18	108	143	13.8	63	43	69	75.1
840	south-coast	2002	4	19	109	127	15.5	64	43	69	65.9
841	south-coast	2002	4	20	110	131	19.8	65	42	75	74.3
842	south-coast	2002	4	21	111	133	22.6	84	44	84	67.3
843	south-coast	2002	4	22	112	126	33.4	83	48	89	70
844	south-coast	2002	4	23	113	128	26.3	80	50	87	74.7
845	south-coast	2002	4	24	114	134	29.7	51	45	78	86.3
846	south-coast	2002	4	25	115	131	13	51	51	73	82.2
847	south-coast	2002	4	26	116	115	12.9	56	48	70	99
848	south-coast	2002	4	27	117	126	17.4	52	48	66	88.6
849	south-coast	2002	4	28	118	127	12.9	59	45	79	67.9
850	south-coast	2002	4	29	119	137	11.9	47	47	74	71.3
851	south-coast	2002	4	30	120	135	11.4	55	48	70	76.2
852	south-coast	2002	5	1	121	132	14.3	62	46	67	79.1
853	south-coast	2002	5	2	122	125	17.8	60	45	73	79.4
854	south-coast	2002	5	3	123	140	24.9	68	45	78	77.5
855	south-coast	2002	5	4	124	122	32.3	79	46	81	81.8
856	south-coast	2002	5	5	125	110	36.6	82	45	79	83.5
857	south-coast	2002	5	6	126	125	40.2	66	45	78	84.5
858	south-coast	2002	5	7	127	118	30.3	57	54	74	89.3
859	south-coast	2002	5	8	128	137	29.4	66	53	76	80
860	south-coast	2002	5	9	129	145	35.2	84	49	82	80.5
861	south-coast	2002	5	10	130	143	27.6	73	50	76	80
862	south-coast	2002	5	11	131	138	23.9	73	49	80	70.6
863	south-coast	2002	5	12	132	163	24.3	77	50	96	54.7
864	south-coast	2002	5	13	133	145	27.8	90	49	95	58.4
865	south-coast	2002	5	14	134	129	30.3	90	53	89	70.4
866	south-coast	2002	5	15	135	151	31.6	77	48	80	81.9
867	south-coast	2002	5	16	136	133	29.5	70	55	75	85.9
868	south-coast	2002	5	17	137	120	51.2	82	51	85	82.4

869	south-coast	2002	5	18	138	121	49.4	101	46	80	91.3
870	south-coast	2002	5	19	139	118	24.7	77	47	75	88.7
871	south-coast	2002	5	20	140	116	10.6	51	53	68	84.5
872	south-coast	2002	5	21	141	107	13.3	55	46	70	74.5
873	south-coast	2002	5	22	142	140	19	66	45	81	75.6
874	south-coast	2002	5	23	143	120	23.5	76	48	80	70.7
875	south-coast	2002	5	24	144	146	29	85	50	82	79.5
876	south-coast	2002	5	25	145	150	28.1	94	48	84	79.8
877	south-coast	2002	5	26	146	132	29.2	88	49	80	79.7
878	south-coast	2002	5	27	147	112	33.9	94	51	80	81.2
879	south-coast	2002	5	28	148	134	41.5	80	50	89	79.7
880	south-coast	2002	5	29	149	140	50.2	97	54	101	81.3
881	south-coast	2002	5	30	150	126	49.5	103	56	96	83.8
882	south-coast	2002	5	31	151	133	42.8	85	58	90	79.2
883	south-coast	2002	6	1	152	128	39.6	78	53	91	78.6
884	south-coast	2002	6	2	153	149	23.6	75	51	80	79.3
885	south-coast	2002	6	3	154	129	22.3	68	56	76	79.7
886	south-coast	2002	6	4	155	123	38.2	90	52	94	77.6
887	south-coast	2002	6	5	156	123	42.7	108	56	104	81
888	south-coast	2002	6	6	157	119	45.1	110	58	98	81
889	south-coast	2002	6	7	158	109	37.7	100	59	89	91.3
890	south-coast	2002	6	8	159	119	32.6	90	57	84	86.4
891	south-coast	2002	6	9	160	124	17.8	73	53	83	86.9
892	south-coast	2002	6	10	161	113	21.4	66	56	83	79.5
893	south-coast	2002	6	11	162	104	23.9	73	56	85	78.8
894	south-coast	2002	6	12	163	123	28.1	94	52	90	75.2
895	south-coast	2002	6	13	164	138	30.2	93	51	90	75
896	south-coast	2002	6	14	165	126	28.1	97	53	92	74.8
897	south-coast	2002	6	15	166	138	25.4	138	52	99	72.9
898	south-coast	2002	6	16	167	130	28.5	131	53	99	74
899	south-coast	2002	6	17	168	137	37	131	50	95	75.8
900	south-coast	2002	6	18	169	136	34.1	113	55	93	77.8
901	south-coast	2002	6	19	170	122	30.7	110	58	91	82
902	south-coast	2002	6	20	171	129	25.4	74	58	87	89.3
903	south-coast	2002	6	21	172	118	20.8	63	58	80	91.5
904	south-coast	2002	6	22	173	111	34.9	104	56	91	78.5
905	south-coast	2002	6	23	174	119	34.7	131	57	95	82
906	south-coast	2002	6	24	175	131	44.8	113	54	95	81.9
907	south-coast	2002	6	25	176	112	51.3	99	58	95	79.1
908	south-coast	2002	6	26	177	119	33.6	87	53	95	81.4
909	south-coast	2002	6	27	178	112	27.9	78	55	90	80.4
910	south-coast	2002	6	28	179	136	25.7	86	56	90	78.1
911	south-coast	2002	6	29	180	128	31.9	128	53	94	79.7
912	south-coast	2002	6	30	181	127	30.5	120	54	96	82.2
913	south-coast	2002	7	1	182	120	39	93	58	99	81.2
914	south-coast	2002	7	2	183	124	38.5	85	57	99	79.3
915	south-coast	2002	7	3	184	117	30.4	82	57	92	83
916	south-coast	2002	7	4	185	136	41.4	107	55	92	83.6
917	south-coast	2002	7	5	186	119	60	102	58	89	82.9

918	south-coast	2002	7	6	187	137	40.6	113	58	93	83.7
919	south-coast	2002	7	7	188	137	48.4	122	59	97	83.4
920	south-coast	2002	7	8	189	129	36.5	120	59	105	82.9
921	south-coast	2002	7	9	190	134	34.7	139	60	106	80.3
922	south-coast	2002	7	10	191	115	29.3	144	61	103	79.5
923	south-coast	2002	7	11	192	135	32.3	81	64	93	85.8
924	south-coast	2002	7	12	193	140	25.9	120	61	102	81.1
925	south-coast	2002	7	13	194	123	21.5	120	65	99	78
926	south-coast	2002	7	14	195	108	28.9	117	65	101	81.3
927	south-coast	2002	7	15	196	125	29.5	83	59	97	82.6
928	south-coast	2002	7	16	197	134	26	62	62	94	81.2
929	south-coast	2002	7	17	198	119	24.7	78	61	94	80.9
930	south-coast	2002	7	18	199	116	25.3	83	64	92	80.9
931	south-coast	2002	7	19	200	138	41.8	83	62	91	89.5
932	south-coast	2002	7	20	201	109	41.5	109	61	90	87.5
933	south-coast	2002	7	21	202	115	31.5	107	59	89	84
934	south-coast	2002	7	22	203	114	41.7	99	59	93	77.1
935	south-coast	2002	7	23	204	124	23.8	93	57	102	69.9
936	south-coast	2002	7	24	205	125	22.6	81	62	101	67.3
937	south-coast	2002	7	25	206	129	20.8	99	60	100	67.8
938	south-coast	2002	7	26	207	145	25.9	97	58	98	83.3
939	south-coast	2002	7	27	208	132	22.4	86	59	92	79.1
940	south-coast	2002	7	28	209	105	25.3	88	57	92	74.8
941	south-coast	2002	7	29	210	113	26.2	106	57	94	81.5
942	south-coast	2002	7	30	211	127	34.8	113	60	96	84.7
943	south-coast	2002	7	31	212	129	41.8	125	62	96	84.2
944	south-coast	2002	8	1	213	126	30.5	126	62	94	84.4
945	south-coast	2002	8	2	214	90	27.6	93	57	91	88.9
946	south-coast	2002	8	3	215	127	36.4	88	62	91	92.7
947	south-coast	2002	8	4	216	110	33.1	99	58	97	79.6
948	south-coast	2002	8	5	217	107	31	69	58	95	84.6
949	south-coast	2002	8	6	218	118	23.2	62	55	93	81.2
950	south-coast	2002	8	7	219	111	21.8	76	56	96	74.9
951	south-coast	2002	8	8	220	124	21.5	109	58	101	77.7
952	south-coast	2002	8	9	221	101	24.6	97	57	106	82.4
953	south-coast	2002	8	10	222	116	27.9	124	59	105	82.8
954	south-coast	2002	8	11	223	112	28.5	142	61	103	81.4
955	south-coast	2002	8	12	224	131	33	121	65	101	84.7
956	south-coast	2002	8	13	225	130	31.3	117	61	99	85.5
957	south-coast	2002	8	14	226	112	38.4	111	60	97	86.7
958	south-coast	2002	8	15	227	119	53.5	117	62	94	87
959	south-coast	2002	8	16	228	108	53.5	116	61	90	88.5
960	south-coast	2002	8	17	229	132	60.5	130	57	89	92.8
961	south-coast	2002	8	18	230	124	63.6	115	56	87	92.8
962	south-coast	2002	8	19	231	134	36	91	62	85	92.7
963	south-coast	2002	8	20	232	114	42.5	94	63	82	92.6
964	south-coast	2002	8	21	233	127	54.4	89	60	84	87.1
965	south-coast	2002	8	22	234	110	42.7	96	58	89	76.1
966	south-coast	2002	8	23	235	131	44.1	90	55	92	75.9

967	south-coast	2002	8	24	236	138	28.5	97	54	97	76.1
968	south-coast	2002	8	25	237	120	24.3	113	52	97	77.5
969	south-coast	2002	8	26	238	123	29.1	101	53	96	80.2
970	south-coast	2002	8	27	239	118	26.6	110	60	96	86.9
971	south-coast	2002	8	28	240	96	27.7	75	58	96	81
972	south-coast	2002	8	29	241	97	30.4	78	56	94	80
973	south-coast	2002	8	30	242	125	31.5	75	55	100	78.1
974	south-coast	2002	8	31	243	129	34.3	82	58	103	78.6
975	south-coast	2002	9	1	244	131	19.1	84	66	109	60.4
976	south-coast	2002	9	2	245	148	22.8	99	63	107	66.5
977	south-coast	2002	9	3	246	126	24.7	92	63	102	81.9
978	south-coast	2002	9	4	247	94	28.4	92	64	100	80.8
979	south-coast	2002	9	5	248	123	43.4	70	66	91	81.8
980	south-coast	2002	9	6	249	136	14.2	43	66	85	78.9
981	south-coast	2002	9	7	250	131	11.2	54	60	96	71.7
982	south-coast	2002	9	8	251	126	14	65	53	89	70.6
983	south-coast	2002	9	9	252	115	19.1	67	59	96	68.6
984	south-coast	2002	9	10	253	122	19.2	75	60	99	72.7
985	south-coast	2002	9	11	254	120	26.9	86	57	94	82.1
986	south-coast	2002	9	12	255	103	35.9	91	56	95	85.9
987	south-coast	2002	9	13	256	150	26.5	99	56	90	82.7
988	south-coast	2002	9	14	257	104	38.8	105	55	91	87.6
989	south-coast	2002	9	15	258	118	33.8	108	55	94	81.3
990	south-coast	2002	9	16	259	124	16.4	74	54	92	80.3
991	south-coast	2002	9	17	260	111	29.3	80	57	83	86.3
992	south-coast	2002	9	18	261	125	31.8	76	62	82	79.5
993	south-coast	2002	9	19	262	133	28.9	65	55	100	83.8
994	south-coast	2002	9	20	263	104	51.7	89	58	96	85.5
995	south-coast	2002	9	21	264	109	26.8	106	57	98	82.1
996	south-coast	2002	9	22	265	106	18.1	123	56	106	72.4
997	south-coast	2002	9	23	266	133	29.8	81	60	107	74.1
998	south-coast	2002	9	24	267	133	39.6	111	58	102	86.6
999	south-coast	2002	9	25	268	106	53	85	63	96	86.5
1000	south-coast	2002	9	26	269	126	59.4	86	62	90	87.2
1001	south-coast	2002	9	27	270	101	41.3	52	56	81	85.6
1002	south-coast	2002	9	28	271	110	10.3	36	60	76	95.4
1003	south-coast	2002	9	29	272	114	7.5	49	53	76	84.1
1004	south-coast	2002	9	30	273	141	12.4	46	53	77	82.9
1005	south-coast	2002	10	1	274	126	12	49	58	76	87.9
1006	south-coast	2002	10	2	275	111	18.3	51	48	73	77
1007	south-coast	2002	10	3	276	113	25	58	47	79	70.4
1008	south-coast	2002	10	4	277	135	29.8	77	49	85	73.3
1009	south-coast	2002	10	5	278	145	29.1	75	51	91	70.3
1010	south-coast	2002	10	6	279	155	17.1	68	52	97	65.3
1011	south-coast	2002	10	7	280	137	20.7	70	55	97	70.2
1012	south-coast	2002	10	8	281	152	38.2	84	52	94	83.5
1013	south-coast	2002	10	9	282	146	59.6	83	56	91	85.3
1014	south-coast	2002	10	10	283	138	57.7	74	53	83	89.5
1015	south-coast	2002	10	11	284	129	56.9	62	55	76	83.3

1016	south-coast	2002	10	12	285	118	41.4	62	53	75	88.6
1017	south-coast	2002	10	13	286	140	56.9	62	50	90	90.8
1018	south-coast	2002	10	14	287	106	82.1	65	51	81	92
1019	south-coast	2002	10	15	288	132	72.4	85	52	74	93.4
1020	south-coast	2002	10	16	289	117	61.4	61	56	73	92.3
1021	south-coast	2002	10	17	290	99	36.3	58	54	70	96.8
1022	south-coast	2002	10	18	291	136	42.8	58	55	70	88.8
1023	south-coast	2002	10	19	292	134	55.2	68	49	78	85.7
1024	south-coast	2002	10	20	293	124	70.8	76	49	78	93.8
1025	south-coast	2002	10	21	294	139	67.3	64	51	71	93.4
1026	south-coast	2002	10	22	295	122	47.8	56	55	70	86.7
1027	south-coast	2002	10	23	296	139	43.1	58	52	69	85.1
1028	south-coast	2002	10	24	297	122	53.2	62	55	71	85.1
1029	south-coast	2002	10	25	298	138	47.5	63	54	68	87.3
1030	south-coast	2002	10	26	299	144	47.4	47	50	69	92.3
1031	south-coast	2002	10	27	300	137	27.1	60	48	70	97.3
1032	south-coast	2002	10	28	301	134	39.9	63	45	74	88.3
1033	south-coast	2002	10	29	302	136	40.3	57	52	70	90
1034	south-coast	2002	10	30	303	143	34.1	52	50	70	88.9
1035	south-coast	2002	10	31	304	114	48.7	52	52	71	91.9
1036	south-coast	2002	11	1	305	127	66.3	54	50	70	86.3
1037	south-coast	2002	11	2	306	143	49.7	65	48	76	87.1
1038	south-coast	2002	11	3	307	126	38.4	52	45	82	75.9
1039	south-coast	2002	11	4	308	144	38.6	65	41	75	83.3
1040	south-coast	2002	11	5	309	123	58.8	51	42	81	85.4
1041	south-coast	2002	11	6	310	137	64.7	51	45	83	80.5
1042	south-coast	2002	11	7	311	113	62.6	45	47	78	86.9
1043	south-coast	2002	11	8	312	119	10.7	29	48	71	100
1044	south-coast	2002	11	9	313	144	4.9	27	49	66	100
1045	south-coast	2002	11	10	314	121	20.4	46	50	71	86.9
1046	south-coast	2002	11	11	315	133	14.3	48	47	80	66
1047	south-coast	2002	11	12	316	132	10.9	48	47	86	49
1048	south-coast	2002	11	13	317	141	25.9	42	48	80	69.2
1049	south-coast	2002	11	14	318	132	45.2	47	47	85	88
1050	south-coast	2002	11	15	319	148	18	48	47	87	44.5
1051	south-coast	2002	11	16	320	138	15.4	47	45	85	56
1052	south-coast	2002	11	17	321	135	25.5	47	46	82	67.1
1053	south-coast	2002	11	18	322	148	21.6	52	44	85	47
1054	south-coast	2002	11	19	323	132	22.1	47	45	90	56
1055	south-coast	2002	11	20	324	135	18.1	43	52	94	36.4
1056	south-coast	2002	11	21	325	128	14.7	50	51	91	44.6
1057	south-coast	2002	11	22	326	148	36.3	51	48	82	70.1
1058	south-coast	2002	11	23	327	131	38.9	52	43	71	86.6
1059	south-coast	2002	11	24	328	150	48.1	45	43	72	90.1
1060	south-coast	2002	11	25	329	129	36	50	44	74	56
1061	south-coast	2002	11	26	330	153	13.5	50	44	76	29
1062	south-coast	2002	11	27	331	134	19	47	46	72	48.3
1063	south-coast	2002	11	28	332	132	19.8	47	49	80	56
1064	south-coast	2002	11	29	333	144	9.6	36	47	71	75.8

1065	south-coast	2002	11	30	334	137	15.6	38	49	68	94.2
1066	south-coast	2002	12	1	335	127	23.8	47	42	70	81.5
1067	south-coast	2002	12	2	336	127	20.5	46	42	73	71.1
1068	south-coast	2002	12	3	337	140	34.4	43	42	70	84.3
1069	south-coast	2002	12	4	338	112	55.2	43	41	72	85
1070	south-coast	2002	12	5	339	141	45.2	41	43	75	70
1071	south-coast	2002	12	6	340	132	34.4	42	43	72	81.5
1072	south-coast	2002	12	7	341	132	57.7	54	44	69	87.5
1073	south-coast	2002	12	8	342	121	52.9	45	40	72	84.5
1074	south-coast	2002	12	9	343	116	45.7	38	41	69	81.1
1075	south-coast	2002	12	10	344	128	45	45	39	68	87.3
1076	south-coast	2002	12	11	345	138	30.8	43	43	74	74.7
1077	south-coast	2002	12	12	346	136	36.6	42	41	74	71.5
1078	south-coast	2002	12	13	347	158	49.7	45	42	71	80.9
1079	south-coast	2002	12	14	348	147	51.8	47	44	69	79.5
1080	south-coast	2002	12	15	349	159	36.1	41	43	68	93.2
1081	south-coast	2002	12	16	350	156	17.4	42	42	68	95.2
1082	south-coast	2002	12	17	351	145	7	43	40	67	98.8
1083	south-coast	2002	12	18	352	157	18.2	42	36	66	77.3
1084	south-coast	2002	12	19	353	151	20.7	45	32	64	72.4
1085	south-coast	2002	12	20	354	160	9.6	44	35	65	91.7
1086	south-coast	2002	12	21	355	129	12.6	42	42	64	98
1087	south-coast	2002	12	22	356	168	21.6	43	40	67	75.5
1088	south-coast	2002	12	23	357	135	20.9	39	35	65	68.7
1089	south-coast	2002	12	24	358	143	32.7	42	33	65	74.3
1090	south-coast	2002	12	25	359	157	55.4	40	33	66	68.7
1091	south-coast	2002	12	26	360	139	49.2	39	37	67	67.2
1092	south-coast	2002	12	27	361	127	30.9	45	38	72	66.4
1093	south-coast	2002	12	28	362	150	21.7	41	40	72	70.6
1094	south-coast	2002	12	29	363	128	10.2	45	40	74	92.3
1095	south-coast	2002	12	30	364	157	18.7	44	36	68	78.2
1096	south-coast	2002	12	31	365	161	37.2	42	39	69	90.5
1097	south-coast	2003	1	1	1	132	44	46	37	80	69.5
1098	south-coast	2003	1	2	2	156	13.2	45	40	83	46
1099	south-coast	2003	1	3	3	177	35	45	45	83	63
1100	south-coast	2003	1	4	4	166	17	34	45	83	50.8
1101	south-coast	2003	1	5	5	185	32.6	46	46	84	58.5
1102	south-coast	2003	1	6	6	157	13	46	38	82	63
1103	south-coast	2003	1	7	7	183	8.6	46	49	84	40.4
1104	south-coast	2003	1	8	8	177	16.3	35	50	79	80.5
1105	south-coast	2003	1	9	9	125	35.3	37	46	67	92.8
1106	south-coast	2003	1	10	10	147	28.7	39	44	65	95.6
1107	south-coast	2003	1	11	11	148	43.7	41	43	67	86.9
1108	south-coast	2003	1	12	12	159	59.5	48	42	72	94.5
1109	south-coast	2003	1	13	13	116	46.8	43	43	79	91
1110	south-coast	2003	1	14	14	140	44.5	42	43	75	94
1111	south-coast	2003	1	15	15	158	35.6	46	42	79	63
1112	south-coast	2003	1	16	16	134	14.2	45	40	85	44.5
1113	south-coast	2003	1	17	17	175	15.3	44	43	88	43.9

1114	south-coast	2003	1	18	18	129	33.1	41	46	85	75.2
1115	south-coast	2003	1	19	19	163	38.6	44	40	77	85.7
1116	south-coast	2003	1	20	20	153	38	46	42	74	87.3
1117	south-coast	2003	1	21	21	161	42.9	43	46	69	88
1118	south-coast	2003	1	22	22	138	72.9	37	43	68	91.3
1119	south-coast	2003	1	23	23	143	69.5	30	47	76	86.6
1120	south-coast	2003	1	24	24	156	44.9	43	45	81	76.8
1121	south-coast	2003	1	25	25	150	22.5	42	46	86	56.7
1122	south-coast	2003	1	26	26	142	12.5	44	47	85	45
1123	south-coast	2003	1	27	27	132	19.5	45	45	82	63
1124	south-coast	2003	1	28	28	142	39.3	47	45	77	82.9
1125	south-coast	2003	1	29	29	160	54.1	53	44	80	87
1126	south-coast	2003	1	30	30	142	51.8	44	44	87	72.2
1127	south-coast	2003	1	31	31	133	16.1	36	52	94	50.7
1128	south-coast	2003	2	1	32	153	23.9	54	50	85	71.3
1129	south-coast	2003	2	2	33	125	20.6	47	48	72	64.5
1130	south-coast	2003	2	3	34	140	14.2	46	37	80	39.9
1131	south-coast	2003	2	4	35	145	22.7	40	38	73	59
1132	south-coast	2003	2	5	36	144	22.7	46	36	72	63
1133	south-coast	2003	2	6	37	139	17.4	45	35	72	56.7
1134	south-coast	2003	2	7	38	159	11.6	43	35	72	53.6
1135	south-coast	2003	2	8	39	171	19.3	46	38	68	63.3
1136	south-coast	2003	2	9	40	143	23.2	49	35	69	65
1137	south-coast	2003	2	10	41	149	12.9	44	38	71	39.7
1138	south-coast	2003	2	11	42	157	10.1	45	40	69	90.2
1139	south-coast	2003	2	12	43	144	6.8	43	39	68	99.5
1140	south-coast	2003	2	13	44	173	11	36	47	64	100
1141	south-coast	2003	2	14	45	151	27.4	42	52	66	95.6
1142	south-coast	2003	2	15	46	166	33.9	45	48	66	97.6
1143	south-coast	2003	2	16	47	138	27.9	44	47	68	95
1144	south-coast	2003	2	17	48	135	35	41	49	65	86.8
1145	south-coast	2003	2	18	49	126	34	45	44	70	91.3
1146	south-coast	2003	2	19	50	140	39.3	53	43	69	87.1
1147	south-coast	2003	2	20	51	158	10.4	47	44	73	78.3
1148	south-coast	2003	2	21	52	146	17.4	48	42	73	77.9
1149	south-coast	2003	2	22	53	148	21.6	57	42	74	82.5
1150	south-coast	2003	2	23	54	161	36.5	50	42	70	94
1151	south-coast	2003	2	24	55	155	10.4	46	47	67	95.2
1152	south-coast	2003	2	25	56	142	6.3	44	44	62	98.3
1153	south-coast	2003	2	26	57	143	14.5	48	44	64	98.6
1154	south-coast	2003	2	27	58	146	7.4	45	45	64	97.3
1155	south-coast	2003	2	28	59	155	10.9	45	44	66	87.4
1156	south-coast	2003	3	1	60	140	15.5	51	44	63	89
1157	south-coast	2003	3	2	61	152	12.4	52	41	71	65.7
1158	south-coast	2003	3	3	62	150	13.6	48	40	65	75.2
1159	south-coast	2003	3	4	63	169	34	45	42	60	97.1
1160	south-coast	2003	3	5	64	136	21.3	44	38	67	83.1
1161	south-coast	2003	3	6	65	137	22.4	51	40	70	77.5
1162	south-coast	2003	3	7	66	144	32.9	60	40	70	81.3

1163	south-coast	2003	3	8	67	163	53.2	63	42	73	82.3
1164	south-coast	2003	3	9	68	137	48.2	68	45	78	81.7
1165	south-coast	2003	3	10	69	145	49.3	63	44	80	77.5
1166	south-coast	2003	3	11	70	146	45.2	81	45	77	86.3
1167	south-coast	2003	3	12	71	145	56.9	78	48	78	86.3
1168	south-coast	2003	3	13	72	139	61.2	73	50	78	87.8
1169	south-coast	2003	3	14	73	147	47	48	53	72	89.3
1170	south-coast	2003	3	15	74	158	21.9	42	50	64	100
1171	south-coast	2003	3	16	75	133	7.3	49	47	66	98.5
1172	south-coast	2003	3	17	76	153	5.3	48	43	70	81.7
1173	south-coast	2003	3	18	77	126	7.6	48	42	72	61.8
1174	south-coast	2003	3	19	78	118	15.9	51	44	72	68.8
1175	south-coast	2003	3	20	79	162	22.1	55	42	72	87.8
1176	south-coast	2003	3	21	80	160	25.8	58	44	78	74.5
1177	south-coast	2003	3	22	81	147	33	66	46	78	71.5
1178	south-coast	2003	3	23	82	164	28.9	67	44	74	88.3
1179	south-coast	2003	3	24	83	125	25.8	51	46	69	98.8
1180	south-coast	2003	3	25	84	132	25.1	52	48	83	81.5
1181	south-coast	2003	3	26	85	120	23.4	58	50	85	73.9
1182	south-coast	2003	3	27	86	121	12.4	54	50	83	67.4
1183	south-coast	2003	3	28	87	123	11.3	55	48	79	64
1184	south-coast	2003	3	29	88	137	9.6	60	44	84	39.2
1185	south-coast	2003	3	30	89	135	10	70	44	91	36.5
1186	south-coast	2003	3	31	90	165	15.4	70	49	90	64
1187	south-coast	2003	4	1	91	139	14.2	69	46	83	80.2
1188	south-coast	2003	4	2	92	137	6.8	52	51	67	82.5
1189	south-coast	2003	4	3	93	138	11.8	56	40	65	79.2
1190	south-coast	2003	4	4	94	142	8.1	55	39	65	85.7
1191	south-coast	2003	4	5	95	145	8.1	56	40	68	75.2
1192	south-coast	2003	4	6	96	122	16.2	62	38	70	74
1193	south-coast	2003	4	7	97	138	13.3	58	43	81	60.8
1194	south-coast	2003	4	8	98	138	11.6	66	47	87	46.9
1195	south-coast	2003	4	9	99	136	25.8	82	49	86	61
1196	south-coast	2003	4	10	100	145	22	72	50	80	80.9
1197	south-coast	2003	4	11	101	149	28.6	54	47	74	87.9
1198	south-coast	2003	4	12	102	151	10.1	52	54	74	84.4
1199	south-coast	2003	4	13	103	136	4.4	52	51	73	91.4
1200	south-coast	2003	4	14	104	128	5.9	51	48	69	98.9
1201	south-coast	2003	4	15	105	119	10.4	55	42	70	90.8
1202	south-coast	2003	4	16	106	142	17.7	64	39	69	73.6
1203	south-coast	2003	4	17	107	128	13.2	46	40	68	92.1
1204	south-coast	2003	4	18	108	147	11.8	55	48	67	94.7
1205	south-coast	2003	4	19	109	144	14.3	64	43	75	72.5
1206	south-coast	2003	4	20	110	129	18.1	72	46	78	68.5
1207	south-coast	2003	4	21	111	148	18	60	38	69	83.1
1208	south-coast	2003	4	22	112	145	11.6	57	45	64	86.2
1209	south-coast	2003	4	23	113	133	11.3	58	46	68	87.8
1210	south-coast	2003	4	24	114	139	20.8	60	50	69	89.3
1211	south-coast	2003	4	25	115	122	12.6	50	49	71	84.4

1212	south-coast	2003	4	26	116	131	13	64	46	73	81.3
1213	south-coast	2003	4	27	117	149	16.5	70	45	73	75.5
1214	south-coast	2003	4	28	118	129	12.6	57	45	72	81.6
1215	south-coast	2003	4	29	119	134	9.1	57	44	69	76
1216	south-coast	2003	4	30	120	126	15.1	57	43	69	79.9
1217	south-coast	2003	5	1	121	123	17.7	73	44	73	75.9
1218	south-coast	2003	5	2	122	145	14.2	61	44	70	79.1
1219	south-coast	2003	5	3	123	131	7	48	48	70	95.3
1220	south-coast	2003	5	4	124	131	8.4	54	50	70	88.7
1221	south-coast	2003	5	5	125	131	17.4	60	46	70	88.5
1222	south-coast	2003	5	6	126	138	26.4	63	52	70	93.8
1223	south-coast	2003	5	7	127	122	9.9	47	51	69	95
1224	south-coast	2003	5	8	128	129	10.3	55	49	67	91.9
1225	south-coast	2003	5	9	129	120	10.9	57	48	69	76.7
1226	south-coast	2003	5	10	130	129	15.2	72	41	77	71
1227	south-coast	2003	5	11	131	120	23.1	100	45	84	70.5
1228	south-coast	2003	5	12	132	158	28.2	89	44	87	75.7
1229	south-coast	2003	5	13	133	141	32.6	71	56	75	83
1230	south-coast	2003	5	14	134	140	25.4	58	55	74	87.1
1231	south-coast	2003	5	15	135	137	28	69	49	78	77.9
1232	south-coast	2003	5	16	136	130	39.8	114	48	88	78.8
1233	south-coast	2003	5	17	137	142	45.3	105	56	84	89.1
1234	south-coast	2003	5	18	138	136	42.3	97	54	79	85
1235	south-coast	2003	5	19	139	146	45.5	99	50	91	87.1
1236	south-coast	2003	5	20	140	140	50.5	99	53	98	84.8
1237	south-coast	2003	5	21	141	139	41	108	52	101	76.8
1238	south-coast	2003	5	22	142	135	36.4	113	52	96	85.9
1239	south-coast	2003	5	23	143	140	39.8	104	57	88	86.1
1240	south-coast	2003	5	24	144	134	49.6	79	53	76	93.8
1241	south-coast	2003	5	25	145	136	29.4	81	55	76	89.4
1242	south-coast	2003	5	26	146	135	38.2	107	55	84	82.3
1243	south-coast	2003	5	27	147	131	48.5	97	55	102	84
1244	south-coast	2003	5	28	148	127	34.1	137	56	107	83.4
1245	south-coast	2003	5	29	149	135	32.3	105	55	92	91.5
1246	south-coast	2003	5	30	150	135	48.9	113	58	89	88.5
1247	south-coast	2003	5	31	151	122	40.3	93	57	90	86.3
1248	south-coast	2003	6	1	152	138	45.1	125	56	93	88.9
1249	south-coast	2003	6	2	153	130	45.1	103	57	91	92.8
1250	south-coast	2003	6	3	154	114	34.9	82	52	80	95.4
1251	south-coast	2003	6	4	155	99	24.3	87	58	81	89.9
1252	south-coast	2003	6	5	156	144	39.7	85	59	77	93.4
1253	south-coast	2003	6	6	157	137	35.9	85	58	76	90.8
1254	south-coast	2003	6	7	158	132	52.9	98	56	82	89
1255	south-coast	2003	6	8	159	134	55.1	106	56	82	88.8
1256	south-coast	2003	6	9	160	123	21.9	82	57	72	99.8
1257	south-coast	2003	6	10	161	91	12.2	52	56	76	94.3
1258	south-coast	2003	6	11	162	126	18.4	73	58	78	86.6
1259	south-coast	2003	6	12	163	113	15.9	75	55	75	84.3
1260	south-coast	2003	6	13	164	130	37.4	89	57	83	80.5

1261	south-coast	2003	6	14	165	140	48.2	126	55	93	79
1262	south-coast	2003	6	15	166	116	40.1	129	56	94	83.3
1263	south-coast	2003	6	16	167	134	39.9	114	58	92	84.2
1264	south-coast	2003	6	17	168	118	58.3	124	59	92	83.1
1265	south-coast	2003	6	18	169	113	60.5	120	58	85	92.7
1266	south-coast	2003	6	19	170	118	27.2	96	57	74	95.9
1267	south-coast	2003	6	20	171	127	14.7	48	56	66	99.1
1268	south-coast	2003	6	21	172	120	14.6	50	55	66	99.3
1269	south-coast	2003	6	22	173	124	15.9	51	54	68	98.4
1270	south-coast	2003	6	23	174	152	22	63	57	74	88.5
1271	south-coast	2003	6	24	175	150	19.1	71	56	83	80.5
1272	south-coast	2003	6	25	176	130	22.4	79	55	103	77.3
1273	south-coast	2003	6	26	177	122	30.5	96	56	104	82.2
1274	south-coast	2003	6	27	178	149	27.5	113	56	97	85.5
1275	south-coast	2003	6	28	179	129	31.8	142	58	96	90
1276	south-coast	2003	6	29	180	129	39	141	57	95	86.4
1277	south-coast	2003	6	30	181	130	32.6	118	59	100	81.1
1278	south-coast	2003	7	1	182	123	22.7	108	62	100	77.6
1279	south-coast	2003	7	2	183	116	25.6	105	63	98	76.4
1280	south-coast	2003	7	3	184	148	18.5	106	64	98	70.1
1281	south-coast	2003	7	4	185	148	69.6	119	62	100	68.6
1282	south-coast	2003	7	5	186	130	121.2	144	64	100	79
1283	south-coast	2003	7	6	187	133	19.9	120	62	94	88
1284	south-coast	2003	7	7	188	106	27.9	110	63	92	82.5
1285	south-coast	2003	7	8	189	124	37.9	127	63	94	85.5
1286	south-coast	2003	7	9	190	139	40.3	146	63	96	87.3
1287	south-coast	2003	7	10	191	116	47.2	146	65	99	83.8
1288	south-coast	2003	7	11	192	136	28.2	140	65	102	83.1
1289	south-coast	2003	7	12	193	128	23.4	117	66	101	81.7
1290	south-coast	2003	7	13	194	149	25.7	148	67	102	82.1
1291	south-coast	2003	7	14	195	137	31.9	152	66	102	85.8
1292	south-coast	2003	7	15	196	135	24.7	120	68	104	86.6
1293	south-coast	2003	7	16	197	132	20.4	104	70	104	82.1
1294	south-coast	2003	7	17	198	133	34.1	133	69	94	81
1295	south-coast	2003	7	18	199	144	25.9	74	70	94	95.3
1296	south-coast	2003	7	19	200	121	20.6	99	67	98	81.6
1297	south-coast	2003	7	20	201	106	20.8	109	65	100	82.2
1298	south-coast	2003	7	21	202	119	19.2	106	67	98	83.5
1299	south-coast	2003	7	22	203	104	21	96	67	89	91
1300	south-coast	2003	7	23	204	129	27	109	66	100	85.5
1301	south-coast	2003	7	24	205	151	31.3	119	58	94	87.5
1302	south-coast	2003	7	25	206	117	29.8	97	65	97	85.1
1303	south-coast	2003	7	26	207	104	32.4	116	65	96	84.7
1304	south-coast	2003	7	27	208	128	17.8	119	66	95	81.9
1305	south-coast	2003	7	28	209	123	17.1	83	66	94	86.8
1306	south-coast	2003	7	29	210	116	21.6	94	67	94	82.7
1307	south-coast	2003	7	30	211	126	17	69	66	95	84.3
1308	south-coast	2003	7	31	212	118	16.3	87	67	95	83
1309	south-coast	2003	8	1	213	141	14.6	75	66	91	81.5

1310	south-coast	2003	8	2	214	134	10.1	73	62	92	79.5
1311	south-coast	2003	8	3	215	127	11.7	87	60	93	79.4
1312	south-coast	2003	8	4	216	124	14.1	77	60	96	76.9
1313	south-coast	2003	8	5	217	123	13.5	58	58	97	77.2
1314	south-coast	2003	8	6	218	131	14.3	74	58	95	77.6
1315	south-coast	2003	8	7	219	121	16.2	90	63	101	74.2
1316	south-coast	2003	8	8	220	131	15.5	101	64	103	70.4
1317	south-coast	2003	8	9	221	134	15	118	68	104	69
1318	south-coast	2003	8	10	222	116	16.3	122	68	105	70.7
1319	south-coast	2003	8	11	223	132	18.1	78	68	102	67.8
1320	south-coast	2003	8	12	224	123	22.3	89	68	106	68.5
1321	south-coast	2003	8	13	225	110	22.5	88	65	103	71.5
1322	south-coast	2003	8	14	226	132	19.2	116	67	107	68.8
1323	south-coast	2003	8	15	227	153	19.9	98	69	102	68.3
1324	south-coast	2003	8	16	228	120	18.1	100	69	101	66.1
1325	south-coast	2003	8	17	229	140	21	153	60	104	69.3
1326	south-coast	2003	8	18	230	130	17.2	116	68	102	81.8
1327	south-coast	2003	8	19	231	128	18.4	79	66	100	82.6
1328	south-coast	2003	8	20	232	122	20.9	63	67	97	83.4
1329	south-coast	2003	8	21	233	128	21.4	72	67	92	81.3
1330	south-coast	2003	8	22	234	128	19.2	74	63	91	78
1331	south-coast	2003	8	23	235	147	10.5	90	60	98	78.1
1332	south-coast	2003	8	24	236	137	12	96	64	105	81.5
1333	south-coast	2003	8	25	237	127	17.9	89	65	100	85.8
1334	south-coast	2003	8	26	238	137	18.4	84	66	100	84.1
1335	south-coast	2003	8	27	239	111	21.1	86	67	98	85.7
1336	south-coast	2003	8	28	240	125	20.8	87	66	99	77.5
1337	south-coast	2003	8	29	241	123	19.9	86	64	98	78.4
1338	south-coast	2003	8	30	242	107	23.4	112	64	96	82.2
1339	south-coast	2003	8	31	243	99	29.5	135	65	98	80.4
1340	south-coast	2003	9	1	244	129	30.5	124	65	99	86.5
1341	south-coast	2003	9	2	245	116	28.2	95	65	101	88.5
1342	south-coast	2003	9	3	246	123	21.6	96	65	98	80.6
1343	south-coast	2003	9	4	247	137	19.7	90	65	104	73.5
1344	south-coast	2003	9	5	248	139	22.6	91	66	103	70.4
1345	south-coast	2003	9	6	249	134	20.4	90	65	99	66.2
1346	south-coast	2003	9	7	250	112	17.4	85	64	96	75.2
1347	south-coast	2003	9	8	251	129	16.5	58	60	92	85.5
1348	south-coast	2003	9	9	252	127	13.9	57	58	86	80
1349	south-coast	2003	9	10	253	128	19.6	66	58	85	79
1350	south-coast	2003	9	11	254	136	32.4	86	58	99	86.6
1351	south-coast	2003	9	12	255	125	38.9	100	60	95	88.9
1352	south-coast	2003	9	13	256	142	31.9	111	60	94	87.6
1353	south-coast	2003	9	14	257	140	37.9	126	62	98	89.4
1354	south-coast	2003	9	15	258	125	50.7	94	62	96	88.3
1355	south-coast	2003	9	16	259	133	41.9	82	59	93	88.2
1356	south-coast	2003	9	17	260	118	28	65	56	90	85.1
1357	south-coast	2003	9	18	261	117	27.9	78	57	96	81.6
1358	south-coast	2003	9	19	262	140	34	85	59	99	88.2

1359	south-coast	2003	9	20	263	132	40.8	123	59	99	87.5
1360	south-coast	2003	9	21	264	112	34.2	125	61	109	88.6
1361	south-coast	2003	9	22	265	121	39.4	87	63	106	89.6
1362	south-coast	2003	9	23	266	146	40.4	88	63	105	91.1
1363	south-coast	2003	9	24	267	119	46.4	67	61	84	91.4
1364	south-coast	2003	9	25	268	111	50.3	83	59	92	85.3
1365	south-coast	2003	9	26	269	122	53.1	88	58	92	91.8
1366	south-coast	2003	9	27	270	121	54	133	56	96	92.4
1367	south-coast	2003	9	28	271	123	65.4	126	58	91	96.8
1368	south-coast	2003	9	29	272	126	55.9	98	60	96	87.7
1369	south-coast	2003	9	30	273	118	56.2	66	61	94	94.8
1370	south-coast	2003	10	1	274	127	43.7	50	62	86	87.2
1371	south-coast	2003	10	2	275	118	38.2	56	56	81	90.9
1372	south-coast	2003	10	3	276	135	31.1	66	58	72	92.9
1373	south-coast	2003	10	4	277	125	42.1	71	53	80	88
1374	south-coast	2003	10	5	278	118	53.8	84	54	85	91.2
1375	south-coast	2003	10	6	279	122	80.3	78	54	87	90.9
1376	south-coast	2003	10	7	280	130	86.9	77	55	87	90
1377	south-coast	2003	10	8	281	127	79.1	93	54	93	86.4
1378	south-coast	2003	10	9	282	136	76.6	84	59	89	99.4
1379	south-coast	2003	10	10	283	106	49.3	60	57	78	91.8
1380	south-coast	2003	10	11	284	123	29.7	72	57	92	83.9
1381	south-coast	2003	10	12	285	116	30.5	95	58	93	81.2
1382	south-coast	2003	10	13	286	119	27.6	88	58	100	79.9
1383	south-coast	2003	10	14	287	138	32.9	76	59	95	89.8
1384	south-coast	2003	10	15	288	146	37.2	67	52	87	87.1
1385	south-coast	2003	10	16	289	117	47.6	71	51	94	86.5
1386	south-coast	2003	10	17	290	128	48.2	69	55	98	85
1387	south-coast	2003	10	18	291	129	23.3	68	62	102	79.3
1388	south-coast	2003	10	19	292	113	33.3	95	61	101	84.7
1389	south-coast	2003	10	20	293	121	21.8	55	61	102	73.8
1390	south-coast	2003	10	21	294	155	23.3	59	62	104	74.7
1391	south-coast	2003	10	22	295	139	25.8	66	60	100	81.3
1392	south-coast	2003	10	23	296	112	23	55	59	99	75.3
1393	south-coast	2003	10	24	297	141	98.1	57	60	99	92
1394	south-coast	2003	10	25	298	117	53.6	84	56	99	92.4
1395	south-coast	2003	10	26	299	144	115.5	104	56	93	53
1396	south-coast	2003	10	27	300	120	107.2	76	56	94	61
1397	south-coast	2003	10	28	301	154	96	63	56	90	65.8
1398	south-coast	2003	10	29	302	121	89.2	78	53	83	91.4
1399	south-coast	2003	10	30	303	125	29.8	48	56	71	93.5
1400	south-coast	2003	10	31	304	139	9.8	46	46	66	79.5
1401	south-coast	2003	11	1	305	136	10.2	46	50	68	91
1402	south-coast	2003	11	2	306	108	21.7	48	40	68	86.4
1403	south-coast	2003	11	3	307	125	15	44	46	68	91.7
1404	south-coast	2003	11	4	308	129	17.9	47	41	64	84
1405	south-coast	2003	11	5	309	137	40.6	46	39	68	78.9
1406	south-coast	2003	11	6	310	138	46.6	59	45	73	77.7
1407	south-coast	2003	11	7	311	140	36.5	46	45	74	80.8

1408	south-coast	2003	11	8	312	152	27.3	53	46	77	78.2
1409	south-coast	2003	11	9	313	134	23.8	40	48	70	92
1410	south-coast	2003	11	10	314	129	14.7	35	48	74	79.2
1411	south-coast	2003	11	11	315	132	18.7	42	49	73	78.4
1412	south-coast	2003	11	12	316	120	13.9	40	53	71	98.3
1413	south-coast	2003	11	13	317	145	15.1	46	43	71	87
1414	south-coast	2003	11	14	318	142	23.4	43	42	69	85.6
1415	south-coast	2003	11	15	319	140	15.4	43	46	66	90.9
1416	south-coast	2003	11	16	320	131	12.8	41	46	65	93.3
1417	south-coast	2003	11	17	321	151	30.8	40	44	67	93.9
1418	south-coast	2003	11	18	322	131	39.6	41	43	81	72.1
1419	south-coast	2003	11	19	323	150	18.5	41	46	84	71.9
1420	south-coast	2003	11	20	324	151	34.3	44	45	77	88.5
1421	south-coast	2003	11	21	325	125	33.1	42	47	67	92.7
1422	south-coast	2003	11	22	326	130	11.4	48	43	66	57.9
1423	south-coast	2003	11	23	327	162	22	47	34	73	56
1424	south-coast	2003	11	24	328	138	18.1	38	36	69	53.8
1425	south-coast	2003	11	25	329	135	23.9	46	40	65	79.6
1426	south-coast	2003	11	26	330	146	33.5	46	41	64	80
1427	south-coast	2003	11	27	331	151	15.2	45	45	71	44.8
1428	south-coast	2003	11	28	332	160	9.6	44	41	74	48.3
1429	south-coast	2003	11	29	333	185	28.8	45	45	77	56
1430	south-coast	2003	11	30	334	166	33.7	51	41	78	73
1431	south-coast	2003	12	1	335	145	38.8	49	41	72	78.4
1432	south-coast	2003	12	2	336	187	48.5	47	36	74	79.5
1433	south-coast	2003	12	3	337	150	36.4	50	42	80	84.6
1434	south-coast	2003	12	4	338	167	43.6	47	44	78	93
1435	south-coast	2003	12	5	339	161	66.2	43	45	79	96.5
1436	south-coast	2003	12	6	340	178	64.1	49	43	70	94.2
1437	south-coast	2003	12	7	341	157	35.3	38	53	65	100
1438	south-coast	2003	12	8	342	165	18.2	42	45	68	71
1439	south-coast	2003	12	9	343	152	18.5	41	37	72	56.4
1440	south-coast	2003	12	10	344	161	18.6	42	40	63	87.2
1441	south-coast	2003	12	11	345	167	20	41	46	62	87.9
1442	south-coast	2003	12	12	346	159	19.7	38	38	60	71.7
1443	south-coast	2003	12	13	347	153	35.4	36	37	63	74.4
1444	south-coast	2003	12	14	348	157	36.5	41	36	57	92.5
1445	south-coast	2003	12	15	349	173	16.6	40	36	65	66.2
1446	south-coast	2003	12	16	350	163	12	42	32	68	42.1
1447	south-coast	2003	12	17	351	194	24.3	45	35	72	59
1448	south-coast	2003	12	18	352	167	28.9	43	41	79	48
1449	south-coast	2003	12	19	353	194	37.3	30	44	78	55.3
1450	south-coast	2003	12	20	354	191	37.1	37	45	66	77
1451	south-coast	2003	12	21	355	170	29	41	41	65	92.8
1452	south-coast	2003	12	22	356	175	17.8	41	41	71	68.3
1453	south-coast	2003	12	23	357	164	24.5	43	44	69	68.7
1454	south-coast	2003	12	24	358	184	20.1	35	43	61	94.2
1455	south-coast	2003	12	25	359	167	8.4	37	52	63	99.8
1456	south-coast	2003	12	26	360	180	11.8	40	35	58	82.3

1457	south-coast	2003	12	27	361	167	17.1	41	31	60	64.8
1458	south-coast	2003	12	28	362	185	24.2	43	30	61	56.1
1459	south-coast	2003	12	29	363	175	35.3	44	35	61	67.3
1460	south-coast	2003	12	30	364	194	20.9	38	35	63	61.6
1461	south-coast	2003	12	31	365	192	27.1	38	36	65	64.5
1462	south-coast	2004	1	1	1	204	61	46	37	61	71.5
1463	south-coast	2004	1	2	2	212	30.7	40	42	60	94.8
1464	south-coast	2004	1	3	3	163	10.2	41	41	61	68.5
1465	south-coast	2004	1	4	4	174	18.6	40	32	63	63
1466	south-coast	2004	1	5	5	195	12.9	43	34	65	42.5
1467	south-coast	2004	1	6	6	179	16.9	47	34	65	41.5
1468	south-coast	2004	1	7	7	189	34.6	47	41	69	65
1469	south-coast	2004	1	8	8	202	41.1	40	40	79	75.5
1470	south-coast	2004	1	9	9	196	43.2	38	44	75	79.5
1471	south-coast	2004	1	10	10	208	28.6	43	42	80	68.3
1472	south-coast	2004	1	11	11	167	29.4	47	42	81	66.4
1473	south-coast	2004	1	12	12	203	49.7	47	43	77	89.8
1474	south-coast	2004	1	13	13	162	35.1	47	44	82	73
1475	south-coast	2004	1	14	14	170	16.6	41	45	78	58
1476	south-coast	2004	1	15	15	181	20.4	45	44	74	78.2
1477	south-coast	2004	1	16	16	201	47.3	40	39	66	94.8
1478	south-coast	2004	1	17	17	167	49.3	45	41	72	86.4
1479	south-coast	2004	1	18	18	153	61.8	50	38	64	88.2
1480	south-coast	2004	1	19	19	145	69.3	49	40	65	91
1481	south-coast	2004	1	20	20	158	58.4	40	41	62	97.4
1482	south-coast	2004	1	21	21	145	22.4	52	43	67	64.9
1483	south-coast	2004	1	22	22	161	38.6	49	46	72	63
1484	south-coast	2004	1	23	23	181	45.8	45	40	72	58.5
1485	south-coast	2004	1	24	24	155	18.5	46	39	65	82
1486	south-coast	2004	1	25	25	154	19.6	43	46	64	88.2
1487	south-coast	2004	1	26	26	148	25.1	43	34	64	84.1
1488	south-coast	2004	1	27	27	162	34.6	41	39	63	86.5
1489	south-coast	2004	1	28	28	153	38	44	44	68	91.3
1490	south-coast	2004	1	29	29	166	37.5	40	40	70	85.9
1491	south-coast	2004	1	30	30	146	37	49	41	67	86.1
1492	south-coast	2004	1	31	31	168	19.2	46	48	64	84.7
1493	south-coast	2004	2	1	32	153	19	52	37	65	75.8
1494	south-coast	2004	2	2	33	154	22	55	39	64	84.8
1495	south-coast	2004	2	3	34	147	9.8	40	41	60	93.1
1496	south-coast	2004	2	4	35	142	10.1	42	41	65	81.6
1497	south-coast	2004	2	5	36	157	17.4	47	36	66	63
1498	south-coast	2004	2	6	37	134	17	45	36	69	63
1499	south-coast	2004	2	7	38	135	18.3	46	39	72	49.5
1500	south-coast	2004	2	8	39	172	10.6	46	36	69	46.4
1501	south-coast	2004	2	9	40	161	14.9	46	35	68	63
1502	south-coast	2004	2	10	41	148	12.1	48	37	69	41.7
1503	south-coast	2004	2	11	42	156	19.7	48	35	73	58.6
1504	south-coast	2004	2	12	43	158	21.5	55	38	73	63
1505	south-coast	2004	2	13	44	145	14.3	51	33	69	43

1506	south-coast	2004	2	14	45	177	23.9	45	37	66	58.7
1507	south-coast	2004	2	15	46	159	35.2	52	37	66	80.8
1508	south-coast	2004	2	16	47	152	48.5	46	45	72	84
1509	south-coast	2004	2	17	48	163	46.2	63	49	83	79.1
1510	south-coast	2004	2	18	49	146	48.2	58	46	73	93.8
1511	south-coast	2004	2	19	50	173	27.1	50	41	61	90.4
1512	south-coast	2004	2	20	51	152	26.5	48	49	62	95.5
1513	south-coast	2004	2	21	52	161	27.9	52	44	59	96.4
1514	south-coast	2004	2	22	53	156	5	50	47	59	100
1515	south-coast	2004	2	23	54	146	7.2	49	43	61	97.1
1516	south-coast	2004	2	24	55	149	24.2	46	47	61	94.6
1517	south-coast	2004	2	25	56	145	14	50	43	62	87.1
1518	south-coast	2004	2	26	57	163	6.7	51	48	62	93.6
1519	south-coast	2004	2	27	58	159	16.5	47	45	61	91.6
1520	south-coast	2004	2	28	59	153	11.3	52	37	64	82.4
1521	south-coast	2004	2	29	60	153	16	53	37	62	84.7
1522	south-coast	2004	3	1	61	144	21.1	52	45	60	94.3
1523	south-coast	2004	3	2	62	161	8.7	54	42	60	91.2
1524	south-coast	2004	3	3	63	161	11.6	51	42	72	70.6
1525	south-coast	2004	3	4	64	158	15.3	46	44	66	84.7
1526	south-coast	2004	3	5	65	149	19.5	50	46	67	89.2
1527	south-coast	2004	3	6	66	139	32.2	60	45	77	81.5
1528	south-coast	2004	3	7	67	163	19	61	50	87	64
1529	south-coast	2004	3	8	68	164	11.9	50	51	92	54.8
1530	south-coast	2004	3	9	69	144	19.8	60	54	90	58.6
1531	south-coast	2004	3	10	70	170	40.1	52	54	88	88.2
1532	south-coast	2004	3	11	71	134	24.2	54	55	87	89.9
1533	south-coast	2004	3	12	72	152	39.5	59	50	82	90.6
1534	south-coast	2004	3	13	73	134	55.3	55	54	79	85.8
1535	south-coast	2004	3	14	74	124	53.8	83	49	84	92.5
1536	south-coast	2004	3	15	75	136	75	59	48	84	89.5
1537	south-coast	2004	3	16	76	141	57.3	61	53	91	92.5
1538	south-coast	2004	3	17	77	150	48.2	62	54	91	90
1539	south-coast	2004	3	18	78	151	66.3	70	52	88	90.1
1540	south-coast	2004	3	19	79	147	100.5	72	49	84	90.7
1541	south-coast	2004	3	20	80	128	73.6	74	51	94	89.3
1542	south-coast	2004	3	21	81	121	74.5	98	50	85	89.4
1543	south-coast	2004	3	22	82	129	73.6	74	54	90	92.8
1544	south-coast	2004	3	23	83	118	59.5	72	53	74	96.3
1545	south-coast	2004	3	24	84	126	20.7	58	52	69	90.3
1546	south-coast	2004	3	25	85	126	56.1	68	53	73	87.7
1547	south-coast	2004	3	26	86	118	12.3	53	54	70	88.6
1548	south-coast	2004	3	27	87	145	17.2	54	46	79	70.4
1549	south-coast	2004	3	28	88	119	15.9	75	50	90	64
1550	south-coast	2004	3	29	89	133	13.2	61	50	94	39.8
1551	south-coast	2004	3	30	90	130	21.6	68	51	88	90.5
1552	south-coast	2004	3	31	91	135	59.9	62	48	77	85.9
1553	south-coast	2004	4	1	92	147	14.3	48	54	69	99.5
1554	south-coast	2004	4	2	93	162	21	51	47	66	92.9

1555	south-coast	2004	4	3	94	139	33.3	54	52	66	98
1556	south-coast	2004	4	4	95	125	23.7	69	51	74	83.6
1557	south-coast	2004	4	5	96	148	25.9	61	50	71	88.4
1558	south-coast	2004	4	6	97	147	35	62	49	71	90.3
1559	south-coast	2004	4	7	98	126	28.9	58	53	74	85.6
1560	south-coast	2004	4	8	99	143	40.1	75	48	73	90.2
1561	south-coast	2004	4	9	100	141	90.7	67	56	78	86.8
1562	south-coast	2004	4	10	101	128	55.8	80	53	72	94.3
1563	south-coast	2004	4	11	102	140	35.7	82	50	82	82.5
1564	south-coast	2004	4	12	103	131	38.9	76	49	85	85.6
1565	south-coast	2004	4	13	104	125	32.3	72	46	80	81.3
1566	south-coast	2004	4	14	105	130	29.4	71	44	77	76.9
1567	south-coast	2004	4	15	106	124	27.9	64	47	78	80.1
1568	south-coast	2004	4	16	107	122	13.6	55	50	74	77.2
1569	south-coast	2004	4	17	108	122	12.7	49	46	69	94.3
1570	south-coast	2004	4	18	109	99	19.6	55	44	68	80.6
1571	south-coast	2004	4	19	110	121	14.2	55	44	70	78.1
1572	south-coast	2004	4	20	111	136	15.9	55	44	74	80.7
1573	south-coast	2004	4	21	112	151	40.9	54	47	73	87.5
1574	south-coast	2004	4	22	113	113	12.6	64	47	83	71.5
1575	south-coast	2004	4	23	114	156	10.1	60	49	88	50.5
1576	south-coast	2004	4	24	115	146	45.5	75	50	90	61
1577	south-coast	2004	4	25	116	133	15	90	54	97	60.6
1578	south-coast	2004	4	26	117	141	18.2	75	57	100	48.8
1579	south-coast	2004	4	27	118	144	22.5	80	60	100	61
1580	south-coast	2004	4	28	119	132	17.5	88	57	95	93.2
1581	south-coast	2004	4	29	120	126	15.1	65	55	73	82.8
1582	south-coast	2004	4	30	121	134	34.4	67	48	87	82.1
1583	south-coast	2004	5	1	122	156	18.1	88	51	96	54.1
1584	south-coast	2004	5	2	123	139	16.1	87	57	101	43.7
1585	south-coast	2004	5	3	124	128	19.3	87	61	102	62
1586	south-coast	2004	5	4	125	140	24.3	95	63	99	72
1587	south-coast	2004	5	5	126	110	20.4	73	56	92	80.7
1588	south-coast	2004	5	6	127	131	14.1	70	52	90	62
1589	south-coast	2004	5	7	128	125	15.3	62	61	91	62.8
1590	south-coast	2004	5	8	129	116	12.3	75	53	90	64
1591	south-coast	2004	5	9	130	134	16.1	102	52	89	70.1
1592	south-coast	2004	5	10	131	118	17.5	78	52	81	80.6
1593	south-coast	2004	5	11	132	124	14.9	75	56	78	80.3
1594	south-coast	2004	5	12	133	134	16.3	70	54	81	70.5
1595	south-coast	2004	5	13	134	124	17.4	87	54	89	70.3
1596	south-coast	2004	5	14	135	126	18.9	95	55	93	64.9
1597	south-coast	2004	5	15	136	111	19.4	96	54	88	73.9
1598	south-coast	2004	5	16	137	121	17.4	88	54	85	84.9
1599	south-coast	2004	5	17	138	144	17.3	63	58	78	83.3
1600	south-coast	2004	5	18	139	115	20	72	58	78	77.9
1601	south-coast	2004	5	19	140	129	13.8	75	51	79	82.3
1602	south-coast	2004	5	20	141	107	13.6	68	56	77	81.1
1603	south-coast	2004	5	21	142	137	16.3	63	56	71	82.9

1604	south-coast	2004	5	22	143	124	13.1	74	56	76	81.8
1605	south-coast	2004	5	23	144	128	11.8	70	57	74	86
1606	south-coast	2004	5	24	145	118	13.4	64	56	76	100
1607	south-coast	2004	5	25	146	124	11.2	65	56	73	75.2
1608	south-coast	2004	5	26	147	114	13.5	59	56	74	76.3
1609	south-coast	2004	5	27	148	115	25.6	80	49	81	85.2
1610	south-coast	2004	5	28	149	121	30.6	73	56	71	91.2
1611	south-coast	2004	5	29	150	137	16	72	57	80	82.5
1612	south-coast	2004	5	30	151	141	23.7	100	53	95	74.4
1613	south-coast	2004	5	31	152	147	17.4	102	56	96	70.6
1614	south-coast	2004	6	1	153	116	19.6	102	57	95	81.8
1615	south-coast	2004	6	2	154	135	28.6	122	58	93	85
1616	south-coast	2004	6	3	155	133	31.4	108	58	93	85.2
1617	south-coast	2004	6	4	156	127	32.3	119	59	97	87.3
1618	south-coast	2004	6	5	157	102	40.3	145	60	96	87.2
1619	south-coast	2004	6	6	158	105	37.8	125	59	91	86.8
1620	south-coast	2004	6	7	159	122	42.1	75	60	80	95.6
1621	south-coast	2004	6	8	160	124	26.7	70	61	77	92.5
1622	south-coast	2004	6	9	161	118	12.4	56	56	76	85.3
1623	south-coast	2004	6	10	162	114	13.3	65	56	81	85.2
1624	south-coast	2004	6	11	163	125	19.5	84	54	88	83.5
1625	south-coast	2004	6	12	164	127	23.7	108	55	88	82.2
1626	south-coast	2004	6	13	165	125	18.1	117	57	90	83.5
1627	south-coast	2004	6	14	166	109	22.7	101	58	90	86.5
1628	south-coast	2004	6	15	167	127	23.8	96	58	88	89.8
1629	south-coast	2004	6	16	168	115	25.8	73	59	83	94.1
1630	south-coast	2004	6	17	169	102	31.2	81	61	86	87.7
1631	south-coast	2004	6	18	170	130	32.8	90	59	86	85.8
1632	south-coast	2004	6	19	171	101	40.4	77	59	80	91.5
1633	south-coast	2004	6	20	172	114	47.7	105	58	86	88.2
1634	south-coast	2004	6	21	173	115	38.9	76	59	83	88.5
1635	south-coast	2004	6	22	174	131	43.6	90	59	87	89.5
1636	south-coast	2004	6	23	175	134	56.4	82	61	94	89.8
1637	south-coast	2004	6	24	176	130	40.7	96	59	95	84.8
1638	south-coast	2004	6	25	177	97	38.1	106	58	97	82.3
1639	south-coast	2004	6	26	178	134	43.9	119	59	96	83.7
1640	south-coast	2004	6	27	179	122	28.9	104	58	90	87.2
1641	south-coast	2004	6	28	180	101	19.5	67	60	85	86.1
1642	south-coast	2004	6	29	181	110	15.9	67	59	83	80.9
1643	south-coast	2004	6	30	182	115	15.2	57	59	82	77
1644	south-coast	2004	7	1	183	133	16.5	74	55	84	80.9
1645	south-coast	2004	7	2	184	118	22.8	76	54	87	80.3
1646	south-coast	2004	7	3	185	120	29.6	89	58	83	86.3
1647	south-coast	2004	7	4	186	117	46.9	110	58	90	82.5
1648	south-coast	2004	7	5	187	113	102.2	116	60	95	86.5
1649	south-coast	2004	7	6	188	114	32.2	93	60	96	82.2
1650	south-coast	2004	7	7	189	106	24.7	79	61	88	86
1651	south-coast	2004	7	8	190	125	28.6	66	59	86	84
1652	south-coast	2004	7	9	191	141	17.1	73	57	93	79.5

1653	south-coast	2004	7	10	192	120	13.8	84	58	98	78.2
1654	south-coast	2004	7	11	193	136	26.5	121	60	102	80.1
1655	south-coast	2004	7	12	194	140	23.8	88	64	102	79.1
1656	south-coast	2004	7	13	195	125	21.9	69	65	101	72
1657	south-coast	2004	7	14	196	107	21.8	72	65	102	73.6
1658	south-coast	2004	7	15	197	128	14.7	73	69	97	67.9
1659	south-coast	2004	7	16	198	110	16.8	76	64	97	64.7
1660	south-coast	2004	7	17	199	114	15.5	91	63	101	69
1661	south-coast	2004	7	18	200	113	18.8	105	66	100	71.7
1662	south-coast	2004	7	19	201	107	14.3	80	67	99	74.1
1663	south-coast	2004	7	20	202	126	23.1	90	66	100	74.1
1664	south-coast	2004	7	21	203	126	15.2	75	63	100	82.7
1665	south-coast	2004	7	22	204	116	18	81	62	96	84.9
1666	south-coast	2004	7	23	205	120	35	100	61	97	94.2
1667	south-coast	2004	7	24	206	114	31.2	116	63	101	94.9
1668	south-coast	2004	7	25	207	116	20.3	115	62	102	92.1
1669	south-coast	2004	7	26	208	129	26	88	64	103	98.5
1670	south-coast	2004	7	27	209	110	20.6	88	60	99	89.2
1671	south-coast	2004	7	28	210	113	18.9	91	59	95	88.6
1672	south-coast	2004	7	29	211	117	29.8	107	59	94	91.5
1673	south-coast	2004	7	30	212	121	24.9	96	60	92	91.1
1674	south-coast	2004	7	31	213	114	23.4	92	59	95	92.9
1675	south-coast	2004	8	1	214	134	27.8	96	62	95	91.6
1676	south-coast	2004	8	2	215	122	29.3	88	62	89	89.9
1677	south-coast	2004	8	3	216	110	28.3	88	57	93	91
1678	south-coast	2004	8	4	217	126	33.5	111	59	91	91.1
1679	south-coast	2004	8	5	218	110	21.7	99	58	94	93.6
1680	south-coast	2004	8	6	219	124	28.1	94	57	98	97.4
1681	south-coast	2004	8	7	220	138	28.4	123	57	100	99.3
1682	south-coast	2004	8	8	221	128	21.1	121	64	101	99
1683	south-coast	2004	8	9	222	122	19.4	98	65	103	92.8
1684	south-coast	2004	8	10	223	134	52.8	100	66	105	89.9
1685	south-coast	2004	8	11	224	133	21.4	104	67	103	97.8
1686	south-coast	2004	8	12	225	105	21.6	116	66	94	95.6
1687	south-coast	2004	8	13	226	127	33.5	114	66	96	95.8
1688	south-coast	2004	8	14	227	127	24.9	102	66	96	95.9
1689	south-coast	2004	8	15	228	111	17.2	87	64	94	91.8
1690	south-coast	2004	8	16	229	131	29	90	63	96	94.9
1691	south-coast	2004	8	17	230	131	31	94	63	95	96.8
1692	south-coast	2004	8	18	231	147	31.9	96	62	94	99
1693	south-coast	2004	8	19	232	115	36.8	84	62	92	91.8
1694	south-coast	2004	8	20	233	114	17.1	77	60	89	92.5
1695	south-coast	2004	8	21	234	121	22.8	92	61	85	90.9
1696	south-coast	2004	8	22	235	131	28.3	69	57	88	84.3
1697	south-coast	2004	8	23	236	104	10.5	55	56	83	91.3
1698	south-coast	2004	8	24	237	104	17.2	55	55	84	88.6
1699	south-coast	2004	8	25	238	115	82.3	72	58	90	92.9
1700	south-coast	2004	8	26	239	119	19.3	73	59	88	92.6
1701	south-coast	2004	8	27	240	133	18.7	74	58	91	91.3

1702	south-coast	2004	8	28	241	106	28.3	115	60	97	93
1703	south-coast	2004	8	29	242	120	27.9	111	61	97	90.3
1704	south-coast	2004	8	30	243	132	27.8	106	58	101	94.5
1705	south-coast	2004	8	31	244	118	33.6	108	61	105	97.5
1706	south-coast	2004	9	1	245	138	29.6	113	62	105	93.6
1707	south-coast	2004	9	2	246	135	24.1	98	64	101	93.5
1708	south-coast	2004	9	3	247	114	25.8	72	60	90	82.7
1709	south-coast	2004	9	4	248	114	18.1	83	55	98	89.8
1710	south-coast	2004	9	5	249	135	13	99	55	103	50.9
1711	south-coast	2004	9	6	250	121	21.4	104	59	101	74.1
1712	south-coast	2004	9	7	251	133	20.8	97	59	105	81.5
1713	south-coast	2004	9	8	252	135	22.4	96	66	104	88.3
1714	south-coast	2004	9	9	253	106	30.2	81	69	98	94.7
1715	south-coast	2004	9	10	254	116	20.8	102	69	100	93.5
1716	south-coast	2004	9	11	255	127	20.4	90	69	100	92.5
1717	south-coast	2004	9	12	256	97	23.8	78	60	97	97.4
1718	south-coast	2004	9	13	257	109	17.1	72	59	88	87.3
1719	south-coast	2004	9	14	258	108	14.4	67	63	82	88
1720	south-coast	2004	9	15	259	122	36.1	82	61	86	93.7
1721	south-coast	2004	9	16	260	122	47	102	66	91	88.8
1722	south-coast	2004	9	17	261	121	39.9	95	60	90	91.5
1723	south-coast	2004	9	18	262	129	35.5	82	57	89	90.7
1724	south-coast	2004	9	19	263	98	8.1	60	60	78	83.9
1725	south-coast	2004	9	20	264	133	9	64	53	77	73.7
1726	south-coast	2004	9	21	265	101	14.5	57	55	89	63
1727	south-coast	2004	9	22	266	155	9.9	59	53	94	45
1728	south-coast	2004	9	23	267	114	13.8	75	57	96	62.3
1729	south-coast	2004	9	24	268	141	31.9	71	57	98	85.7
1730	south-coast	2004	9	25	269	114	30.4	93	57	100	97.8
1731	south-coast	2004	9	26	270	130	38	107	58	99	77.4
1732	south-coast	2004	9	27	271	124	67.4	80	57	94	98.3
1733	south-coast	2004	9	28	272	104	31.8	65	51	85	86.3
1734	south-coast	2004	9	29	273	117	12.3	63	55	76	86.6
1735	south-coast	2004	9	30	274	127	15.9	62	55	73	93.5
1736	south-coast	2004	10	1	275	133	26.1	66	59	81	90.8
1737	south-coast	2004	10	2	276	129	32.1	68	52	88	93.4
1738	south-coast	2004	10	3	277	128	44.5	88	53	87	96.3
1739	south-coast	2004	10	4	278	124	45.9	73	55	85	96.6
1740	south-coast	2004	10	5	279	134	42.4	65	53	88	93.7
1741	south-coast	2004	10	6	280	116	60.5	69	55	96	98
1742	south-coast	2004	10	7	281	121	66.7	89	58	93	96.5
1743	south-coast	2004	10	8	282	138	62.5	86	57	100	71.4
1744	south-coast	2004	10	9	283	130	90.2	85	55	89	98.5
1745	south-coast	2004	10	10	284	128	13	64	59	76	87.8
1746	south-coast	2004	10	11	285	120	21.6	60	50	84	91.2
1747	south-coast	2004	10	12	286	123	24.3	58	59	91	98
1748	south-coast	2004	10	13	287	121	35.2	59	57	91	95.9
1749	south-coast	2004	10	14	288	118	22.5	60	52	95	77.7
1750	south-coast	2004	10	15	289	141	42.5	70	50	90	95.8

1751	south-coast	2004	10	16	290	123	39.2	67	57	73	92.5
1752	south-coast	2004	10	17	291	120	9.4	51	56	72	97.4
1753	south-coast	2004	10	18	292	110	12.4	47	56	72	97.8
1754	south-coast	2004	10	19	293	115	7.4	49	54	72	99.6
1755	south-coast	2004	10	20	294	135	5.4	52	56	72	100
1756	south-coast	2004	10	21	295	130	14.1	52	49	72	93
1757	south-coast	2004	10	22	296	133	11.4	60	41	70	82.1
1758	south-coast	2004	10	23	297	146	26.2	64	45	73	85.5
1759	south-coast	2004	10	24	298	110	40.2	76	47	70	92.1
1760	south-coast	2004	10	25	299	127	20.1	55	54	70	91
1761	south-coast	2004	10	26	300	128	7.6	58	54	67	91.2
1762	south-coast	2004	10	27	301	121	8	57	46	67	99.9
1763	south-coast	2004	10	28	302	127	7.8	53	47	67	93.2
1764	south-coast	2004	10	29	303	105	15.1	50	44	69	84.1
1765	south-coast	2004	10	30	304	118	24.3	54	42	73	80.4
1766	south-coast	2004	10	31	305	124	28.7	63	43	76	83.1
1767	south-coast	2004	11	1	306	120	13.6	53	49	74	50.6
1768	south-coast	2004	11	2	307	137	16	43	40	77	58.8
1769	south-coast	2004	11	3	308	125	18.4	56	42	76	73.4
1770	south-coast	2004	11	4	309	124	10.1	59	47	69	88.8
1771	south-coast	2004	11	5	310	118	20.7	56	39	69	80.9
1772	south-coast	2004	11	6	311	143	17.4	58	43	70	85.5
1773	south-coast	2004	11	7	312	119	16.7	52	43	66	91.1
1774	south-coast	2004	11	8	313	125	23	58	50	65	97.2
1775	south-coast	2004	11	9	314	116	13	52	49	65	96.5
1776	south-coast	2004	11	10	315	125	32.9	49	42	68	88.3
1777	south-coast	2004	11	11	316	125	33.5	46	46	66	93.9
1778	south-coast	2004	11	12	317	127	23.4	43	47	72	88.2
1779	south-coast	2004	11	13	318	142	24.3	48	46	69	91.8
1780	south-coast	2004	11	14	319	155	20.9	51	45	78	70.8
1781	south-coast	2004	11	15	320	110	14.8	46	45	76	68
1782	south-coast	2004	11	16	321	138	20.6	44	47	73	69.3
1783	south-coast	2004	11	17	322	128	37.7	44	48	76	77.7
1784	south-coast	2004	11	18	323	131	36.2	45	46	75	78.6
1785	south-coast	2004	11	19	324	117	52	52	45	73	91.7
1786	south-coast	2004	11	20	325	140	40.8	55	45	66	86.5
1787	south-coast	2004	11	21	326	103	7.3	47	38	61	97
1788	south-coast	2004	11	22	327	135	13.6	45	35	64	90
1789	south-coast	2004	11	23	328	112	30.5	44	36	61	87.3
1790	south-coast	2004	11	24	329	120	32.3	43	39	70	82.2
1791	south-coast	2004	11	25	330	131	43.5	47	40	69	83.5
1792	south-coast	2004	11	26	331	114	50.4	58	42	65	88.6
1793	south-coast	2004	11	27	332	151	39	48	43	64	98.8
1794	south-coast	2004	11	28	333	110	7.1	47	43	63	59
1795	south-coast	2004	11	29	334	120	16.8	44	38	62	56
1796	south-coast	2004	11	30	335	143	20.1	40	29	62	49.5
1797	south-coast	2004	12	1	336	156	27.5	39	30	62	49.8
1798	south-coast	2004	12	2	337	148	22.8	43	31	64	59
1799	south-coast	2004	12	3	338	140	19.6	46	32	65	53

1800	south-coast	2004	12	4	339	150	29.5	50	32	64	72.2
1801	south-coast	2004	12	5	340	163	20.3	51	43	64	99.1
1802	south-coast	2004	12	6	341	160	28.1	49	36	58	95.2
1803	south-coast	2004	12	7	342	135	20.8	48	41	58	95.8
1804	south-coast	2004	12	8	343	140	18.3	43	46	63	99.8
1805	south-coast	2004	12	9	344	127	33.4	41	48	73	93.4
1806	south-coast	2004	12	10	345	138	19.9	34	45	82	80.9
1807	south-coast	2004	12	11	346	132	23.2	35	46	83	77.4
1808	south-coast	2004	12	12	347	175	33.4	45	47	78	97.8
1809	south-coast	2004	12	13	348	145	46.5	43	44	77	95.9
1810	south-coast	2004	12	14	349	136	52.9	43	45	79	77.8
1811	south-coast	2004	12	15	350	140	30.9	44	44	79	72
1812	south-coast	2004	12	16	351	130	19.8	47	43	75	48.8
1813	south-coast	2004	12	17	352	156	26	45	40	74	59
1814	south-coast	2004	12	18	353	151	20.9	46	39	79	38.3
1815	south-coast	2004	12	19	354	170	11	46	41	79	60.8
1816	south-coast	2004	12	20	355	130	23.3	43	41	76	63.7
1817	south-coast	2004	12	21	356	134	26.2	41	42	70	90.3
1818	south-coast	2004	12	22	357	117	30.9	47	40	63	83.3
1819	south-coast	2004	12	23	358	156	20.9	50	37	66	59
1820	south-coast	2004	12	24	359	141	23.3	45	37	66	59.9
1821	south-coast	2004	12	25	360	128	41.7	50	34	68	66
1822	south-coast	2004	12	26	361	161	45.4	51	40	60	89.8
1823	south-coast	2004	12	27	362	144	23.9	51	45	62	84.6
1824	south-coast	2004	12	28	363	145	6.4	48	50	62	98.2
1825	south-coast	2004	12	29	364	171	8.4	49	47	60	100
1826	south-coast	2004	12	30	365	156	12.1	45	41	60	94.3
1827	south-coast	2004	12	31	366	163	16.5	45	43	59	99
1828	south-coast	2005	1	1	1	134	42.2	45	37	57	91.5
1829	south-coast	2005	1	2	2	143	20	44	37	57	96.2
1830	south-coast	2005	1	3	3	133	9.1	45	41	56	99.8
1831	south-coast	2005	1	4	4	155	18	44	37	56	99.4
1832	south-coast	2005	1	5	5	133	13.8	41	36	57	88.6
1833	south-coast	2005	1	6	6	145	22.6	41	34	57	90.2
1834	south-coast	2005	1	7	7	149	16.4	44	39	55	100
1835	south-coast	2005	1	8	8	157	6	45	48	59	100
1836	south-coast	2005	1	9	9	174	4.5	41	52	60	100
1837	south-coast	2005	1	10	10	169	6.6	43	53	60	100
1838	south-coast	2005	1	11	11	165	4.6	53	49	59	92.8
1839	south-coast	2005	1	12	12	159	13.8	44	39	63	83.9
1840	south-coast	2005	1	13	13	143	29.5	32	36	64	80.5
1841	south-coast	2005	1	14	14	133	26.5	39	40	71	73.5
1842	south-coast	2005	1	15	15	135	25.9	34	39	75	68
1843	south-coast	2005	1	16	16	140	18	41	41	80	69.1
1844	south-coast	2005	1	17	17	149	16.2	41	45	82	51.4
1845	south-coast	2005	1	18	18	163	14.6	38	46	80	57.6
1846	south-coast	2005	1	19	19	146	13.8	43	46	82	57.2
1847	south-coast	2005	1	20	20	188	16.9	38	47	81	66
1848	south-coast	2005	1	21	21	161	29.4	44	47	72	77.3

1849	south-coast	2005	1	22	22	156	64	45	44	77	91
1850	south-coast	2005	1	23	23	117	37.8	48	45	78	95
1851	south-coast	2005	1	24	24	147	43	53	46	76	91
1852	south-coast	2005	1	25	25	145	34.7	38	48	70	95.7
1853	south-coast	2005	1	26	26	132	19.3	39	51	66	99.8
1854	south-coast	2005	1	27	27	126	12.6	44	49	61	95.5
1855	south-coast	2005	1	28	28	151	13.8	41	41	59	96.9
1856	south-coast	2005	1	29	29	135	9.5	43	40	63	86.7
1857	south-coast	2005	1	30	30	141	14.7	46	38	65	76.5
1858	south-coast	2005	1	31	31	154	17	45	40	74	62.8
1859	south-coast	2005	2	1	32	139	13.9	49	41	72	61.2
1860	south-coast	2005	2	2	33	180	12.3	50	41	72	52.1
1861	south-coast	2005	2	3	34	152	15.6	52	46	72	47.8
1862	south-coast	2005	2	4	35	159	13.8	48	41	74	52.3
1863	south-coast	2005	2	5	36	173	20.1	38	41	74	66.8
1864	south-coast	2005	2	6	37	155	18	50	44	67	99.4
1865	south-coast	2005	2	7	38	155	16	46	46	59	99.2
1866	south-coast	2005	2	8	39	141	18.6	45	43	69	84.8
1867	south-coast	2005	2	9	40	141	22.9	50	39	70	73.5
1868	south-coast	2005	2	10	41	155	10.3	45	46	69	72.8
1869	south-coast	2005	2	11	42	148	5.1	45	49	65	100
1870	south-coast	2005	2	12	43	142	8.8	46	49	64	99.3
1871	south-coast	2005	2	13	44	168	27.9	46	52	64	98
1872	south-coast	2005	2	14	45	162	19.6	42	50	64	95.2
1873	south-coast	2005	2	15	46	153	22	37	43	62	92
1874	south-coast	2005	2	16	47	165	18.2	36	41	69	81.6
1875	south-coast	2005	2	17	48	134	12.4	36	45	72	83.6
1876	south-coast	2005	2	18	49	142	8.5	48	49	60	99.7
1877	south-coast	2005	2	19	50	171	4	51	48	62	99.5
1878	south-coast	2005	2	20	51	161	4	47	47	62	94.9
1879	south-coast	2005	2	21	52	138	10.3	43	42	60	99.8
1880	south-coast	2005	2	22	53	146	4.3	48	47	60	94.1
1881	south-coast	2005	2	23	54	137	5.3	51	46	62	97.4
1882	south-coast	2005	2	24	55	161	29.6	48	41	66	93.2
1883	south-coast	2005	2	25	56	144	33.8	45	41	65	93.3
1884	south-coast	2005	2	26	57	156	35.2	51	41	66	95.5
1885	south-coast	2005	2	27	58	158	24.7	55	45	66	94
1886	south-coast	2005	2	28	59	148	20.4	47	45	64	93.7
1887	south-coast	2005	3	1	60	166	20.4	45	45	67	94.1
1888	south-coast	2005	3	2	61	132	19.7	42	48	64	94
1889	south-coast	2005	3	3	62	137	12.1	46	51	65	92.8
1890	south-coast	2005	3	4	63	159	6.8	48	50	62	98.1
1891	south-coast	2005	3	5	64	153	12.9	49	46	66	95.6
1892	south-coast	2005	3	6	65	132	21.1	50	46	76	93.9
1893	south-coast	2005	3	7	66	129	34.4	53	45	79	97.8
1894	south-coast	2005	3	8	67	139	54.7	61	46	73	96.8
1895	south-coast	2005	3	9	68	132	63.1	53	45	81	97.7
1896	south-coast	2005	3	10	69	152	73.7	66	49	86	90.4
1897	south-coast	2005	3	11	70	143	78	63	52	90	92

1898	south-coast	2005	3	12	71	138	46.9	75	49	73	94.8
1899	south-coast	2005	3	13	72	126	27.2	53	52	64	100
1900	south-coast	2005	3	14	73	148	14.7	51	51	74	87.5
1901	south-coast	2005	3	15	74	127	11.4	52	41	73	52.8
1902	south-coast	2005	3	16	75	156	13.1	54	42	74	56.2
1903	south-coast	2005	3	17	76	126	24	60	44	69	93.3
1904	south-coast	2005	3	18	77	154	15.4	53	52	61	93.3
1905	south-coast	2005	3	19	78	150	11.7	45	45	65	100
1906	south-coast	2005	3	20	79	137	11.2	52	46	63	92.5
1907	south-coast	2005	3	21	80	144	10.9	49	48	70	88.4
1908	south-coast	2005	3	22	81	147	9.1	46	46	65	94.2
1909	south-coast	2005	3	23	82	141	6.3	50	45	62	95.6
1910	south-coast	2005	3	24	83	139	8.3	49	45	61	95.6
1911	south-coast	2005	3	25	84	142	14.9	53	43	66	87.9
1912	south-coast	2005	3	26	85	125	16.5	53	41	74	82.4
1913	south-coast	2005	3	27	86	154	15.4	66	45	75	78.1
1914	south-coast	2005	3	28	87	124	15.7	50	47	69	84
1915	south-coast	2005	3	29	88	129	12.6	53	41	69	77.3
1916	south-coast	2005	3	30	89	134	9.5	58	42	75	63.3
1917	south-coast	2005	3	31	90	149	9.6	62	45	79	41.3
1918	south-coast	2005	4	1	91	152	10	63	41	82	41.4
1919	south-coast	2005	4	2	92	142	14	75	45	83	54.6
1920	south-coast	2005	4	3	93	131	21.6	76	44	76	93.4
1921	south-coast	2005	4	4	94	141	11.9	56	49	69	85.7
1922	south-coast	2005	4	5	95	131	12.8	64	41	85	77.9
1923	south-coast	2005	4	6	96	147	17.2	74	48	87	68.1
1924	south-coast	2005	4	7	97	129	17.8	77	47	85	94.6
1925	south-coast	2005	4	8	98	159	8.3	54	43	68	82.7
1926	south-coast	2005	4	9	99	138	6.9	55	41	68	77.6
1927	south-coast	2005	4	10	100	133	11	59	44	78	79.7
1928	south-coast	2005	4	11	101	133	14.9	65	44	80	88.2
1929	south-coast	2005	4	12	102	136	18.9	69	46	83	82.8
1930	south-coast	2005	4	13	103	131	29.7	71	46	79	88.5
1931	south-coast	2005	4	14	104	156	20.6	70	43	83	67.1
1932	south-coast	2005	4	15	105	149	28.8	77	47	87	83.3
1933	south-coast	2005	4	16	106	152	38.5	91	50	88	79.4
1934	south-coast	2005	4	17	107	132	37.2	102	49	83	98.1
1935	south-coast	2005	4	18	108	137	30.1	76	53	73	91.5
1936	south-coast	2005	4	19	109	120	17.2	61	50	67	88.8
1937	south-coast	2005	4	20	110	135	18.6	59	45	73	88
1938	south-coast	2005	4	21	111	140	18.1	79	44	85	87.5
1939	south-coast	2005	4	22	112	145	25.8	64	54	78	90.8
1940	south-coast	2005	4	23	113	145	13.2	54	43	74	87.2
1941	south-coast	2005	4	24	114	144	5.6	47	50	66	97.5
1942	south-coast	2005	4	25	115	145	17.7	51	50	70	92.1
1943	south-coast	2005	4	26	116	154	31.9	63	46	77	93
1944	south-coast	2005	4	27	117	135	12.2	59	47	70	90
1945	south-coast	2005	4	28	118	112	5.6	50	50	66	91.7
1946	south-coast	2005	4	29	119	128	8.5	54	48	71	89.2

1947	south-coast	2005	4	30	120	133	16	69	46	77	90.9
1948	south-coast	2005	5	1	121	132	24.5	74	49	74	91.6
1949	south-coast	2005	5	2	122	141	15.2	54	55	74	90.8
1950	south-coast	2005	5	3	123	123	22.1	63	54	79	90.2
1951	south-coast	2005	5	4	124	145	37	73	51	80	94.9
1952	south-coast	2005	5	5	125	129	10	52	55	69	96.1
1953	south-coast	2005	5	6	126	130	4.9	51	49	66	94.5
1954	south-coast	2005	5	7	127	146	9.5	50	47	70	89.4
1955	south-coast	2005	5	8	128	109	12.2	64	48	73	91.7
1956	south-coast	2005	5	9	129	142	11	49	49	68	92.7
1957	south-coast	2005	5	10	130	154	10.8	55	46	71	76.2
1958	south-coast	2005	5	11	131	118	10.9	59	44	79	78
1959	south-coast	2005	5	12	132	140	14.5	75	49	87	77.3
1960	south-coast	2005	5	13	133	124	18.7	83	53	90	83.3
1961	south-coast	2005	5	14	134	152	18.4	106	54	91	90.5
1962	south-coast	2005	5	15	135	137	21.2	125	56	92	94
1963	south-coast	2005	5	16	136	147	27.4	77	56	84	94.7
1964	south-coast	2005	5	17	137	137	14.8	61	57	76	93.5
1965	south-coast	2005	5	18	138	117	23.8	79	52	82	85.4
1966	south-coast	2005	5	19	139	161	34.1	95	54	91	90.1
1967	south-coast	2005	5	20	140	121	15.4	95	59	94	91.1
1968	south-coast	2005	5	21	141	144	18.3	111	58	99	86.8
1969	south-coast	2005	5	22	142	122	28.2	145	59	99	93
1970	south-coast	2005	5	23	143	139	33.2	109	58	91	96
1971	south-coast	2005	5	24	144	137	29.3	92	56	90	90.6
1972	south-coast	2005	5	25	145	135	40.5	109	54	91	96.8
1973	south-coast	2005	5	26	146	139	40.5	98	56	89	87.4
1974	south-coast	2005	5	27	147	120	38.6	102	54	86	95.8
1975	south-coast	2005	5	28	148	142	36.6	93	57	79	96.5
1976	south-coast	2005	5	29	149	122	22.2	91	59	84	94.3
1977	south-coast	2005	5	30	150	128	12.8	80	54	82	89.2
1978	south-coast	2005	5	31	151	115	28.7	83	54	86	92.9
1979	south-coast	2005	6	1	152	130	32.5	87	51	81	96.5
1980	south-coast	2005	6	2	153	117	10.3	56	57	75	96.3
1981	south-coast	2005	6	3	154	125	17.6	65	58	80	98.1
1982	south-coast	2005	6	4	155	119	27.6	91	56	81	97.2
1983	south-coast	2005	6	5	156	107	16.7	81	56	76	92.6
1984	south-coast	2005	6	6	157	128	21.7	73	57	77	87.7
1985	south-coast	2005	6	7	158	129	17.3	76	54	80	89.2
1986	south-coast	2005	6	8	159	124	18.3	82	51	82	84.6
1987	south-coast	2005	6	9	160	124	17.6	54	52	75	92.4
1988	south-coast	2005	6	10	161	141	25.4	64	59	79	95.1
1989	south-coast	2005	6	11	162	118	32.9	73	58	76	96.8
1990	south-coast	2005	6	12	163	119	27.6	92	59	82	90.9
1991	south-coast	2005	6	13	164	127	30.9	106	57	95	83.8
1992	south-coast	2005	6	14	165	129	37	105	59	89	98.7
1993	south-coast	2005	6	15	166	105	42.7	88	61	85	96.3
1994	south-coast	2005	6	16	167	120	11.8	72	61	80	96
1995	south-coast	2005	6	17	168	139	7.1	46	57	78	93.6

1996	south-coast	2005	6	18	169	137	9.7	63	54	81	84.3
1997	south-coast	2005	6	19	170	125	8.4	86	51	89	87.3
1998	south-coast	2005	6	20	171	130	10.2	80	54	94	88.8
1999	south-coast	2005	6	21	172	145	16.7	89	57	98	85.8
2000	south-coast	2005	6	22	173	120	14.3	84	60	98	88.4
2001	south-coast	2005	6	23	174	126	17.5	79	56	93	96.8
2002	south-coast	2005	6	24	175	125	22.7	76	55	93	92.6
2003	south-coast	2005	6	25	176	140	12	73	52	92	89.4
2004	south-coast	2005	6	26	177	109	11.9	88	53	87	88
2005	south-coast	2005	6	27	178	118	21.6	75	52	89	90.5
2006	south-coast	2005	6	28	179	111	22.2	79	55	86	89.6
2007	south-coast	2005	6	29	180	110	26.7	115	55	90	98.8
2008	south-coast	2005	6	30	181	127	31.6	108	57	93	86.1
2009	south-coast	2005	7	1	182	120	34.3	108	57	85	98.3
2010	south-coast	2005	7	2	183	136	28.1	94	60	86	99
2011	south-coast	2005	7	3	184	141	33.6	97	58	83	98
2012	south-coast	2005	7	4	185	146	59.3	99	59	86	96.3
2013	south-coast	2005	7	5	186	107	132.6	99	58	84	98.6
2014	south-coast	2005	7	6	187	111	40.5	90	59	83	96
2015	south-coast	2005	7	7	188	128	21	85	59	84	96.2
2016	south-coast	2005	7	8	189	126	19.3	79	60	85	96.4
2017	south-coast	2005	7	9	190	121	33.6	104	62	83	94.8
2018	south-coast	2005	7	10	191	123	24.6	107	62	83	97
2019	south-coast	2005	7	11	192	120	28.2	103	62	86	87.3
2020	south-coast	2005	7	12	193	126	35	124	62	87	88.5
2021	south-coast	2005	7	13	194	114	20.6	109	63	95	79.5
2022	south-coast	2005	7	14	195	115	23.2	77	65	94	83.6
2023	south-coast	2005	7	15	196	127	31.2	142	62	88	88.8
2024	south-coast	2005	7	16	197	119	29.6	141	63	88	88.5
2025	south-coast	2005	7	17	198	132	25.1	141	63	90	85.9
2026	south-coast	2005	7	18	199	150	32.9	127	64	88	88
2027	south-coast	2005	7	19	200	128	20.8	109	64	92	98.9
2028	south-coast	2005	7	20	201	133	17.4	81	66	96	95.3
2029	south-coast	2005	7	21	202	155	20.8	109	68	96	95.9
2030	south-coast	2005	7	22	203	144	18	119	67	97	94.9
2031	south-coast	2005	7	23	204	130	16	85	67	95	97.3
2032	south-coast	2005	7	24	205	127	17.6	91	66	89	98.8
2033	south-coast	2005	7	25	206	133	13.9	112	67	89	99
2034	south-coast	2005	7	26	207	151	18.1	119	64	89	99
2035	south-coast	2005	7	27	208	108	22.6	118	67	89	99
2036	south-coast	2005	7	28	209	111	16.4	102	66	89	99
2037	south-coast	2005	7	29	210	130	17.4	87	64	89	99
2038	south-coast	2005	7	30	211	122	23.4	102	66	91	98.9
2039	south-coast	2005	7	31	212	111	15.2	102	68	91	99
2040	south-coast	2005	8	1	213	118	20.8	88	64	98	85.8
2041	south-coast	2005	8	2	214	100	28.8	94	62	97	89.5
2042	south-coast	2005	8	3	215	112	24.1	87	63	97	84.5
2043	south-coast	2005	8	4	216	136	29.7	107	64	97	84.5
2044	south-coast	2005	8	5	217	129	46.6	109	67	99	85.5

2045	south-coast	2005	8	6	218	120	22	118	67	102	84
2046	south-coast	2005	8	7	219	115	22.8	114	65	97	85.7
2047	south-coast	2005	8	8	220	143	25.4	84	64	94	78.9
2048	south-coast	2005	8	9	221	135	18.5	108	64	97	83.2
2049	south-coast	2005	8	10	222	126	22.6	79	66	93	87
2050	south-coast	2005	8	11	223	139	27.2	98	64	94	86.3
2051	south-coast	2005	8	12	224	122	24.4	98	63	90	90
2052	south-coast	2005	8	13	225	143	29.9	98	61	86	88.6
2053	south-coast	2005	8	14	226	130	34.8	87	62	83	87
2054	south-coast	2005	8	15	227	111	30.9	68	61	82	88.3
2055	south-coast	2005	8	16	228	126	36.3	77	60	88	83.5
2056	south-coast	2005	8	17	229	113	32.4	91	58	89	85
2057	south-coast	2005	8	18	230	127	30.5	66	58	90	81.5
2058	south-coast	2005	8	19	231	124	24.2	72	56	92	83.1
2059	south-coast	2005	8	20	232	124	24.7	92	60	98	83.7
2060	south-coast	2005	8	21	233	125	21	106	61	99	84
2061	south-coast	2005	8	22	234	130	20.4	86	61	104	83.9
2062	south-coast	2005	8	23	235	124	30.4	86	60	95	88.3
2063	south-coast	2005	8	24	236	116	26.9	65	59	98	80.5
2064	south-coast	2005	8	25	237	143	19.1	82	64	101	60.4
2065	south-coast	2005	8	26	238	127	23.4	79	67	103	56.8
2066	south-coast	2005	8	27	239	145	20	130	67	103	69.7
2067	south-coast	2005	8	28	240	113	18.3	121	66	105	69.5
2068	south-coast	2005	8	29	241	136	28.5	108	65	102	76.2
2069	south-coast	2005	8	30	242	125	18.1	94	64	101	88.6
2070	south-coast	2005	8	31	243	130	16.9	80	58	99	83.9
2071	south-coast	2005	9	1	244	120	29.5	90	55	94	97.1
2072	south-coast	2005	9	2	245	103	38.4	92	58	98	98.9
2073	south-coast	2005	9	3	246	147	28.7	97	57	97	99
2074	south-coast	2005	9	4	247	106	25.3	96	55	97	98.9
2075	south-coast	2005	9	5	248	121	17.6	96	56	97	93.8
2076	south-coast	2005	9	6	249	132	16.1	71	57	97	98.6
2077	south-coast	2005	9	7	250	128	28.5	72	57	98	98.7
2078	south-coast	2005	9	8	251	111	19.3	70	57	90	97.6
2079	south-coast	2005	9	9	252	135	18.1	50	54	83	96
2080	south-coast	2005	9	10	253	143	17	59	59	79	97.4
2081	south-coast	2005	9	11	254	120	13.1	63	59	79	96.8
2082	south-coast	2005	9	12	255	132	16	59	54	80	96.1
2083	south-coast	2005	9	13	256	131	23.7	62	53	77	95.7
2084	south-coast	2005	9	14	257	115	19.1	66	57	80	93.5
2085	south-coast	2005	9	15	258	132	28.8	91	52	85	97.3
2086	south-coast	2005	9	16	259	138	39.4	76	58	79	98.4
2087	south-coast	2005	9	17	260	130	42.7	105	52	85	98.8
2088	south-coast	2005	9	18	261	128	24.2	80	51	89	97
2089	south-coast	2005	9	19	262	133	34.8	66	51	91	97.8
2090	south-coast	2005	9	20	263	142	28.4	53	63	81	99
2091	south-coast	2005	9	21	264	126	30.2	74	58	93	98.9
2092	south-coast	2005	9	22	265	117	33	73	55	93	98.5
2093	south-coast	2005	9	23	266	133	37.9	79	58	91	98.9

2094	south-coast	2005	9	24	267	127	29.9	66	56	81	98.4
2095	south-coast	2005	9	25	268	129	31.8	77	53	90	98.3
2096	south-coast	2005	9	26	269	136	22.6	65	47	95	94.8
2097	south-coast	2005	9	27	270	116	14.6	61	59	90	95.9
2098	south-coast	2005	9	28	271	155	17.4	66	56	99	69.3
2099	south-coast	2005	9	29	272	157	18.3	61	61	100	49.6
2100	south-coast	2005	9	30	273	146	28.1	85	60	98	81.8
2101	south-coast	2005	10	1	274	138	37.5	73	61	93	99
2102	south-coast	2005	10	2	275	134	38.7	84	54	87	98.9
2103	south-coast	2005	10	3	276	114	35.6	60	54	82	97.8
2104	south-coast	2005	10	4	277	109	16.9	55	49	89	78.6
2105	south-coast	2005	10	5	278	145	11	52	51	92	29.4
2106	south-coast	2005	10	6	279	133	13.5	72	53	95	39
2107	south-coast	2005	10	7	280	126	27.4	77	54	93	88.8
2108	south-coast	2005	10	8	281	141	18.4	62	51	85	96.1
2109	south-coast	2005	10	9	282	123	20.6	69	52	80	97.1
2110	south-coast	2005	10	10	283	110	21.3	59	52	86	98
2111	south-coast	2005	10	11	284	120	27.7	67	51	84	99
2112	south-coast	2005	10	12	285	115	36.4	71	50	86	99
2113	south-coast	2005	10	13	286	140	30.5	55	53	99	94.7
2114	south-coast	2005	10	14	287	132	19.9	64	57	97	65
2115	south-coast	2005	10	15	288	129	15.6	68	54	91	97.5
2116	south-coast	2005	10	16	289	121	9.4	51	49	72	86.2
2117	south-coast	2005	10	17	290	121	16.5	36	57	73	100
2118	south-coast	2005	10	18	291	142	12	41	53	64	98.8
2119	south-coast	2005	10	19	292	112	35.5	52	52	74	98.9
2120	south-coast	2005	10	20	293	145	47	64	49	80	99
2121	south-coast	2005	10	21	294	128	82.2	59	49	78	99
2122	south-coast	2005	10	22	295	137	112.6	56	57	70	99
2123	south-coast	2005	10	23	296	129	95.9	61	57	74	99
2124	south-coast	2005	10	24	297	104	58.4	54	58	69	99
2125	south-coast	2005	10	25	298	133	18.7	38	53	67	98.3
2126	south-coast	2005	10	26	299	138	23.6	46	53	71	98.8
2127	south-coast	2005	10	27	300	138	20	42	54	68	98.9
2128	south-coast	2005	10	28	301	122	15.6	44	54	71	97.7
2129	south-coast	2005	10	29	302	143	23.4	52	47	74	97.3
2130	south-coast	2005	10	30	303	121	28.9	59	47	80	97.5
2131	south-coast	2005	10	31	304	133	22	48	49	89	61.3
2132	south-coast	2005	11	1	305	125	16.5	50	52	89	57.1
2133	south-coast	2005	11	2	306	141	27.3	59	50	84	99
2134	south-coast	2005	11	3	307	137	32.3	45	49	71	95.9
2135	south-coast	2005	11	4	308	150	20.3	48	53	68	98.3
2136	south-coast	2005	11	5	309	124	50.8	55	44	77	96.8
2137	south-coast	2005	11	6	310	109	58.4	57	48	77	95.8
2138	south-coast	2005	11	7	311	137	61.1	58	50	82	98.3
2139	south-coast	2005	11	8	312	141	35.3	46	47	71	95.3
2140	south-coast	2005	11	9	313	137	16.6	30	55	66	98.7
2141	south-coast	2005	11	10	314	124	11.6	40	49	72	98.4
2142	south-coast	2005	11	11	315	132	15.3	38	48	65	97.7

2143	south-coast	2005	11	12	316	151	42.7	51	42	78	98.3
2144	south-coast	2005	11	13	317	129	33.2	47	46	86	97.9
2145	south-coast	2005	11	14	318	140	38.9	54	46	87	98.5
2146	south-coast	2005	11	15	319	144	20.5	47	50	86	75.7
2147	south-coast	2005	11	16	320	133	12.6	47	44	83	52
2148	south-coast	2005	11	17	321	137	21.4	39	44	85	52.6
2149	south-coast	2005	11	18	322	160	21.2	45	47	85	34.4
2150	south-coast	2005	11	19	323	140	14.9	43	43	89	40
2151	south-coast	2005	11	20	324	146	11.4	49	46	87	37.7
2152	south-coast	2005	11	21	325	137	24.2	53	46	85	42.8
2153	south-coast	2005	11	22	326	143	19.1	51	49	86	57.8
2154	south-coast	2005	11	23	327	140	30.4	44	50	79	90.5
2155	south-coast	2005	11	24	328	150	44.3	42	47	81	98.5
2156	south-coast	2005	11	25	329	142	54.5	56	45	74	99.5
2157	south-coast	2005	11	26	330	125	28.3	47	50	70	76.5
2158	south-coast	2005	11	27	331	130	11.4	44	36	69	32.4
2159	south-coast	2005	11	28	332	145	12.3	40	36	71	43.7
2160	south-coast	2005	11	29	333	131	21	34	39	69	79.4
2161	south-coast	2005	11	30	334	137	38.7	40	39	70	97.4
2162	south-coast	2005	12	1	335	140	46.7	37	43	75	99
2163	south-coast	2005	12	2	336	162	36.1	30	48	66	98.2
2164	south-coast	2005	12	3	337	139	15.3	42	46	64	81.3
2165	south-coast	2005	12	4	338	147	16.1	43	33	69	44.8
2166	south-coast	2005	12	5	339	156	15.5	43	33	69	35
2167	south-coast	2005	12	6	340	155	32.8	41	36	72	50.7
2168	south-coast	2005	12	7	341	159	27.2	44	37	73	71.6
2169	south-coast	2005	12	8	342	139	34.1	40	45	63	54.4
2170	south-coast	2005	12	9	343	147	13.2	45	46	71	67
2171	south-coast	2005	12	10	344	140	18.5	44	41	74	59.4
2172	south-coast	2005	12	11	345	148	24.7	47	44	74	68
2173	south-coast	2005	12	12	346	160	40.1	44	39	66	94.6
2174	south-coast	2005	12	13	347	160	44.9	45	38	65	98.4
2175	south-coast	2005	12	14	348	163	53.4	44	37	72	97.8
2176	south-coast	2005	12	15	349	148	40.8	42	42	67	98.3
2177	south-coast	2005	12	16	350	156	41.4	37	34	61	95.6
2178	south-coast	2005	12	17	351	153	36.4	46	43	59	96.3
2179	south-coast	2005	12	18	352	182	52.1	44	41	63	96
2180	south-coast	2005	12	19	353	193	29.6	33	41	73	90.3
2181	south-coast	2005	12	20	354	166	21.6	31	44	77	68.9
2182	south-coast	2005	12	21	355	185	34.1	34	48	85	83.7
2183	south-coast	2005	12	22	356	189	28.1	25	48	76	98.3
2184	south-coast	2005	12	23	357	186	44	36	49	80	99
2185	south-coast	2005	12	24	358	187	36.3	43	48	86	94.5
2186	south-coast	2005	12	25	359	190	47.7	50	46	77	99
2187	south-coast	2005	12	26	360	182	33.3	35	51	61	99.8
2188	south-coast	2005	12	27	361	193	41.2	36	49	66	99
2189	south-coast	2005	12	28	362	197	43.8	34	42	64	98.9
2190	south-coast	2005	12	29	363	167	34.5	40	47	65	98.9
2191	south-coast	2005	12	30	364	186	44.6	33	42	67	94.9

2192	south-coast	2005	12	31	365	166	25.1	34	43	61	99
2193	south-coast	2006	1	1	1	195	12.7	38	46	59	99
2194	south-coast	2006	1	2	2	167	16	42	50	61	100
2195	south-coast	2006	1	3	3	169	14.6	37	41	61	98.5
2196	south-coast	2006	1	4	4	159	18.3	43	42	78	69.5
2197	south-coast	2006	1	5	5	193	21.8	44	44	81	47.1
2198	south-coast	2006	1	6	6	176	12.9	45	44	83	46.3
2199	south-coast	2006	1	7	7	163	20.3	50	42	81	73.1
2200	south-coast	2006	1	8	8	155	31.8	43	39	73	93.1
2201	south-coast	2006	1	9	9	181	10.6	43	39	74	48.1
2202	south-coast	2006	1	10	10	189	11	47	39	72	49.5
2203	south-coast	2006	1	11	11	155	32.2	36	33	71	83.5
2204	south-coast	2006	1	12	12	157	38.9	35	40	74	97.2
2205	south-coast	2006	1	13	13	190	29.4	46	44	80	96.6
2206	south-coast	2006	1	14	14	150	27.3	41	44	67	98.3
2207	south-coast	2006	1	15	15	152	8.2	42	41	60	75.5
2208	south-coast	2006	1	16	16	149	11.9	41	41	66	52.9
2209	south-coast	2006	1	17	17	176	26.3	35	38	70	66.3
2210	south-coast	2006	1	18	18	159	25	38	38	64	97
2211	south-coast	2006	1	19	19	166	9.3	39	42	62	70.3
2212	south-coast	2006	1	20	20	141	16.1	43	33	69	57.4
2213	south-coast	2006	1	21	21	170	21.2	43	32	70	41.3
2214	south-coast	2006	1	22	22	162	17.4	47	35	69	39.7
2215	south-coast	2006	1	23	23	169	9	51	35	72	25.3
2216	south-coast	2006	1	24	24	158	11.2	49	41	75	42.4
2217	south-coast	2006	1	25	25	154	11.7	47	37	73	93
2218	south-coast	2006	1	26	26	163	23	50	42	63	96.5
2219	south-coast	2006	1	27	27	158	22.1	40	46	65	88.1
2220	south-coast	2006	1	28	28	154	28.5	43	39	65	95.6
2221	south-coast	2006	1	29	29	158	39.5	52	40	67	97.2
2222	south-coast	2006	1	30	30	149	52	51	40	69	97.5
2223	south-coast	2006	1	31	31	133	48.1	42	45	67	97.3
2224	south-coast	2006	2	1	32	164	43.9	44	43	68	97.1
2225	south-coast	2006	2	2	33	158	42.8	37	43	67	97.9
2226	south-coast	2006	2	3	34	176	41.8	48	44	88	98
2227	south-coast	2006	2	4	35	162	63	50	44	79	98
2228	south-coast	2006	2	5	36	146	43.7	50	49	86	92.8
2229	south-coast	2006	2	6	37	145	22.5	53	40	82	63
2230	south-coast	2006	2	7	38	152	23.3	49	42	82	39.4
2231	south-coast	2006	2	8	39	166	16	47	43	86	26.4
2232	south-coast	2006	2	9	40	148	23	48	46	86	53.7
2233	south-coast	2006	2	10	41	145	44.9	54	43	84	96.5
2234	south-coast	2006	2	11	42	148	58.5	53	42	87	98
2235	south-coast	2006	2	12	43	151	22.6	52	43	87	51.5
2236	south-coast	2006	2	13	44	137	44.5	45	46	88	35.7
2237	south-coast	2006	2	14	45	145	21	60	44	81	92.9
2238	south-coast	2006	2	15	46	167	12.6	52	49	63	85
2239	south-coast	2006	2	16	47	157	18.6	49	33	62	61.8
2240	south-coast	2006	2	17	48	166	12.3	52	40	61	83.5

2241	south-coast	2006	2	18	49	149	6.8	43	37	58	88.8
2242	south-coast	2006	2	19	50	160	9.8	48	40	57	92
2243	south-coast	2006	2	20	51	152	16.5	51	30	59	78.8
2244	south-coast	2006	2	21	52	161	17.5	47	34	64	70
2245	south-coast	2006	2	22	53	151	24.7	49	33	72	66.5
2246	south-coast	2006	2	23	54	157	23.1	48	37	74	83.9
2247	south-coast	2006	2	24	55	159	31.6	45	38	73	98
2248	south-coast	2006	2	25	56	165	50.9	49	39	76	97.8
2249	south-coast	2006	2	26	57	138	42.5	60	49	78	78.7
2250	south-coast	2006	2	27	58	151	28.3	61	46	72	79.9
2251	south-coast	2006	2	28	59	177	10.2	47	49	65	98
2252	south-coast	2006	3	1	60	153	11.4	44	45	66	85
2253	south-coast	2006	3	2	61	155	14.9	51	44	64	78.1
2254	south-coast	2006	3	3	62	160	17.5	48	53	59	95.1
2255	south-coast	2006	3	4	63	149	15.8	54	41	61	82
2256	south-coast	2006	3	5	64	159	27.6	61	43	65	73
2257	south-coast	2006	3	6	65	142	33.5	60	50	63	85.3
2258	south-coast	2006	3	7	66	147	9.4	49	51	62	88.2
2259	south-coast	2006	3	8	67	167	10.1	49	46	66	82.9
2260	south-coast	2006	3	9	68	148	17.8	47	48	63	91.5
2261	south-coast	2006	3	10	69	152	9.7	55	44	59	92.7
2262	south-coast	2006	3	11	70	138	7.9	56	40	53	99
2263	south-coast	2006	3	12	71	139	13.4	51	42	54	91.7
2264	south-coast	2006	3	13	72	153	14.8	51	43	58	75
2265	south-coast	2006	3	14	73	170	18.9	52	44	64	70.9
2266	south-coast	2006	3	15	74	153	16.2	53	49	64	78.3
2267	south-coast	2006	3	16	75	133	19.5	53	46	67	74.4
2268	south-coast	2006	3	17	76	146	17.1	47	42	61	92
2269	south-coast	2006	3	18	77	125	8.1	53	44	60	89.1
2270	south-coast	2006	3	19	78	156	15	54	43	59	94
2271	south-coast	2006	3	20	79	154	16.6	54	42	58	92.9
2272	south-coast	2006	3	21	80	153	9.5	53	50	61	85.3
2273	south-coast	2006	3	22	81	164	13.4	57	46	70	66
2274	south-coast	2006	3	23	82	140	12.9	54	47	79	58.3
2275	south-coast	2006	3	24	83	158	30.8	58	50	79	53.4
2276	south-coast	2006	3	25	84	156	19.2	60	50	76	87.4
2277	south-coast	2006	3	26	85	158	11.2	54	52	68	82.5
2278	south-coast	2006	3	27	86	154	20.1	57	52	67	81
2279	south-coast	2006	3	28	87	141	11.3	45	49	62	99.6
2280	south-coast	2006	3	29	88	136	10.7	48	43	62	87.2
2281	south-coast	2006	3	30	89	143	12.4	51	50	64	86.9
2282	south-coast	2006	3	31	90	160	10.5	44	50	61	96.5
2283	south-coast	2006	4	1	91	157	9.2	54	49	62	96
2284	south-coast	2006	4	2	92	147	21.6	60	39	69	90.3
2285	south-coast	2006	4	3	93	132	22	46	47	67	84.9
2286	south-coast	2006	4	4	94	169	9.5	53	53	72	96.8
2287	south-coast	2006	4	5	95	141	6.8	58	39	60	96.1
2288	south-coast	2006	4	6	96	153	12.7	53	41	65	95.8
2289	south-coast	2006	4	7	97	144	21.4	58	41	69	95.3

2290	south-coast	2006	4	8	98	156	19.6	57	34	68	95
2291	south-coast	2006	4	9	99	134	15	61	45	68	96.3
2292	south-coast	2006	4	10	100	130	8.7	51	43	63	97.1
2293	south-coast	2006	4	11	101	145	11.6	49	42	68	96.4
2294	south-coast	2006	4	12	102	146	15.8	57	43	80	96.4
2295	south-coast	2006	4	13	103	136	13.2	45	46	90	85.8
2296	south-coast	2006	4	14	104	143	11.1	46	55	84	98
2297	south-coast	2006	4	15	105	161	11.3	49	51	67	97.9
2298	south-coast	2006	4	16	106	138	9.7	52	47	65	97.2
2299	south-coast	2006	4	17	107	123	9.9	59	50	67	89.2
2300	south-coast	2006	4	18	108	146	15.5	65	44	80	77.8
2301	south-coast	2006	4	19	109	134	18.5	65	47	83	94.4
2302	south-coast	2006	4	20	110	128	22.8	74	46	81	96.8
2303	south-coast	2006	4	21	111	144	21.6	67	47	80	97.9
2304	south-coast	2006	4	22	112	124	15	50	45	67	95.8
2305	south-coast	2006	4	23	113	142	11.9	54	42	61	89.3
2306	south-coast	2006	4	24	114	141	15.6	53	45	69	95.9
2307	south-coast	2006	4	25	115	126	19.5	56	37	70	98
2308	south-coast	2006	4	26	116	124	16	50	47	63	96.9
2309	south-coast	2006	4	27	117	137	20.8	49	50	63	95.6
2310	south-coast	2006	4	28	118	128	23.3	69	53	63	98
2311	south-coast	2006	4	29	119	140	44.1	71	52	77	97.3
2312	south-coast	2006	4	30	120	130	46.3	89	48	79	98
2313	south-coast	2006	5	1	121	135	57.3	87	48	83	98
2314	south-coast	2006	5	2	122	131	55	72	55	79	98
2315	south-coast	2006	5	3	123	160	24.9	60	55	75	98
2316	south-coast	2006	5	4	124	112	21.3	59	50	65	97.5
2317	south-coast	2006	5	5	125	122	24.6	66	45	71	96.2
2318	south-coast	2006	5	6	126	139	35.9	71	55	73	98
2319	south-coast	2006	5	7	127	129	43.2	93	56	78	98
2320	south-coast	2006	5	8	128	124	50.9	82	49	79	98
2321	south-coast	2006	5	9	129	151	34.6	78	57	75	98
2322	south-coast	2006	5	10	130	139	53.7	81	46	89	98
2323	south-coast	2006	5	11	131	144	62.6	90	54	91	98
2324	south-coast	2006	5	12	132	128	52.7	98	55	84	98
2325	south-coast	2006	5	13	133	128	22.5	96	58	87	98
2326	south-coast	2006	5	14	134	130	36.4	114	53	91	98
2327	south-coast	2006	5	15	135	138	20.1	86	59	86	98
2328	south-coast	2006	5	16	136	140	25	87	50	90	98
2329	south-coast	2006	5	17	137	126	57.3	89	56	93	98
2330	south-coast	2006	5	18	138	146	57.2	101	53	89	98
2331	south-coast	2006	5	19	139	132	33.6	83	56	89	98
2332	south-coast	2006	5	20	140	122	32.1	75	56	85	98
2333	south-coast	2006	5	21	141	110	21.8	57	56	77	98
2334	south-coast	2006	5	22	142	141	11.5	54	48	68	98
2335	south-coast	2006	5	23	143	126	18.4	62	48	81	97.1
2336	south-coast	2006	5	24	144	126	21.3	77	44	91	98
2337	south-coast	2006	5	25	145	118	24.1	92	46	88	99.3
2338	south-coast	2006	5	26	146	129	25.8	71	59	80	100

2339	south-coast	2006	5	27	147	124	10.7	66	56	72	90.3
2340	south-coast	2006	5	28	148	104	10.8	72	46	81	77
2341	south-coast	2006	5	29	149	116	13.4	80	43	85	98.6
2342	south-coast	2006	5	30	150	155	16.3	85	51	92	97.7
2343	south-coast	2006	5	31	151	125	25.1	93	55	99	99.5
2344	south-coast	2006	6	1	152	143	22.9	103	57	94	78.6
2345	south-coast	2006	6	2	153	121	19.5	92	51	99	72.8
2346	south-coast	2006	6	3	154	131	18.6	131	59	104	65.2
2347	south-coast	2006	6	4	155	145	26.5	126	64	104	83.1
2348	south-coast	2006	6	5	156	122	30.4	101	62	91	86.9
2349	south-coast	2006	6	6	157	126	41.6	71	55	88	90.3
2350	south-coast	2006	6	7	158	111	42.6	85	58	86	94.1
2351	south-coast	2006	6	8	159	127	22.8	90	55	86	94.5
2352	south-coast	2006	6	9	160	131	18.9	81	54	83	91.2
2353	south-coast	2006	6	10	161	114	19.7	94	52	81	82.7
2354	south-coast	2006	6	11	162	110	16.2	84	46	83	88.3
2355	south-coast	2006	6	12	163	115	15	68	46	86	82.6
2356	south-coast	2006	6	13	164	111	16	69	41	86	70.3
2357	south-coast	2006	6	14	165	121	16	80	52	92	61.5
2358	south-coast	2006	6	15	166	132	15.6	80	46	88	73.5
2359	south-coast	2006	6	16	167	124	26.8	85	59	104	63.2
2360	south-coast	2006	6	17	168	119	18	111	62	96	83.3
2361	south-coast	2006	6	18	169	139	17.6	103	62	95	84.6
2362	south-coast	2006	6	19	170	122	26.5	92	49	94	82
2363	south-coast	2006	6	20	171	134	27.4	94	49	97	80.7
2364	south-coast	2006	6	21	172	117	22.1	109	55	90	80.5
2365	south-coast	2006	6	22	173	138	37.4	116	55	98	99
2366	south-coast	2006	6	23	174	123	24.8	114	46	97	82.2
2367	south-coast	2006	6	24	175	119	30.2	109	53	90	83.9
2368	south-coast	2006	6	25	176	123	29	112	60	99	73.5
2369	south-coast	2006	6	26	177	119	24.4	80	60	99	71.5
2370	south-coast	2006	6	27	178	131	19.2	76	56	95	68
2371	south-coast	2006	6	28	179	139	28.8	84	59	104	59.5
2372	south-coast	2006	6	29	180	118	15.3	93	62	102	69.6
2373	south-coast	2006	6	30	181	121	15.5	104	60	100	69.7
2374	south-coast	2006	7	1	182	137	19.4	119	63	106	60.8
2375	south-coast	2006	7	2	183	134	17.7	107	66	105	70.6
2376	south-coast	2006	7	3	184	105	17.1	91	68	100	72
2377	south-coast	2006	7	4	185	135	72.2	80	68	99	72.2
2378	south-coast	2006	7	5	186	127	39.3	89	64	99	70.7
2379	south-coast	2006	7	6	187	134	18.8	92	55	99	73.2
2380	south-coast	2006	7	7	188	124	20.6	100	58	104	72.5
2381	south-coast	2006	7	8	189	125	73.7	108	66	101	77.3
2382	south-coast	2006	7	9	190	121	16.8	104	66	103	79.5
2383	south-coast	2006	7	10	191	131	32.8	90	63	98	76.9
2384	south-coast	2006	7	11	192	125	17.8	74	63	100	78.2
2385	south-coast	2006	7	12	193	118	12.8	90	61	104	73
2386	south-coast	2006	7	13	194	132	15.5	99	50	106	78.6
2387	south-coast	2006	7	14	195	137	15.2	98	64	107	78.2

2388	south-coast	2006	7	15	196	136	20	135	66	105	70.5
2389	south-coast	2006	7	16	197	127	19.1	104	68	102	80
2390	south-coast	2006	7	17	198	138	15.4	73	58	100	83.2
2391	south-coast	2006	7	18	199	144	17	95	70	101	80.8
2392	south-coast	2006	7	19	200	127	21.9	106	70	100	77.8
2393	south-coast	2006	7	20	201	132	19.2	112	68	103	77.8
2394	south-coast	2006	7	21	202	132	21.4	109	69	112	78.4
2395	south-coast	2006	7	22	203	144	32.6	128	68	118	72.3
2396	south-coast	2006	7	23	204	131	20.4	108	71	110	77.2
2397	south-coast	2006	7	24	205	129	28.2	130	72	107	76.3
2398	south-coast	2006	7	25	206	139	26	142	73	107	87.5
2399	south-coast	2006	7	26	207	119	29	96	70	105	77.9
2400	south-coast	2006	7	27	208	121	21.8	77	73	99	82.3
2401	south-coast	2006	7	28	209	143	30.5	83	68	96	87.1
2402	south-coast	2006	7	29	210	133	13.3	70	72	92	96
2403	south-coast	2006	7	30	211	119	11	54	66	92	94.3
2404	south-coast	2006	7	31	212	117	10.4	58	66	81	94
2405	south-coast	2006	8	1	213	134	10.1	53	68	81	84.6
2406	south-coast	2006	8	2	214	129	12.3	82	66	84	79
2407	south-coast	2006	8	3	215	118	24.7	72	66	83	91.3
2408	south-coast	2006	8	4	216	123	12.7	80	66	82	83.5
2409	south-coast	2006	8	5	217	132	12.6	91	62	84	74.7
2410	south-coast	2006	8	6	218	128	17.7	90	62	82	84.5
2411	south-coast	2006	8	7	219	118	22.9	80	62	84	80.8
2412	south-coast	2006	8	8	220	127	18.2	102	62	90	73.3
2413	south-coast	2006	8	9	221	132	27.3	92	65	96	78
2414	south-coast	2006	8	10	222	106	17.4	97	66	93	78.3
2415	south-coast	2006	8	11	223	137	19.7	87	66	91	79.2
2416	south-coast	2006	8	12	224	119	24.1	89	63	87	77.9
2417	south-coast	2006	8	13	225	102	19.9	88	61	85	76.9
2418	south-coast	2006	8	14	226	106	22.1	84	60	83	77.4
2419	south-coast	2006	8	15	227	119	27.9	91	59	82	78
2420	south-coast	2006	8	16	228	123	25.1	86	60	82	78.7
2421	south-coast	2006	8	17	229	126	22.9	82	62	87	74.4
2422	south-coast	2006	8	18	230	132	24	79	61	87	79.8
2423	south-coast	2006	8	19	231	109	26.5	97	59	86	82.8
2424	south-coast	2006	8	20	232	136	25.3	98	61	85	82.6
2425	south-coast	2006	8	21	233	116	34.5	95	64	89	76.5
2426	south-coast	2006	8	22	234	125	20.4	96	65	96	74.2
2427	south-coast	2006	8	23	235	133	20.3	99	66	95	74.1
2428	south-coast	2006	8	24	236	125	22.1	79	64	92	78.5
2429	south-coast	2006	8	25	237	148	21.2	70	60	90	79.8
2430	south-coast	2006	8	26	238	140	16.6	85	65	88	76.3
2431	south-coast	2006	8	27	239	120	22.7	101	64	94	74.3
2432	south-coast	2006	8	28	240	126	17.1	96	63	92	78.6
2433	south-coast	2006	8	29	241	125	25.2	101	64	73	77.5
2434	south-coast	2006	8	30	242	120	25.7	104	64	92	81.4
2435	south-coast	2006	8	31	243	127	31.4	93	64	92	82.9
2436	south-coast	2006	9	1	244	111	21.3	83	62	104	77.6

2437	south-coast	2006	9	2	245	130	29.6	104	64	106	72
2438	south-coast	2006	9	3	246	124	16.5	93	66	107	69.5
2439	south-coast	2006	9	4	247	120	16.7	102	67	104	70.7
2440	south-coast	2006	9	5	248	103	18.3	82	65	107	70.6
2441	south-coast	2006	9	6	249	129	15.8	85	66	105	76.7
2442	south-coast	2006	9	7	250	130	24.3	83	64	99	79.1
2443	south-coast	2006	9	8	251	136	77.5	72	59	92	82.8
2444	south-coast	2006	9	9	252	113	43.7	78	58	91	78.8
2445	south-coast	2006	9	10	253	125	27.3	89	52	96	76.2
2446	south-coast	2006	9	11	254	112	22.9	63	58	99	82.1
2447	south-coast	2006	9	12	255	127	23.7	88	62	100	82.5
2448	south-coast	2006	9	13	256	127	21.7	89	63	95	84.1
2449	south-coast	2006	9	14	257	129	24.5	60	60	81	95.7
2450	south-coast	2006	9	15	258	103	10.3	49	60	79	86
2451	south-coast	2006	9	16	259	140	13.9	61	54	92	67.5
2452	south-coast	2006	9	17	260	116	24.9	74	52	97	64.7
2453	south-coast	2006	9	18	261	129	34.6	74	55	99	60.9
2454	south-coast	2006	9	19	262	126	48.7	84	56	93	74
2455	south-coast	2006	9	20	263	116	25.8	61	53	87	79.1
2456	south-coast	2006	9	21	264	124	15.7	69	52	80	79.4
2457	south-coast	2006	9	22	265	107	14	57	57	86	84.8
2458	south-coast	2006	9	23	266	117	11.4	51	55	94	77.6
2459	south-coast	2006	9	24	267	110	10.6	61	52	98	73.5
2460	south-coast	2006	9	25	268	123	18.8	59	56	99	83.8
2461	south-coast	2006	9	26	269	108	37.4	65	56	92	86.8
2462	south-coast	2006	9	27	270	132	26.3	59	54	98	83.7
2463	south-coast	2006	9	28	271	131	21.9	53	57	102	81
2464	south-coast	2006	9	29	272	144	77.8	63	56	97	80.3
2465	south-coast	2006	9	30	273	113	35.4	71	55	90	87.9
2466	south-coast	2006	10	1	274	115	38.5	66	61	78	86.2
2467	south-coast	2006	10	2	275	125	27.8	48	61	88	78.6
2468	south-coast	2006	10	3	276	127	11.3	39	55	87	74.6
2469	south-coast	2006	10	4	277	137	9.9	44	53	85	76.5
2470	south-coast	2006	10	5	278	123	14.4	48	50	80	77.8
2471	south-coast	2006	10	6	279	127	11.8	50	50	74	82.7
2472	south-coast	2006	10	7	280	130	12.8	58	50	80	79.2
2473	south-coast	2006	10	8	281	134	34.7	65	48	81	83.6
2474	south-coast	2006	10	9	282	131	37.4	59	54	75	91.4
2475	south-coast	2006	10	10	283	133	28.5	63	56	79	86
2476	south-coast	2006	10	11	284	125	32.6	63	46	85	78.8
2477	south-coast	2006	10	12	285	140	16.5	59	48	79	83.2
2478	south-coast	2006	10	13	286	116	8.1	44	57	77	81.4
2479	south-coast	2006	10	14	287	138	16.9	51	54	71	97.1
2480	south-coast	2006	10	15	288	132	17.8	51	55	69	98.5
2481	south-coast	2006	10	16	289	139	22.1	49	55	66	98.3
2482	south-coast	2006	10	17	290	121	14.4	45	51	74	88.1
2483	south-coast	2006	10	18	291	121	11.8	45	51	83	53.8
2484	south-coast	2006	10	19	292	127	11	47	46	90	35.1
2485	south-coast	2006	10	20	293	129	31.3	48	50	92	51.7

2486	south-coast	2006	10	21	294	141	30.4	61	50	96	79
2487	south-coast	2006	10	22	295	115	30.4	58	52	94	80.6
2488	south-coast	2006	10	23	296	119	21.3	55	53	92	65.6
2489	south-coast	2006	10	24	297	133	36	59	56	89	86.7
2490	south-coast	2006	10	25	298	120	42	63	51	82	90.2
2491	south-coast	2006	10	26	299	114	23.4	66	47	86	42.3
2492	south-coast	2006	10	27	300	124	13.8	52	43	91	28.5
2493	south-coast	2006	10	28	301	124	18.7	63	47	94	43.9
2494	south-coast	2006	10	29	302	163	36.5	79	45	84	80.7
2495	south-coast	2006	10	30	303	155	26.1	61	46	73	87.2
2496	south-coast	2006	10	31	304	112	32.3	57	53	71	86
2497	south-coast	2006	11	1	305	144	53.6	69	46	77	84.5
2498	south-coast	2006	11	2	306	125	28.3	58	47	82	85.3
2499	south-coast	2006	11	3	307	123	39.8	40	49	78	91.7
2500	south-coast	2006	11	4	308	144	27.1	48	49	90	81.3
2501	south-coast	2006	11	5	309	147	16.9	47	49	91	52.6
2502	south-coast	2006	11	6	310	123	16.6	46	52	97	33.4
2503	south-coast	2006	11	7	311	146	16.3	45	55	98	34.3
2504	south-coast	2006	11	8	312	124	25.7	64	52	90	67.3
2505	south-coast	2006	11	9	313	131	43.3	48	47	80	88.3
2506	south-coast	2006	11	10	314	122	28.1	53	44	86	69.5
2507	south-coast	2006	11	11	315	132	17.6	60	48	82	82.2
2508	south-coast	2006	11	12	316	116	12.6	48	45	79	62.2
2509	south-coast	2006	11	13	317	127	18.8	47	45	78	54.6
2510	south-coast	2006	11	14	318	121	16.6	36	46	74	88.2
2511	south-coast	2006	11	15	319	124	13	51	47	87	69.9
2512	south-coast	2006	11	16	320	125	24.7	51	47	88	55.2
2513	south-coast	2006	11	17	321	136	26.5	48	48	86	85.9
2514	south-coast	2006	11	18	322	144	38.3	56	50	93	84.2
2515	south-coast	2006	11	19	323	146	29.3	50	49	93	60.8
2516	south-coast	2006	11	20	324	132	24.6	50	54	93	50.8
2517	south-coast	2006	11	21	325	143	24.9	52	50	86	88.9
2518	south-coast	2006	11	22	326	126	50.6	50	47	79	87.3
2519	south-coast	2006	11	23	327	142	52.8	47	48	77	89.3
2520	south-coast	2006	11	24	328	114	54.4	51	43	67	94.3
2521	south-coast	2006	11	25	329	124	40.1	50	49	66	87.7
2522	south-coast	2006	11	26	330	137	21.4	49	43	67	89.3
2523	south-coast	2006	11	27	331	131	13.8	41	48	63	99.6
2524	south-coast	2006	11	28	332	132	18.1	43	47	62	86.8
2525	south-coast	2006	11	29	333	127	7.9	44	33	65	46.5
2526	south-coast	2006	11	30	334	139	18.4	40	30	68	35.1
2527	south-coast	2006	12	1	335	115	25	43	36	80	33.7
2528	south-coast	2006	12	2	336	155	18.8	47	40	80	41.9
2529	south-coast	2006	12	3	337	148	11.1	47	33	79	25.5
2530	south-coast	2006	12	4	338	135	25.1	46	33	80	28.4
2531	south-coast	2006	12	5	339	148	17.2	43	37	82	42.6
2532	south-coast	2006	12	6	340	150	30.3	41	37	85	74.7
2533	south-coast	2006	12	7	341	152	55.9	44	42	86	71.1
2534	south-coast	2006	12	8	342	167	24.2	47	43	81	59.4

2535	south-coast	2006	12	9	343	156	25.3	49	43	69	88
2536	south-coast	2006	12	10	344	140	7.8	42	48	62	92
2537	south-coast	2006	12	11	345	128	13.6	42	43	68	84.4
2538	south-coast	2006	12	12	346	115	22.2	42	41	71	73.8
2539	south-coast	2006	12	13	347	144	47	39	43	77	68.5
2540	south-coast	2006	12	14	348	134	36.1	30	47	81	74.5
2541	south-coast	2006	12	15	349	165	35.6	39	45	74	95.2
2542	south-coast	2006	12	16	350	152	21.9	39	49	62	97
2543	south-coast	2006	12	17	351	131	14.2	38	36	57	88
2544	south-coast	2006	12	18	352	158	12.7	40	31	59	64.9
2545	south-coast	2006	12	19	353	122	25.6	42	31	61	56.7
2546	south-coast	2006	12	20	354	142	19.7	34	30	60	60.5
2547	south-coast	2006	12	21	355	135	21.7	29	31	64	71.6
2548	south-coast	2006	12	22	356	165	31	40	38	66	66.7
2549	south-coast	2006	12	23	357	147	18.3	44	38	73	47.8
2550	south-coast	2006	12	24	358	138	31.5	38	40	79	54.5
2551	south-coast	2006	12	25	359	160	31.4	43	41	81	38.8
2552	south-coast	2006	12	26	360	147	30.4	41	43	76	57.7
2553	south-coast	2006	12	27	361	152	30.3	40	46	65	80
2554	south-coast	2006	12	28	362	160	12.6	38	37	63	55.7
2555	south-coast	2006	12	29	363	145	14.5	38	37	70	55
2556	south-coast	2006	12	30	364	141	14.2	36	34	70	78.6
2557	south-coast	2006	12	31	365	137	68.5	43	35	67	78.6
2558	south-coast	2007	1	1	1	151	38.4	42	36	72	68.8
2559	south-coast	2007	1	2	2	158	17.4	43	36	75	48.9
2560	south-coast	2007	1	3	3	139	19.9	38	44	75	61.3
2561	south-coast	2007	1	4	4	164	64.6	38	37	68	87.9
2562	south-coast	2007	1	5	5	136	6.1	45	40	61	47.5
2563	south-coast	2007	1	6	6	152	18.8	43	39	69	39
2564	south-coast	2007	1	7	7	160	19.1	46	41	76	40.9
2565	south-coast	2007	1	8	8	148	13.8	45	41	83	33.7
2566	south-coast	2007	1	9	9	188	14.6	41	41	84	37.5
2567	south-coast	2007	1	10	10	169	39.6	43	41	78	63.2
2568	south-coast	2007	1	11	11	160	19.2	44	37	66	85.9
2569	south-coast	2007	1	12	12	160	22.3	39	31	56	67.2
2570	south-coast	2007	1	13	13	166	11.7	40	27	55	40.4
2571	south-coast	2007	1	14	14	157	20.8	43	24	56	34
2572	south-coast	2007	1	15	15	139	30.7	45	27	60	41.6
2573	south-coast	2007	1	16	16	169	25	45	27	66	45.9
2574	south-coast	2007	1	17	17	183	15.1	43	30	62	57.5
2575	south-coast	2007	1	18	18	161	28.5	45	30	66	44.8
2576	south-coast	2007	1	19	19	149	13.8	41	46	70	59.6
2577	south-coast	2007	1	20	20	161	14.5	43	34	62	82.6
2578	south-coast	2007	1	21	21	170	20.1	44	38	64	65.6
2579	south-coast	2007	1	22	22	152	16.6	47	38	72	42.5
2580	south-coast	2007	1	23	23	164	20.4	36	36	70	47.8
2581	south-coast	2007	1	24	24	148	36	37	39	77	64.6
2582	south-coast	2007	1	25	25	172	28.3	45	40	79	54.3
2583	south-coast	2007	1	26	26	163	23.8	46	39	73	70.5

2584	south-coast	2007	1	27	27	156	24.4	48	45	62	89.4
2585	south-coast	2007	1	28	28	164	20.8	50	39	65	75.4
2586	south-coast	2007	1	29	29	174	30.2	50	40	70	83.7
2587	south-coast	2007	1	30	30	161	27.7	39	42	62	94.4
2588	south-coast	2007	1	31	31	162	14.6	44	43	57	97.8
2589	south-coast	2007	2	1	32	138	19.5	42	47	57	96.2
2590	south-coast	2007	2	2	33	162	28.5	45	36	66	77.5
2591	south-coast	2007	2	3	34	146	25.5	49	37	76	70.5
2592	south-coast	2007	2	4	35	143	17.4	47	41	86	44.4
2593	south-coast	2007	2	5	36	174	22.8	39	47	86	46
2594	south-coast	2007	2	6	37	147	29.9	51	48	82	83.3
2595	south-coast	2007	2	7	38	138	45.7	55	45	73	89.6
2596	south-coast	2007	2	8	39	151	48.7	46	38	72	85.6
2597	south-coast	2007	2	9	40	147	43.7	40	44	68	83.9
2598	south-coast	2007	2	10	41	149	38.3	40	46	75	87.6
2599	south-coast	2007	2	11	42	151	24.5	42	49	66	98.1
2600	south-coast	2007	2	12	43	177	7.3	43	47	61	91.3
2601	south-coast	2007	2	13	44	121	5.8	41	42	63	90
2602	south-coast	2007	2	14	45	158	17.5	42	40	70	70.6
2603	south-coast	2007	2	15	46	152	17.8	41	37	78	68.5
2604	south-coast	2007	2	16	47	151	16.7	47	46	85	67.3
2605	south-coast	2007	2	17	48	155	15	46	49	86	42.9
2606	south-coast	2007	2	18	49	138	10.5	53	50	82	66.7
2607	south-coast	2007	2	19	50	143	6.2	45	47	65	92.4
2608	south-coast	2007	2	20	51	132	17.4	45	40	65	87
2609	south-coast	2007	2	21	52	151	24.8	53	40	72	83.4
2610	south-coast	2007	2	22	53	158	14.3	43	44	65	95
2611	south-coast	2007	2	23	54	139	10.5	46	41	60	78.4
2612	south-coast	2007	2	24	55	153	9	49	33	70	53.6
2613	south-coast	2007	2	25	56	142	14.5	49	35	64	73.9
2614	south-coast	2007	2	26	57	140	22.6	44	47	63	92.7
2615	south-coast	2007	2	27	58	139	4.8	47	38	60	96
2616	south-coast	2007	2	28	59	138	4.9	45	37	61	81.8
2617	south-coast	2007	3	1	60	149	22.6	47	31	65	60.3
2618	south-coast	2007	3	2	61	137	10.5	48	33	72	48.5
2619	south-coast	2007	3	3	62	158	12.5	50	42	75	34.8
2620	south-coast	2007	3	4	63	169	9.6	52	38	79	31.2
2621	south-coast	2007	3	5	64	147	10.9	54	38	89	26.3
2622	south-coast	2007	3	6	65	145	20.5	48	55	86	48.6
2623	south-coast	2007	3	7	66	162	17	52	55	82	71.7
2624	south-coast	2007	3	8	67	162	18.2	50	43	76	79
2625	south-coast	2007	3	9	68	147	24.2	51	43	73	83.7
2626	south-coast	2007	3	10	69	163	37.3	57	43	89	80.9
2627	south-coast	2007	3	11	70	149	17	65	49	95	57.7
2628	south-coast	2007	3	12	71	139	20.1	66	52	94	42.5
2629	south-coast	2007	3	13	72	155	20.7	70	51	88	74.5
2630	south-coast	2007	3	14	73	139	27.5	66	46	82	79.8
2631	south-coast	2007	3	15	74	141	51.2	64	47	88	83.2
2632	south-coast	2007	3	16	75	144	60.2	83	50	96	85.9

2633	south-coast	2007	3	17	76	157	47	85	49	87	85.8
2634	south-coast	2007	3	18	77	153	42.4	80	48	72	87.8
2635	south-coast	2007	3	19	78	139	34.2	57	51	67	91.4
2636	south-coast	2007	3	20	79	146	28.5	46	48	66	95.8
2637	south-coast	2007	3	21	80	135	7.9	47	48	64	93.6
2638	south-coast	2007	3	22	81	124	9.6	43	41	80	72.2
2639	south-coast	2007	3	23	82	141	13.1	51	50	78	84.1
2640	south-coast	2007	3	24	83	134	9.9	62	51	73	84.8
2641	south-coast	2007	3	25	84	148	35.5	71	48	78	88
2642	south-coast	2007	3	26	85	128	23.1	68	49	69	95.6
2643	south-coast	2007	3	27	86	120	5.7	48	43	64	80.8
2644	south-coast	2007	3	28	87	127	8.9	50	36	67	56.7
2645	south-coast	2007	3	29	88	145	10.6	51	41	80	59.4
2646	south-coast	2007	3	30	89	146	10.8	60	43	81	63.4
2647	south-coast	2007	3	31	90	157	20	71	44	85	78.9
2648	south-coast	2007	4	1	91	128	22.6	85	45	85	77.9
2649	south-coast	2007	4	2	92	139	29.8	69	45	82	77.7
2650	south-coast	2007	4	3	93	137	30.5	59	47	82	81
2651	south-coast	2007	4	4	94	146	48.6	62	50	84	85.1
2652	south-coast	2007	4	5	95	152	49.3	75	54	82	87.7
2653	south-coast	2007	4	6	96	138	36.8	61	53	72	93.4
2654	south-coast	2007	4	7	97	137	25.8	55	53	68	99.1
2655	south-coast	2007	4	8	98	130	25	48	54	63	99.8
2656	south-coast	2007	4	9	99	154	22.3	51	53	71	90.5
2657	south-coast	2007	4	10	100	138	15.1	60	52	80	79.5
2658	south-coast	2007	4	11	101	130	14.7	62	46	73	79.5
2659	south-coast	2007	4	12	102	138	15	50	51	67	66.5
2660	south-coast	2007	4	13	103	148	9.2	53	45	81	41.9
2661	south-coast	2007	4	14	104	126	10.4	62	45	77	76
2662	south-coast	2007	4	15	105	122	14.8	53	46	67	94.6
2663	south-coast	2007	4	16	106	146	14.1	57	46	74	90.1
2664	south-coast	2007	4	17	107	126	20.1	60	44	76	85.5
2665	south-coast	2007	4	18	108	130	13.3	57	50	71	62.4
2666	south-coast	2007	4	19	109	130	10	66	38	74	45.8
2667	south-coast	2007	4	20	110	144	6.3	56	48	61	97.4
2668	south-coast	2007	4	21	111	152	24.3	57	44	65	89.7
2669	south-coast	2007	4	22	112	115	10.9	56	44	64	91
2670	south-coast	2007	4	23	113	140	8.5	51	47	71	86
2671	south-coast	2007	4	24	114	128	19	52	43	83	69.9
2672	south-coast	2007	4	25	115	158	15.8	67	48	86	73.8
2673	south-coast	2007	4	26	116	110	19.2	71	49	81	78.3
2674	south-coast	2007	4	27	117	146	27	65	50	98	72.2
2675	south-coast	2007	4	28	118	126	32.3	83	54	96	86.5
2676	south-coast	2007	4	29	119	145	38.5	98	55	93	89.7
2677	south-coast	2007	4	30	120	147	45.6	67	55	87	86.5
2678	south-coast	2007	5	1	121	152	38.1	63	54	76	88.3
2679	south-coast	2007	5	2	122	148	18.2	58	54	77	85
2680	south-coast	2007	5	3	123	131	12.1	58	53	74	78.2
2681	south-coast	2007	5	4	124	126	9.8	49	45	68	86.1

2682	south-coast	2007	5	5	125	151	11.4	61	50	73	68.5
2683	south-coast	2007	5	6	126	134	12.2	62	54	89	30.5
2684	south-coast	2007	5	7	127	129	9.1	67	50	93	28.8
2685	south-coast	2007	5	8	128	144	11.5	69	55	98	36.3
2686	south-coast	2007	5	9	129	119	19	80	57	95	77.4
2687	south-coast	2007	5	10	130	134	18.3	76	54	93	75.4
2688	south-coast	2007	5	11	131	116	14.5	69	52	88	74.5
2689	south-coast	2007	5	12	132	111	34.8	80	48	84	76.9
2690	south-coast	2007	5	13	133	141	27.2	93	48	85	76.5
2691	south-coast	2007	5	14	134	134	26.1	84	48	84	77.6
2692	south-coast	2007	5	15	135	142	33.1	77	52	80	87.8
2693	south-coast	2007	5	16	136	144	37.9	75	53	80	86.5
2694	south-coast	2007	5	17	137	139	44.9	75	52	84	84.8
2695	south-coast	2007	5	18	138	132	58	78	54	85	86
2696	south-coast	2007	5	19	139	133	49.7	97	52	85	85.5
2697	south-coast	2007	5	20	140	133	36.5	105	52	82	89.3
2698	south-coast	2007	5	21	141	118	17.2	70	56	72	97.8
2699	south-coast	2007	5	22	142	132	16.1	63	55	75	88.9
2700	south-coast	2007	5	23	143	115	19.5	60	55	85	78.7
2701	south-coast	2007	5	24	144	137	22.7	74	50	90	79.3
2702	south-coast	2007	5	25	145	127	21	80	54	88	81.1
2703	south-coast	2007	5	26	146	131	23.7	96	54	89	82.2
2704	south-coast	2007	5	27	147	135	31.3	91	53	87	79.5
2705	south-coast	2007	5	28	148	125	13.1	79	53	87	80.5
2706	south-coast	2007	5	29	149	100	12	92	57	83	81.3
2707	south-coast	2007	5	30	150	122	32.8	92	52	85	82.4
2708	south-coast	2007	5	31	151	124	31.3	78	50	83	83.1
2709	south-coast	2007	6	1	152	137	31.4	83	51	82	82.3
2710	south-coast	2007	6	2	153	142	30.6	102	56	86	84.7
2711	south-coast	2007	6	3	154	128	32	93	55	87	87.4
2712	south-coast	2007	6	4	155	114	39.9	97	55	93	81.5
2713	south-coast	2007	6	5	156	110	29.3	66	56	79	95.2
2714	south-coast	2007	6	6	157	126	21.2	50	55	73	79
2715	south-coast	2007	6	7	158	117	11.4	58	47	85	66.4
2716	south-coast	2007	6	8	159	144	16.3	66	50	84	76.8
2717	south-coast	2007	6	9	160	125	20.2	85	52	84	83.3
2718	south-coast	2007	6	10	161	146	21.1	94	52	91	83
2719	south-coast	2007	6	11	162	114	33.2	92	54	87	84.3
2720	south-coast	2007	6	12	163	123	26.5	91	52	95	73.6
2721	south-coast	2007	6	13	164	139	23.4	90	58	101	76.6
2722	south-coast	2007	6	14	165	152	20.7	102	59	97	77.1
2723	south-coast	2007	6	15	166	118	14	89	59	96	80.2
2724	south-coast	2007	6	16	167	117	15.1	83	58	95	78
2725	south-coast	2007	6	17	168	125	22.8	80	56	91	79.7
2726	south-coast	2007	6	18	169	123	19.2	88	56	92	77.2
2727	south-coast	2007	6	19	170	109	14.4	83	56	98	73.7
2728	south-coast	2007	6	20	171	141	16.3	71	56	98	76.8
2729	south-coast	2007	6	21	172	134	16.3	83	57	95	75.3
2730	south-coast	2007	6	22	173	137	17.5	105	59	100	80.2

2731	south-coast	2007	6	23	174	143	23.9	113	59	93	85.1
2732	south-coast	2007	6	24	175	132	18.2	104	57	93	81.9
2733	south-coast	2007	6	25	176	114	17.6	86	56	96	75.9
2734	south-coast	2007	6	26	177	135	20.6	90	57	96	76.8
2735	south-coast	2007	6	27	178	133	18.7	92	56	95	76.1
2736	south-coast	2007	6	28	179	108	17.1	97	59	96	74.1
2737	south-coast	2007	6	29	180	115	23.6	103	59	99	74.5
2738	south-coast	2007	6	30	181	127	18.7	124	59	99	75
2739	south-coast	2007	7	1	182	126	18	137	61	102	73
2740	south-coast	2007	7	2	183	129	20.3	131	64	103	73.3
2741	south-coast	2007	7	3	184	133	18.7	135	62	106	71.7
2742	south-coast	2007	7	4	185	96	43.6	126	63	103	82.7
2743	south-coast	2007	7	5	186	119	78.5	124	64	99	81.8
2744	south-coast	2007	7	6	187	117	22.7	110	64	102	82.1
2745	south-coast	2007	7	7	188	123	24.6	106	63	101	81.1
2746	south-coast	2007	7	8	189	108	20.2	95	61	94	83.7
2747	south-coast	2007	7	9	190	98	19.4	81	60	92	81.6
2748	south-coast	2007	7	10	191	136	17.2	61	62	89	86.9
2749	south-coast	2007	7	11	192	124	24.9	59	62	89	80.8
2750	south-coast	2007	7	12	193	118	10.9	67	56	93	71.2
2751	south-coast	2007	7	13	194	117	16.1	95	58	101	69
2752	south-coast	2007	7	14	195	120	19.8	96	68	98	73.8
2753	south-coast	2007	7	15	196	136	14.6	82	65	97	84.4
2754	south-coast	2007	7	16	197	113	19.3	87	63	96	84.2
2755	south-coast	2007	7	17	198	112	56.5	94	60	94	79.1
2756	south-coast	2007	7	18	199	103	11	80	60	94	73.3
2757	south-coast	2007	7	19	200	125	11.1	76	60	97	79.5
2758	south-coast	2007	7	20	201	113	18.9	91	61	96	85
2759	south-coast	2007	7	21	202	121	18.3	92	61	96	84.8
2760	south-coast	2007	7	22	203	104	21.9	99	63	93	80.5
2761	south-coast	2007	7	23	204	117	22.7	88	63	93	81.8
2762	south-coast	2007	7	24	205	120	16.8	91	65	97	79.4
2763	south-coast	2007	7	25	206	114	19	89	66	100	74.2
2764	south-coast	2007	7	26	207	132	16.8	80	66	101	70.2
2765	south-coast	2007	7	27	208	132	13.7	88	65	99	72.3
2766	south-coast	2007	7	28	209	122	14.3	91	65	98	74.6
2767	south-coast	2007	7	29	210	106	16.1	102	63	98	74.9
2768	south-coast	2007	7	30	211	122	15.1	104	62	96	81.1
2769	south-coast	2007	7	31	212	131	16.7	94	65	94	85.4
2770	south-coast	2007	8	1	213	139	28.1	86	65	94	84.7
2771	south-coast	2007	8	2	214	119	21.4	104	64	96	82.6
2772	south-coast	2007	8	3	215	113	20.5	103	63	98	79.5
2773	south-coast	2007	8	4	216	101	18.2	115	65	96	81.3
2774	south-coast	2007	8	5	217	118	15.3	90	64	91	83
2775	south-coast	2007	8	6	218	119	16.1	64	62	91	78.5
2776	south-coast	2007	8	7	219	120	16.6	64	57	88	76
2777	south-coast	2007	8	8	220	123	12.4	83	57	92	73.5
2778	south-coast	2007	8	9	221	115	15.9	88	57	95	78.4
2779	south-coast	2007	8	10	222	116	26	102	59	95	78

2780	south-coast	2007	8	11	223	111	24.4	124	60	97	80.8
2781	south-coast	2007	8	12	224	119	17.3	113	63	102	76.8
2782	south-coast	2007	8	13	225	122	16	88	64	104	71
2783	south-coast	2007	8	14	226	118	14.8	75	65	105	72.8
2784	south-coast	2007	8	15	227	123	14.9	95	68	103	71.2
2785	south-coast	2007	8	16	228	117	32.6	90	68	104	65
2786	south-coast	2007	8	17	229	138	17.2	92	68	101	75.6
2787	south-coast	2007	8	18	230	132	16.9	80	67	103	67.9
2788	south-coast	2007	8	19	231	107	22	96	68	102	70.7
2789	south-coast	2007	8	20	232	103	18.8	90	62	103	69
2790	south-coast	2007	8	21	233	130	17.6	103	61	102	76.3
2791	south-coast	2007	8	22	234	118	23.9	107	61	98	83.4
2792	south-coast	2007	8	23	235	115	19.4	86	61	94	83.4
2793	south-coast	2007	8	24	236	123	26.3	91	61	97	82.8
2794	south-coast	2007	8	25	237	122	25.3	93	63	94	80
2795	south-coast	2007	8	26	238	105	12.2	80	66	94	72.2
2796	south-coast	2007	8	27	239	124	10.8	75	65	98	72.3
2797	south-coast	2007	8	28	240	140	18.3	73	65	104	72.8
2798	south-coast	2007	8	29	241	124	16.4	74	64	109	72.5
2799	south-coast	2007	8	30	242	134	18.3	83	67	113	68.6
2800	south-coast	2007	8	31	243	126	19	97	66	110	70.5
2801	south-coast	2007	9	1	244	123	16.8	113	67	110	68.4
2802	south-coast	2007	9	2	245	157	20.1	102	68	106	63
2803	south-coast	2007	9	3	246	140	24.5	116	69	108	57.6
2804	south-coast	2007	9	4	247	129	13.5	74	70	104	61.5
2805	south-coast	2007	9	5	248	126	13.7	71	63	102	81
2806	south-coast	2007	9	6	249	106	13.8	66	58	89	82.6
2807	south-coast	2007	9	7	250	111	12.6	69	57	92	78.2
2808	south-coast	2007	9	8	251	120	12.3	80	58	92	75.3
2809	south-coast	2007	9	9	252	123	15.7	88	58	89	73.8
2810	south-coast	2007	9	10	253	142	11.2	58	56	94	67.7
2811	south-coast	2007	9	11	254	132	13.4	67	56	99	70.3
2812	south-coast	2007	9	12	255	133	16.6	63	61	100	70.4
2813	south-coast	2007	9	13	256	113	14.7	69	62	100	70
2814	south-coast	2007	9	14	257	110	14.1	59	57	93	70
2815	south-coast	2007	9	15	258	124	45.7	78	52	94	73.5
2816	south-coast	2007	9	16	259	133	13	75	51	90	80.4
2817	south-coast	2007	9	17	260	127	12.1	59	55	81	81.5
2818	south-coast	2007	9	18	261	106	20.6	73	54	84	82.9
2819	south-coast	2007	9	19	262	118	15.3	57	58	79	90.6
2820	south-coast	2007	9	20	263	118	11.6	49	56	71	89.9
2821	south-coast	2007	9	21	264	126	15.6	49	46	85	72.3
2822	south-coast	2007	9	22	265	134	10.8	43	55	72	96.6
2823	south-coast	2007	9	23	266	121	10.6	56	53	75	89.1
2824	south-coast	2007	9	24	267	136	18.4	67	49	89	77.4
2825	south-coast	2007	9	25	268	124	16.4	71	54	92	75
2826	south-coast	2007	9	26	269	114	17.5	71	55	95	75.7
2827	south-coast	2007	9	27	270	134	26	83	56	92	80.2
2828	south-coast	2007	9	28	271	121	19	55	56	82	92.8

2829	south-coast	2007	9	29	272	118	10.5	59	56	80	77.8
2830	south-coast	2007	9	30	273	133	13.5	65	52	97	70
2831	south-coast	2007	10	1	274	114	13	59	58	90	73.8
2832	south-coast	2007	10	2	275	127	16.3	55	56	95	73
2833	south-coast	2007	10	3	276	115	20.7	56	50	93	72.3
2834	south-coast	2007	10	4	277	125	20.6	60	52	86	83
2835	south-coast	2007	10	5	278	135	15	47	56	73	86.5
2836	south-coast	2007	10	6	279	128	15.6	50	43	79	56.7
2837	south-coast	2007	10	7	280	136	8.1	57	41	86	38
2838	south-coast	2007	10	8	281	126	13.9	57	47	92	43.6
2839	south-coast	2007	10	9	282	125	19.3	55	47	87	70.5
2840	south-coast	2007	10	10	283	121	15.1	55	46	80	76.9
2841	south-coast	2007	10	11	284	122	18.3	57	47	80	83.5
2842	south-coast	2007	10	12	285	139	15.7	55	50	72	86.2
2843	south-coast	2007	10	13	286	126	13.9	36	52	69	95.8
2844	south-coast	2007	10	14	287	143	31.7	53	55	79	89.1
2845	south-coast	2007	10	15	288	106	46.6	55	51	70	99.1
2846	south-coast	2007	10	16	289	140	26.1	50	54	69	97.3
2847	south-coast	2007	10	17	290	128	10.7	45	48	72	91.1
2848	south-coast	2007	10	18	291	138	16.5	57	50	89	70.3
2849	south-coast	2007	10	19	292	129	16.7	47	51	93	59.8
2850	south-coast	2007	10	20	293	149	17.6	61	53	87	78.3
2851	south-coast	2007	10	21	294	125	42	49	50	84	45.1
2852	south-coast	2007	10	22	295	104	32.7	57	50	87	28.5
2853	south-coast	2007	10	23	296	148	29.7	67	52	94	33
2854	south-coast	2007	10	24	297	136	72.1	54	56	99	41.5
2855	south-coast	2007	10	25	298	169	50.4	52	56	94	48.5
2856	south-coast	2007	10	26	299	119	75.7	77	52	91	83.5
2857	south-coast	2007	10	27	300	139	47.7	54	54	79	78.7
2858	south-coast	2007	10	28	301	135	24.5	57	57	95	68.6
2859	south-coast	2007	10	29	302	132	19.3	45	58	85	70.4
2860	south-coast	2007	10	30	303	126	17.5	45	54	76	85.8
2861	south-coast	2007	10	31	304	120	27.1	50	48	83	81.8
2862	south-coast	2007	11	1	305	109	45	55	48	80	90.9
2863	south-coast	2007	11	2	306	126	58.6	47	46	90	87
2864	south-coast	2007	11	3	307	115	47	54	50	91	86
2865	south-coast	2007	11	4	308	133	48.9	53	51	88	91
2866	south-coast	2007	11	5	309	104	70.8	48	48	75	94.8
2867	south-coast	2007	11	6	310	130	42.9	48	46	78	94.2
2868	south-coast	2007	11	7	311	145	51.5	47	46	82	94.9
2869	south-coast	2007	11	8	312	106	72.4	44	54	65	96.9
2870	south-coast	2007	11	9	313	146	46.5	51	53	66	98.5
2871	south-coast	2007	11	10	314	128	16.1	52	49	69	89.6
2872	south-coast	2007	11	11	315	145	29	45	44	65	91.6
2873	south-coast	2007	11	12	316	125	26.3	46	49	84	71.5
2874	south-coast	2007	11	13	317	111	12.4	46	49	89	52
2875	south-coast	2007	11	14	318	128	37.5	38	53	92	45.3
2876	south-coast	2007	11	15	319	156	17.1	38	52	90	53.8
2877	south-coast	2007	11	16	320	111	41	45	51	82	91

2878	south-coast	2007	11	17	321	160	69.6	60	48	75	88.7
2879	south-coast	2007	11	18	322	116	72.1	61	44	85	94.1
2880	south-coast	2007	11	19	323	123	82.9	56	46	84	90.2
2881	south-coast	2007	11	20	324	135	67	49	45	78	92.6
2882	south-coast	2007	11	21	325	123	56.7	43	40	78	81.9
2883	south-coast	2007	11	22	326	129	42.7	55	39	68	88.5
2884	south-coast	2007	11	23	327	120	29.5	51	39	69	74.2
2885	south-coast	2007	11	24	328	116	23.5	46	44	81	41.5
2886	south-coast	2007	11	25	329	137	16.3	46	42	71	48.8
2887	south-coast	2007	11	26	330	125	26.5	43	40	77	45.4
2888	south-coast	2007	11	27	331	157	21.3	38	45	75	53.4
2889	south-coast	2007	11	28	332	135	15.6	42	44	75	36.7
2890	south-coast	2007	11	29	333	140	26.3	40	43	77	35.2
2891	south-coast	2007	11	30	334	154	17.5	38	51	71	85.8
2892	south-coast	2007	12	1	335	143	12	41	45	59	95.3
2893	south-coast	2007	12	2	336	113	20.5	42	35	62	78.6
2894	south-coast	2007	12	3	337	145	13.8	42	35	80	71.3
2895	south-coast	2007	12	4	338	135	29.9	49	42	87	61.2
2896	south-coast	2007	12	5	339	152	37.4	39	47	81	80.3
2897	south-coast	2007	12	6	340	148	16.2	45	42	72	90.1
2898	south-coast	2007	12	7	341	148	6.7	41	49	60	100
2899	south-coast	2007	12	8	342	125	10.7	39	42	58	97.7
2900	south-coast	2007	12	9	343	127	9.1	41	40	63	82.3
2901	south-coast	2007	12	10	344	127	12.7	38	34	63	71.4
2902	south-coast	2007	12	11	345	140	11.6	41	38	62	68.2
2903	south-coast	2007	12	12	346	128	12.1	42	33	65	64.2
2904	south-coast	2007	12	13	347	155	21.5	41	34	65	56.1
2905	south-coast	2007	12	14	348	138	38.4	41	33	64	72.5
2906	south-coast	2007	12	15	349	147	33	38	35	70	60.7
2907	south-coast	2007	12	16	350	145	22.8	39	38	71	55.7
2908	south-coast	2007	12	17	351	157	31	39	35	61	82.8
2909	south-coast	2007	12	18	352	132	16.2	37	40	59	99.4
2910	south-coast	2007	12	19	353	134	5.5	40	49	62	100
2911	south-coast	2007	12	20	354	140	16.6	40	48	59	100
2912	south-coast	2007	12	21	355	121	10.6	46	44	63	65.6
2913	south-coast	2007	12	22	356	129	19.4	43	31	64	46.6
2914	south-coast	2007	12	23	357	128	16.6	46	37	78	39.2
2915	south-coast	2007	12	24	358	160	18.1	41	44	74	52.2
2916	south-coast	2007	12	25	359	143	15.4	45	33	69	44
2917	south-coast	2007	12	26	360	145	16.1	46	32	64	46
2918	south-coast	2007	12	27	361	168	8.5	40	40	58	46
2919	south-coast	2007	12	28	362	162	12.9	34	33	58	50
2920	south-coast	2007	12	29	363	151	37.4	33	33	57	82.7
2921	south-coast	2007	12	30	364	145	51.2	36	34	62	88.1
2922	south-coast	2007	12	31	365	138	22.2	45	37	73	58.3
2923	south-coast	2008	1	1	1	146	50	44	35	70	39.2
2924	south-coast	2008	1	2	2	166	13.4	39	35	74	36.2
2925	south-coast	2008	1	3	3	150	20	49	43	70	47.8
2926	south-coast	2008	1	4	4	156	14.5	41	44	63	95.8

2927	south-coast	2008	1	5	5	150	14.8	43	50	60	100
2928	south-coast	2008	1	6	6	178	8.8	43	47	57	99
2929	south-coast	2008	1	7	7	131	12.1	39	40	60	96.6
2930	south-coast	2008	1	8	8	155	31.1	38	37	58	93.7
2931	south-coast	2008	1	9	9	162	35.3	35	43	60	97.8
2932	south-coast	2008	1	10	10	149	56.2	47	37	67	87.1
2933	south-coast	2008	1	11	11	159	53.1	44	42	72	85.5
2934	south-coast	2008	1	12	12	146	55.1	43	42	78	71.9
2935	south-coast	2008	1	13	13	143	23.3	47	40	77	50
2936	south-coast	2008	1	14	14	132	19.1	43	42	75	42.3
2937	south-coast	2008	1	15	15	168	22.7	38	38	69	57.5
2938	south-coast	2008	1	16	16	175	37	47	38	66	61.4
2939	south-coast	2008	1	17	17	176	13.6	47	33	67	33
2940	south-coast	2008	1	18	18	178	19	45	34	67	48.4
2941	south-coast	2008	1	19	19	152	33.3	41	35	68	54
2942	south-coast	2008	1	20	20	162	26.8	46	33	64	70.2
2943	south-coast	2008	1	21	21	166	14.3	43	39	59	98.9
2944	south-coast	2008	1	22	22	147	28.8	44	42	56	95
2945	south-coast	2008	1	23	23	170	17.1	41	34	59	94.1
2946	south-coast	2008	1	24	24	150	10.6	42	38	55	99
2947	south-coast	2008	1	25	25	174	15.5	38	43	59	99
2948	south-coast	2008	1	26	26	157	14.1	38	35	68	81.8
2949	south-coast	2008	1	27	27	156	9	48	45	65	99
2950	south-coast	2008	1	28	28	164	10.4	44	45	57	99
2951	south-coast	2008	1	29	29	169	15.3	39	40	58	94.8
2952	south-coast	2008	1	30	30	161	15.7	44	42	61	79.8
2953	south-coast	2008	1	31	31	150	14.3	38	33	66	66.6
2954	south-coast	2008	2	1	32	165	19.9	37	34	61	85.8
2955	south-coast	2008	2	2	33	179	16.5	41	42	58	97.1
2956	south-coast	2008	2	3	34	165	12.2	41	44	56	100
2957	south-coast	2008	2	4	35	155	7.9	41	37	60	86.8
2958	south-coast	2008	2	5	36	187	11.5	45	33	62	56.2
2959	south-coast	2008	2	6	37	162	24.6	40	33	62	67.8
2960	south-coast	2008	2	7	38	170	31.7	36	36	73	69.9
2961	south-coast	2008	2	8	39	185	44.9	41	41	81	68.7
2962	south-coast	2008	2	9	40	167	27.5	41	43	82	61.5
2963	south-coast	2008	2	10	41	169	27.4	43	44	80	65
2964	south-coast	2008	2	11	42	152	35.6	44	45	81	63.5
2965	south-coast	2008	2	12	43	166	40.5	43	47	83	74.3
2966	south-coast	2008	2	13	44	169	32.8	56	46	79	93
2967	south-coast	2008	2	14	45	161	16.7	44	45	60	75.8
2968	south-coast	2008	2	15	46	161	15	53	46	68	50.8
2969	south-coast	2008	2	16	47	178	20	43	35	67	72.8
2970	south-coast	2008	2	17	48	176	37.3	54	36	65	87.2
2971	south-coast	2008	2	18	49	170	53.3	49	41	69	91.3
2972	south-coast	2008	2	19	50	173	34.7	52	48	60	99
2973	south-coast	2008	2	20	51	167	16.1	42	48	58	100
2974	south-coast	2008	2	21	52	175	13.7	46	42	58	98.3
2975	south-coast	2008	2	22	53	171	10.1	47	44	60	100

2976	south-coast	2008	2	23	54	180	11.6	49	37	63	86.1
2977	south-coast	2008	2	24	55	189	14	48	46	59	95.5
2978	south-coast	2008	2	25	56	169	19.8	45	43	69	77.4
2979	south-coast	2008	2	26	57	164	11.7	47	42	80	52.3
2980	south-coast	2008	2	27	58	181	16	48	44	83	64.1
2981	south-coast	2008	2	28	59	199	25.5	54	46	81	85.8
2982	south-coast	2008	2	29	60	163	34.8	52	44	70	94.5
2983	south-coast	2008	3	1	61	165	43.3	55	43	66	99.2
2984	south-coast	2008	3	2	62	181	28	55	43	73	69.5
2985	south-coast	2008	3	3	63	155	14.1	50	38	74	48.2
2986	south-coast	2008	3	4	64	180	18	47	40	75	60.4
2987	south-coast	2008	3	5	65	158	23.4	55	40	76	79.1
2988	south-coast	2008	3	6	66	158	26.6	56	37	73	67.8
2989	south-coast	2008	3	7	67	158	20.7	51	42	78	64.8
2990	south-coast	2008	3	8	68	169	28.7	62	44	74	70.9
2991	south-coast	2008	3	9	69	164	18.5	64	50	79	65.6
2992	south-coast	2008	3	10	70	167	12	58	47	83	46.3
2993	south-coast	2008	3	11	71	181	27.4	67	48	83	55.6
2994	south-coast	2008	3	12	72	176	34.6	64	48	81	72.2
2995	south-coast	2008	3	13	73	157	30.3	60	50	75	83.8
2996	south-coast	2008	3	14	74	169	21.8	51	48	75	95.6
2997	south-coast	2008	3	15	75	158	13.9	52	46	64	87.5
2998	south-coast	2008	3	16	76	133	7.9	50	35	66	83.8
2999	south-coast	2008	3	17	77	176	10.8	51	43	71	59.1
3000	south-coast	2008	3	18	78	165	16.5	51	39	76	67.6
3001	south-coast	2008	3	19	79	176	31.8	58	43	76	83.1
3002	south-coast	2008	3	20	80	152	46	61	42	73	82.9
3003	south-coast	2008	3	21	81	158	31	57	42	83	67.2
3004	south-coast	2008	3	22	82	147	25.2	62	46	86	56.5
3005	south-coast	2008	3	23	83	170	18.4	63	47	88	29.3
3006	south-coast	2008	3	24	84	137	23.4	72	49	86	52.8
3007	south-coast	2008	3	25	85	154	33.6	72	48	83	82.1
3008	south-coast	2008	3	26	86	153	32.9	66	47	77	77.7
3009	south-coast	2008	3	27	87	134	22.4	61	47	75	80.5
3010	south-coast	2008	3	28	88	151	22	67	45	72	82.6
3011	south-coast	2008	3	29	89	137	22.3	57	49	66	93.4
3012	south-coast	2008	3	30	90	148	12.6	53	50	66	99
3013	south-coast	2008	3	31	91	164	17.9	54	44	69	83.8
3014	south-coast	2008	4	1	92	132	32.4	62	40	69	86
3015	south-coast	2008	4	2	93	139	31	57	41	68	82.8
3016	south-coast	2008	4	3	94	125	20.6	45	50	69	96.6
3017	south-coast	2008	4	4	95	139	30.7	51	39	79	85.3
3018	south-coast	2008	4	5	96	131	28.9	51	50	72	97.4
3019	south-coast	2008	4	6	97	144	20.3	58	50	67	97.1
3020	south-coast	2008	4	7	98	133	24.8	58	50	71	89.5
3021	south-coast	2008	4	8	99	125	22.5	59	48	66	94.8
3022	south-coast	2008	4	9	100	126	16.8	56	45	67	87.9
3023	south-coast	2008	4	10	101	129	20.5	61	39	82	65
3024	south-coast	2008	4	11	102	136	23	64	47	90	50.1

3025	south-coast	2008	4	12	103	138	17.9	66	49	93	25.5
3026	south-coast	2008	4	13	104	157	10.9	68	52	98	32.6
3027	south-coast	2008	4	14	105	149	20.2	69	55	93	50.2
3028	south-coast	2008	4	15	106	148	23.9	60	46	83	82.5
3029	south-coast	2008	4	16	107	144	25	64	49	82	72.3
3030	south-coast	2008	4	17	108	133	24.1	76	47	87	59.9
3031	south-coast	2008	4	18	109	122	25.3	82	49	88	73.3
3032	south-coast	2008	4	19	110	140	25.3	79	47	81	81.4
3033	south-coast	2008	4	20	111	141	21.9	73	43	69	83.5
3034	south-coast	2008	4	21	112	145	25.3	72	42	74	79.6
3035	south-coast	2008	4	22	113	131	31	82	44	77	68.7
3036	south-coast	2008	4	23	114	134	21	65	43	72	81.4
3037	south-coast	2008	4	24	115	148	24.9	67	47	81	72.2
3038	south-coast	2008	4	25	116	137	23.5	71	47	90	52.4
3039	south-coast	2008	4	26	117	136	23.6	86	52	96	31.8
3040	south-coast	2008	4	27	118	144	20	77	56	95	25.3
3041	south-coast	2008	4	28	119	138	22.5	82	55	99	34.6
3042	south-coast	2008	4	29	120	138	31.8	89	54	91	74.8
3043	south-coast	2008	4	30	121	123	22.2	65	54	78	97.8
3044	south-coast	2008	5	1	122	122	23.5	75	49	83	67.4
3045	south-coast	2008	5	2	123	125	23.4	81	46	88	62.5
3046	south-coast	2008	5	3	124	135	26.1	88	46	84	69.9
3047	south-coast	2008	5	4	125	129	21.6	76	47	80	80.3
3048	south-coast	2008	5	5	126	126	15.8	60	51	71	82.5
3049	south-coast	2008	5	6	127	127	28.4	66	53	72	92
3050	south-coast	2008	5	7	128	128	21.3	52	52	64	99
3051	south-coast	2008	5	8	129	158	36.3	72	52	76	85
3052	south-coast	2008	5	9	130	121	49	63	48	71	96.8
3053	south-coast	2008	5	10	131	134	31.3	86	54	81	85.2
3054	south-coast	2008	5	11	132	121	37.1	82	50	79	87.5
3055	south-coast	2008	5	12	133	107	21	61	50	70	99.4
3056	south-coast	2008	5	13	134	114	21.7	71	47	86	72.7
3057	south-coast	2008	5	14	135	121	29.8	78	49	90	70.9
3058	south-coast	2008	5	15	136	147	33.5	71	57	99	73
3059	south-coast	2008	5	16	137	111	20.2	71	61	99	62.3
3060	south-coast	2008	5	17	138	146	25.1	96	60	103	63
3061	south-coast	2008	5	18	139	142	28.6	118	64	103	64.8
3062	south-coast	2008	5	19	140	113	24.9	113	64	100	71.6
3063	south-coast	2008	5	20	141	138	25.3	81	60	91	84
3064	south-coast	2008	5	21	142	130	22.1	68	56	82	85
3065	south-coast	2008	5	22	143	133	22.6	54	51	80	96.8
3066	south-coast	2008	5	23	144	116	17.9	46	47	64	99
3067	south-coast	2008	5	24	145	119	9	53	46	62	92.9
3068	south-coast	2008	5	25	146	116	10.5	56	44	65	84.8
3069	south-coast	2008	5	26	147	142	12.6	54	49	66	88
3070	south-coast	2008	5	27	148	97	18.1	62	45	74	85.5
3071	south-coast	2008	5	28	149	129	13.9	58	46	78	71.9
3072	south-coast	2008	5	29	150	113	15.3	64	46	79	67.9
3073	south-coast	2008	5	30	151	115	34.2	76	48	82	69

3074	south-coast	2008	5	31	152	123	23.1	77	49	82	73.8
3075	south-coast	2008	6	1	153	135	24.8	90	50	85	74
3076	south-coast	2008	6	2	154	111	24.8	76	52	83	76
3077	south-coast	2008	6	3	155	117	30.3	80	52	80	84.9
3078	south-coast	2008	6	4	156	132	22.1	66	55	74	99
3079	south-coast	2008	6	5	157	140	25.1	75	53	87	71.1
3080	south-coast	2008	6	6	158	142	25.7	96	54	90	67.3
3081	south-coast	2008	6	7	159	113	27	99	55	88	76.7
3082	south-coast	2008	6	8	160	123	30.9	95	54	93	80.1
3083	south-coast	2008	6	9	161	147	37.8	101	55	98	79
3084	south-coast	2008	6	10	162	135	42	101	54	92	87.4
3085	south-coast	2008	6	11	163	125	35.5	90	58	88	82.4
3086	south-coast	2008	6	12	164	118	37.4	98	57	90	81.8
3087	south-coast	2008	6	13	165	159	37.9	95	57	96	80.5
3088	south-coast	2008	6	14	166	127	37.5	108	58	96	82.3
3089	south-coast	2008	6	15	167	121	35.1	118	59	99	78.3
3090	south-coast	2008	6	16	168	134	38.6	99	60	102	73.6
3091	south-coast	2008	6	17	169	128	44.7	105	62	103	73.1
3092	south-coast	2008	6	18	170	139	35	114	61	104	69.8
3093	south-coast	2008	6	19	171	131	32.1	112	60	107	71.1
3094	south-coast	2008	6	20	172	132	28.7	111	63	109	69.8
3095	south-coast	2008	6	21	173	125	27.6	117	68	108	56.7
3096	south-coast	2008	6	22	174	109	27.6	111	68	105	62.4
3097	south-coast	2008	6	23	175	117	29.1	102	63	100	68.6
3098	south-coast	2008	6	24	176	124	31.7	93	60	96	76.2
3099	south-coast	2008	6	25	177	138	42.9	106	59	93	83
3100	south-coast	2008	6	26	178	131	47.5	95	59	90	81.5
3101	south-coast	2008	6	27	179	130	47.8	120	58	91	81.3
3102	south-coast	2008	6	28	180	123	45.3	113	58	96	79.5
3103	south-coast	2008	6	29	181	124	34	93	61	99	75.4
3104	south-coast	2008	6	30	182	120	25.4	84	61	100	74
3105	south-coast	2008	7	1	183	118	21.8	80	62	102	73.1
3106	south-coast	2008	7	2	184	125	35	103	60	102	74.5
3107	south-coast	2008	7	3	185	113	38	118	62	102	78.1
3108	south-coast	2008	7	4	186	147	54.6	124	62	105	70.4
3109	south-coast	2008	7	5	187	120	77.6	104	64	102	71.4
3110	south-coast	2008	7	6	188	100	41.5	99	62	99	84.7
3111	south-coast	2008	7	7	189	113	52.6	110	61	101	83.8
3112	south-coast	2008	7	8	190	117	45.6	120	64	99	85.3
3113	south-coast	2008	7	9	191	122	38.5	108	62	96	86.5
3114	south-coast	2008	7	10	192	131	39.7	90	64	98	83.9
3115	south-coast	2008	7	11	193	113	26.2	75	65	88	78.9
3116	south-coast	2008	7	12	194	135	25.9	88	64	96	78.3
3117	south-coast	2008	7	13	195	114	26.3	80	66	96	81.8
3118	south-coast	2008	7	14	196	124	27.9	74	64	96	73.7
3119	south-coast	2008	7	15	197	131	26.2	92	66	94	71.2
3120	south-coast	2008	7	16	198	133	27.8	95	66	96	73.3
3121	south-coast	2008	7	17	199	124	34.8	126	66	96	77.4
3122	south-coast	2008	7	18	200	126	44.9	122	64	95	85.7

3123	south-coast	2008	7	19	201	124	39.9	101	63	94	87.6
3124	south-coast	2008	7	20	202	136	27.3	82	62	86	85
3125	south-coast	2008	7	21	203	118	29.9	83	61	94	78.3
3126	south-coast	2008	7	22	204	109	31.1	85	61	94	80.9
3127	south-coast	2008	7	23	205	109	44.9	94	59	93	77.1
3128	south-coast	2008	7	24	206	129	40.8	118	59	98	80.3
3129	south-coast	2008	7	25	207	123	45.7	92	63	100	80.3
3130	south-coast	2008	7	26	208	115	34.7	101	64	95	81.5
3131	south-coast	2008	7	27	209	109	28	83	63	89	83.1
3132	south-coast	2008	7	28	210	111	33	90	63	89	83
3133	south-coast	2008	7	29	211	113	33.3	100	61	91	82.4
3134	south-coast	2008	7	30	212	108	30.5	107	62	94	81
3135	south-coast	2008	7	31	213	102	38.3	99	62	96	81.1
3136	south-coast	2008	8	1	214	121	33	112	61	98	83.8
3137	south-coast	2008	8	2	215	117	27.3	131	64	99	84.3
3138	south-coast	2008	8	3	216	120	19.8	101	64	97	80.3
3139	south-coast	2008	8	4	217	110	20.6	76	68	98	77.1
3140	south-coast	2008	8	5	218	118	16.1	79	67	97	75.1
3141	south-coast	2008	8	6	219	125	19.1	78	66	99	72.5
3142	south-coast	2008	8	7	220	124	21	78	67	100	72
3143	south-coast	2008	8	8	221	140	21.9	81	67	97	71.6
3144	south-coast	2008	8	9	222	143	15.3	89	65	93	76.1
3145	south-coast	2008	8	10	223	128	15.3	87	62	94	77.7
3146	south-coast	2008	8	11	224	107	20	93	59	94	80
3147	south-coast	2008	8	12	225	139	22.8	103	60	97	79.7
3148	south-coast	2008	8	13	226	142	27	103	61	99	75.1
3149	south-coast	2008	8	14	227	105	32.6	115	66	100	77.7
3150	south-coast	2008	8	15	228	131	22.3	117	66	94	80.4
3151	south-coast	2008	8	16	229	104	33.1	83	63	94	77.1
3152	south-coast	2008	8	17	230	118	21.3	85	62	93	79.1
3153	south-coast	2008	8	18	231	122	21.2	74	62	91	78.4
3154	south-coast	2008	8	19	232	102	17.8	63	60	91	80.9
3155	south-coast	2008	8	20	233	114	16.1	74	57	92	79.5
3156	south-coast	2008	8	21	234	111	16.9	101	61	92	83.4
3157	south-coast	2008	8	22	235	105	18	92	61	92	81.8
3158	south-coast	2008	8	23	236	123	22.6	101	61	96	84.6
3159	south-coast	2008	8	24	237	110	20.5	105	62	104	80.7
3160	south-coast	2008	8	25	238	111	19	86	65	103	80.6
3161	south-coast	2008	8	26	239	129	20.1	85	67	97	85.3
3162	south-coast	2008	8	27	240	119	30.6	84	61	96	82.8
3163	south-coast	2008	8	28	241	107	32.5	119	64	96	86.9
3164	south-coast	2008	8	29	242	102	23.2	80	64	102	78.8
3165	south-coast	2008	8	30	243	126	24.2	83	68	96	79.3
3166	south-coast	2008	8	31	244	144	17.8	64	67	91	85.4
3167	south-coast	2008	9	1	245	135	15	66	62	93	82.5
3168	south-coast	2008	9	2	246	98	16.4	73	62	99	83
3169	south-coast	2008	9	3	247	100	17.2	75	64	100	81
3170	south-coast	2008	9	4	248	109	16.8	81	65	99	77.5
3171	south-coast	2008	9	5	249	130	21.2	82	66	101	77.9

3172	south-coast	2008	9	6	250	127	25.1	108	64	99	82.6
3173	south-coast	2008	9	7	251	122	24.1	103	66	99	85.3
3174	south-coast	2008	9	8	252	124	23.5	73	63	94	81.6
3175	south-coast	2008	9	9	253	113	24.1	59	60	94	77.3
3176	south-coast	2008	9	10	254	107	22.1	70	61	87	76.9
3177	south-coast	2008	9	11	255	115	28.6	67	57	85	85.3
3178	south-coast	2008	9	12	256	121	44.5	76	59	79	91.1
3179	south-coast	2008	9	13	257	118	53.3	98	61	86	85.9
3180	south-coast	2008	9	14	258	141	41	113	57	92	86.6
3181	south-coast	2008	9	15	259	127	31	84	59	103	74.5
3182	south-coast	2008	9	16	260	120	20.6	78	66	97	89.3
3183	south-coast	2008	9	17	261	127	23.2	65	63	94	83.9
3184	south-coast	2008	9	18	262	112	22.7	69	65	94	76.4
3185	south-coast	2008	9	19	263	120	17.8	62	62	90	71.2
3186	south-coast	2008	9	20	264	130	9.6	64	58	85	80.1
3187	south-coast	2008	9	21	265	107	14.9	64	53	82	81.5
3188	south-coast	2008	9	22	266	136	19.9	65	54	85	82.3
3189	south-coast	2008	9	23	267	133	24	66	56	88	83.8
3190	south-coast	2008	9	24	268	111	20.3	77	55	100	79.2
3191	south-coast	2008	9	25	269	116	14.6	59	64	100	75.4
3192	south-coast	2008	9	26	270	130	15.8	61	61	97	80
3193	south-coast	2008	9	27	271	119	19.2	73	61	96	86.3
3194	south-coast	2008	9	28	272	113	22.1	76	61	95	86.3
3195	south-coast	2008	9	29	273	118	17.4	59	61	96	75.7
3196	south-coast	2008	9	30	274	116	17.3	66	65	102	55.6
3197	south-coast	2008	10	1	275	128	15.8	73	65	101	51
3198	south-coast	2008	10	2	276	137	15.6	70	66	97	71.9
3199	south-coast	2008	10	3	277	121	13.5	41	58	86	88.1
3200	south-coast	2008	10	4	278	125	12	37	57	75	98.5
3201	south-coast	2008	10	5	279	113	15.9	56	58	77	88.2
3202	south-coast	2008	10	6	280	123	15	63	53	92	68.8
3203	south-coast	2008	10	7	281	115	13	64	49	97	45
3204	south-coast	2008	10	8	282	131	15.5	66	52	99	45.5
3205	south-coast	2008	10	9	283	121	27.5	78	57	95	88.3
3206	south-coast	2008	10	10	284	103	23.1	58	58	79	90.3
3207	south-coast	2008	10	11	285	120	10.2	47	45	71	56.5
3208	south-coast	2008	10	12	286	124	9.7	48	41	73	35.2
3209	south-coast	2008	10	13	287	126	17.2	44	41	78	31.2
3210	south-coast	2008	10	14	288	122	20.8	54	49	89	42
3211	south-coast	2008	10	15	289	143	18.9	50	46	93	34.5
3212	south-coast	2008	10	16	290	123	15.5	64	51	94	45.5
3213	south-coast	2008	10	17	291	151	16.7	63	53	95	53.6
3214	south-coast	2008	10	18	292	119	18.3	67	53	90	77.3
3215	south-coast	2008	10	19	293	119	20	63	51	83	85.2
3216	south-coast	2008	10	20	294	127	31.6	57	49	79	86.4
3217	south-coast	2008	10	21	295	118	32.2	59	52	94	79.3
3218	south-coast	2008	10	22	296	122	13.3	52	51	96	49.6
3219	south-coast	2008	10	23	297	117	11.3	57	50	97	20.8
3220	south-coast	2008	10	24	298	117	17.7	62	51	91	43

3221	south-coast	2008	10	25	299	119	24.2	74	51	94	65.9
3222	south-coast	2008	10	26	300	134	38.1	86	51	92	81.8
3223	south-coast	2008	10	27	301	131	46.4	68	51	96	77.8
3224	south-coast	2008	10	28	302	140	21.3	64	56	96	70.9
3225	south-coast	2008	10	29	303	137	23.6	85	57	94	77
3226	south-coast	2008	10	30	304	120	18.2	61	56	90	81.1
3227	south-coast	2008	10	31	305	142	14.7	42	60	85	77.1
3228	south-coast	2008	11	1	306	138	13.6	36	56	78	81.7
3229	south-coast	2008	11	2	307	144	14.1	40	56	75	92
3230	south-coast	2008	11	3	308	138	13.2	47	52	69	92.8
3231	south-coast	2008	11	4	309	119	12.2	40	53	68	95.5
3232	south-coast	2008	11	5	310	108	10.3	46	45	75	64.8
3233	south-coast	2008	11	6	311	113	8.8	41	44	82	36.8
3234	south-coast	2008	11	7	312	125	12	41	50	85	41.4
3235	south-coast	2008	11	8	313	120	14.8	45	47	84	50.2
3236	south-coast	2008	11	9	314	121	11.6	42	52	83	89.9
3237	south-coast	2008	11	10	315	130	12.3	42	43	67	86.2
3238	south-coast	2008	11	11	316	134	21.4	43	43	72	80.1
3239	south-coast	2008	11	12	317	117	20.5	36	38	86	72.8
3240	south-coast	2008	11	13	318	143	32.9	34	51	89	70.1
3241	south-coast	2008	11	14	319	130	28.7	47	47	91	61.8
3242	south-coast	2008	11	15	320	123	39.9	48	51	90	33.2
3243	south-coast	2008	11	16	321	130	78.3	84	48	89	29.1
3244	south-coast	2008	11	17	322	125	41.8	47	49	90	40.1
3245	south-coast	2008	11	18	323	141	31.8	43	50	90	49.8
3246	south-coast	2008	11	19	324	127	34	52	52	87	78.2
3247	south-coast	2008	11	20	325	104	50.9	45	51	82	86.4
3248	south-coast	2008	11	21	326	114	38.2	55	50	85	64.8
3249	south-coast	2008	11	22	327	143	38.9	54	52	82	76.5
3250	south-coast	2008	11	23	328	114	53.1	56	47	75	93.7
3251	south-coast	2008	11	24	329	132	26.8	53	46	77	82.4
3252	south-coast	2008	11	25	330	146	19.6	46	50	75	82.5
3253	south-coast	2008	11	26	331	128	11.4	33	49	70	98.7
3254	south-coast	2008	11	27	332	134	12.9	37	51	66	99.6
3255	south-coast	2008	11	28	333	130	26.4	38	47	64	99.1
3256	south-coast	2008	11	29	334	140	40.6	41	45	77	89
3257	south-coast	2008	11	30	335	122	33.3	41	48	84	89.1
3258	south-coast	2008	12	1	336	108	28.5	40	49	85	89.5
3259	south-coast	2008	12	2	337	126	57.8	40	45	74	98.6
3260	south-coast	2008	12	3	338	125	59.9	50	42	72	89.9
3261	south-coast	2008	12	4	339	129	57.7	45	46	68	90.5
3262	south-coast	2008	12	5	340	110	31.9	45	41	75	72.4
3263	south-coast	2008	12	6	341	127	12.7	47	41	77	52.3
3264	south-coast	2008	12	7	342	127	17.9	40	51	71	90.8
3265	south-coast	2008	12	8	343	140	20.7	42	50	62	99
3266	south-coast	2008	12	9	344	110	13.8	42	40	72	69.2
3267	south-coast	2008	12	10	345	142	10.7	40	37	74	42.5
3268	south-coast	2008	12	11	346	121	20.1	30	44	83	46.7
3269	south-coast	2008	12	12	347	148	27.1	34	46	72	94

3270	south-coast	2008	12	13	348	138	13.6	41	46	63	99
3271	south-coast	2008	12	14	349	121	16.4	38	32	58	72.7
3272	south-coast	2008	12	15	350	141	6.3	39	41	58	99.7
3273	south-coast	2008	12	16	351	140	14.8	40	40	58	92.3
3274	south-coast	2008	12	17	352	150	7.6	38	38	56	99.2
3275	south-coast	2008	12	18	353	137	12.2	38	35	57	97.4
3276	south-coast	2008	12	19	354	130	20.8	41	32	58	91.7
3277	south-coast	2008	12	20	355	149	33.3	45	32	59	86.5
3278	south-coast	2008	12	21	356	129	24.2	42	35	66	80.2
3279	south-coast	2008	12	22	357	162	23.5	38	41	63	98.4
3280	south-coast	2008	12	23	358	140	14.5	37	39	58	95.7
3281	south-coast	2008	12	24	359	133	15.2	38	40	56	89.8
3282	south-coast	2008	12	25	360	168	7	40	44	57	100
3283	south-coast	2008	12	26	361	131	13.7	38	31	57	78.3
3284	south-coast	2008	12	27	362	143	15.8	40	30	60	51.7
3285	south-coast	2008	12	28	363	141	12.6	39	33	66	63.5
3286	south-coast	2008	12	29	364	137	21.1	35	38	75	82.4
3287	south-coast	2008	12	30	365	161	21.7	36	41	76	82.1
3288	south-coast	2008	12	31	366	153	45.5	40	41	73	93
3289	south-coast	2009	1	1	1	153	74.1	39	39	76	93.8
3290	south-coast	2009	1	2	2	155	63.9	58	39	67	100
3291	south-coast	2009	1	3	3	163	43.1	36	45	59	91.5
3292	south-coast	2009	1	4	4	135	22.3	43	41	61	68
3293	south-coast	2009	1	5	5	146	21.5	42	30	60	61.2
3294	south-coast	2009	1	6	6	136	34.5	31	30	64	82.2
3295	south-coast	2009	1	7	7	131	40.7	27	39	72	80.3
3296	south-coast	2009	1	8	8	163	48	32	42	74	92.2
3297	south-coast	2009	1	9	9	150	46.2	46	43	75	76.2
3298	south-coast	2009	1	10	10	152	26.3	46	36	75	45.9
3299	south-coast	2009	1	11	11	158	18.3	47	47	84	33.2
3300	south-coast	2009	1	12	12	164	13	44	44	85	30.9
3301	south-coast	2009	1	13	13	146	18	40	47	84	37.1
3302	south-coast	2009	1	14	14	119	13.4	45	36	87	38.3
3303	south-coast	2009	1	15	15	144	18.1	41	44	83	33.5
3304	south-coast	2009	1	16	16	154	22.6	43	42	80	39
3305	south-coast	2009	1	17	17	164	16.1	44	41	81	55.2
3306	south-coast	2009	1	18	18	141	15.5	45	42	80	54.3
3307	south-coast	2009	1	19	19	160	29.4	45	42	80	54.9
3308	south-coast	2009	1	20	20	157	14.9	46	47	81	52
3309	south-coast	2009	1	21	21	180	19.4	43	53	81	73.5
3310	south-coast	2009	1	22	22	141	23.9	41	50	73	75.8
3311	south-coast	2009	1	23	23	138	15	29	53	66	98.6
3312	south-coast	2009	1	24	24	160	10.6	34	49	62	100
3313	south-coast	2009	1	25	25	144	11.7	40	47	61	100
3314	south-coast	2009	1	26	26	133	7.5	42	40	59	87.6
3315	south-coast	2009	1	27	27	125	11.7	43	32	65	55.7
3316	south-coast	2009	1	28	28	143	14.9	44	35	75	47.4
3317	south-coast	2009	1	29	29	132	14.1	45	42	80	43.3
3318	south-coast	2009	1	30	30	158	13.2	44	41	80	38.3

3319	south-coast	2009	1	31	31	141	26.7	44	42	79	56.4
3320	south-coast	2009	2	1	32	130	26	45	40	81	100
3321	south-coast	2009	2	2	33	154	15.3	47	41	81	36.6
3322	south-coast	2009	2	3	34	151	16.1	47	43	82	45.2
3323	south-coast	2009	2	4	35	155	11.2	48	44	82	36.7
3324	south-coast	2009	2	5	36	156	10.7	47	46	76	85.7
3325	south-coast	2009	2	6	37	144	6.8	43	50	61	100
3326	south-coast	2009	2	7	38	169	6	42	43	58	100
3327	south-coast	2009	2	8	39	146	9.5	42	44	60	100
3328	south-coast	2009	2	9	40	140	6.8	43	36	59	100
3329	south-coast	2009	2	10	41	118	12	42	30	58	96.5
3330	south-coast	2009	2	11	42	135	23.7	44	32	59	86.4
3331	south-coast	2009	2	12	43	154	20	40	40	60	90.3
3332	south-coast	2009	2	13	44	141	11.7	41	35	58	97.8
3333	south-coast	2009	2	14	45	161	15.6	46	38	57	89.8
3334	south-coast	2009	2	15	46	164	23.2	48	35	59	81.3
3335	south-coast	2009	2	16	47	158	8.7	43	42	58	99.9
3336	south-coast	2009	2	17	48	160	8.3	43	38	60	100
3337	south-coast	2009	2	18	49	156	11.8	47	35	66	80
3338	south-coast	2009	2	19	50	127	12.9	48	35	74	76.5
3339	south-coast	2009	2	20	51	158	13	47	38	76	75.8
3340	south-coast	2009	2	21	52	158	27.2	49	43	74	67.5
3341	south-coast	2009	2	22	53	137	37.5	39	53	71	83
3342	south-coast	2009	2	23	54	165	23.4	35	51	74	86.3
3343	south-coast	2009	2	24	55	168	21.4	40	42	72	82.7
3344	south-coast	2009	2	25	56	158	24.7	47	43	69	97.4
3345	south-coast	2009	2	26	57	162	25.6	45	43	67	95
3346	south-coast	2009	2	27	58	135	36.2	48	49	69	88.9
3347	south-coast	2009	2	28	59	162	21.2	56	44	83	71.5
3348	south-coast	2009	3	1	60	156	14.5	66	48	84	57.5
3349	south-coast	2009	3	2	61	158	26.1	36	48	83	70
3350	south-coast	2009	3	3	62	134	14.5	45	48	69	81.9
3351	south-coast	2009	3	4	63	148	12.1	44	40	64	98.3
3352	south-coast	2009	3	5	64	133	12.7	49	43	64	84.3
3353	south-coast	2009	3	6	65	146	12.7	44	35	62	85
3354	south-coast	2009	3	7	66	142	15.6	54	42	67	79.8
3355	south-coast	2009	3	8	67	128	21.8	54	38	67	87.9
3356	south-coast	2009	3	9	68	144	13.5	50	46	63	100
3357	south-coast	2009	3	10	69	147	14.3	60	43	70	79.5
3358	south-coast	2009	3	11	70	147	25.3	55	39	68	93.5
3359	south-coast	2009	3	12	71	157	18.5	53	47	65	92.2
3360	south-coast	2009	3	13	72	140	23.9	53	38	75	84.1
3361	south-coast	2009	3	14	73	130	33.8	60	42	69	93
3362	south-coast	2009	3	15	74	155	18.5	52	41	66	95.1
3363	south-coast	2009	3	16	75	145	22.7	65	40	76	78.6
3364	south-coast	2009	3	17	76	126	39	61	46	83	72.1
3365	south-coast	2009	3	18	77	135	34.1	84	49	86	75
3366	south-coast	2009	3	19	78	148	58.8	77	52	86	89.5
3367	south-coast	2009	3	20	79	167	54.4	62	44	80	90.3

3368	south-coast	2009	3	21	80	132	36	58	51	70	93
3369	south-coast	2009	3	22	81	124	11.9	53	50	62	89.2
3370	south-coast	2009	3	23	82	125	10.5	66	42	69	49.1
3371	south-coast	2009	3	24	83	138	13.9	56	40	77	44.5
3372	south-coast	2009	3	25	84	125	16.8	61	42	80	52.4
3373	south-coast	2009	3	26	85	138	32.4	63	43	77	89.3
3374	south-coast	2009	3	27	86	152	20.1	60	44	79	70.7
3375	south-coast	2009	3	28	87	137	15.8	69	44	83	60.7
3376	south-coast	2009	3	29	88	148	49.1	69	44	82	94.7
3377	south-coast	2009	3	30	89	106	15.6	58	47	75	70.7
3378	south-coast	2009	3	31	90	143	19.3	66	42	77	73.4
3379	south-coast	2009	4	1	91	136	24	68	43	73	84.1
3380	south-coast	2009	4	2	92	154	24.2	69	46	71	92.4
3381	south-coast	2009	4	3	93	143	16.8	58	50	66	94.7
3382	south-coast	2009	4	4	94	131	16.6	64	39	74	61.8
3383	south-coast	2009	4	5	95	150	11.6	66	40	81	38.8
3384	south-coast	2009	4	6	96	149	12.9	64	40	88	47.9
3385	south-coast	2009	4	7	97	147	15.2	65	45	86	82.7
3386	south-coast	2009	4	8	98	110	9.7	54	48	70	91.7
3387	south-coast	2009	4	9	99	125	13.6	56	44	68	92
3388	south-coast	2009	4	10	100	130	14.6	52	49	64	100
3389	south-coast	2009	4	11	101	107	14.2	54	47	66	98.5
3390	south-coast	2009	4	12	102	135	17.3	64	43	78	78.8
3391	south-coast	2009	4	13	103	138	22.2	66	43	82	78.8
3392	south-coast	2009	4	14	104	134	20	59	52	79	77.6
3393	south-coast	2009	4	15	105	118	9.8	52	40	62	69.6
3394	south-coast	2009	4	16	106	133	14	57	38	69	73.9
3395	south-coast	2009	4	17	107	135	16.9	64	41	80	66.3
3396	south-coast	2009	4	18	108	153	20.7	80	46	91	61.3
3397	south-coast	2009	4	19	109	167	17	76	52	96	47.2
3398	south-coast	2009	4	20	110	158	13.1	74	57	101	33.6
3399	south-coast	2009	4	21	111	155	16.9	95	60	100	47.5
3400	south-coast	2009	4	22	112	147	26.1	81	54	96	91
3401	south-coast	2009	4	23	113	139	28.6	65	55	75	91.7
3402	south-coast	2009	4	24	114	121	20.6	58	54	72	90.9
3403	south-coast	2009	4	25	115	154	12	60	52	71	83.3
3404	south-coast	2009	4	26	116	132	17.8	67	41	72	76.5
3405	south-coast	2009	4	27	117	133	20.8	63	41	72	84.8
3406	south-coast	2009	4	28	118	132	18.8	63	51	68	89.3
3407	south-coast	2009	4	29	119	148	23	68	49	72	83.5
3408	south-coast	2009	4	30	120	111	29.6	85	45	80	74.7
3409	south-coast	2009	5	1	121	148	26	62	52	79	71.6
3410	south-coast	2009	5	2	122	132	13.3	49	58	80	90.6
3411	south-coast	2009	5	3	123	138	20.5	68	52	81	83.4
3412	south-coast	2009	5	4	124	126	16.7	72	52	81	79
3413	south-coast	2009	5	5	125	134	16.2	62	48	84	81.1
3414	south-coast	2009	5	6	126	131	31.4	85	52	91	74.5
3415	south-coast	2009	5	7	127	125	24	95	61	95	68.1
3416	south-coast	2009	5	8	128	148	23.3	97	57	94	87.5

3417	south-coast	2009	5	9	129	134	33.4	103	56	89	89.1
3418	south-coast	2009	5	10	130	110	33.4	102	54	86	89.6
3419	south-coast	2009	5	11	131	118	42	94	54	86	88.2
3420	south-coast	2009	5	12	132	121	33	86	56	84	88
3421	south-coast	2009	5	13	133	140	41	94	53	83	83.9
3422	south-coast	2009	5	14	134	140	37.7	94	53	88	86.7
3423	south-coast	2009	5	15	135	135	35	87	55	91	89
3424	south-coast	2009	5	16	136	133	38.4	97	55	96	88
3425	south-coast	2009	5	17	137	137	37.3	103	59	96	89.7
3426	south-coast	2009	5	18	138	146	33.3	85	59	95	86.3
3427	south-coast	2009	5	19	139	131	23.2	67	59	92	87.3
3428	south-coast	2009	5	20	140	131	21.9	63	57	92	80.2
3429	south-coast	2009	5	21	141	121	24.6	66	49	87	85
3430	south-coast	2009	5	22	142	132	26	64	55	83	80.8
3431	south-coast	2009	5	23	143	120	26.2	77	54	84	79
3432	south-coast	2009	5	24	144	124	25.2	77	54	82	80.8
3433	south-coast	2009	5	25	145	129	23.5	79	54	77	83.2
3434	south-coast	2009	5	26	146	110	28.1	82	56	81	84.2
3435	south-coast	2009	5	27	147	128	37.8	87	55	87	84
3436	south-coast	2009	5	28	148	130	49.2	96	57	86	85.1
3437	south-coast	2009	5	29	149	109	48.5	70	60	77	96
3438	south-coast	2009	5	30	150	112	49.8	83	58	76	95.8
3439	south-coast	2009	5	31	151	121	36.3	90	57	80	94
3440	south-coast	2009	6	1	152	108	32.8	82	57	81	91.7
3441	south-coast	2009	6	2	153	160	35.2	71	59	89	85
3442	south-coast	2009	6	3	154	116	32.8	45	57	82	94.5
3443	south-coast	2009	6	4	155	91	18.1	47	57	76	83.9
3444	south-coast	2009	6	5	156	133	8.4	40	54	76	93.2
3445	south-coast	2009	6	6	157	132	10.1	41	54	71	93.3
3446	south-coast	2009	6	7	158	129	11.5	52	56	75	89.2
3447	south-coast	2009	6	8	159	131	15.5	51	55	76	87.7
3448	south-coast	2009	6	9	160	140	18.3	43	55	71	96.8
3449	south-coast	2009	6	10	161	122	18.9	43	57	67	99.9
3450	south-coast	2009	6	11	162	115	16.1	42	56	67	94.5
3451	south-coast	2009	6	12	163	143	17.8	48	56	68	97.8
3452	south-coast	2009	6	13	164	126	18	50	56	71	97.3
3453	south-coast	2009	6	14	165	104	14.1	48	53	78	87.5
3454	south-coast	2009	6	15	166	116	14.5	44	53	75	90.9
3455	south-coast	2009	6	16	167	125	18	50	58	80	85.8
3456	south-coast	2009	6	17	168	136	21.6	69	57	83	83.4
3457	south-coast	2009	6	18	169	133	30.1	87	56	89	86.3
3458	south-coast	2009	6	19	170	132	33.4	101	60	92	88.3
3459	south-coast	2009	6	20	171	125	22.1	63	60	91	94.8
3460	south-coast	2009	6	21	172	110	18.8	65	52	91	80.4
3461	south-coast	2009	6	22	173	135	24.3	85	52	86	81.9
3462	south-coast	2009	6	23	174	109	26.2	95	54	88	82
3463	south-coast	2009	6	24	175	111	30.5	97	57	87	85.7
3464	south-coast	2009	6	25	176	132	30.3	99	56	90	88.2
3465	south-coast	2009	6	26	177	147	30.4	92	55	92	83.8

3466	south-coast	2009	6	27	178	144	31	104	59	99	82.8
3467	south-coast	2009	6	28	179	132	32.8	104	62	103	84.2
3468	south-coast	2009	6	29	180	130	29.8	81	61	96	86.8
3469	south-coast	2009	6	30	181	101	28.3	98	60	95	88.7
3470	south-coast	2009	7	1	182	112	26.7	83	61	95	84.5
3471	south-coast	2009	7	2	183	139	26.4	70	51	96	85.1
3472	south-coast	2009	7	3	184	96	33	94	53	95	87.4
3473	south-coast	2009	7	4	185	106	31.1	67	53	97	81.4
3474	south-coast	2009	7	5	186	119	75.9	74	50	99	80.3
3475	south-coast	2009	7	6	187	111	25.4	82	58	95	78.7
3476	south-coast	2009	7	7	188	116	27.5	84	58	94	84.3
3477	south-coast	2009	7	8	189	98	28.3	83	57	93	83.6
3478	south-coast	2009	7	9	190	127	26.4	85	55	95	80
3479	south-coast	2009	7	10	191	110	25.5	90	57	98	72.4
3480	south-coast	2009	7	11	192	123	22.9	93	53	100	70
3481	south-coast	2009	7	12	193	102	22.6	91	53	100	71.5
3482	south-coast	2009	7	13	194	112	20.8	75	53	101	70.8
3483	south-coast	2009	7	14	195	137	27	91	62	97	78.9
3484	south-coast	2009	7	15	196	132	34.2	99	64	100	82.3
3485	south-coast	2009	7	16	197	114	28.6	90	64	101	78.3
3486	south-coast	2009	7	17	198	119	22.3	110	64	100	78.8
3487	south-coast	2009	7	18	199	122	30.8	128	64	106	74.2
3488	south-coast	2009	7	19	200	114	28.5	109	66	106	74.6
3489	south-coast	2009	7	20	201	110	27.3	83	67	103	73.9
3490	south-coast	2009	7	21	202	143	26	105	67	102	74
3491	south-coast	2009	7	22	203	131	28.1	100	67	101	73.8
3492	south-coast	2009	7	23	204	137	29.5	100	67	99	82.7
3493	south-coast	2009	7	24	205	140	31.8	111	66	95	84.7
3494	south-coast	2009	7	25	206	129	26.5	99	65	97	84.2
3495	south-coast	2009	7	26	207	109	23.9	86	67	95	83.1
3496	south-coast	2009	7	27	208	158	22.7	102	67	100	84.1
3497	south-coast	2009	7	28	209	124	22.4	101	64	97	84.4
3498	south-coast	2009	7	29	210	125	23	99	63	94	84.8
3499	south-coast	2009	7	30	211	118	27.8	87	62	92	83
3500	south-coast	2009	7	31	212	133	28.3	108	63	96	83.8
3501	south-coast	2009	8	1	213	128	29.7	108	63	94	84.8
3502	south-coast	2009	8	2	214	114	25.7	95	62	95	84.8
3503	south-coast	2009	8	3	215	104	15.1	71	62	98	70.6
3504	south-coast	2009	8	4	216	147	11.2	79	66	102	65.9
3505	south-coast	2009	8	5	217	116	16.1	60	64	101	61
3506	south-coast	2009	8	6	218	133	10.1	53	59	91	69.8
3507	south-coast	2009	8	7	219	109	11.9	59	56	83	78.8
3508	south-coast	2009	8	8	220	108	18.1	85	58	88	77.5
3509	south-coast	2009	8	9	221	119	26.6	100	58	91	80.6
3510	south-coast	2009	8	10	222	129	32.4	90	58	94	84
3511	south-coast	2009	8	11	223	114	31.3	101	60	96	86.9
3512	south-coast	2009	8	12	224	117	33.1	103	62	97	87.8
3513	south-coast	2009	8	13	225	144	37.7	84	59	96	98.8
3514	south-coast	2009	8	14	226	129	32.1	81	59	91	80.7

3515	south-coast	2009	8	15	227	129	39.4	97	60	89	82.5
3516	south-coast	2009	8	16	228	111	40.5	97	56	89	83.9
3517	south-coast	2009	8	17	229	125	42.5	101	56	92	88.2
3518	south-coast	2009	8	18	230	105	46.4	105	57	92	89.3
3519	south-coast	2009	8	19	231	120	45.6	104	58	91	87.5
3520	south-coast	2009	8	20	232	126	50.2	108	59	95	87.6
3521	south-coast	2009	8	21	233	104	34.8	84	62	99	81.4
3522	south-coast	2009	8	22	234	128	25.7	77	68	95	85.3
3523	south-coast	2009	8	23	235	122	26.5	74	62	94	87.3
3524	south-coast	2009	8	24	236	117	22.9	72	62	96	78
3525	south-coast	2009	8	25	237	106	16.1	71	61	99	70.3
3526	south-coast	2009	8	26	238	116	82.9	95	60	102	57.4
3527	south-coast	2009	8	27	239	119	66.7	89	62	107	46.6
3528	south-coast	2009	8	28	240	130	27.1	101	65	107	70.1
3529	south-coast	2009	8	29	241	121	24.5	104	66	108	69.9
3530	south-coast	2009	8	30	242	129	41.4	118	64	106	68.1
3531	south-coast	2009	8	31	243	113	66.2	108	64	104	68.3
3532	south-coast	2009	9	1	244	118	28.8	78	70	103	80.5
3533	south-coast	2009	9	2	245	130	37.2	93	68	103	76.9
3534	south-coast	2009	9	3	246	146	36.6	104	68	100	72.1
3535	south-coast	2009	9	4	247	119	33.8	107	68	100	81
3536	south-coast	2009	9	5	248	131	27.6	82	66	99	74.2
3537	south-coast	2009	9	6	249	111	25.9	86	56	91	80.3
3538	south-coast	2009	9	7	250	121	21.8	80	57	96	83.4
3539	south-coast	2009	9	8	251	122	19.9	74	56	91	98.3
3540	south-coast	2009	9	9	252	101	26.5	73	57	95	83.4
3541	south-coast	2009	9	10	253	148	27.1	76	62	100	73.4
3542	south-coast	2009	9	11	254	131	22.2	91	65	96	78.9
3543	south-coast	2009	9	12	255	109	25.8	76	64	93	88.7
3544	south-coast	2009	9	13	256	113	26.6	61	61	90	87.5
3545	south-coast	2009	9	14	257	130	14.4	46	61	78	93.4
3546	south-coast	2009	9	15	258	97	15.6	59	58	84	83.3
3547	south-coast	2009	9	16	259	134	22.7	81	57	93	80.2
3548	south-coast	2009	9	17	260	126	28	87	60	99	81.3
3549	south-coast	2009	9	18	261	117	31	91	63	102	86.2
3550	south-coast	2009	9	19	262	108	35.1	94	63	100	87
3551	south-coast	2009	9	20	263	106	30	86	59	94	84.8
3552	south-coast	2009	9	21	264	122	25.6	76	59	95	88.7
3553	south-coast	2009	9	22	265	114	23.2	72	60	106	86.5
3554	south-coast	2009	9	23	266	132	14.5	60	61	102	75.3
3555	south-coast	2009	9	24	267	104	18.6	65	62	103	55.7
3556	south-coast	2009	9	25	268	128	23.7	92	62	106	70.6
3557	south-coast	2009	9	26	269	119	26.4	91	64	108	81.4
3558	south-coast	2009	9	27	270	104	32.4	105	58	100	90.6
3559	south-coast	2009	9	28	271	131	48.2	74	58	93	89.3
3560	south-coast	2009	9	29	272	125	37.9	61	55	84	87.2
3561	south-coast	2009	9	30	273	114	24.7	60	58	86	70.6
3562	south-coast	2009	10	1	274	146	12.6	58	51	92	35.6
3563	south-coast	2009	10	2	275	124	13.5	65	52	93	62.3

3564	south-coast	2009	10	3	276	136	15.3	76	51	86	79.7
3565	south-coast	2009	10	4	277	104	12.3	49	55	79	93
3566	south-coast	2009	10	5	278	117	13	49	44	71	80.3
3567	south-coast	2009	10	6	279	139	17.3	51	44	76	81.3
3568	south-coast	2009	10	7	280	128	20.8	55	54	73	89.5
3569	south-coast	2009	10	8	281	126	24.5	63	49	76	85
3570	south-coast	2009	10	9	282	129	28.8	67	48	78	86.6
3571	south-coast	2009	10	10	283	127	37	73	48	79	92
3572	south-coast	2009	10	11	284	134	22.6	59	51	76	87.5
3573	south-coast	2009	10	12	285	127	13.8	46	51	69	99
3574	south-coast	2009	10	13	286	128	8.9	30	56	73	100
3575	south-coast	2009	10	14	287	138	10.1	26	58	74	100
3576	south-coast	2009	10	15	288	149	27.5	47	53	91	91.2
3577	south-coast	2009	10	16	289	130	30.6	51	60	100	65.6
3578	south-coast	2009	10	17	290	145	25.7	72	60	98	79.5
3579	south-coast	2009	10	18	291	126	33.1	83	57	94	95.4
3580	south-coast	2009	10	19	292	130	26.8	64	57	81	91.5
3581	south-coast	2009	10	20	293	121	13.3	45	53	79	80
3582	south-coast	2009	10	21	294	118	14.1	49	50	86	69.1
3583	south-coast	2009	10	22	295	134	19.5	62	51	89	77.3
3584	south-coast	2009	10	23	296	103	24.2	54	52	92	80.6
3585	south-coast	2009	10	24	297	110	32.6	75	59	87	86.1
3586	south-coast	2009	10	25	298	129	45.3	81	58	88	83.7
3587	south-coast	2009	10	26	299	110	13	48	53	91	46.1
3588	south-coast	2009	10	27	300	135	18	49	51	90	51
3589	south-coast	2009	10	28	301	116	10.3	45	47	71	37.3
3590	south-coast	2009	10	29	302	141	11	41	46	71	33.2
3591	south-coast	2009	10	30	303	134	18.2	40	44	77	57.6
3592	south-coast	2009	10	31	304	150	20.7	43	47	89	67.4
3593	south-coast	2009	11	1	305	160	18.5	57	49	93	61.5
3594	south-coast	2009	11	2	306	154	27.7	56	49	94	73
3595	south-coast	2009	11	3	307	141	34.5	60	52	94	84
3596	south-coast	2009	11	4	308	145	36	48	50	86	94.4
3597	south-coast	2009	11	5	309	141	41.4	47	46	74	91.1
3598	south-coast	2009	11	6	310	122	38.7	53	54	74	89.3
3599	south-coast	2009	11	7	311	135	44.1	55	46	74	93.8
3600	south-coast	2009	11	8	312	136	53.4	61	44	80	90.8
3601	south-coast	2009	11	9	313	150	37.5	51	44	85	86.6
3602	south-coast	2009	11	10	314	126	20.8	45	49	86	73.6
3603	south-coast	2009	11	11	315	106	23	44	56	81	78
3604	south-coast	2009	11	12	316	133	24.5	49	50	72	89.3
3605	south-coast	2009	11	13	317	157	19.3	43	51	69	97.9
3606	south-coast	2009	11	14	318	136	26.6	48	43	68	89.5
3607	south-coast	2009	11	15	319	125	21.5	50	39	74	58.3
3608	south-coast	2009	11	16	320	128	14.8	50	39	80	33.9
3609	south-coast	2009	11	17	321	126	17.3	47	43	84	49.3
3610	south-coast	2009	11	18	322	128	26.1	53	40	73	86.8
3611	south-coast	2009	11	19	323	126	29.6	50	41	81	76.7
3612	south-coast	2009	11	20	324	122	27.9	59	42	77	91.7

3613	south-coast	2009	11	21	325	131	41.5	61	43	66	93.5
3614	south-coast	2009	11	22	326	137	35.9	55	41	72	83
3615	south-coast	2009	11	23	327	173	28.6	48	41	78	74.5
3616	south-coast	2009	11	24	328	142	19.2	48	40	76	45.8
3617	south-coast	2009	11	25	329	134	21.2	44	40	80	33.8
3618	south-coast	2009	11	26	330	122	17.6	45	41	83	39.4
3619	south-coast	2009	11	27	331	145	21.1	53	41	78	69.4
3620	south-coast	2009	11	28	332	133	12.2	38	43	66	99.3
3621	south-coast	2009	11	29	333	142	10.2	44	43	73	70.6
3622	south-coast	2009	11	30	334	117	15	43	44	78	52.4
3623	south-coast	2009	12	1	335	142	21.7	40	40	73	74.4
3624	south-coast	2009	12	2	336	146	25.5	38	51	65	92.2
3625	south-coast	2009	12	3	337	130	37.1	42	46	72	89.4
3626	south-coast	2009	12	4	338	156	35.7	40	37	70	73.1
3627	south-coast	2009	12	5	339	120	24	45	39	67	88.6
3628	south-coast	2009	12	6	340	146	15.8	46	43	58	100
3629	south-coast	2009	12	7	341	137	9.9	40	43	58	100
3630	south-coast	2009	12	8	342	131	12.2	37	30	58	91.3
3631	south-coast	2009	12	9	343	153	24.6	39	33	57	81.9
3632	south-coast	2009	12	10	344	135	32.1	43	34	62	75
3633	south-coast	2009	12	11	345	165	35	37	43	59	100
3634	south-coast	2009	12	12	346	133	17	41	50	60	100
3635	south-coast	2009	12	13	347	133	9.9	44	48	61	100
3636	south-coast	2009	12	14	348	145	17.1	41	47	62	92.3
3637	south-coast	2009	12	15	349	140	22.8	44	38	69	89.8
3638	south-coast	2009	12	16	350	135	18.6	45	40	72	75.4
3639	south-coast	2009	12	17	351	162	26.4	42	41	74	66.3
3640	south-coast	2009	12	18	352	153	26.4	45	43	79	58.4
3641	south-coast	2009	12	19	353	157	13.3	45	45	78	54.8
3642	south-coast	2009	12	20	354	140	16.4	42	44	73	60.2
3643	south-coast	2009	12	21	355	128	31	46	44	69	70
3644	south-coast	2009	12	22	356	156	14.8	43	44	62	77.3
3645	south-coast	2009	12	23	357	151	12.5	41	42	69	58.5
3646	south-coast	2009	12	24	358	151	37.7	44	36	69	68.6
3647	south-coast	2009	12	25	359	164	46.5	45	36	68	80.4
3648	south-coast	2009	12	26	360	151	47.9	41	36	60	89.9
3649	south-coast	2009	12	27	361	136	40.8	44	34	63	76.7
3650	south-coast	2009	12	28	362	155	23.2	46	34	65	58.6
3651	south-coast	2009	12	29	363	154	18.8	45	42	64	68.5
3652	south-coast	2009	12	30	364	149	31.3	41	38	61	100
3653	south-coast	2009	12	31	365	136	23.2	44	38	68	78.4
3654	south-coast	2010	1	1	1	146	54.2	43	39	71	60.7
3655	south-coast	2010	1	2	2	158	35.4	44	39	79	54.4
3656	south-coast	2010	1	3	3	150	20.3	43	40	78	60.6
3657	south-coast	2010	1	4	4	126	19.2	44	40	75	57.4
3658	south-coast	2010	1	5	5	146	20.5	43	40	77	54.5
3659	south-coast	2010	1	6	6	159	24.3	37	43	76	65.9
3660	south-coast	2010	1	7	7	146	34.5	34	42	77	77
3661	south-coast	2010	1	8	8	165	30.7	35	46	77	70

3662	south-coast	2010	1	9	9	150	15.7	42	44	74	65.2
3663	south-coast	2010	1	10	10	162	21.3	42	45	78	54.8
3664	south-coast	2010	1	11	11	158	25.6	41	45	77	58.9
3665	south-coast	2010	1	12	12	151	28.3	44	48	77	85.8
3666	south-coast	2010	1	13	13	159	36.1	43	43	70	97
3667	south-coast	2010	1	14	14	168	17.8	51	44	71	62.3
3668	south-coast	2010	1	15	15	139	14.7	45	40	72	46.1
3669	south-coast	2010	1	16	16	151	23.6	42	45	70	63.4
3670	south-coast	2010	1	17	17	154	25.6	51	42	66	94.4
3671	south-coast	2010	1	18	18	146	28.8	45	42	61	100
3672	south-coast	2010	1	19	19	140	14.5	45	40	61	99.3
3673	south-coast	2010	1	20	20	130	15.4	43	45	58	100
3674	south-coast	2010	1	21	21	151	9	47	43	60	99
3675	south-coast	2010	1	22	22	135	12.7	44	37	60	99.9
3676	south-coast	2010	1	23	23	120	22.7	69	36	59	99
3677	south-coast	2010	1	24	24	148	28.3	45	33	63	94.3
3678	south-coast	2010	1	25	25	160	27.2	43	33	66	80.7
3679	south-coast	2010	1	26	26	151	30.4	39	38	63	95.8
3680	south-coast	2010	1	27	27	122	23.5	39	45	67	98.2
3681	south-coast	2010	1	28	28	133	15.3	42	43	70	76.3
3682	south-coast	2010	1	29	29	119	25.2	41	42	68	84.9
3683	south-coast	2010	1	30	30	129	36	46	39	65	92.8
3684	south-coast	2010	1	31	31	139	41	52	38	67	89.1
3685	south-coast	2010	2	1	32	152	35.3	49	38	68	88.4
3686	south-coast	2010	2	2	33	138	40.8	51	41	66	91.7
3687	south-coast	2010	2	3	34	132	36.7	46	39	68	89.3
3688	south-coast	2010	2	4	35	143	25.4	48	43	67	97.5
3689	south-coast	2010	2	5	36	145	14.4	47	46	60	100
3690	south-coast	2010	2	6	37	130	9.4	42	51	60	100
3691	south-coast	2010	2	7	38	133	16.7	43	38	63	95.8
3692	south-coast	2010	2	8	39	154	20.6	45	38	64	92.6
3693	south-coast	2010	2	9	40	159	16.2	43	44	62	100
3694	south-coast	2010	2	10	41	143	16.9	40	38	64	100
3695	south-coast	2010	2	11	42	166	25.7	47	37	64	89.5
3696	south-coast	2010	2	12	43	161	42.7	44	47	69	90.1
3697	south-coast	2010	2	13	44	153	31.8	47	41	79	75.9
3698	south-coast	2010	2	14	45	142	18.8	50	41	83	63.5
3699	south-coast	2010	2	15	46	146	17.4	51	41	83	62.4
3700	south-coast	2010	2	16	47	153	15.6	50	45	83	66.1
3701	south-coast	2010	2	17	48	151	21.7	48	48	82	70.8
3702	south-coast	2010	2	18	49	150	44.4	63	45	75	90.5
3703	south-coast	2010	2	19	50	149	23	48	44	66	94.4
3704	south-coast	2010	2	20	51	149	10.6	46	44	64	99
3705	south-coast	2010	2	21	52	126	10.8	42	47	64	100
3706	south-coast	2010	2	22	53	155	8.5	48	47	67	71.4
3707	south-coast	2010	2	23	54	155	11.1	47	34	67	49.3
3708	south-coast	2010	2	24	55	142	19.8	46	46	63	75.7
3709	south-coast	2010	2	25	56	147	30	44	44	72	90.4
3710	south-coast	2010	2	26	57	143	18.1	43	47	73	76.5

3711	south-coast	2010	2	27	58	143	12.2	45	48	69	98.6
3712	south-coast	2010	2	28	59	121	14	46	39	68	94.8
3713	south-coast	2010	3	1	60	174	21.3	45	39	73	87.2
3714	south-coast	2010	3	2	61	145	16.6	44	49	66	94.3
3715	south-coast	2010	3	3	62	124	11.9	46	49	65	96.3
3716	south-coast	2010	3	4	63	120	14.3	52	44	65	92.8
3717	south-coast	2010	3	5	64	149	14	52	38	68	76.2
3718	south-coast	2010	3	6	65	151	14.4	45	46	66	79.3
3719	south-coast	2010	3	7	66	143	9.7	46	42	66	99.5
3720	south-coast	2010	3	8	67	136	14.4	46	46	66	100
3721	south-coast	2010	3	9	68	145	16.1	46	40	60	83.4
3722	south-coast	2010	3	10	69	139	11.3	46	36	64	75.5
3723	south-coast	2010	3	11	70	153	14.3	50	37	68	64.1
3724	south-coast	2010	3	12	71	145	18.9	54	39	72	62.7
3725	south-coast	2010	3	13	72	136	17	51	47	68	84.5
3726	south-coast	2010	3	14	73	139	13.5	58	46	73	64.5
3727	south-coast	2010	3	15	74	157	12.2	55	49	82	46.7
3728	south-coast	2010	3	16	75	160	10	60	46	86	61.8
3729	south-coast	2010	3	17	76	140	10.8	60	48	88	56.5
3730	south-coast	2010	3	18	77	163	22.3	67	48	84	81.6
3731	south-coast	2010	3	19	78	177	25	54	47	80	77
3732	south-coast	2010	3	20	79	139	28.1	65	46	84	77.2
3733	south-coast	2010	3	21	80	148	26.4	69	46	83	79.7
3734	south-coast	2010	3	22	81	132	26.3	66	46	79	82.8
3735	south-coast	2010	3	23	82	113	19.5	57	47	79	76.2
3736	south-coast	2010	3	24	83	128	24.6	63	44	78	85.6
3737	south-coast	2010	3	25	84	136	23	60	43	76	87.5
3738	south-coast	2010	3	26	85	153	17.6	53	46	76	75.3
3739	south-coast	2010	3	27	86	146	13.5	59	45	83	43.9
3740	south-coast	2010	3	28	87	146	9.6	65	44	85	36.5
3741	south-coast	2010	3	29	88	155	15.8	70	47	84	55.5
3742	south-coast	2010	3	30	89	150	14.7	54	45	82	89.9
3743	south-coast	2010	3	31	90	146	10.8	52	49	69	97.3
3744	south-coast	2010	4	1	91	139	11.4	52	41	65	87.6
3745	south-coast	2010	4	2	92	140	44.4	59	39	68	80
3746	south-coast	2010	4	3	93	135	19.9	62	41	70	83.1
3747	south-coast	2010	4	4	94	125	17.2	54	46	68	89.1
3748	south-coast	2010	4	5	95	118	8.5	50	46	64	92
3749	south-coast	2010	4	6	96	158	12	58	38	76	62.3
3750	south-coast	2010	4	7	97	136	10.4	56	46	84	43.7
3751	south-coast	2010	4	8	98	143	13.6	63	46	83	45
3752	south-coast	2010	4	9	99	139	20.6	71	47	82	83.3
3753	south-coast	2010	4	10	100	134	20.4	60	48	77	84.5
3754	south-coast	2010	4	11	101	145	19	55	48	69	95
3755	south-coast	2010	4	12	102	153	15.2	54	47	63	99.3
3756	south-coast	2010	4	13	103	176	19.8	56	41	68	85.6
3757	south-coast	2010	4	14	104	125	19.3	61	42	73	84.5
3758	south-coast	2010	4	15	105	129	24.4	73	44	78	81.7
3759	south-coast	2010	4	16	106	131	31.9	74	47	74	84.8

3760	south-coast	2010	4	17	107	136	26.4	83	46	81	75.2
3761	south-coast	2010	4	18	108	125	27.6	80	49	82	81.9
3762	south-coast	2010	4	19	109	123	21.3	64	49	81	79.3
3763	south-coast	2010	4	20	110	133	12.4	55	48	74	92.6
3764	south-coast	2010	4	21	111	124	7.7	48	43	63	99.1
3765	south-coast	2010	4	22	112	105	9.5	43	41	59	99.9
3766	south-coast	2010	4	23	113	146	20.4	53	41	71	87.4
3767	south-coast	2010	4	24	114	127	24.7	65	51	76	83.6
3768	south-coast	2010	4	25	115	124	31.9	82	47	84	86.5
3769	south-coast	2010	4	26	116	140	37.3	74	47	85	90.5
3770	south-coast	2010	4	27	117	147	34.7	73	50	77	94.4
3771	south-coast	2010	4	28	118	143	12.8	55	53	70	97.1
3772	south-coast	2010	4	29	119	123	10.2	52	44	70	72.8
3773	south-coast	2010	4	30	120	112	10.4	50	48	75	58.1
3774	south-coast	2010	5	1	121	140	12.9	57	42	75	74.8
3775	south-coast	2010	5	2	122	133	16.8	66	47	76	82.8
3776	south-coast	2010	5	3	123	136	18.3	69	47	85	72
3777	south-coast	2010	5	4	124	151	22	77	50	86	82
3778	south-coast	2010	5	5	125	148	25.5	78	52	84	85.1
3779	south-coast	2010	5	6	126	149	29.8	73	53	79	80.7
3780	south-coast	2010	5	7	127	137	27.8	80	51	87	70.1
3781	south-coast	2010	5	8	128	136	23	89	51	85	74.3
3782	south-coast	2010	5	9	129	113	15.8	60	46	80	88
3783	south-coast	2010	5	10	130	127	10.7	57	46	69	94
3784	south-coast	2010	5	11	131	127	10.7	55	45	69	74.7
3785	south-coast	2010	5	12	132	117	13.4	59	42	79	71.9
3786	south-coast	2010	5	13	133	141	20	70	51	80	77.3
3787	south-coast	2010	5	14	134	144	25	62	49	81	83.1
3788	south-coast	2010	5	15	135	112	37.1	80	50	87	87.7
3789	south-coast	2010	5	16	136	139	36.4	87	54	84	94.3
3790	south-coast	2010	5	17	137	121	17.2	60	54	74	98.5
3791	south-coast	2010	5	18	138	121	13.9	42	51	71	99.1
3792	south-coast	2010	5	19	139	121	26	64	55	80	89
3793	south-coast	2010	5	20	140	138	25.9	72	54	85	79.1
3794	south-coast	2010	5	21	141	135	23.6	69	53	81	80.3
3795	south-coast	2010	5	22	142	139	19.4	58	53	78	88.2
3796	south-coast	2010	5	23	143	126	14.8	50	48	70	91.2
3797	south-coast	2010	5	24	144	137	18.7	54	44	73	75.4
3798	south-coast	2010	5	25	145	127	16.1	60	44	76	76.3
3799	south-coast	2010	5	26	146	129	18	54	47	75	83
3800	south-coast	2010	5	27	147	121	14.7	47	55	71	95.2
3801	south-coast	2010	5	28	148	134	14.7	56	48	73	79.6
3802	south-coast	2010	5	29	149	134	13.6	72	47	90	64.9
3803	south-coast	2010	5	30	150	152	18.5	82	48	92	84.5
3804	south-coast	2010	5	31	151	148	22.7	85	55	85	84.2
3805	south-coast	2010	6	1	152	125	24.8	72	52	81	83.9
3806	south-coast	2010	6	2	153	140	31.2	71	56	82	80.7
3807	south-coast	2010	6	3	154	114	34.5	96	50	87	88.1
3808	south-coast	2010	6	4	155	134	36.9	97	58	89	88.3

3809	south-coast	2010	6	5	156	122	40.8	123	60	95	90.7
3810	south-coast	2010	6	6	157	125	31.2	103	60	95	87.7
3811	south-coast	2010	6	7	158	131	30.4	80	60	90	90.4
3812	south-coast	2010	6	8	159	138	31.5	81	60	87	94
3813	south-coast	2010	6	9	160	129	23.7	77	58	82	88.9
3814	south-coast	2010	6	10	161	134	19.9	68	58	78	95.8
3815	south-coast	2010	6	11	162	112	17.1	53	57	71	94.8
3816	south-coast	2010	6	12	163	120	20.7	62	56	76	90.8
3817	south-coast	2010	6	13	164	128	33	84	54	87	90.6
3818	south-coast	2010	6	14	165	126	30.2	88	54	94	87.2
3819	south-coast	2010	6	15	166	134	25.2	79	58	93	86.4
3820	south-coast	2010	6	16	167	113	24.8	69	55	84	82.7
3821	south-coast	2010	6	17	168	131	27.8	97	53	87	82.8
3822	south-coast	2010	6	18	169	131	30	88	55	85	82.2
3823	south-coast	2010	6	19	170	138	23.8	84	53	86	77.6
3824	south-coast	2010	6	20	171	114	19.5	78	53	87	77.9
3825	south-coast	2010	6	21	172	137	19.6	64	53	82	81.2
3826	south-coast	2010	6	22	173	127	26.3	88	52	87	82.5
3827	south-coast	2010	6	23	174	128	29.2	112	55	90	85.5
3828	south-coast	2010	6	24	175	124	30.4	101	57	93	84.1
3829	south-coast	2010	6	25	176	151	28.7	97	56	89	84.9
3830	south-coast	2010	6	26	177	129	22.1	76	58	87	85.8
3831	south-coast	2010	6	27	178	142	29.9	90	58	87	86.6
3832	south-coast	2010	6	28	179	131	29	98	58	86	93.4
3833	south-coast	2010	6	29	180	118	32.9	85	59	87	91.7
3834	south-coast	2010	6	30	181	133	39	109	58	93	92.9
3835	south-coast	2010	7	1	182	126	37.5	92	57	94	82.9
3836	south-coast	2010	7	2	183	126	28.9	88	57	93	82.8
3837	south-coast	2010	7	3	184	142	27.1	88	57	89	87.5
3838	south-coast	2010	7	4	185	106	36.3	76	56	84	84.6
3839	south-coast	2010	7	5	186	108	67.8	85	57	84	93.4
3840	south-coast	2010	7	6	187	117	29.9	75	59	80	93
3841	south-coast	2010	7	7	188	119	36.1	78	57	82	90.5
3842	south-coast	2010	7	8	189	133	41.7	102	57	83	91.8
3843	south-coast	2010	7	9	190	121	44.4	99	55	92	90.6
3844	south-coast	2010	7	10	191	127	35.9	107	60	91	89.2
3845	south-coast	2010	7	11	192	126	32.2	88	60	91	91
3846	south-coast	2010	7	12	193	120	30.4	79	60	92	88.6
3847	south-coast	2010	7	13	194	147	19.6	93	61	97	68.6
3848	south-coast	2010	7	14	195	135	18.8	84	62	103	66.2
3849	south-coast	2010	7	15	196	137	29.2	97	65	105	70
3850	south-coast	2010	7	16	197	120	28.5	105	65	103	68.3
3851	south-coast	2010	7	17	198	134	25	118	64	103	72.2
3852	south-coast	2010	7	18	199	131	23.6	103	64	105	89
3853	south-coast	2010	7	19	200	122	26.3	99	64	96	87.8
3854	south-coast	2010	7	20	201	148	25.5	99	63	94	90.3
3855	south-coast	2010	7	21	202	113	29.5	94	60	90	91.4
3856	south-coast	2010	7	22	203	131	28.9	94	59	92	89.6
3857	south-coast	2010	7	23	204	136	25.3	96	61	92	86.3

3858	south-coast	2010	7	24	205	127	25.6	102	61	94	83.8
3859	south-coast	2010	7	25	206	108	21.8	98	61	92	88.8
3860	south-coast	2010	7	26	207	106	26.9	80	60	87	89.8
3861	south-coast	2010	7	27	208	112	34.8	84	58	86	87.9
3862	south-coast	2010	7	28	209	124	23.4	78	55	89	81.3
3863	south-coast	2010	7	29	210	125	21	82	58	92	81.3
3864	south-coast	2010	7	30	211	112	26.4	94	55	90	84.4
3865	south-coast	2010	7	31	212	109	28.4	94	60	91	88.6
3866	south-coast	2010	8	1	213	120	28	94	60	90	82.9
3867	south-coast	2010	8	2	214	123	24.5	84	56	96	85.4
3868	south-coast	2010	8	3	215	134	23.3	93	59	98	81.7
3869	south-coast	2010	8	4	216	124	24.6	92	60	97	85.7
3870	south-coast	2010	8	5	217	130	30.6	96	55	91	87
3871	south-coast	2010	8	6	218	126	32.1	87	55	89	86.6
3872	south-coast	2010	8	7	219	125	29.9	82	54	88	86.5
3873	south-coast	2010	8	8	220	109	23.4	71	56	87	82.2
3874	south-coast	2010	8	9	221	105	23.4	91	53	89	85.8
3875	south-coast	2010	8	10	222	117	26.1	86	53	90	86.9
3876	south-coast	2010	8	11	223	111	29.7	75	53	91	83.8
3877	south-coast	2010	8	12	224	131	24.4	90	53	92	86.8
3878	south-coast	2010	8	13	225	117	22.5	80	56	93	87.6
3879	south-coast	2010	8	14	226	121	21.5	82	55	96	86.6
3880	south-coast	2010	8	15	227	117	23.9	96	56	99	83.8
3881	south-coast	2010	8	16	228	161	25.6	91	56	102	84.1
3882	south-coast	2010	8	17	229	147	23.3	79	60	100	68.3
3883	south-coast	2010	8	18	230	122	18.7	71	64	98	63.6
3884	south-coast	2010	8	19	231	135	21.4	82	64	97	70.5
3885	south-coast	2010	8	20	232	126	19.5	69	61	97	73.7
3886	south-coast	2010	8	21	233	116	20.5	85	60	99	72.6
3887	south-coast	2010	8	22	234	124	18.1	77	61	97	70.5
3888	south-coast	2010	8	23	235	136	21	79	59	105	69.1
3889	south-coast	2010	8	24	236	132	27.2	94	68	109	74.7
3890	south-coast	2010	8	25	237	113	25.9	92	70	107	77
3891	south-coast	2010	8	26	238	130	23.4	89	61	106	79.1
3892	south-coast	2010	8	27	239	124	26.8	74	57	99	85.8
3893	south-coast	2010	8	28	240	115	26.5	72	57	88	84.2
3894	south-coast	2010	8	29	241	135	25.5	60	52	80	82.5
3895	south-coast	2010	8	30	242	133	21.4	63	46	79	79.8
3896	south-coast	2010	8	31	243	119	22.5	77	54	90	74.1
3897	south-coast	2010	9	1	244	118	30.2	84	59	100	84.5
3898	south-coast	2010	9	2	245	136	30.3	89	58	104	86.5
3899	south-coast	2010	9	3	246	132	26.1	85	61	106	82.5
3900	south-coast	2010	9	4	247	103	21.2	99	59	105	78.2
3901	south-coast	2010	9	5	248	146	27	84	59	104	88.3
3902	south-coast	2010	9	6	249	118	30	87	57	90	91.1
3903	south-coast	2010	9	7	250	127	32	61	59	82	96.5
3904	south-coast	2010	9	8	251	109	20.7	55	57	70	95.9
3905	south-coast	2010	9	9	252	108	18.3	63	56	77	87.1
3906	south-coast	2010	9	10	253	142	23	68	50	86	83.8

3907	south-coast	2010	9	11	254	137	31	82	50	94	92.8
3908	south-coast	2010	9	12	255	143	33.8	87	57	94	90.3
3909	south-coast	2010	9	13	256	120	23.8	70	59	95	79.5
3910	south-coast	2010	9	14	257	139	18.2	59	57	93	74.7
3911	south-coast	2010	9	15	258	116	20	55	56	91	81.8
3912	south-coast	2010	9	16	259	109	24.1	83	47	92	84.3
3913	south-coast	2010	9	17	260	116	31.7	79	54	91	89.1
3914	south-coast	2010	9	18	261	114	37.6	90	52	92	90.1
3915	south-coast	2010	9	19	262	137	32.8	89	54	93	86.6
3916	south-coast	2010	9	20	263	135	25.2	76	54	92	91.3
3917	south-coast	2010	9	21	264	138	40.5	63	52	83	97.3
3918	south-coast	2010	9	22	265	126	24.8	51	58	72	91.6
3919	south-coast	2010	9	23	266	124	33	68	50	88	77.3
3920	south-coast	2010	9	24	267	153	25.1	74	57	101	72.8
3921	south-coast	2010	9	25	268	145	24.9	90	61	106	64.9
3922	south-coast	2010	9	26	269	146	22.1	96	62	108	59.4
3923	south-coast	2010	9	27	270	159	19.8	75	66	111	49.3
3924	south-coast	2010	9	28	271	137	23.6	69	71	109	75.7
3925	south-coast	2010	9	29	272	126	22.5	72	65	99	70.1
3926	south-coast	2010	9	30	273	120	21	64	65	99	78.6
3927	south-coast	2010	10	1	274	131	25.4	70	63	95	84.4
3928	south-coast	2010	10	2	275	112	29.7	68	64	94	85
3929	south-coast	2010	10	3	276	128	30.5	64	61	91	93.9
3930	south-coast	2010	10	4	277	139	20.4	35	56	82	99.9
3931	south-coast	2010	10	5	278	113	14.2	43	53	65	100
3932	south-coast	2010	10	6	279	146	16.3	46	50	65	100
3933	south-coast	2010	10	7	280	130	19.9	48	52	79	90.5
3934	south-coast	2010	10	8	281	121	25	57	54	83	79.6
3935	south-coast	2010	10	9	282	126	27.9	68	52	93	74
3936	south-coast	2010	10	10	283	127	23.8	72	52	95	72.2
3937	south-coast	2010	10	11	284	149	27.2	72	59	92	93.7
3938	south-coast	2010	10	12	285	151	39.7	56	57	93	91.3
3939	south-coast	2010	10	13	286	152	33.6	51	59	98	94.1
3940	south-coast	2010	10	14	287	120	50.5	54	57	90	95.8
3941	south-coast	2010	10	15	288	134	59.2	55	60	77	97.8
3942	south-coast	2010	10	16	289	133	50.7	55	60	76	100
3943	south-coast	2010	10	17	290	125	30	61	59	75	99.8
3944	south-coast	2010	10	18	291	113	19	38	56	66	99
3945	south-coast	2010	10	19	292	133	30.4	28	58	72	100
3946	south-coast	2010	10	20	293	117	17.4	36	56	68	100
3947	south-coast	2010	10	21	294	113	25.7	37	56	65	99.7
3948	south-coast	2010	10	22	295	152	23.2	40	55	67	98
3949	south-coast	2010	10	23	296	141	19.5	37	55	69	98.5
3950	south-coast	2010	10	24	297	125	21.6	44	53	70	96.1
3951	south-coast	2010	10	25	298	129	14.2	42	57	75	97.4
3952	south-coast	2010	10	26	299	140	23.5	45	47	75	90.3
3953	south-coast	2010	10	27	300	124	12.8	46	46	82	45.5
3954	south-coast	2010	10	28	301	133	12.1	51	44	85	43.2
3955	south-coast	2010	10	29	302	151	19.2	50	49	85	63.3

3956	south-coast	2010	10	30	303	151	16.5	46	50	79	96.2
3957	south-coast	2010	10	31	304	120	24.2	48	48	74	90.7
3958	south-coast	2010	11	1	305	122	18.9	46	48	87	72.2
3959	south-coast	2010	11	2	306	125	16.1	46	52	93	48.7
3960	south-coast	2010	11	3	307	130	16.4	43	48	98	53.2
3961	south-coast	2010	11	4	308	140	28.3	47	53	101	53.3
3962	south-coast	2010	11	5	309	129	14.6	51	53	96	47.5
3963	south-coast	2010	11	6	310	128	18.4	49	50	88	76.5
3964	south-coast	2010	11	7	311	144	21.3	45	54	78	91.7
3965	south-coast	2010	11	8	312	132	11.9	42	53	69	92
3966	south-coast	2010	11	9	313	120	19.1	44	41	69	71.5
3967	south-coast	2010	11	10	314	118	16.8	43	41	67	87.3
3968	south-coast	2010	11	11	315	122	13.2	41	43	75	60.2
3969	south-coast	2010	11	12	316	130	12.3	42	39	79	48.6
3970	south-coast	2010	11	13	317	134	19	45	45	81	45.5
3971	south-coast	2010	11	14	318	161	13	47	45	80	45.8
3972	south-coast	2010	11	15	319	145	21.8	47	46	77	76.8
3973	south-coast	2010	11	16	320	147	31.4	47	46	76	90.6
3974	south-coast	2010	11	17	321	157	48.7	42	42	81	88.7
3975	south-coast	2010	11	18	322	133	41.8	55	46	78	95.2
3976	south-coast	2010	11	19	323	151	56.6	49	48	68	95.5
3977	south-coast	2010	11	20	324	142	13	40	50	64	100
3978	south-coast	2010	11	21	325	121	18.7	41	45	61	99.9
3979	south-coast	2010	11	22	326	135	19.7	41	40	61	89.9
3980	south-coast	2010	11	23	327	145	16.5	39	37	60	95.8
3981	south-coast	2010	11	24	328	140	15.6	42	44	59	94.5
3982	south-coast	2010	11	25	329	137	27.1	45	33	64	63.3
3983	south-coast	2010	11	26	330	135	26.6	45	31	69	50.5
3984	south-coast	2010	11	27	331	151	21.9	42	33	64	76.7
3985	south-coast	2010	11	28	332	150	14.1	42	41	58	75.4
3986	south-coast	2010	11	29	333	157	12.2	43	35	63	45.8
3987	south-coast	2010	11	30	334	122	15.7	44	31	65	43.9
3988	south-coast	2010	12	1	335	150	24.8	45	35	76	48.3
3989	south-coast	2010	12	2	336	161	23.7	41	40	77	72.4
3990	south-coast	2010	12	3	337	153	38.9	44	43	74	81.8
3991	south-coast	2010	12	4	338	160	45.3	44	42	66	87.9
3992	south-coast	2010	12	5	339	143	40	41	43	68	81.3
3993	south-coast	2010	12	6	340	147	28.6	39	47	68	93.7
3994	south-coast	2010	12	7	341	129	26.9	46	41	78	83.9
3995	south-coast	2010	12	8	342	153	25.9	44	41	78	79.3
3996	south-coast	2010	12	9	343	153	37.2	45	43	72	87.6
3997	south-coast	2010	12	10	344	172	54.1	33	45	71	97.9
3998	south-coast	2010	12	11	345	139	27	40	46	80	81.9
3999	south-coast	2010	12	12	346	146	13	36	46	86	76.5
4000	south-coast	2010	12	13	347	131	19.9	34	46	83	65.3
4001	south-coast	2010	12	14	348	134	35.1	45	44	75	100
4002	south-coast	2010	12	15	349	138	29.3	32	49	58	100
4003	south-coast	2010	12	16	350	139	13.9	42	47	62	90.8
4004	south-coast	2010	12	17	351	146	15.2	38	43	58	99

4005	south-coast	2010	12	18	352	147	15	36	52	60	99.3
4006	south-coast	2010	12	19	353	155	10	40	51	60	100
4007	south-coast	2010	12	20	354	133	10.7	41	50	60	99.8
4008	south-coast	2010	12	21	355	145	13.2	45	48	59	100
4009	south-coast	2010	12	22	356	142	10.9	46	48	58	98.2
4010	south-coast	2010	12	23	357	153	23.7	40	43	61	92.6
4011	south-coast	2010	12	24	358	130	27.1	38	39	71	83.4
4012	south-coast	2010	12	25	359	140	39.8	40	40	66	92.8
4013	south-coast	2010	12	26	360	148	14	41	46	59	93.2
4014	south-coast	2010	12	27	361	133	20.1	44	38	69	75.1
4015	south-coast	2010	12	28	362	123	20.4	44	38	68	80.7
4016	south-coast	2010	12	29	363	160	15.8	41	45	62	96.6
4017	south-coast	2010	12	30	364	128	12.5	41	38	57	62.1
4018	south-coast	2010	12	31	365	133	34.5	40	29	55	67
4019	south-coast	2011	1	1	1	171	34.8	40	31	57	49.5
4020	south-coast	2011	1	2	2	152	19.3	45	35	56	71.7
4021	south-coast	2011	1	3	3	165	10.6	39	39	56	88.1
4022	south-coast	2011	1	4	4	168	13.8	43	33	64	67.7
4023	south-coast	2011	1	5	5	164	15.4	44	34	65	67.2
4024	south-coast	2011	1	6	6	174	17.2	42	39	69	54.4
4025	south-coast	2011	1	7	7	143	26.8	39	37	66	88
4026	south-coast	2011	1	8	8	176	35.1	41	43	60	91
4027	south-coast	2011	1	9	9	154	29	43	46	59	86.1
4028	south-coast	2011	1	10	10	175	27.3	44	40	63	70.8
4029	south-coast	2011	1	11	11	133	11.3	40	38	63	42.2
4030	south-coast	2011	1	12	12	176	15.7	38	41	75	45.4
4031	south-coast	2011	1	13	13	163	22.2	44	40	77	55.2
4032	south-coast	2011	1	14	14	177	14.9	47	46	83	47.9
4033	south-coast	2011	1	15	15	153	27.4	47	45	83	42.6
4034	south-coast	2011	1	16	16	151	22.9	39	47	85	52.2
4035	south-coast	2011	1	17	17	136	32	39	53	89	68.2
4036	south-coast	2011	1	18	18	142	32.8	42	52	86	69.8
4037	south-coast	2011	1	19	19	146	42.4	43	48	80	92.6
4038	south-coast	2011	1	20	20	149	25.9	47	45	76	50.8
4039	south-coast	2011	1	21	21	159	27	44	42	76	35
4040	south-coast	2011	1	22	22	143	27.3	44	42	74	65.3
4041	south-coast	2011	1	23	23	148	21.8	49	47	76	48.5
4042	south-coast	2011	1	24	24	160	18.1	45	39	76	39.9
4043	south-coast	2011	1	25	25	164	27.8	40	44	76	42.6
4044	south-coast	2011	1	26	26	148	19.7	45	45	77	41.9
4045	south-coast	2011	1	27	27	148	15.7	42	38	77	38.3
4046	south-coast	2011	1	28	28	156	18	43	41	76	46.6
4047	south-coast	2011	1	29	29	153	25.3	53	38	73	86
4048	south-coast	2011	1	30	30	168	16.4	43	42	64	96.5
4049	south-coast	2011	1	31	31	147	14.4	44	41	64	91.1
4050	south-coast	2011	2	1	32	153	19.8	47	38	66	78.4
4051	south-coast	2011	2	2	33	162	16.6	48	36	65	43.9
4052	south-coast	2011	2	3	34	142	13	43	31	65	53.3
4053	south-coast	2011	2	4	35	150	24.2	44	34	71	82.4

4054	south-coast	2011	2	5	36	171	41.6	44	42	78	85.5
4055	south-coast	2011	2	6	37	155	35.5	44	46	79	79.7
4056	south-coast	2011	2	7	38	164	20.9	48	46	78	69.2
4057	south-coast	2011	2	8	39	169	23.4	46	44	73	86
4058	south-coast	2011	2	9	40	158	14.8	45	37	71	49
4059	south-coast	2011	2	10	41	181	12.1	44	34	71	35.9
4060	south-coast	2011	2	11	42	158	14.4	47	36	76	42.1
4061	south-coast	2011	2	12	43	172	16.6	49	39	80	36
4062	south-coast	2011	2	13	44	154	20.5	55	42	80	63.4
4063	south-coast	2011	2	14	45	148	26.4	56	39	69	76.4
4064	south-coast	2011	2	15	46	170	20.5	42	45	64	90.8
4065	south-coast	2011	2	16	47	186	9.8	44	51	62	98.9
4066	south-coast	2011	2	17	48	144	13.5	46	37	59	79.7
4067	south-coast	2011	2	18	49	174	13.9	47	40	64	86.8
4068	south-coast	2011	2	19	50	165	10	48	43	56	91.4
4069	south-coast	2011	2	20	51	153	15.7	49	38	57	86.7
4070	south-coast	2011	2	21	52	161	21.2	49	35	59	83.3
4071	south-coast	2011	2	22	53	141	19.9	52	36	62	82
4072	south-coast	2011	2	23	54	165	22.3	49	39	60	88
4073	south-coast	2011	2	24	55	154	17.3	48	42	59	85.8
4074	south-coast	2011	2	25	56	174	13.4	46	44	58	94.8
4075	south-coast	2011	2	26	57	156	12.7	46	39	56	83.5
4076	south-coast	2011	2	27	58	140	18.8	49	29	58	73.3
4077	south-coast	2011	2	28	59	159	18.1	48	33	66	67.2
4078	south-coast	2011	3	1	60	173	21.6	52	36	66	68.9
4079	south-coast	2011	3	2	61	138	25	57	41	66	73.5
4080	south-coast	2011	3	3	62	171	28.9	42	49	68	93.5
4081	south-coast	2011	3	4	63	162	25.9	50	43	78	79.6
4082	south-coast	2011	3	5	64	159	16.4	59	45	80	56.2
4083	south-coast	2011	3	6	65	169	18.3	60	47	76	86.4
4084	south-coast	2011	3	7	66	143	12.1	63	49	66	94
4085	south-coast	2011	3	8	67	147	15.7	50	42	73	71
4086	south-coast	2011	3	9	68	196	14	50	45	84	66.8
4087	south-coast	2011	3	10	69	156	20.5	55	48	82	65.7
4088	south-coast	2011	3	11	70	183	25	57	46	77	78.3
4089	south-coast	2011	3	12	71	159	27.5	50	46	68	90.4
4090	south-coast	2011	3	13	72	142	33.4	71	45	71	88
4091	south-coast	2011	3	14	73	151	31.3	60	46	74	80.3
4092	south-coast	2011	3	15	74	151	28	56	49	81	80.2
4093	south-coast	2011	3	16	75	142	22.7	52	48	77	85.2
4094	south-coast	2011	3	17	76	139	23	54	49	72	75.7
4095	south-coast	2011	3	18	77	139	19.8	55	42	73	70.4
4096	south-coast	2011	3	19	78	159	17.7	57	47	67	93.6
4097	south-coast	2011	3	20	79	149	14.3	54	46	58	88.2
4098	south-coast	2011	3	21	80	151	13.5	54	39	57	100
4099	south-coast	2011	3	22	81	148	15.5	52	39	62	92.9
4100	south-coast	2011	3	23	82	154	16.4	52	37	66	82.9
4101	south-coast	2011	3	24	83	148	16.3	53	38	60	96.3
4102	south-coast	2011	3	25	84	145	11.5	55	46	61	100

4103	south-coast	2011	3	26	85	153	17.3	51	45	61	100
4104	south-coast	2011	3	27	86	143	11.7	53	48	62	99.4
4105	south-coast	2011	3	28	87	137	22	46	44	68	89.6
4106	south-coast	2011	3	29	88	151	31.5	48	44	78	86
4107	south-coast	2011	3	30	89	156	21.6	51	50	86	73.9
4108	south-coast	2011	3	31	90	170	19.5	61	56	94	64.1
4109	south-coast	2011	4	1	91	169	25.7	81	54	94	71.9
4110	south-coast	2011	4	2	92	160	27.5	51	55	87	91.8
4111	south-coast	2011	4	3	93	126	14.4	48	53	69	97.8
4112	south-coast	2011	4	4	94	147	16.3	64	46	83	75.8
4113	south-coast	2011	4	5	95	143	26.8	66	49	83	81.3
4114	south-coast	2011	4	6	96	144	28	51	41	71	86.8
4115	south-coast	2011	4	7	97	141	16.3	54	53	71	98.5
4116	south-coast	2011	4	8	98	143	9	54	40	58	94.8
4117	south-coast	2011	4	9	99	156	13.9	57	33	61	90
4118	south-coast	2011	4	10	100	159	17.8	64	37	70	71.6
4119	south-coast	2011	4	11	101	142	20.4	65	44	76	69.6
4120	south-coast	2011	4	12	102	126	21.9	66	44	73	79.3
4121	south-coast	2011	4	13	103	132	19.5	60	51	69	83.8
4122	south-coast	2011	4	14	104	138	23.8	71	42	78	58.3
4123	south-coast	2011	4	15	105	138	27.3	72	48	88	48.9
4124	south-coast	2011	4	16	106	164	28	89	41	95	54.9
4125	south-coast	2011	4	17	107	147	21.8	86	54	92	90.7
4126	south-coast	2011	4	18	108	148	19.6	46	54	78	99
4127	south-coast	2011	4	19	109	144	19.8	49	54	69	91.3
4128	south-coast	2011	4	20	110	133	21.6	43	54	71	94.8
4129	south-coast	2011	4	21	111	156	19.5	46	53	71	97.2
4130	south-coast	2011	4	22	112	136	19.8	52	53	72	90.1
4131	south-coast	2011	4	23	113	125	18.9	58	53	70	99.7
4132	south-coast	2011	4	24	114	136	18	46	52	69	99.9
4133	south-coast	2011	4	25	115	140	17.1	53	50	77	81
4134	south-coast	2011	4	26	116	129	19	65	52	78	68.8
4135	south-coast	2011	4	27	117	176	17.9	74	49	89	54.9
4136	south-coast	2011	4	28	118	151	19.4	79	54	86	65.1
4137	south-coast	2011	4	29	119	149	21.4	67	43	80	76.3
4138	south-coast	2011	4	30	120	130	11.9	63	45	79	41.3
4139	south-coast	2011	5	1	121	132	8	61	49	82	29.1
4140	south-coast	2011	5	2	122	131	10.7	61	46	87	30.5
4141	south-coast	2011	5	3	123	163	13.5	76	51	95	36.8
4142	south-coast	2011	5	4	124	137	23	98	52	98	65.8
4143	south-coast	2011	5	5	125	145	27.5	97	52	95	80.2
4144	south-coast	2011	5	6	126	143	39	90	52	87	83.2
4145	south-coast	2011	5	7	127	122	36.3	76	53	85	91.2
4146	south-coast	2011	5	8	128	117	14.6	54	55	76	100
4147	south-coast	2011	5	9	129	148	10.4	61	50	65	100
4148	south-coast	2011	5	10	130	114	17.4	61	49	71	83.5
4149	south-coast	2011	5	11	131	150	23.2	67	45	79	84.9
4150	south-coast	2011	5	12	132	140	28.5	83	50	89	75.5
4151	south-coast	2011	5	13	133	169	24.3	78	53	84	83.6

4152	south-coast	2011	5	14	134	125	20.3	60	55	80	92.1
4153	south-coast	2011	5	15	135	124	12.1	57	51	70	91.8
4154	south-coast	2011	5	16	136	122	11.5	52	48	66	86
4155	south-coast	2011	5	17	137	114	11.3	56	48	70	99.7
4156	south-coast	2011	5	18	138	153	12.2	55	49	64	100
4157	south-coast	2011	5	19	139	148	18	57	48	71	88.1
4158	south-coast	2011	5	20	140	138	24.2	72	52	76	84.6
4159	south-coast	2011	5	21	141	140	30.5	81	50	83	86.4
4160	south-coast	2011	5	22	142	129	26.2	69	55	83	95.7
4161	south-coast	2011	5	23	143	118	19.3	64	54	72	95.1
4162	south-coast	2011	5	24	144	114	21.7	66	50	76	83.8
4163	south-coast	2011	5	25	145	122	24.4	84	50	80	76.2
4164	south-coast	2011	5	26	146	123	17.8	71	49	81	81.2
4165	south-coast	2011	5	27	147	141	21.5	78	52	84	77.9
4166	south-coast	2011	5	28	148	135	17.9	67	53	82	86.6
4167	south-coast	2011	5	29	149	134	10.4	55	52	73	84.4
4168	south-coast	2011	5	30	150	132	12	70	45	82	65.3
4169	south-coast	2011	5	31	151	120	15	77	49	84	72.8
4170	south-coast	2011	6	1	152	134	13.5	62	47	76	72
4171	south-coast	2011	6	2	153	125	15.4	62	45	79	68.6
4172	south-coast	2011	6	3	154	137	17.5	71	49	83	74.6
4173	south-coast	2011	6	4	155	142	17.3	68	48	83	77.5
4174	south-coast	2011	6	5	156	126	15.9	67	48	87	76.3
4175	south-coast	2011	6	6	157	133	11.4	54	51	74	80.9
4176	south-coast	2011	6	7	158	112	15.5	63	49	80	79.8
4177	south-coast	2011	6	8	159	112	21.7	56	53	78	95.8
4178	south-coast	2011	6	9	160	146	31.8	65	56	76	94
4179	south-coast	2011	6	10	161	128	38.3	74	56	78	94.4
4180	south-coast	2011	6	11	162	142	34.1	80	56	79	95.6
4181	south-coast	2011	6	12	163	137	22.8	76	56	79	91.2
4182	south-coast	2011	6	13	164	131	26.6	66	54	84	86.8
4183	south-coast	2011	6	14	165	135	31.7	96	56	91	83.1
4184	south-coast	2011	6	15	166	142	34.6	97	58	90	87.8
4185	south-coast	2011	6	16	167	136	27.6	65	57	83	93.8
4186	south-coast	2011	6	17	168	141	23.6	74	58	82	90.5
4187	south-coast	2011	6	18	169	141	25.5	84	58	85	86.3
4188	south-coast	2011	6	19	170	108	23	76	57	84	91.5
4189	south-coast	2011	6	20	171	140	31.7	90	55	96	83.1
4190	south-coast	2011	6	21	172	147	36.5	115	58	98	84.9
4191	south-coast	2011	6	22	173	123	37	120	60	98	84.6
4192	south-coast	2011	6	23	174	129	35	94	59	97	86.8
4193	south-coast	2011	6	24	175	131	33.5	80	58	94	83.3
4194	south-coast	2011	6	25	176	130	35.2	92	58	95	81
4195	south-coast	2011	6	26	177	126	27.5	100	58	96	78.4
4196	south-coast	2011	6	27	178	138	27.6	101	58	99	77.4
4197	south-coast	2011	6	28	179	138	27.2	87	54	94	84.3
4198	south-coast	2011	6	29	180	127	23.6	52	58	86	84
4199	south-coast	2011	6	30	181	121	17.6	80	55	93	73.5
4200	south-coast	2011	7	1	182	167	26.3	113	60	103	74.3

4201	south-coast	2011	7	2	183	147	29.4	136	63	103	79.6
4202	south-coast	2011	7	3	184	135	46.8	115	64	104	80.5
4203	south-coast	2011	7	4	185	125	31.7	93	65	101	77.2
4204	south-coast	2011	7	5	186	136	94.6	97	67	98	77.5
4205	south-coast	2011	7	6	187	137	25.8	87	66	99	78.2
4206	south-coast	2011	7	7	188	131	27.3	101	66	99	77.5
4207	south-coast	2011	7	8	189	130	33.4	93	65	97	77.4
4208	south-coast	2011	7	9	190	130	24.8	86	64	98	80.8
4209	south-coast	2011	7	10	191	126	29.8	83	62	97	83.2
4210	south-coast	2011	7	11	192	135	30.6	87	60	89	84.6
4211	south-coast	2011	7	12	193	116	27.4	80	58	87	84.5
4212	south-coast	2011	7	13	194	125	22.8	70	62	83	88.3
4213	south-coast	2011	7	14	195	141	21.8	58	61	89	89.8
4214	south-coast	2011	7	15	196	139	19.2	63	61	85	81.6
4215	south-coast	2011	7	16	197	137	22.9	79	58	86	84.3
4216	south-coast	2011	7	17	198	131	24.9	94	55	95	76.4
4217	south-coast	2011	7	18	199	122	21.6	92	54	99	80
4218	south-coast	2011	7	19	200	132	18.5	88	63	100	75.3
4219	south-coast	2011	7	20	201	124	23.9	83	61	98	80.7
4220	south-coast	2011	7	21	202	127	31.2	87	60	95	81.5
4221	south-coast	2011	7	22	203	131	31.7	85	56	91	81.5
4222	south-coast	2011	7	23	204	111	32.2	93	56	96	76.3
4223	south-coast	2011	7	24	205	132	29	99	54	99	80.2
4224	south-coast	2011	7	25	206	136	20.3	85	64	97	73.3
4225	south-coast	2011	7	26	207	118	46.9	69	65	96	77.3
4226	south-coast	2011	7	27	208	123	23.9	84	56	89	87.5
4227	south-coast	2011	7	28	209	121	26.5	88	61	92	84.5
4228	south-coast	2011	7	29	210	127	30.6	92	63	90	89.3
4229	south-coast	2011	7	30	211	140	28.8	93	62	94	86
4230	south-coast	2011	7	31	212	141	97.5	64	64	92	84.5
4231	south-coast	2011	8	1	213	137	26.3	93	63	102	73.2
4232	south-coast	2011	8	2	214	120	17.2	82	62	103	74.5
4233	south-coast	2011	8	3	215	113	17.8	72	62	101	77.8
4234	south-coast	2011	8	4	216	128	19.8	76	59	100	77.8
4235	south-coast	2011	8	5	217	103	22.9	70	59	95	77.5
4236	south-coast	2011	8	6	218	146	29.5	84	60	95	80.2
4237	south-coast	2011	8	7	219	137	31.5	100	59	97	82
4238	south-coast	2011	8	8	220	138	30.4	97	60	95	83.6
4239	south-coast	2011	8	9	221	131	33	85	61	94	85.4
4240	south-coast	2011	8	10	222	108	36.4	79	59	89	87.3
4241	south-coast	2011	8	11	223	145	31.3	78	58	88	87.5
4242	south-coast	2011	8	12	224	146	43.2	90	61	92	85.6
4243	south-coast	2011	8	13	225	112	36.9	105	62	93	86.8
4244	south-coast	2011	8	14	226	125	38.1	108	62	97	85.5
4245	south-coast	2011	8	15	227	146	31	101	62	98	83
4246	south-coast	2011	8	16	228	127	38.2	96	62	100	83.8
4247	south-coast	2011	8	17	229	111	36.7	103	62	102	80.3
4248	south-coast	2011	8	18	230	135	34.7	103	62	101	83.2
4249	south-coast	2011	8	19	231	130	39.2	99	61	92	86.2

4250	south-coast	2011	8	20	232	137	36	90	60	90	86.7
4251	south-coast	2011	8	21	233	138	28.8	92	56	92	82.9
4252	south-coast	2011	8	22	234	117	26.2	85	61	97	81.8
4253	south-coast	2011	8	23	235	131	23	94	63	102	80.5
4254	south-coast	2011	8	24	236	111	27.2	102	63	102	78.3
4255	south-coast	2011	8	25	237	140	29.9	104	63	105	72.3
4256	south-coast	2011	8	26	238	143	29.7	97	66	108	72
4257	south-coast	2011	8	27	239	150	26.8	111	74	110	73.8
4258	south-coast	2011	8	28	240	114	33.8	113	65	106	71.8
4259	south-coast	2011	8	29	241	146	31.3	97	64	105	75.7
4260	south-coast	2011	8	30	242	126	32.5	82	61	100	80.6
4261	south-coast	2011	8	31	243	118	34.2	90	58	98	80.6
4262	south-coast	2011	9	1	244	117	32.6	88	58	98	79.2
4263	south-coast	2011	9	2	245	118	36.9	91	60	98	82
4264	south-coast	2011	9	3	246	123	31.6	96	59	100	79.3
4265	south-coast	2011	9	4	247	110	28.6	91	61	98	78.3
4266	south-coast	2011	9	5	248	118	31.2	85	62	96	73.5
4267	south-coast	2011	9	6	249	133	21.8	80	62	104	66.9
4268	south-coast	2011	9	7	250	135	27.4	91	65	106	70
4269	south-coast	2011	9	8	251	125	25	85	66	103	68.3
4270	south-coast	2011	9	9	252	122	27.2	97	61	94	81.3
4271	south-coast	2011	9	10	253	120	29.4	68	60	85	92.3
4272	south-coast	2011	9	11	254	113	23.1	68	61	86	83
4273	south-coast	2011	9	12	255	119	25.6	73	55	97	77
4274	south-coast	2011	9	13	256	127	28.9	68	63	94	78.8
4275	south-coast	2011	9	14	257	112	32.5	76	60	88	87.1
4276	south-coast	2011	9	15	258	133	41.3	80	60	90	88.9
4277	south-coast	2011	9	16	259	131	41.5	72	62	81	96.9
4278	south-coast	2011	9	17	260	132	36.2	61	61	82	90.3
4279	south-coast	2011	9	18	261	120	36.5	85	55	99	81.4
4280	south-coast	2011	9	19	262	134	40.3	78	61	102	83.1
4281	south-coast	2011	9	20	263	128	50.6	86	60	95	87.7
4282	south-coast	2011	9	21	264	132	44	85	58	96	86.5
4283	south-coast	2011	9	22	265	112	44.2	69	59	103	84.5
4284	south-coast	2011	9	23	266	124	53.2	83	62	97	88.2
4285	south-coast	2011	9	24	267	124	45.2	82	62	85	96.2
4286	south-coast	2011	9	25	268	127	39.3	66	61	78	94.3
4287	south-coast	2011	9	26	269	132	45.1	67	56	83	91.5
4288	south-coast	2011	9	27	270	132	45.2	67	57	102	80
4289	south-coast	2011	9	28	271	114	57.3	74	60	97	92.7
4290	south-coast	2011	9	29	272	126	41.2	75	59	93	82.7
4291	south-coast	2011	9	30	273	115	31.5	51	59	94	86
4292	south-coast	2011	10	1	274	133	23	62	62	98	72.1
4293	south-coast	2011	10	2	275	134	17	53	62	93	70.3
4294	south-coast	2011	10	3	276	128	15.9	39	60	86	73
4295	south-coast	2011	10	4	277	103	13.8	41	56	78	99.3
4296	south-coast	2011	10	5	278	126	13.1	39	54	68	100
4297	south-coast	2011	10	6	279	134	15.6	44	49	68	96.3
4298	south-coast	2011	10	7	280	126	15.8	45	46	74	75.1

4299	south-coast	2011	10	8	281	127	17.4	54	48	84	64.8
4300	south-coast	2011	10	9	282	146	16	62	51	84	64.3
4301	south-coast	2011	10	10	283	156	24.8	65	52	85	78.8
4302	south-coast	2011	10	11	284	145	29.3	71	54	92	80.3
4303	south-coast	2011	10	12	285	148	19.2	61	59	101	57.7
4304	south-coast	2011	10	13	286	110	19.5	70	61	103	51.1
4305	south-coast	2011	10	14	287	126	30.6	78	60	95	80
4306	south-coast	2011	10	15	288	139	44.8	100	57	88	88.7
4307	south-coast	2011	10	16	289	150	38.7	81	55	91	86.9
4308	south-coast	2011	10	17	290	117	34.3	56	56	93	85
4309	south-coast	2011	10	18	291	148	45	61	56	91	92.1
4310	south-coast	2011	10	19	292	130	50.9	68	54	84	94.8
4311	south-coast	2011	10	20	293	130	52.3	58	53	80	91.5
4312	south-coast	2011	10	21	294	120	71	60	55	80	91.1
4313	south-coast	2011	10	22	295	125	43.8	57	54	97	86.6
4314	south-coast	2011	10	23	296	127	62.6	68	57	96	88.3
4315	south-coast	2011	10	24	297	123	73.8	66	48	82	95.8
4316	south-coast	2011	10	25	298	114	33.5	44	57	70	99
4317	south-coast	2011	10	26	299	138	23.7	47	55	79	92.3
4318	south-coast	2011	10	27	300	125	21.3	51	46	80	70.5
4319	south-coast	2011	10	28	301	123	21.4	55	45	85	57.6
4320	south-coast	2011	10	29	302	136	29	62	48	89	62.6
4321	south-coast	2011	10	30	303	152	36.9	63	51	94	68.4
4322	south-coast	2011	10	31	304	133	40	73	51	89	85
4323	south-coast	2011	11	1	305	129	69.3	64	49	83	88.5
4324	south-coast	2011	11	2	306	138	26.5	48	47	81	41.4
4325	south-coast	2011	11	3	307	109	15.5	48	44	80	54.5
4326	south-coast	2011	11	4	308	139	16.8	42	49	71	98.9
4327	south-coast	2011	11	5	309	132	18.4	41	40	62	90.4
4328	south-coast	2011	11	6	310	141	20.2	37	39	75	93.8
4329	south-coast	2011	11	7	311	139	17.3	39	38	64	82.3
4330	south-coast	2011	11	8	312	126	23.1	44	38	75	65.9
4331	south-coast	2011	11	9	313	141	16.4	47	38	76	46.7
4332	south-coast	2011	11	10	314	141	15.8	47	41	73	45.1
4333	south-coast	2011	11	11	315	156	21.8	49	51	75	48.8
4334	south-coast	2011	11	12	316	160	14.6	47	48	67	87
4335	south-coast	2011	11	13	317	153	17.4	43	45	72	89.8
4336	south-coast	2011	11	14	318	150	35.3	42	42	69	91.3
4337	south-coast	2011	11	15	319	139	34.3	42	52	69	93
4338	south-coast	2011	11	16	320	148	50.8	44	44	75	91.8
4339	south-coast	2011	11	17	321	133	46.8	50	46	78	86.4
4340	south-coast	2011	11	18	322	141	44.1	50	43	69	98.9
4341	south-coast	2011	11	19	323	130	16.1	41	48	62	100
4342	south-coast	2011	11	20	324	142	19.8	42	43	60	99.3
4343	south-coast	2011	11	21	325	143	21	41	44	62	94.3
4344	south-coast	2011	11	22	326	143	26.9	43	38	73	87.4
4345	south-coast	2011	11	23	327	144	28.9	42	42	71	82
4346	south-coast	2011	11	24	328	122	31	43	45	64	94.1
4347	south-coast	2011	11	25	329	114	32.3	43	41	70	81.3

4348	south-coast	2011	11	26	330	136	25.8	45	45	82	61
4349	south-coast	2011	11	27	331	143	13.2	43	45	82	56
4350	south-coast	2011	11	28	332	149	17.2	35	47	83	58
4351	south-coast	2011	11	29	333	139	31.6	30	50	83	59.6
4352	south-coast	2011	11	30	334	120	29.6	43	46	78	81
4353	south-coast	2011	12	1	335	136	14.5	40	50	64	53.3
4354	south-coast	2011	12	2	336	126	20.1	37	41	65	62.4
4355	south-coast	2011	12	3	337	128	15.8	44	37	64	50.1
4356	south-coast	2011	12	4	338	147	22.9	40	33	64	47.3
4357	south-coast	2011	12	5	339	163	20.2	42	35	64	45.5
4358	south-coast	2011	12	6	340	145	19.7	41	30	65	45.3
4359	south-coast	2011	12	7	341	166	17.1	40	33	71	45.4
4360	south-coast	2011	12	8	342	154	27.4	35	36	72	67
4361	south-coast	2011	12	9	343	157	35	40	38	74	70.3
4362	south-coast	2011	12	10	344	159	31	45	38	75	68.9
4363	south-coast	2011	12	11	345	162	36.8	46	36	65	79
4364	south-coast	2011	12	12	346	144	20.1	41	45	57	99
4365	south-coast	2011	12	13	347	155	14.7	35	39	61	100
4366	south-coast	2011	12	14	348	154	25.7	40	39	61	89.8
4367	south-coast	2011	12	15	349	152	31.9	40	36	61	95.3
4368	south-coast	2011	12	16	350	140	20.4	46	37	68	62.1
4369	south-coast	2011	12	17	351	147	17.4	42	49	68	63.4
4370	south-coast	2011	12	18	352	173	22.5	35	39	62	95.7
4371	south-coast	2011	12	19	353	148	27.5	37	44	60	94.8
4372	south-coast	2011	12	20	354	116	25.8	37	38	66	75
4373	south-coast	2011	12	21	355	153	26.7	35	36	64	81.9
4374	south-coast	2011	12	22	356	157	18.5	43	38	64	52
4375	south-coast	2011	12	23	357	152	20.6	41	39	70	42.4
4376	south-coast	2011	12	24	358	167	37.3	41	37	75	42.1
4377	south-coast	2011	12	25	359	161	42	41	36	75	47.4
4378	south-coast	2011	12	26	360	150	35.3	38	38	75	57
4379	south-coast	2011	12	27	361	150	35.4	39	38	76	73.5
4380	south-coast	2011	12	28	362	189	31	36	41	81	57.8
4381	south-coast	2011	12	29	363	140	37.7	33	45	79	73.3
4382	south-coast	2011	12	30	364	154	55.3	39	45	78	93.3
4383	south-coast	2011	12	31	365	164	63.4	42	44	86	86.5
4384	south-coast	2012	1	1	1	165	50.2	42	43	83	79.5
4385	south-coast	2012	1	2	2	153	32.9	41	45	86	79.3
4386	south-coast	2012	1	3	3	145	27.9	35	48	86	60
4387	south-coast	2012	1	4	4	156	26.1	40	48	87	57.6
4388	south-coast	2012	1	5	5	160	17.7	40	48	85	40
4389	south-coast	2012	1	6	6	177	28	47	46	79	68.8
4390	south-coast	2012	1	7	7	139	41	48	41	71	89.4
4391	south-coast	2012	1	8	8	135	28	43	47	74	56.9
4392	south-coast	2012	1	9	9	141	17.8	39	44	76	48.6
4393	south-coast	2012	1	10	10	150	21.9	38	42	72	63.9
4394	south-coast	2012	1	11	11	158	32.1	34	43	73	72.3
4395	south-coast	2012	1	12	12	157	32.8	44	46	76	66.5
4396	south-coast	2012	1	13	13	164	17.7	44	40	78	33

4397	south-coast	2012	1	14	14	163	26.5	38	39	74	45.5
4398	south-coast	2012	1	15	15	185	26.7	43	40	67	66.9
4399	south-coast	2012	1	16	16	145	91.6	40	40	60	96
4400	south-coast	2012	1	17	17	139	27	42	35	60	79.6
4401	south-coast	2012	1	18	18	151	28.5	40	34	69	74.1
4402	south-coast	2012	1	19	19	152	36.4	39	36	71	78.2
4403	south-coast	2012	1	20	20	161	37.5	43	40	63	81.9
4404	south-coast	2012	1	21	21	161	21.1	39	46	64	93
4405	south-coast	2012	1	22	22	152	22	36	41	64	89.6
4406	south-coast	2012	1	23	23	140	19.7	36	41	60	99
4407	south-coast	2012	1	24	24	158	19.5	41	43	69	78
4408	south-coast	2012	1	25	25	149	15.9	36	45	82	55.8
4409	south-coast	2012	1	26	26	166	19.7	38	47	83	62.4
4410	south-coast	2012	1	27	27	136	16.3	45	48	80	56.4
4411	south-coast	2012	1	28	28	158	19.6	46	38	77	39
4412	south-coast	2012	1	29	29	156	12	44	40	80	33.4
4413	south-coast	2012	1	30	30	156	22	49	40	80	67.4
4414	south-coast	2012	1	31	31	145	26.3	49	38	72	77.2
4415	south-coast	2012	2	1	32	160	32.5	44	41	70	78.7
4416	south-coast	2012	2	2	33	156	36.6	46	40	74	80.8
4417	south-coast	2012	2	3	34	164	19.3	45	37	72	47.8
4418	south-coast	2012	2	4	35	157	10.6	48	39	71	51.1
4419	south-coast	2012	2	5	36	148	12.3	50	40	73	49.3
4420	south-coast	2012	2	6	37	160	16.2	49	40	72	53.8
4421	south-coast	2012	2	7	38	164	15.9	48	49	69	75.8
4422	south-coast	2012	2	8	39	145	15.8	47	43	76	62.7
4423	south-coast	2012	2	9	40	163	13.3	45	45	81	52.6
4424	south-coast	2012	2	10	41	148	17.4	51	45	78	76
4425	south-coast	2012	2	11	42	150	17.7	42	45	72	94.9
4426	south-coast	2012	2	12	43	132	15.4	42	48	64	99.7
4427	south-coast	2012	2	13	44	143	11.9	41	49	59	100
4428	south-coast	2012	2	14	45	145	13.9	40	38	62	91.4
4429	south-coast	2012	2	15	46	136	14	39	45	58	100
4430	south-coast	2012	2	16	47	143	12.2	45	42	69	70.5
4431	south-coast	2012	2	17	48	163	12.4	43	40	73	54.5
4432	south-coast	2012	2	18	49	143	18.5	46	39	69	80.8
4433	south-coast	2012	2	19	50	160	19.8	45	47	62	95.3
4434	south-coast	2012	2	20	51	150	23.8	44	41	64	84.4
4435	south-coast	2012	2	21	52	150	17.4	47	39	75	67.4
4436	south-coast	2012	2	22	53	173	18.1	45	48	84	65.4
4437	south-coast	2012	2	23	54	165	20.2	51	46	85	71
4438	south-coast	2012	2	24	55	152	43.3	47	47	84	82.7
4439	south-coast	2012	2	25	56	146	40.1	56	49	73	80
4440	south-coast	2012	2	26	57	152	33.7	58	44	68	82
4441	south-coast	2012	2	27	58	147	14	45	36	64	99.5
4442	south-coast	2012	2	28	59	147	13.1	43	38	57	96.8
4443	south-coast	2012	2	29	60	168	15.9	45	36	60	93.1
4444	south-coast	2012	3	1	61	183	87.7	47	48	64	89
4445	south-coast	2012	3	2	62	141	14.7	50	40	72	58.8

4446	south-coast	2012	3	3	63	156	11.5	52	47	80	36.3
4447	south-coast	2012	3	4	64	164	13.9	51	48	90	47.1
4448	south-coast	2012	3	5	65	176	15.7	48	47	89	53.3
4449	south-coast	2012	3	6	66	161	18.4	49	44	80	75
4450	south-coast	2012	3	7	67	159	10.1	50	41	66	44
4451	south-coast	2012	3	8	68	161	11.8	54	39	77	47.3
4452	south-coast	2012	3	9	69	145	12.8	57	42	83	33.1
4453	south-coast	2012	3	10	70	180	17.3	64	44	81	64
4454	south-coast	2012	3	11	71	147	19.3	59	48	75	75.9
4455	south-coast	2012	3	12	72	176	22.7	55	41	75	77.5
4456	south-coast	2012	3	13	73	147	25.8	50	43	70	80.9
4457	south-coast	2012	3	14	74	158	19.5	46	42	72	79.3
4458	south-coast	2012	3	15	75	166	25.3	50	43	74	81.5
4459	south-coast	2012	3	16	76	152	27.5	42	50	68	94
4460	south-coast	2012	3	17	77	144	9.7	48	43	62	100
4461	south-coast	2012	3	18	78	152	8.7	45	40	56	100
4462	south-coast	2012	3	19	79	158	13	50	37	61	97.3
4463	south-coast	2012	3	20	80	146	20	56	38	71	70.7
4464	south-coast	2012	3	21	81	143	51.6	63	43	79	73.5
4465	south-coast	2012	3	22	82	153	28.9	65	47	80	83.3
4466	south-coast	2012	3	23	83	161	25.9	39	45	73	85.1
4467	south-coast	2012	3	24	84	150	23.3	44	49	69	79.4
4468	south-coast	2012	3	25	85	147	13.6	50	47	68	90.7
4469	south-coast	2012	3	26	86	166	11.7	54	43	71	92.3
4470	south-coast	2012	3	27	87	156	21.3	55	41	68	75.8
4471	south-coast	2012	3	28	88	132	15.3	50	41	69	81
4472	south-coast	2012	3	29	89	142	33.9	52	49	75	82.3
4473	south-coast	2012	3	30	90	143	44.5	69	48	75	85.1
4474	south-coast	2012	3	31	91	122	27.1	60	48	71	97.4
4475	south-coast	2012	4	1	92	126	11	52	42	73	69.9
4476	south-coast	2012	4	2	93	143	8.3	55	42	78	47
4477	south-coast	2012	4	3	94	156	14.8	66	46	79	63.8
4478	south-coast	2012	4	4	95	168	17.8	70	47	79	74.8
4479	south-coast	2012	4	5	96	144	18.2	61	44	75	72
4480	south-coast	2012	4	6	97	130	12.8	62	39	76	53.7
4481	south-coast	2012	4	7	98	162	16.3	71	42	86	33.5
4482	south-coast	2012	4	8	99	140	21.7	80	47	88	69.3
4483	south-coast	2012	4	9	100	163	19.5	67	47	89	74.3
4484	south-coast	2012	4	10	101	140	19.1	58	44	79	72.8
4485	south-coast	2012	4	11	102	158	9.5	49	49	67	100
4486	south-coast	2012	4	12	103	160	9.7	52	47	65	96.4
4487	south-coast	2012	4	13	104	157	10.6	53	48	60	100
4488	south-coast	2012	4	14	105	143	10.3	51	43	63	97.7
4489	south-coast	2012	4	15	106	143	13	57	39	72	78.5
4490	south-coast	2012	4	16	107	146	15.3	68	46	83	77.7
4491	south-coast	2012	4	17	108	165	16.3	72	51	86	73.7
4492	south-coast	2012	4	18	109	133	19.4	66	52	86	77.6
4493	south-coast	2012	4	19	110	166	26	71	53	87	79.9
4494	south-coast	2012	4	20	111	155	37.5	67	55	99	84.2

4495	south-coast	2012	4	21	112	148	49.8	97	57	97	85.1
4496	south-coast	2012	4	22	113	144	38.8	90	56	92	84.7
4497	south-coast	2012	4	23	114	137	17.3	61	55	74	99
4498	south-coast	2012	4	24	115	156	18.3	47	52	77	73.8
4499	south-coast	2012	4	25	116	146	21	50	57	91	73.8
4500	south-coast	2012	4	26	117	122	12.1	47	54	80	100
4501	south-coast	2012	4	27	118	128	17.8	53	54	76	75.3
4502	south-coast	2012	4	28	119	150	23.1	78	49	90	73.8
4503	south-coast	2012	4	29	120	149	26.1	78	55	85	81.2
4504	south-coast	2012	4	30	121	144	29.5	72	58	80	86
4505	south-coast	2012	5	1	122	128	20.3	49	55	65	99.4
4506	south-coast	2012	5	2	123	122	15.4	50	53	66	100
4507	south-coast	2012	5	3	124	130	23.1	51	55	70	97.2
4508	south-coast	2012	5	4	125	133	27.9	63	52	75	82.2
4509	south-coast	2012	5	5	126	129	26.3	74	49	83	77.1
4510	south-coast	2012	5	6	127	133	26	71	50	80	80.7
4511	south-coast	2012	5	7	128	148	28.9	67	50	91	81
4512	south-coast	2012	5	8	129	130	31.6	69	52	93	79.4
4513	south-coast	2012	5	9	130	136	29	86	54	95	78.8
4514	south-coast	2012	5	10	131	138	29.5	79	54	88	82.6
4515	south-coast	2012	5	11	132	118	33.2	71	53	82	74.2
4516	south-coast	2012	5	12	133	126	32.8	102	53	88	79.8
4517	south-coast	2012	5	13	134	138	32.9	99	51	90	81
4518	south-coast	2012	5	14	135	140	25.6	86	52	89	74.1
4519	south-coast	2012	5	15	136	142	23.4	77	50	87	72.8
4520	south-coast	2012	5	16	137	104	27.9	98	55	96	74.8
4521	south-coast	2012	5	17	138	131	32.4	96	52	91	80.4
4522	south-coast	2012	5	18	139	130	27.2	66	53	79	77.3
4523	south-coast	2012	5	19	140	135	32.3	90	52	89	79.5
4524	south-coast	2012	5	20	141	124	36	106	54	99	78.9
4525	south-coast	2012	5	21	142	140	36.7	100	55	97	78.3
4526	south-coast	2012	5	22	143	131	34.3	103	55	93	80.5
4527	south-coast	2012	5	23	144	143	28.9	83	56	82	81.5
4528	south-coast	2012	5	24	145	127	21.5	70	55	78	82
4529	south-coast	2012	5	25	146	137	12	54	49	71	99.6
4530	south-coast	2012	5	26	147	104	9.1	52	48	68	91.9
4531	south-coast	2012	5	27	148	136	16.5	66	46	82	70.9
4532	south-coast	2012	5	28	149	140	18.2	82	53	90	73.8
4533	south-coast	2012	5	29	150	145	20.1	81	55	90	73.2
4534	south-coast	2012	5	30	151	139	21	75	54	87	76.9
4535	south-coast	2012	5	31	152	126	35.2	91	55	98	76.6
4536	south-coast	2012	6	1	153	142	40.7	96	59	98	83.2
4537	south-coast	2012	6	2	154	128	34.8	96	56	90	83
4538	south-coast	2012	6	3	155	135	22.5	85	58	88	79.7
4539	south-coast	2012	6	4	156	136	22.7	62	58	83	88
4540	south-coast	2012	6	5	157	116	18.4	67	59	79	82
4541	south-coast	2012	6	6	158	128	20.6	74	52	88	65.9
4542	south-coast	2012	6	7	159	130	25.4	79	54	95	73.5
4543	south-coast	2012	6	8	160	113	24	87	57	90	79.7

4544	south-coast	2012	6	9	161	119	29.5	78	55	82	78.7
4545	south-coast	2012	6	10	162	132	26.9	84	53	86	76.3
4546	south-coast	2012	6	11	163	117	29	90	53	91	78.7
4547	south-coast	2012	6	12	164	126	32.3	95	56	94	79.9
4548	south-coast	2012	6	13	165	130	30.8	91	56	92	80.6
4549	south-coast	2012	6	14	166	120	26.5	77	58	87	78.2
4550	south-coast	2012	6	15	167	116	29.1	72	58	81	81
4551	south-coast	2012	6	16	168	145	31.2	88	55	93	79.3
4552	south-coast	2012	6	17	169	127	25.8	99	58	100	79.6
4553	south-coast	2012	6	18	170	121	35	73	58	94	82.5
4554	south-coast	2012	6	19	171	139	25.3	82	57	89	79.6
4555	south-coast	2012	6	20	172	120	30.7	102	57	92	78
4556	south-coast	2012	6	21	173	136	31.9	96	58	90	83
4557	south-coast	2012	6	22	174	139	28.9	80	57	89	76.3
4558	south-coast	2012	6	23	175	131	18.6	71	56	89	73.5
4559	south-coast	2012	6	24	176	126	14.6	65	55	91	71.3
4560	south-coast	2012	6	25	177	100	12.7	71	55	91	73.3
4561	south-coast	2012	6	26	178	138	13.3	70	54	94	72
4562	south-coast	2012	6	27	179	129	18.3	81	55	96	74.3
4563	south-coast	2012	6	28	180	131	17.8	84	58	98	81.4
4564	south-coast	2012	6	29	181	138	21.7	84	58	96	77.9
4565	south-coast	2012	6	30	182	121	16.5	90	58	95	76.4
4566	south-coast	2012	7	1	183	140	18.1	83	59	92	76.3
4567	south-coast	2012	7	2	184	136	17.5	75	59	92	84.8
4568	south-coast	2012	7	3	185	119	22.6	81	61	87	84
4569	south-coast	2012	7	4	186	129	35.8	78	60	86	86.8
4570	south-coast	2012	7	5	187	127	41.3	69	59	87	84
4571	south-coast	2012	7	6	188	111	28.6	83	59	93	78.3
4572	south-coast	2012	7	7	189	142	23.8	89	59	98	79.5
4573	south-coast	2012	7	8	190	129	21.5	87	57	100	78
4574	south-coast	2012	7	9	191	133	19.4	105	58	105	77.3
4575	south-coast	2012	7	10	192	138	24	107	62	106	77.3
4576	south-coast	2012	7	11	193	117	31.6	112	62	107	80.3
4577	south-coast	2012	7	12	194	138	31.1	57	62	94	81.3
4578	south-coast	2012	7	13	195	123	41	72	65	93	77.8
4579	south-coast	2012	7	14	196	128	31.1	85	63	97	81.3
4580	south-coast	2012	7	15	197	126	25.2	71	62	95	75
4581	south-coast	2012	7	16	198	129	19	57	56	89	73.5
4582	south-coast	2012	7	17	199	134	25.5	64	58	88	69.5
4583	south-coast	2012	7	18	200	127	15.8	67	58	95	74.4
4584	south-coast	2012	7	19	201	132	15.9	77	64	102	69.5
4585	south-coast	2012	7	20	202	115	18.8	83	64	100	72.1
4586	south-coast	2012	7	21	203	158	26.2	94	63	100	78.1
4587	south-coast	2012	7	22	204	130	25.5	91	64	98	79.6
4588	south-coast	2012	7	23	205	124	23.5	83	62	98	81.9
4589	south-coast	2012	7	24	206	146	25.5	81	59	94	78.7
4590	south-coast	2012	7	25	207	142	23.4	78	57	94	78.7
4591	south-coast	2012	7	26	208	127	24.4	84	57	94	76.4
4592	south-coast	2012	7	27	209	123	26	85	57	96	77.7

4593	south-coast	2012	7	28	210	142	22.3	96	55	91	76.5
4594	south-coast	2012	7	29	211	140	23.9	102	59	96	77.3
4595	south-coast	2012	7	30	212	105	24.9	85	62	96	77.5
4596	south-coast	2012	7	31	213	128	26.7	81	63	94	79.8
4597	south-coast	2012	8	1	214	115	23.9	88	57	95	78.8
4598	south-coast	2012	8	2	215	115	22.5	104	58	97	77.1
4599	south-coast	2012	8	3	216	137	24.5	90	58	89	78.5
4600	south-coast	2012	8	4	217	136	27.6	97	58	92	82.8
4601	south-coast	2012	8	5	218	132	25.2	108	58	102	77.9
4602	south-coast	2012	8	6	219	128	22.2	91	63	109	75.1
4603	south-coast	2012	8	7	220	131	25.3	100	66	107	71.2
4604	south-coast	2012	8	8	221	123	28.1	109	66	108	72
4605	south-coast	2012	8	9	222	134	26.1	106	65	109	73
4606	south-coast	2012	8	10	223	114	27.9	102	66	108	74.4
4607	south-coast	2012	8	11	224	137	29	111	69	105	74.5
4608	south-coast	2012	8	12	225	143	22.1	109	67	107	74
4609	south-coast	2012	8	13	226	126	22.7	103	66	107	75.8
4610	south-coast	2012	8	14	227	142	22.4	97	63	106	76.3
4611	south-coast	2012	8	15	228	143	21.7	82	66	98	78.2
4612	south-coast	2012	8	16	229	144	25	85	66	105	72.9
4613	south-coast	2012	8	17	230	136	27.2	87	69	100	70.6
4614	south-coast	2012	8	18	231	148	22.1	84	68	99	70.9
4615	south-coast	2012	8	19	232	145	24.6	96	66	100	67.8
4616	south-coast	2012	8	20	233	139	22.9	85	66	100	74.9
4617	south-coast	2012	8	21	234	142	26.5	73	69	96	82.7
4618	south-coast	2012	8	22	235	147	22.6	67	61	93	82.3
4619	south-coast	2012	8	23	236	134	28.4	76	60	92	83.8
4620	south-coast	2012	8	24	237	128	28	70	60	92	83.8
4621	south-coast	2012	8	25	238	128	32.1	85	57	89	80.8
4622	south-coast	2012	8	26	239	110	30.7	81	56	94	74.1
4623	south-coast	2012	8	27	240	125	22.2	78	60	102	72.3
4624	south-coast	2012	8	28	241	132	18.6	74	66	104	73.3
4625	south-coast	2012	8	29	242	122	23.9	89	69	105	79.1
4626	south-coast	2012	8	30	243	129	26.6	75	67	95	75.6
4627	south-coast	2012	8	31	244	139	25.4	64	65	97	73.1
4628	south-coast	2012	9	1	245	152	20.2	79	61	97	71.7
4629	south-coast	2012	9	2	246	112	18	88	60	100	73
4630	south-coast	2012	9	3	247	141	22.6	99	62	103	78
4631	south-coast	2012	9	4	248	136	20.3	74	65	100	74.6
4632	south-coast	2012	9	5	249	130	20.4	59	68	91	70.3
4633	south-coast	2012	9	6	250	127	20.9	79	64	93	69.4
4634	south-coast	2012	9	7	251	125	19.7	77	65	93	75.4
4635	south-coast	2012	9	8	252	133	22.3	101	64	101	71.9
4636	south-coast	2012	9	9	253	128	24.7	87	67	102	72.3
4637	south-coast	2012	9	10	254	130	25.6	58	67	90	84.3
4638	south-coast	2012	9	11	255	139	23.1	49	64	87	99.1
4639	south-coast	2012	9	12	256	133	28.9	71	63	94	87
4640	south-coast	2012	9	13	257	158	25.2	80	61	105	80.7
4641	south-coast	2012	9	14	258	126	18.2	70	66	105	68.9

4642	south-coast	2012	9	15	259	142	17.3	80	66	103	58.2
4643	south-coast	2012	9	16	260	143	22.9	84	64	96	83.5
4644	south-coast	2012	9	17	261	141	29.5	78	61	93	79.2
4645	south-coast	2012	9	18	262	126	30.1	82	61	95	75.4
4646	south-coast	2012	9	19	263	124	24	74	60	99	73.5
4647	south-coast	2012	9	20	264	132	28.4	84	64	102	73.1
4648	south-coast	2012	9	21	265	133	32.6	85	63	101	75.2
4649	south-coast	2012	9	22	266	112	33.8	92	67	98	76.2
4650	south-coast	2012	9	23	267	142	25	81	67	101	75.5
4651	south-coast	2012	9	24	268	120	19.5	52	57	91	74.9
4652	south-coast	2012	9	25	269	134	22.5	58	57	90	77.3
4653	south-coast	2012	9	26	270	115	28.5	64	56	89	80.2
4654	south-coast	2012	9	27	271	127	30.5	61	59	95	78.1
4655	south-coast	2012	9	28	272	116	27.7	54	58	95	77.7
4656	south-coast	2012	9	29	273	143	25.8	75	54	95	83.8
4657	south-coast	2012	9	30	274	138	23.4	87	63	104	74.1
4658	south-coast	2012	10	1	275	138	23.2	72	63	107	72.3
4659	south-coast	2012	10	2	276	114	25.4	80	66	105	70.8
4660	south-coast	2012	10	3	277	134	29.9	59	62	96	80
4661	south-coast	2012	10	4	278	128	30.1	60	57	88	77.7
4662	south-coast	2012	10	5	279	116	33.8	68	58	85	79.2
4663	south-coast	2012	10	6	280	145	31.8	91	60	82	77.5
4664	south-coast	2012	10	7	281	144	25.5	67	61	84	80.3
4665	south-coast	2012	10	8	282	123	29	56	52	82	82.6
4666	south-coast	2012	10	9	283	129	24.9	52	59	79	81.7
4667	south-coast	2012	10	10	284	133	14.3	43	59	76	92.8
4668	south-coast	2012	10	11	285	134	13.4	36	55	72	97.3
4669	south-coast	2012	10	12	286	139	16.4	43	50	71	100
4670	south-coast	2012	10	13	287	108	26.1	55	49	80	76.4
4671	south-coast	2012	10	14	288	143	19.1	56	50	95	66.1
4672	south-coast	2012	10	15	289	147	16.9	55	59	96	58.2
4673	south-coast	2012	10	16	290	142	30.4	71	58	96	74.6
4674	south-coast	2012	10	17	291	117	22.9	80	59	100	61.6
4675	south-coast	2012	10	18	292	136	15.5	48	62	99	76.5
4676	south-coast	2012	10	19	293	150	20.6	59	56	85	84.8
4677	south-coast	2012	10	20	294	119	21.9	58	60	78	93.5
4678	south-coast	2012	10	21	295	104	10.9	46	61	75	100
4679	south-coast	2012	10	22	296	148	13	48	59	74	97.4
4680	south-coast	2012	10	23	297	121	11.8	48	55	72	90.8
4681	south-coast	2012	10	24	298	133	12.2	48	47	75	69.6
4682	south-coast	2012	10	25	299	169	14.8	54	46	82	56.4
4683	south-coast	2012	10	26	300	139	13.5	53	50	87	38.5
4684	south-coast	2012	10	27	301	150	11.6	57	48	93	34.2
4685	south-coast	2012	10	28	302	124	17.3	65	52	92	62.8
4686	south-coast	2012	10	29	303	149	28.5	72	51	89	77.8
4687	south-coast	2012	10	30	304	135	35.1	77	50	87	82.8
4688	south-coast	2012	10	31	305	129	37.3	72	49	80	78.4
4689	south-coast	2012	11	1	306	138	40	63	47	72	87.1
4690	south-coast	2012	11	2	307	156	41.5	54	52	71	80.8

4691	south-coast	2012	11	3	308	157	43.5	57	47	85	81.1
4692	south-coast	2012	11	4	309	143	21.7	56	51	92	53.9
4693	south-coast	2012	11	5	310	141	16.6	49	54	94	34.8
4694	south-coast	2012	11	6	311	143	23.9	58	54	88	73.3
4695	south-coast	2012	11	7	312	146	50.8	61	50	82	85.1
4696	south-coast	2012	11	8	313	146	21.8	45	53	66	93.3
4697	south-coast	2012	11	9	314	137	11.1	44	49	63	98.8
4698	south-coast	2012	11	10	315	153	12.3	44	41	62	72.7
4699	south-coast	2012	11	11	316	127	27.4	46	35	67	46.3
4700	south-coast	2012	11	12	317	153	16	47	38	73	41.8
4701	south-coast	2012	11	13	318	160	15.2	49	41	82	42.3
4702	south-coast	2012	11	14	319	132	20.7	48	45	82	70.5
4703	south-coast	2012	11	15	320	153	26.3	40	50	73	85.3
4704	south-coast	2012	11	16	321	160	23.8	46	51	74	79.3
4705	south-coast	2012	11	17	322	175	14.3	33	54	68	100
4706	south-coast	2012	11	18	323	155	13.8	41	53	69	99.4
4707	south-coast	2012	11	19	324	144	17.4	43	46	71	89.2
4708	south-coast	2012	11	20	325	127	23.7	46	43	77	79.8
4709	south-coast	2012	11	21	326	133	31.1	45	45	73	83.5
4710	south-coast	2012	11	22	327	148	40.9	55	42	81	83.4
4711	south-coast	2012	11	23	328	139	36.4	50	49	85	87.9
4712	south-coast	2012	11	24	329	166	23.2	47	49	87	82.6
4713	south-coast	2012	11	25	330	144	30.2	56	45	83	90.5
4714	south-coast	2012	11	26	331	156	41.2	43	46	73	88.3
4715	south-coast	2012	11	27	332	150	35.5	46	46	78	83.6
4716	south-coast	2012	11	28	333	142	39.1	46	46	68	87.5
4717	south-coast	2012	11	29	334	142	20.8	28	53	68	100
4718	south-coast	2012	11	30	335	154	12.2	23	56	68	99.8
4719	south-coast	2012	12	1	336	141	16.4	36	53	67	100
4720	south-coast	2012	12	2	337	152	12.9	37	55	66	100
4721	south-coast	2012	12	3	338	155	12.4	36	56	68	100
4722	south-coast	2012	12	4	339	149	33	37	52	72	89.6
4723	south-coast	2012	12	5	340	145	38.1	35	41	75	91.5
4724	south-coast	2012	12	6	341	152	44.5	32	46	68	93.2
4725	south-coast	2012	12	7	342	131	53	38	46	80	89.7
4726	south-coast	2012	12	8	343	134	59.3	41	43	72	88.3
4727	south-coast	2012	12	9	344	144	79	49	43	72	84.3
4728	south-coast	2012	12	10	345	124	38.5	42	44	77	77
4729	south-coast	2012	12	11	346	128	28.5	42	42	74	80.9
4730	south-coast	2012	12	12	347	163	24	50	40	68	87.4
4731	south-coast	2012	12	13	348	134	10.3	42	48	61	100
4732	south-coast	2012	12	14	349	146	14.8	42	41	58	99.7
4733	south-coast	2012	12	15	350	161	13	42	42	60	88.4
4734	south-coast	2012	12	16	351	151	13.2	43	46	58	99
4735	south-coast	2012	12	17	352	122	19.1	41	47	65	100
4736	south-coast	2012	12	18	353	146	15	40	51	60	95.2
4737	south-coast	2012	12	19	354	143	10.6	43	39	59	50.4
4738	south-coast	2012	12	20	355	139	17.7	41	31	66	69.7
4739	south-coast	2012	12	21	356	140	24.8	40	33	66	54.8

4740	south-coast	2012	12	22	357	170	31.4	45	35	63	79.1
4741	south-coast	2012	12	23	358	187	22.2	43	42	62	98.7
4742	south-coast	2012	12	24	359	157	22.3	39	48	62	99.9
4743	south-coast	2012	12	25	360	141	40.6	40	42	61	80.5
4744	south-coast	2012	12	26	361	134	23.3	42	41	61	97.2
4745	south-coast	2012	12	27	362	136	18	40	39	62	93.1
4746	south-coast	2012	12	28	363	131	17.9	41	34	62	67.8
4747	south-coast	2012	12	29	364	165	22.8	40	36	61	82.8
4748	south-coast	2012	12	30	365	153	28.3	38	38	56	93.5
4749	south-coast	2012	12	31	366	182	38.9	39	33	59	76.8
