# Supplementary material for: Time series smoother for effect detection
Source: PLoS One. 2018 Apr 23;13(4):e0195360. doi: 10.1371/journal.pone.0195360 (PMC5912770; doi:10.1371/journal.pone.0195360)
Supplement: S1 Appendix — This appendix contains the summaries of the coefficient estimates, standard errors, t statistics and 2-sided p-values of the linear model with main effects and interactions for correlation and partial correlation. (ZIP) [file pone.0195360.s002.zip › SupportingInformation.pdf]

Supporting Information

S1 Dataset Description

The following is the detailed description of the dataset of Los Angeles California from Year 2000 to Year 2012.

- RowID: the number of data entry counting from Jan 1 2000 till Dec 31 2012.
- basin: the air basin south coast in Los Angeles California.
- year: the year of the data entry.
- month: the month of the data entry.
- day: the day of the data entry.
- dayofyear: the day of the year of the data entry.
- AllCause75: the daily number of mortality at Age 75 and above, excluding accidental deaths.
- PM25davg: the daily average level of $PM_{2.5}$ in microgram per cubic meter (μgm^{-3}).
- o3: the daily average level of ozone in parts per billion (ppb).
- tmin.0: the daily minimum temperatures are recorded in Fahrenheit (^oF).
- tmax.0: the daily maximum temperatures are recorded in Fahrenheit (^oF).
- MAXRH.0: the daily maximum relative humidity level in percentages of air-water mixture.

S1 Appendix

The tables below contain the detailed information by moving trimmed mean for Fig 3 in Section Case Study. The rows represent the predictors and the columns represent the coefficient estimates, standard errors, t statistics and 2-sided p-values of the linear model with main effects and interactions. The responses are correlation and partial correlation, respectively. The predictors are window size, gap size, type and trimming percentage lambda specified in the factorial design of scenarios.

Summary of Correlation versus Variables and Interactions

	Estimate	Std. Error	t value	p-value
(Intercept)	0.0640	0.0019	34.58	0.0000
Window	0.0004	0.0000	7.57	0.0000
Gap	0.0031	0.0003	12.34	0.0000
Lambda	0.0037	0.0036	1.01	0.3143
TypeDR	-0.0298	0.0021	-14.11	0.0000
TypeRD	-0.0225	0.0021	-10.67	0.0000
Window:Gap	-0.0001	0.0000	-10.90	0.0000
Window:Lambda	-0.0002	0.0001	-2.57	0.0107
Window:TypeDR	-0.0004	0.0001	-8.52	0.0000
Window:TypeRD	-0.0005	0.0001	-9.15	0.0000
Gap:Lambda	0.0000	0.0003	0.04	0.9683
Gap:TypeDR	-0.0006	0.0002	-3.56	0.0004
Gap:TypeRD	-0.0010	0.0002	-5.56	0.0000
Lambda:TypeDR	-0.0204	0.0029	-6.94	0.0000
Lambda:TypeRD	-0.0192	0.0029	-6.54	0.0000

Summary of Partial Correlation versus Variables and Interactions

	Estimate	Std. Error	t value	p-value
(Intercept)	0.0426	0.0020	21.10	0.0000
Window	0.0002	0.0001	3.98	0.0001
Gap	0.0024	0.0003	8.47	0.0000
Lambda	0.0065	0.0040	1.62	0.1057
TypeDR	-0.0718	0.0023	-31.15	0.0000
TypeRD	0.0493	0.0023	21.39	0.0000
Window:Gap	-0.0000	0.0000	-7.42	0.0000
Window:Lambda	-0.0002	0.0001	-2.46	0.0144
Window:TypeDR	0.0001	0.0001	2.12	0.0345
Window:TypeRD	0.0007	0.0001	12.43	0.0000
Gap:Lambda	0.0003	0.0003	0.80	0.4218
Gap:TypeDR	-0.0005	0.0002	-2.75	0.0064
Gap:TypeRD	0.0002	0.0002	0.81	0.4166
Lambda:TypeDR	-0.0126	0.0032	-3.93	0.0001
Lambda:TypeRD	-0.0260	0.0032	-8.10	0.0000
